# Asian Reference Values for Handgrip Strength, Gait Speed, Five‐Times‐Sit‐to‐Stand Test, Muscle Mass and Calf Circumference

**DOI:** 10.1002/jcsm.70216

**Published:** 2026-02-11

**Authors:** Jozo Grgic, Siew Ling Tey, Dieu Thi Thu Huynh, Yen Ling Low, Zeljko Pedisic, Nina Schaller, Vanessa Kristina Wazny, Weilan Wang, Yasuhiko Saito, Ravindra P. Rannan‐Eliya, Hala Ghattas, Monique Chaaya, Carlos Mendes de Leon, Preeti Gupta, Ecosse L. Lamoureux, Mythily Subramaniam, Edimansyah Abdin, Rahul Malhotra, Angelique Chan, Bayasgalan Tumenbayar, Oyunbileg Luvsandavaajav, Bolormaa Enkhtuvshin, Norma Mansor, Halimah Awang, Andrea B. Maier

**Affiliations:** ^1^ NUS Academy for Healthy Longevity, Yong Loo Lin School of Medicine National University of Singapore Singapore Singapore; ^2^ Department of Sports Science and Physical Education The Chinese University of Hong Kong Hong Kong, China; ^3^ Abbott Nutrition Research and Development, Asia‐Pacific Centre Singapore Singapore; ^4^ School of Public Health, Li Ka Shing Faculty of Medicine The University of Hong Kong Hong Kong, China; ^5^ Department for Preventive Sports Medicine and Sports Cardiology, TUM School of Medicine and Health, TUM University Hospital Technical University of Munich (TUM) Munich Germany; ^6^ College of Economics Nihon University Chiyoda Japan; ^7^ Economic Research Institute for ASEAN and East Asia Jakarta Indonesia; ^8^ Institute for Health Policy Colombo Sri Lanka; ^9^ Department of Health Promotion, Education, and Behavior, Arnold School of Public Health University of South Carolina Columbia South Carolina USA; ^10^ Department of Epidemiology and Population Health, Faculty of Health Sciences American University of Beirut Beirut Lebanon; ^11^ Department of Global Health Georgetown University School of Health Washington District of Columbia USA; ^12^ Singapore Eye Research Institute, Singapore National Eye Centre Singapore Singapore; ^13^ Duke‐NUS Medical School Singapore Singapore; ^14^ Department of Ophthalmology The University of Melbourne Melbourne Victoria Australia; ^15^ Research Division Institute of Mental Health Singapore Singapore; ^16^ Saw Swee Hock School of Public Health National University Singapore Singapore Singapore; ^17^ Centre for Ageing Research & Education (CARE), Duke‐NUS Medical School Singapore Singapore; ^18^ Programme in Health Services and Systems Research (HSSR), Duke‐NUS Medical School Singapore Singapore; ^19^ Postgraduate Training Institute Mongolian National University of Medical Sciences Ulaanbaatar Mongolia; ^20^ Graduate School of Public Health Inje University Republic of Korea; ^21^ Intermed Hospital Ulaanbaatar Mongolia; ^22^ Social Wellbeing Research Centre Universiti Malaya Kuala Lumpur Malaysia; ^23^ Healthy Longevity Translational Research Program, Yong Loo Lin School of Medicine National University of Singapore Singapore; ^24^ Department of Human Movement Sciences, @AgeAmsterdam, Faculty of Behavioural and Movement Sciences, Vrije Universiteit Amsterdam Amsterdam Movement Sciences Amsterdam the Netherlands

**Keywords:** centile, data synthesis, normative, norms

## Abstract

**Background:**

Handgrip strength, gait speed, Five‐Times‐Sit‐to‐Stand Test (FTSST) performance, skeletal muscle mass and calf circumference are important health outcomes, particularly useful for diagnosing sarcopenia. The aim of this study was to establish reference (normative) values for handgrip strength, gait speed, FTSST performance, skeletal muscle mass and calf circumference by pooling individual participant data from national cohorts in Asia.

**Methods:**

We conducted a pooled analysis of individual participant data from 20 national cohorts representing 12 Asian countries (China, India, Indonesia, Israel, Japan, Lebanon, Malaysia, Mongolia, Philippines, Republic of Korea, Singapore and Sri Lanka). We included cohorts with data on at least one relevant outcome collected among community‐dwelling individuals aged 20 years and older (up to 100+ years). Data were harmonized and stratified by sex and 5‐year age groups with the 5th, 10th, 20th, 30th, 40th, 50th, 60th, 70th, 80th, 90th and 95th percentile calculated for each outcome. Pan‐Asian, regional (East, South, Southeast and West Asia) and country‐specific reference values were calculated.

**Results:**

The number of participants in the analysis was as follows: 277921 (54.6% females) for handgrip strength; 13 474 (50.4% females) for 2.5‐m gait speed; 139 121 (54.9% females) for 4‐m gait speed; 55 235 (52.2% females) for FTSST performance; 49 309 (56.6% females) for skeletal muscle mass via bioelectrical impedance analysis or dual‐energy x‐ray absorptiometry; and 19 465 (55.8% females) for calf circumference. The established reference values were generally more favourable among younger age groups, compared with older age groups, and they also varied across Asian regions. The largest regional analysis was performed for handgrip strength, and indicated higher values in East and West Asia, compared with South and Southeast Asia. There were also differences in the established reference values between sexes, typically indicating greater handgrip strength, higher gait speed, better performance on the FTSST, more skeletal muscle mass and larger calf circumference among males, compared with females.

**Conclusions:**

This study provides the most comprehensive set of reference values for handgrip strength, gait speed, FTSST performance, skeletal muscle mass and calf circumference in the Asian population. The findings demonstrate clear gradients by age and sex, as well as regional variation. The reference values for Asian populations provided in this study offer a valuable resource for clinical, public health and research applications.

## 1 Introduction

Sarcopenia is a ‘progressive and generalized skeletal muscle disorder involving the accelerated loss of muscle mass and function’ [1]. Ageing is one of the main risk factors for sarcopenia, as it is associated with a decline in muscle strength, physical function and skeletal muscle mass [1, 2, 3, 4]. While there are no universally accepted methods for assessing sarcopenia, guidelines have been provided by various working groups, including the European Working Group on Sarcopenia in Older People and the Asian Working Group for Sarcopenia [5, 6].

The Asian Working Group for Sarcopenia published updated consensus statements on the methodology for diagnosing sarcopenia [6, 7]. This working group suggested that the probability of having sarcopenia should be determined by using handgrip strength and appendicular skeletal muscle mass as diagnostic criteria; calf circumference assessments are suggested for case‐finding, with gait speed and Five‐Times‐Sit‐to‐Stand Test (FTSST) performance are used as additional outcome measures [6, 7]. To interpret individual test results, it is crucial to establish reference (normative) values which enable age‐ and sex‐matched comparisons. While reference values for these outcomes are valuable for diagnosing sarcopenia, they can also serve as early indicators of potential decline of muscle health enabling timely identification of individuals at risk before overt sarcopenia develops and provide a foundation for guiding intervention prescriptions (e.g., exercise, nutritional supplements) [8]. At population level, reference values can also be used when monitoring secular trends within and across different demographic groups over time [9].

Reference values for some of these outcomes have been established from national cohorts in Canada, the United States of America and the United Kingdom [10, 11, 12]. While several studies have also provided reference values in different Asian countries [13, 14, 15], some of the studies have limited generalizability. For example, reference values for handgrip strength in India were based on community‐dwelling adults aged 45+ years and living in a rural area [13]. In Malaysia, reference values for handgrip strength were derived from a small sample (*n* = 412) of university staff, students and visitors [14]. In Singapore, reference values for handgrip strength, gait speed, FTSST test and skeletal muscle mass were based on data from individuals living in a single residential town (Yishun) [15]. In addition, results of these studies are not necessarily generalisable to whole Asia and its specific regions, including Central, East, South, Southeast and West Asia. These limitations highlight the need for more generalizable, cross‐national reference values for the Asia population.

While traditional data synthesis methods (i.e., meta‐analyses based on aggregate data) are useful for combining results across different studies to enable more accurate inference, such approaches are more challenging when applied to reference values. In particular, the use of specific reference percentiles (e.g., 25th, 50th, 75th percentile) and age classifications across studies makes pooling methodologically challenging [13, 14, 15, 16]. One approach to circumvent this issue is to calculate reference values by pooling individual participant data from different cohorts. However, such an approach has been sparingly utilized to generate reference values for Asia, highlighting a gap in the literature [17]. Therefore, the aim of this study was to establish reference values for handgrip strength, gait speed, FTSST performance, skeletal muscle mass and calf circumference by pooling individual participant data from national cohorts in Asia. The reference values are intended to support standardized assessment and early identification of sarcopenia risk across diverse Asian populations.

## 2 Methods

### 2.1 Data Sources

Asian national cohorts containing data on handgrip strength, gait speed, FTSST performance, skeletal muscle mass or calf circumference were identified using multiple approaches. A comprehensive search strategy (Supplementary file 1) was applied in PubMed/MEDLINE and Scopus to identify studies associated with cohorts containing data on the outcomes. The goal was not to include individual studies themselves, but to track the underlying cohorts represented in these publications. Once a cohort was identified, the possibility of accessing individual participant data was explored. Cohorts outlined in the *Gateway to Global Aging Data*, a platform providing population survey data on ageing worldwide, were explored [18]. Additionally, all articles published under the ‘Cohort Profile’ category in the *International Journal of Epidemiology* were screened [19]. These articles aim to increase the visibility of large cohorts and encourage collaboration and data sharing. In some cases, data access was obtained via online registration on the cohort's website. For others, a study proposal was submitted to request access. If no online data access platform was available, the cohort's principal investigator was contacted to inquire about data availability. Only anonymized data were obtained from each of the studies. The search was performed in September 2024 and updated in April 2025.

### 2.2 Inclusion Criteria

Our analysis included national cohort studies from Asian countries that collected data on at least one of the following outcomes: handgrip strength, gait speed, FTSST performance, skeletal muscle mass and calf circumference. Eligible studies were required to provide access to individual participant data, including age, sex and at least one of the above‐mentioned variables, as these data were needed to generate age‐ and sex‐specific reference values. Only cohorts involving adults (≥ 20 years of age) were included [20]. Although some datasets contained information on younger populations, these data were excluded from our calculations. All studies included nationally representative samples drawn from the general population of community‐dwelling adults.

### 2.3 Data analysis

Sex‐ and age‐specific reference values were calculated. Age was categorized into 5‐year intervals starting at 20 years (e.g., 20–24, 25–29, 30–34), continuing through 95–99 years. Individuals aged 100 years and older were grouped into a single open‐ended category (100+ years). For each sex and age group, reference values were expressed as the 5th, 10th, 20th, 30th, 40th, 50th, 60th, 70th, 80th, 90th and 95th percentiles.

In the main analysis, data were pooled across all countries to generate pan‐Asian reference values, provided sufficient data were available. Reference values were also calculated separately for Central Asia, East Asia, South Asia, Southeast Asia and West Asia, that is, Asian geographic regions according to the United Nations classification [21]. Regional pooling was conducted only when data from at least two countries from the respective region were available. When insufficient data were available for regional pooling, only country‐level values were presented. Prior to pooling, datasets were cleaned by removing participants with negative, zero or missing values.

Reference values for handgrip strength were calculated for the dominant hand, following the guidelines from the Asian Working Group for Sarcopenia [6]. When the dominant hand was not specified in the dataset, the hand with higher handgrip strength was considered as the dominant hand. The test used to determine gait speed varied between cohorts, with the 2.5‐m test used in four cohorts and the 4‐m test used in five. The 3‐m and 10‐m tests were used in one cohort each. Therefore, data pooling was performed only for the 2.5‐m and 4‐m tests. All gait speed reference values were presented in meters per second (m/s). As part of the standard FTSST protocol, the goal is to complete the test as soon as possible; therefore, higher values indicate poorer performance. Skeletal muscle mass reference values were derived from cohorts evaluating fat‐free mass via bioelectrical impedance analysis (BIA) or lean body mass via dual‐energy x‐ray absorptiometry (DXA). Country‐specific reference values distinguish between lean body mass and fat‐free mass. Reference values were also provided for appendicular height‐adjusted muscle mass (kg/m^2^), even though these data were available only in one cohort. All analyses were performed using IBM SPSS Statistics V.30 (SPSS Inc. an IBM Company, Chicago, IL, USA).

## 3 Results

### 3.1 Characteristics of the Included Cohorts

Individual participant data were obtained from 20 cohorts, representing 12 Asian countries (Supplementary file 2). China and Singapore were represented by three cohorts each; India, Japan, the Philippines and the Republic of Korea by two cohorts each; and Indonesia, Israel, Lebanon, Malaysia, Mongolia and Sri Lanka by a single cohort. Data from the following cohort studies were obtained: China Health and Retirement Longitudinal Study (CHARLS); Chinese Longitudinal Health and Longevity Survey (CLHLS); Indonesian Family Life Survey (IFLS); Korea National Health and Nutrition Examination Survey (KNHANES); Korean Longitudinal Study of Aging (KLoSA); Lebanon Study on Aging and HeAlth (LSAHA); Longitudinal Ageing Study in India (LASI); Longitudinal Study of Ageing and Health in the Philippines (LSAHP); Malaysia Ageing and Retirement Survey (MARS); Mongolia Population‐Based Study (MPBS); National Survey of the Japanese Elderly (NSJE); Nihon University Japanese Longitudinal Study of Aging (NUJLSA); Panel on Health and Ageing of Singaporean Elderly (PHASE); Philippine Study on Aging (PSA); PopulatION HEalth and Eye Disease PRofilE in Elderly Singaporeans Study (PIONEER); Sri Lanka Health and Ageing Study (SLHAS); Study on Global AGEing and Adult Health (SAGE); Survey of Health, Ageing and Retirement in Europe (SHARE); Well‐being of the Singapore Elderly (WiSE). SAGE includes data for both China and India. Handgrip strength was evaluated in 18 cohorts, gait speed in 11, FTSST performance in four, skeletal muscle mass in four and calf circumference in two cohorts (Supplementary file 2). Appendicular muscle mass was evaluated in one cohort (KNHANES). Data collection across the included cohorts took place between 2004 and 2024. For cohorts with multiple data collection waves, data were combined only if the waves sampled distinct participants (KNHANES and WiSE) or if individual participants could be identified to prevent data duplication (CHARLS and SHARE). Data collection protocols are summarized in Supplementary files 3–7.

### 3.2 Handgrip Strength

#### 3.2.1 Pan‐Asian Reference Values

For females (*n* = 151 731), reference values for handgrip strength were calculated for age groups between 20 and 100+ years. The highest handgrip strength was found among all groups between the 25–29‐year‐olds and 35–39‐year‐olds (5th percentile = 14.9 kg; 50th percentile = 25.0 kg; 95th percentile = 36.0 kg; Table 1 and Supplementary file 8). The lowest handgrip strength was found among the 95–99‐year‐olds and 100+‐year‐olds (5th percentile = 4.0 kg; 50th percentile = 9.9 kg; 95th percentile = 17.3 kg).

**TABLE 1:** Reference values for handgrip strength for females (pooled *n* = 151 731) and males (pooled *n* = 126 190) in Asia (CHARLS, IFLS, KLoSA, KNHANES, LASI, LSAHA, LSAHP, MARS, NSJE, NUJLSA, PHASE, PIONEER, PSA, SAGE, SHARE, SLHAS, WiSE).

Age (years)	*n*	Percentile (kg)										
		5th	10th	20th	30th	40th	50th	60th	70th	80th	90th	95th
**Females**												
20–24	3990	14.0	17.0	20.0	22.0	24.0	25.0	26.0	28.0	30.0	33.0	36.0
25–29	4405	13.0	17.0	20.0	22.0	24.0	25.0	27.0	28.0	30.0	34.0	36.0
30–34	4732	14.7	18.0	20.0	22.0	24.0	25.0	27.0	28.0	30.0	33.0	36.0
35–39	7625	14.9	17.5	20.0	22.0	23.5	25.0	26.0	28.0	30.0	32.5	35.0
40–44	13 372	14.0	16.0	19.0	21.0	22.5	24.0	25.0	27.0	29.0	32.0	34.5
45–49	20 152	13.5	15.5	18.5	20.0	22.0	23.5	25.0	26.5	28.5	32.0	34.5
50–54	17 824	12.0	14.5	17.0	19.0	20.5	22.0	23.5	25.0	27.0	30.0	33.0
55–59	17 728	12.0	14.0	16.5	18.5	20.0	21.5	23.0	25.0	27.0	30.0	32.0
60–64	19 024	11.0	13.0	15.5	17.5	19.0	20.0	21.5	23.0	25.0	28.0	30.0
65–69	15 432	10.0	12.0	14.5	16.0	17.5	19.0	20.0	22.0	24.0	26.0	29.0
70–74	11 304	9.0	11.0	13.5	15.0	16.5	18.0	19.5	20.5	22.1	25.0	27.0
75–79	8047	8.0	10.0	12.0	14.0	15.0	16.8	18.0	20.0	21.0	23.5	25.5
80–84	5300	7.0	9.0	11.0	12.5	14.0	15.0	16.0	18.0	19.5	21.3	24.0
85–89	1988	5.1	7.5	10.0	11.0	12.5	14.0	15.0	16.0	18.0	20.0	22.0
90–94	625	5.0	6.5	9.0	10.0	11.0	12.0	13.5	15.0	16.0	18.5	20.5
95–99	145	4.0	5.5	7.5	9.0	9.9	11.0	12.0	13.0	14.5	16.5	17.8
100+	38	4.1	6.4	7.2	8.1	10.0	10.0	10.5	12.3	14.0	15.3	17.3
**Males**												
20–24	2938	27.0	30.0	34.0	37.0	39.0	40.0	42.0	44.0	47.0	51.0	54.0
25–29	3358	27.0	30.0	35.0	38.0	40.0	41.0	43.0	46.0	48.0	52.0	55.0
30–34	3268	26.0	31.0	35.0	38.0	40.0	42.0	44.0	46.0	49.0	53.0	56.0
35–39	3724	28.0	31.0	35.0	38.0	40.0	42.0	44.0	46.0	48.9	52.0	55.0
40–44	4239	24.0	29.0	33.0	36.0	39.0	41.0	43.0	45.0	48.0	51.0	54.0
45–49	17 141	22.5	25.5	29.5	32.0	34.0	36.0	38.5	41.0	43.5	48.0	51.0
50–54	15 843	20.5	23.5	27.5	30.0	32.5	34.5	36.8	39.0	42.0	46.0	50.0
55–59	15 704	19.0	22.5	26.0	29.0	31.5	33.5	36.0	38.5	41.0	45.0	49.0
60–64	17 694	17.5	20.5	24.5	27.0	29.0	31.0	33.5	36.0	39.0	43.0	46.0
65–69	15 860	15.8	19.0	22.5	25.0	27.0	29.0	31.0	34.0	36.0	40.0	43.5
70–74	11 715	14.0	17.0	20.5	23.0	25.0	27.5	29.5	31.8	34.0	38.0	40.5
75–79	7805	13.2	16.0	20.0	22.0	24.0	26.0	28.0	30.0	32.5	36.0	39.0
80–84	4648	11.5	14.0	17.9	20.0	22.0	24.0	25.7	28.0	30.0	33.0	36.0
85–89	1672	10.0	12.0	15.0	17.5	19.5	21.0	22.0	24.5	27.0	30.0	32.5
90–94	448	9.0	10.5	13.5	15.0	16.5	18.5	20.0	21.8	24.0	28.0	30.0
95–99	101	7.0	9.0	13.0	15.0	16.0	18.0	19.0	20.0	22.0	26.5	30.0
100+	32	7.3	11.6	12.5	13.5	14.0	15.8	18.3	19.5	20.0	20.5	21.5

Abbreviations: CHARLS = China Health and Retirement Longitudinal Study, IFLS = Indonesian Family Life Survey, KLoSA = Korean Longitudinal Study of Aging, KNHANES = Korea National Health and Nutrition Examination Survey, LASI = Longitudinal Ageing Study in India, LSAHA = Lebanon Study on Aging and HeAlth, LSAHP = Longitudinal Study of Ageing and Health in the Philippines, MARS = Malaysia Ageing and Retirement Survey, NSJE = National Survey of the Japanese Elderly, NUJLSA = Nihon University Japanese Longitudinal Study of Aging, PHASE = Panel on Health and Ageing of Singaporean Elderly, PIONEER = PopulatION HEalth and Eye Disease PRofilE in Elderly Singaporeans Study, PSA = Philippine Study on Ageing, SAGE = Study on Global AGEing and Adult Health, SHARE = Survey of Health, Ageing and Retirement in Europe, SLHAS = Sri Lanka Health and Ageing Study, WiSE = Well‐being of the Singapore Elderly.

For males (*n* = 126 190), reference values for handgrip strength were calculated for age groups between 20 and 100+ years. The highest handgrip strength was found among the 30–34‐year‐olds and 35–39‐year‐olds (5th percentile = 28.0 kg; 50th percentile = 42.0 kg; 95th percentile = 56.0 kg; Table 1 and Supplementary file 9). The lowest handgrip strength was found among the 95–99‐year‐olds and 100+‐year‐olds (5th percentile = 7.0 kg; 50th percentile = 15.8 kg; 95th percentile = 21.5 kg).

Compared with females, males had higher handgrip strength in all 187 percentile‐by‐age comparisons, with the differences between the respective percentiles ranging from 4.0 to 20.0 kg.

#### 3.2.2 Region‐Specific Reference Values

Compared to the pan‐Asian reference values, female handgrip strength in East Asia was higher in 142, equal in 9 and lower in 14 out of 165 percentile‐by‐age comparisons, with differences between the respective percentiles ranging from −3.0 to 4.5 kg (Supplementary file 10). Compared to the pan‐Asian reference values, male handgrip strength in East Asia was higher in 157 and equal in 8 out of 165 percentile‐by‐age comparisons, with differences between the respective percentiles ranging up to 7.9 kg (Supplementary file 11).

Compared to the pan‐Asian reference values, female handgrip strength in South Asia was higher in 4, equal in 9 and lower in 163 out of 176 percentile‐by‐age comparisons, with differences between the respective percentiles ranging from −4.0 to 1.0 kg (Supplementary file 12). Compared to the pan‐Asian reference values, male handgrip strength in South Asia was equal in 1 and lower in 164 out of 165 percentile‐by‐age comparisons, with the differences between the respective percentiles ranging up to −9.0 kg (Supplementary file 13).

Compared to the pan‐Asian reference values, female handgrip strength in Southeast Asia was higher in 8, equal in 16 and lower in 108 out of 132 percentile‐by‐age comparisons, with differences between the respective percentiles ranging from −5.2 to 2.0 kg (Supplementary file 14). Compared to the pan‐Asian reference values, male handgrip strength in Southeast Asia was higher in 14, equal in 17 and lower in 101 out of 132 percentile‐by‐age comparisons, with differences between the respective percentiles ranging from −7.1 to 4.0 kg (Supplementary file 15).

Compared to the pan‐Asian reference values, female handgrip strength in West Asia was higher in 72 and equal in 5 out of 77 percentile‐by‐age comparisons, with differences between the respective percentiles ranging up to 4.0 kg (Supplementary file 16). Compared to the pan‐Asian reference values, male handgrip strength in West Asia was higher in all 88 percentile‐by‐age comparisons, with differences between the respective percentiles ranging from 1.6 to 10.5 kg (Supplementary file 17).

Country‐specific reference values for handgrip strength in China, India, Indonesia, Israel, Japan, Lebanon, Malaysia, the Philippines, Republic of Korea, Singapore and Sri Lanka are provided in Supplementary files 18–39.

### 3.3 Gait Speed

#### 3.3.1 Pan‐Asian Reference Values (2.5‐m Test)

For females (*n* = 6792), reference values for gait speed (2.5‐m test) were calculated for age groups between 50 and 94 years. The highest gait speed was found among the 50–54‐year‐olds (5th percentile = 0.43 m/s; 50th percentile = 0.81 m/s; 95th percentile = 1.24 m/s; Table 2 and Supplementary file 40). The lowest gait speed was found among the 85–89‐year‐olds and 90–94‐year‐olds (5th percentile = 0.18 m/s; 50th percentile = 0.52 m/s; 95th percentile = 1.02 m/s).

**TABLE 2:** Reference values for gait speed (2.5 m) for females (pooled *n* = 6792) and males (pooled *n* = 6682) in Asia (CHARLS, NSJE, PHASE, SHARE).

Age (years)	*n*	Percentile (m/s)										
		5th	10th	20th	30th	40th	50th	60th	70th	80th	90th	95th
**Females**												
20–24	n/a	n/a	n/a	n/a	n/a	n/a	n/a	n/a	n/a	n/a	n/a	n/a
25–29	n/a	n/a	n/a	n/a	n/a	n/a	n/a	n/a	n/a	n/a	n/a	n/a
30–34	n/a	n/a	n/a	n/a	n/a	n/a	n/a	n/a	n/a	n/a	n/a	n/a
35–39	n/a	n/a	n/a	n/a	n/a	n/a	n/a	n/a	n/a	n/a	n/a	n/a
40–44	n/a	n/a	n/a	n/a	n/a	n/a	n/a	n/a	n/a	n/a	n/a	n/a
45–49	n/a	n/a	n/a	n/a	n/a	n/a	n/a	n/a	n/a	n/a	n/a	n/a
50–54	449	0.43	0.50	0.63	0.69	0.76	0.81	0.88	0.94	1.02	1.13	1.24
55–59	514	0.41	0.47	0.58	0.65	0.71	0.76	0.82	0.88	0.98	1.10	1.20
60–64	1738	0.35	0.45	0.55	0.62	0.68	0.74	0.80	0.87	0.95	1.11	1.24
65–69	1326	0.32	0.39	0.49	0.58	0.64	0.71	0.77	0.85	0.95	1.09	1.21
70–74	948	0.28	0.35	0.45	0.53	0.60	0.66	0.73	0.80	0.89	1.01	1.14
75–79	858	0.25	0.32	0.42	0.50	0.58	0.65	0.74	0.82	0.94	1.07	1.21
80–84	564	0.24	0.31	0.41	0.49	0.57	0.65	0.73	0.80	0.90	1.05	1.16
85–89	305	0.18	0.25	0.32	0.38	0.46	0.54	0.61	0.71	0.81	0.96	1.11
90–94	90	0.22	0.25	0.35	0.40	0.46	0.52	0.57	0.62	0.71	0.86	1.02
95–99	n/a	n/a	n/a	n/a	n/a	n/a	n/a	n/a	n/a	n/a	n/a	n/a
100+	n/a	n/a	n/a	n/a	n/a	n/a	n/a	n/a	n/a	n/a	n/a	n/a
**Males**												
20–24	n/a	n/a	n/a	n/a	n/a	n/a	n/a	n/a	n/a	n/a	n/a	n/a
25–29	n/a	n/a	n/a	n/a	n/a	n/a	n/a	n/a	n/a	n/a	n/a	n/a
30–34	n/a	n/a	n/a	n/a	n/a	n/a	n/a	n/a	n/a	n/a	n/a	n/a
35–39	n/a	n/a	n/a	n/a	n/a	n/a	n/a	n/a	n/a	n/a	n/a	n/a
40–44	n/a	n/a	n/a	n/a	n/a	n/a	n/a	n/a	n/a	n/a	n/a	n/a
45–49	n/a	n/a	n/a	n/a	n/a	n/a	n/a	n/a	n/a	n/a	n/a	n/a
50–54	402	0.43	0.50	0.63	0.70	0.75	0.80	0.88	0.95	1.01	1.13	1.21
55–59	481	0.39	0.50	0.60	0.67	0.72	0.79	0.84	0.91	0.98	1.08	1.17
60–64	1767	0.39	0.50	0.59	0.66	0.73	0.78	0.83	0.91	1.00	1.14	1.26
65–69	1355	0.34	0.43	0.56	0.63	0.70	0.75	0.82	0.89	1.00	1.15	1.25
70–74	1011	0.33	0.40	0.50	0.58	0.64	0.71	0.78	0.84	0.94	1.11	1.21
75–79	868	0.31	0.40	0.50	0.60	0.66	0.73	0.81	0.88	0.99	1.11	1.24
80–84	532	0.27	0.36	0.47	0.55	0.63	0.71	0.79	0.85	0.94	1.05	1.18
85–89	223	0.19	0.27	0.39	0.51	0.60	0.66	0.73	0.81	0.90	1.05	1.26
90–94	43	0.20	0.23	0.32	0.41	0.50	0.58	0.64	0.72	0.78	1.01	1.02
95–99	n/a	n/a	n/a	n/a	n/a	n/a	n/a	n/a	n/a	n/a	n/a	n/a
100+	n/a	n/a	n/a	n/a	n/a	n/a	n/a	n/a	n/a	n/a	n/a	n/a

Abbreviations: CHARLS = China Health and Retirement Longitudinal Study, NSJE = National Survey of the Japanese Elderly, PHASE = Panel on Health and Ageing of Singaporean Elderly, SHARE = Survey of Health, Ageing and Retirement in Europe.

For males (*n* = 6682), reference values for gait speed (2.5‐m test) were calculated for age groups between 50 and 94 years. The highest gait speed was found among all groups between the 50–54‐year‐olds and 65–69‐year‐olds (5th percentile = 0.43 m/s; 50th percentile = 0.80 m/s; 95th percentile = 1.26 m/s; Table 2 and Supplementary file 41). The lowest gait speed was found among the 85–89‐year‐olds and 90–94‐year‐olds (5th percentile = 0.19 m/s; 50th percentile = 0.58 m/s; 95th percentile = 1.02 m/s).

Compared with females, males had higher gait speed in 81, equal in 8 and lower in 10 out of 99 percentile‐by‐age comparisons, with differences between the respective percentiles ranging from −0.03 to 0.15 m/s.

#### 3.3.2 Region‐Specific Reference Values

Region‐specific reference values for gait speed (2.5‐m test) were calculated only for East Asia. Compared to the pan‐Asian reference values, female gait speed in East Asia was higher in 63, equal in 12 and lower in 2 out of 77 percentile‐by‐age comparisons, with the differences between the respective percentiles ranging from −0.05 to 0.08 m/s (Supplementary file 42). Compared to the pan‐Asian reference values, male gait speed in East Asia was higher in 54 and equal in 23 out of 77 percentile‐by‐age comparisons, with the differences between the respective percentiles ranging up to 0.18 m/s (Supplementary file 43).

Country‐specific reference values for gait speed (2.5‐m test) in China, Israel, Japan and Singapore are provided in Supplementary files 44–51.

#### 3.3.3 Pan‐Asian Reference Values (4‐m Test)

For females (*n* = 76 443), reference values for gait speed (4‐m test) were calculated for age groups between 20 and 100+ years. The highest gait speed was found among the 20–24‐year‐olds and 25–29‐year‐olds (5th percentile = 0.65 m/s; 50th percentile = 0.93 m/s; 95th percentile = 1.32 m/s; Table 3 and Supplementary file 52). The lowest gait speed was found among the 95–99‐year‐olds and 100+ year‐olds (5th percentile = 0.21 m/s; 50th percentile = 0.43 m/s; 95th percentile = 0.69 m/s).

**TABLE 3:** Reference values for gait speed (4 m) for females (pooled *n* = 76 443) and males (pooled *n* = 62 678) in Asia (LASI, PIONEER, SAGE, SLHAS).

Age (years)	*n*	Percentile (m/s)										
		5th	10th	20th	30th	40th	50th	60th	70th	80th	90th	95th
**Females**												
20–24	766	0.63	0.70	0.77	0.83	0.88	0.93	0.95	1.03	1.11	1.25	1.32
25–29	951	0.65	0.70	0.76	0.82	0.87	0.91	0.95	0.98	1.04	1.18	1.25
30–34	1513	0.63	0.69	0.76	0.80	0.86	0.89	0.93	0.97	1.02	1.14	1.25
35–39	4027	0.63	0.69	0.76	0.79	0.85	0.89	0.93	0.97	1.02	1.13	1.23
40–44	8443	0.61	0.66	0.74	0.78	0.82	0.88	0.92	0.95	1.00	1.11	1.20
45–49	12 231	0.61	0.65	0.73	0.77	0.80	0.86	0.90	0.94	0.98	1.09	1.18
50–54	11 132	0.57	0.63	0.71	0.75	0.78	0.83	0.89	0.93	0.98	1.09	1.22
55–59	10 479	0.54	0.61	0.67	0.73	0.77	0.80	0.86	0.92	0.97	1.06	1.20
60–64	9814	0.49	0.56	0.63	0.69	0.74	0.77	0.81	0.88	0.93	1.02	1.14
65–69	7426	0.44	0.51	0.59	0.64	0.69	0.74	0.78	0.82	0.90	0.98	1.09
70–74	4583	0.39	0.45	0.54	0.59	0.64	0.68	0.74	0.78	0.85	0.95	1.05
75–79	2685	0.36	0.42	0.49	0.55	0.60	0.64	0.69	0.75	0.80	0.92	1.00
80–84	1509	0.30	0.36	0.44	0.49	0.54	0.59	0.64	0.70	0.77	0.88	0.98
85–89	592	0.26	0.32	0.40	0.45	0.50	0.54	0.58	0.64	0.69	0.87	0.95
90–94	196	0.24	0.35	0.41	0.45	0.48	0.52	0.56	0.60	0.65	0.78	0.86
95–99	74	0.25	0.28	0.31	0.39	0.43	0.48	0.51	0.55	0.56	0.72	0.86
100+	22	0.21	0.26	0.33	0.38	0.40	0.43	0.49	0.52	0.56	0.68	0.69
**Males**												
20–24	337	0.67	0.74	0.80	0.85	0.93	0.98	1.03	1.11	1.18	1.29	1.46
25–29	354	0.69	0.74	0.83	0.89	0.95	1.00	1.03	1.08	1.18	1.29	1.45
30–34	383	0.67	0.73	0.82	0.87	0.91	0.96	1.03	1.08	1.18	1.26	1.38
35–39	659	0.63	0.70	0.77	0.82	0.87	0.92	0.96	1.02	1.08	1.21	1.25
40–44	689	0.63	0.71	0.78	0.84	0.89	0.93	0.97	1.03	1.11	1.21	1.29
45–49	10 734	0.64	0.72	0.78	0.85	0.90	0.93	0.97	1.01	1.09	1.20	1.28
50–54	10 221	0.62	0.70	0.77	0.82	0.88	0.93	0.96	1.00	1.09	1.22	1.29
55–59	9317	0.60	0.66	0.75	0.79	0.85	0.90	0.94	0.98	1.05	1.18	1.29
60–64	9607	0.57	0.64	0.72	0.77	0.81	0.87	0.92	0.96	1.02	1.14	1.25
65–69	8561	0.51	0.58	0.66	0.73	0.77	0.82	0.88	0.93	0.98	1.10	1.21
70–74	5666	0.46	0.54	0.61	0.66	0.72	0.76	0.80	0.87	0.93	1.03	1.14
75–79	3321	0.41	0.48	0.57	0.63	0.68	0.73	0.78	0.83	0.91	1.00	1.10
80–84	1816	0.37	0.43	0.51	0.57	0.63	0.67	0.73	0.78	0.86	0.96	1.10
85–89	714	0.32	0.39	0.47	0.52	0.56	0.62	0.67	0.73	0.80	0.91	0.98
90–94	220	0.29	0.37	0.44	0.48	0.54	0.58	0.64	0.68	0.75	0.89	0.98
95–99	57	0.32	0.34	0.42	0.47	0.47	0.55	0.57	0.65	0.69	0.75	0.92
100+	22	0.31	0.38	0.42	0.42	0.43	0.46	0.51	0.59	0.62	0.62	0.81

Abbreviations: LASI = Longitudinal Ageing Study in India, PIONEER = PopulatION HEalth and Eye Disease PRofilE in Elderly Singaporeans Study, SAGE = Study on Global AGEing and Adult Health, SLHAS = Sri Lanka Health and Ageing Study.

For males (*n* = 62 678), reference values for gait speed (4‐m test) were calculated for age groups between 20 and 100+ years. The highest gait speed was found among the 20–24‐year‐olds and 25–29‐year‐olds (5th percentile = 0.69 m/s; 50th percentile = 1.00 m/s; 95th percentile = 1.46 m/s; Table 3 and Supplementary file 53). The lowest gait speed was found among all groups between the 90–94‐year‐olds and 100+‐year‐olds (5th percentile = 0.29 m/s; 50th percentile = 0.46 m/s; 95th percentile = 0.81 m/s).

Compared with females, males had higher gait speed in 185, equal in 1 and lower in 1 out of the 187 percentile‐by‐age comparisons, with the differences between the respective percentiles ranging from −0.06 to 0.20 m/s.

#### 3.3.4 Region‐Specific Reference Values

Region‐specific reference values for gait speed (4‐m test) were calculated only for South Asia. Compared to the pan‐Asian reference values, female gait speed in South Asia was equal in 48 and lower in 128 out of 176 percentile‐by‐age comparisons, with the differences between the respective percentiles ranging up to 0.12 m/s (Supplementary file 54). Compared to the pan‐Asian reference values, male gait speed in South Asia was equal in 27 and lower in 138 out of 165 percentile‐by‐age comparisons, with the differences between the respective percentiles ranging up to 0.17 m/s (Supplementary file 55).

Country‐specific reference values for gait speed (4‐m test) in China, India, Singapore and Sri Lanka are provided in Supplementary files 56–63. Country‐specific reference values for the 3‐m (Lebanon) and 10‐m gait speed test (Singapore) are provided in Supplementary files 64–67.

### 3.4 Five‐Times‐Sit‐to‐Stand Test

#### 3.4.1 Pan‐Asian Reference Values

For females (*n* = 28 826), reference values for FTSST performance were calculated for age groups between 20 and 94 years. The best performance was found among the 20–24‐year‐olds (5th percentile = 9.0 s; 50th percentile = 6.2 s; 95th percentile = 4.4 s; Table 4 and Supplementary file 68). The worst performance was found among all groups between the 75–79‐year‐olds and 90–94‐year‐olds (5th percentile = 32.4 s; 50th percentile = 13.8 s; 95th percentile = 6.7 s).

**TABLE 4:** Reference values for the Five‐Times‐Sit‐to‐Stand Test for females (pooled *n* = 28 826) and males (pooled *n* = 26 409) in Asia (CHARLS, IFLS, PHASE, SHARE).

Age (years)	*n*	Percentile (s)										
		5th	10th	20th	30th	40th	50th	60th	70th	80th	90th	95th
**Females**												
20–24	1971	9.0	8.1	7.2	6.7	6.4	6.2	5.8	5.5	5.2	4.7	4.4
25–29	2237	9.2	8.3	7.3	6.8	6.5	6.2	5.9	5.6	5.2	4.8	4.5
30–34	1759	9.5	8.6	7.6	7.1	6.7	6.4	6.1	5.8	5.4	5.0	4.6
35–39	1676	10.1	9.1	8.1	7.4	6.9	6.6	6.3	6.0	5.6	5.1	4.7
40–44	2461	12.9	11.2	9.8	8.8	8.2	7.6	7.1	6.6	6.1	5.6	5.0
45–49	4275	14.4	12.8	11.1	10.0	9.1	8.4	7.8	7.2	6.6	5.9	5.4
50–54	3235	15.1	13.5	11.5	10.3	9.5	8.8	8.1	7.5	6.9	6.0	5.4
55–59	3401	16.5	14.5	12.3	11.1	10.1	9.4	8.7	8.0	7.2	6.3	5.7
60–64	2953	17.3	15.0	12.9	11.6	10.6	9.7	9.0	8.2	7.5	6.4	5.8
65–69	2150	18.0	15.8	13.7	12.4	11.2	10.3	9.5	8.7	7.8	6.8	6.1
70–74	1437	20.6	18.0	15.0	13.5	12.2	11.2	10.2	9.2	8.1	6.9	6.0
75–79	763	22.9	19.2	16.4	14.6	13.3	12.2	11.1	10.0	8.7	7.5	6.7
80–84	354	23.3	20.9	18.3	16.0	14.3	13.2	11.8	10.2	9.2	7.5	6.4
85–89	118	24.4	21.2	17.4	15.9	14.8	13.8	12.5	11.3	10.1	8.1	6.4
90–94	36	32.4	24.9	17.4	16.0	15.6	13.5	12.6	11.3	10.1	8.1	6.6
95–99	n/a	n/a	n/a	n/a	n/a	n/a	n/a	n/a	n/a	n/a	n/a	n/a
100+	n/a	n/a	n/a	n/a	n/a	n/a	n/a	n/a	n/a	n/a	n/a	n/a
**Males**												
20–24	1584	7.5	6.9	6.2	5.8	5.5	5.2	5.0	4.8	4.5	4.1	3.9
25–29	2019	7.7	7.1	6.3	6.0	5.7	5.3	5.1	4.8	4.5	4.2	3.9
30–34	1749	8.2	7.4	6.6	6.2	5.9	5.6	5.2	5.0	4.7	4.3	4.1
35–39	1575	8.7	7.6	6.9	6.5	6.1	5.8	5.5	5.2	4.9	4.3	4.1
40–44	1709	11.0	9.8	8.4	7.5	6.9	6.5	6.0	5.6	5.2	4.7	4.3
45–49	3651	13.3	11.7	10.0	8.9	8.2	7.5	7.0	6.4	5.9	5.2	4.8
50–54	2891	14.0	12.3	10.6	9.5	8.8	8.1	7.4	6.8	6.2	5.6	5.0
55–59	3157	14.8	13.0	11.3	10.0	9.2	8.5	7.8	7.1	6.4	5.7	5.2
60–64	2838	15.5	13.6	11.7	10.5	9.7	9.0	8.2	7.5	6.9	6.0	5.4
65–69	2222	16.0	14.2	12.1	10.9	10.0	9.3	8.5	8.0	7.1	6.2	5.6
70–74	1547	18.0	15.5	13.1	11.7	10.8	10.0	9.2	8.5	7.7	6.7	6.0
75–79	882	18.6	16.4	14.4	12.9	11.3	10.4	9.6	8.7	7.8	6.7	6.0
80–84	410	19.9	16.9	14.7	13.0	12.0	10.9	9.7	8.8	7.7	6.4	5.7
85–89	151	22.5	20.6	17.0	14.9	13.3	12.4	11.3	10.1	9.2	7.4	6.4
90–94	24	26.3	21.1	18.4	16.8	15.1	14.4	12.5	11.5	9.8	8.5	6.8
95–99	n/a	n/a	n/a	n/a	n/a	n/a	n/a	n/a	n/a	n/a	n/a	n/a
100+	n/a	n/a	n/a	n/a	n/a	n/a	n/a	n/a	n/a	n/a	n/a	n/a

Abbreviations: CHARLS = China Health and Retirement Longitudinal Study, IFLS = Indonesian Family Life Survey, PHASE = Panel on Health and Ageing of Singaporean Elderly, SHARE = Survey of Health, Ageing and Retirement in Europe.

For males (*n* = 26 409), reference values for FTSST performance were calculated for age groups between 20 and 94 years. The best performance was found among the 20–24‐year‐olds (5th percentile = 7.5 s; 50th percentile = 5.2 s; 95th percentile = 3.9 s; Table 4 and Supplementary file 69). The worst performance was found among the 90–94‐year‐olds (5th percentile = 26.3 s; 50th percentile = 14.4 s; 95th percentile = 6.8 s).

Compared with females, males had better FTSST performance in 157, equal in 2 and worse in 6 out of the 165 percentile‐by‐age comparisons, with the differences between the respective percentiles ranging from −1.0 to 6.1 s.

#### 3.4.2 Region‐Specific Reference Values

Region‐specific reference values for FTSST performance were calculated only for Southeast Asia. Compared to the pan‐Asian reference values, female performance in the FTSST test in Southeast Asia was better in 28, equal in 3 and worse in 35 out of 66 percentile‐by‐age comparisons, with the differences between the respective percentiles ranging from −6.2 to 1.2 s (Supplementary file 70). Compared to the pan‐Asian reference values, male performance in the FTSST test in Southeast Asia was better in 40, equal in 7 and worse in 8 out of 55 percentile‐by‐age comparisons, with the differences between the respective percentiles ranging from −2.5 to 0.5 s (Supplementary file 71).

Country‐specific reference values for FTSST performance in China, Indonesia, Israel and Singapore are available in Supplementary files 72–79.

### 3.5 Skeletal Muscle Mass

#### 3.5.1 Pan‐Asian Reference Values

For females (*n* = 27 894), reference values for skeletal muscle mass were calculated for age groups between 20 and 94 years. The largest skeletal muscle mass was found among all groups between the 30–34‐year‐olds and 50–54‐year‐olds (5th percentile = 24.1 kg; 50th percentile = 38.6 kg; 95th percentile = 48.3 kg; Table 5 and Supplementary file 80). The lowest skeletal muscle mass was found among the 85–89‐year‐olds and 90–94‐year‐olds (5th percentile = 22.2 kg; 50th percentile = 28.9 kg; 95th percentile = 36.3 kg).

**TABLE 5:** Reference values for skeletal muscle mass (fat‐free mass and lean body mass) for females (pooled *n* = 27 894) and males (pooled *n* = 21 415) in Asia (KNHANES, MPBS, PIONEER, SLHAS).

Age (years)	*n*	Percentile (kg)										
		5th	10th	20th	30th	40th	50th	60th	70th	80th	90th	95th
**Females**												
20–24	1276	23.6	26.0	31.2	33.5	35.0	36.4	37.7	39.3	41.5	44.6	47.0
25–29	1502	23.5	25.5	30.7	33.8	35.6	37.0	38.4	40.0	41.9	44.3	47.0
30–34	1709	24.0	27.5	33.2	35.0	36.7	38.0	39.3	41.0	42.8	45.6	48.3
35–39	2461	23.4	25.7	33.0	35.1	36.5	37.9	39.0	40.5	42.4	44.8	47.1
40–44	2591	24.0	27.0	33.8	35.9	37.1	38.6	40.0	41.3	43.0	45.6	47.8
45–49	2467	24.1	27.0	33.9	35.9	37.4	38.6	39.7	41.0	42.9	45.4	47.6
50–54	2790	24.1	28.5	33.8	35.5	36.9	38.0	39.0	40.4	42.0	44.8	46.9
55–59	2502	22.7	25.9	33.0	35.0	36.4	37.6	38.7	40.0	41.2	43.4	45.3
60–64	2882	23.8	27.6	32.5	34.4	36.0	37.0	38.2	39.5	41.0	43.2	45.0
65–69	2697	24.0	27.4	32.0	33.9	35.1	36.4	37.6	38.9	40.4	42.6	44.4
70–74	2217	23.2	25.1	29.9	32.0	33.4	34.7	36.0	37.3	39.0	41.0	43.0
75–79	1543	23.9	27.0	30.0	31.9	33.1	34.4	35.6	37.0	38.1	40.2	42.0
80–84	1107	24.1	26.1	28.7	30.2	31.6	32.7	33.9	35.1	36.6	39.0	40.2
85–89	122	22.2	22.8	25.1	26.3	27.6	29.5	30.4	31.9	33.8	36.6	38.6
90–94	28	23.3	23.9	25.1	27.2	28.1	28.9	29.7	30.6	31.4	34.8	36.3
95–99	n/a	n/a	n/a	n/a	n/a	n/a	n/a	n/a	n/a	n/a	n/a	n/a
100+	n/a	n/a	n/a	n/a	n/a	n/a	n/a	n/a	n/a	n/a	n/a	n/a
**Males**												
20–24	948	38.6	42.2	45.8	49.3	52.0	54.3	56.0	58.7	61.2	66.0	70.0
25–29	1267	36.9	41.7	47.6	50.9	52.8	55.0	56.9	59.4	62.0	65.8	69.0
30–34	1310	36.5	40.4	47.5	51.0	53.4	55.6	57.5	59.5	61.9	65.0	69.6
35–39	1961	34.3	37.3	44.1	49.0	52.3	54.4	56.1	58.3	61.0	65.0	68.3
40–44	1978	34.4	37.4	46.0	50.3	52.6	54.7	57.0	59.0	61.6	65.4	68.3
45–49	1854	34.9	37.2	44.7	49.0	51.7	53.8	55.5	57.8	60.0	63.0	66.7
50–54	1914	35.1	37.7	45.5	48.8	51.0	53.0	54.6	56.5	58.6	62.1	65.2
55–59	1891	33.8	36.6	43.9	47.5	49.8	51.5	53.4	55.1	57.5	60.5	62.1
60–64	2266	33.9	36.9	43.0	46.0	48.2	50.0	51.6	53.7	56.0	58.8	61.3
65–69	2183	33.5	37.2	42.1	45.3	47.2	49.0	50.5	52.2	54.4	57.2	60.0
70–74	1716	33.6	36.3	40.2	43.0	45.2	47.0	48.7	50.3	52.3	55.2	57.9
75–79	1208	32.8	35.2	39.3	41.9	43.8	45.7	47.0	49.0	50.6	53.1	55.5
80–84	819	33.3	35.0	38.1	40.2	42.0	43.3	44.9	46.4	48.0	51.0	52.9
85–89	100	31.6	32.1	34.8	37.1	38.0	39.2	40.2	41.9	42.9	45.1	48.4
90–94	n/a	n/a	n/a	n/a	n/a	n/a	n/a	n/a	n/a	n/a	n/a	n/a
95–99	n/a	n/a	n/a	n/a	n/a	n/a	n/a	n/a	n/a	n/a	n/a	n/a
100+	n/a	n/a	n/a	n/a	n/a	n/a	n/a	n/a	n/a	n/a	n/a	n/a

Abbreviations: KNHANES = Korea National Health and Nutrition Examination Survey, MPBS = Mongolia Population‐Based Study, PIONEER = PopulatION HEalth and Eye Disease PRofilE in Elderly Singaporeans Study, SLHAS = Sri Lanka Health and Ageing Study.

For males (*n* = 21 415), reference values for skeletal muscle mass were calculated for age groups between 20 and 89 years. The largest skeletal muscle mass was found among all groups between the 20–24‐year‐olds and 30–34‐year‐olds (5th percentile = 38.6 kg; 50th percentile = 55.6 kg; 95th percentile = 70.0 kg; Table 5 and Supplementary file 81). The lowest skeletal muscle mass was found among the 85–89‐year‐olds (5th percentile = 31.6 kg; 50th percentile = 39.2 kg; 95th percentile = 48.4 kg).

Compared with females, males had more skeletal muscle mass in all 154 percentile‐by‐age comparisons, with the differences between the respective percentiles ranging from 8.2 to 23.0 kg.

#### 3.5.2 Region‐Specific Reference Values

Region‐specific reference values for skeletal muscle mass were calculated only for East Asia. Compared to the pan‐Asian reference values, female skeletal muscle mass in East Asia was higher in 133, equal in 7 and lower in 3 out of 143 percentile‐by‐age comparisons, with the differences between the respective percentiles ranging from −0.4 to 9.0 kg (Supplementary file 82). Compared to the pan‐Asian reference values, male muscle mass in East Asia was higher in 137, equal in 5 and lower in 1 out of the 143 percentile‐by‐age comparisons, with the differences between the respective percentiles ranging from −0.2 to 11.2 kg (Supplementary file 83).

Country‐specific reference values for skeletal muscle mass in Mongolia, Republic of Korea, Singapore and Sri Lanka are available in Supplementary files 84–93. Reference values for appendicular height‐adjusted muscle mass in the Republic of Korea are available in Supplementary files 94–97.

### 3.6 Calf Circumference

#### 3.6.1 Pan‐Asian Reference Values

For females (*n* = 10 859), reference values for calf circumference were calculated for age groups between 60 and 100+ years. The largest calf circumference was found among the 65–69‐year‐olds and 70–74‐year‐olds (5th percentile = 23.4 cm; 50th percentile = 31.0 cm; 95th percentile = 40.0 cm; Table 6 and Supplementary file 98). The lowest calf circumference was found among the 100+‐year‐olds (5th percentile = 19.0 cm; 50th percentile = 26.0 cm; 95th percentile = 34.0 cm).

**TABLE 6:** Reference values for calf circumference for females (pooled *n* = 10 859) and males (pooled *n* = 8606) in Asia (CLHLS, LASI DAD).

Age (years)	*n*	Percentile (cm)										
		5th	10th	20th	30th	40th	50th	60th	70th	80th	90th	95th
**Females**												
20–24	n/a	n/a	n/a	n/a	n/a	n/a	n/a	n/a	n/a	n/a	n/a	n/a
25–29	n/a	n/a	n/a	n/a	n/a	n/a	n/a	n/a	n/a	n/a	n/a	n/a
30–34	n/a	n/a	n/a	n/a	n/a	n/a	n/a	n/a	n/a	n/a	n/a	n/a
35–39	n/a	n/a	n/a	n/a	n/a	n/a	n/a	n/a	n/a	n/a	n/a	n/a
40–44	n/a	n/a	n/a	n/a	n/a	n/a	n/a	n/a	n/a	n/a	n/a	n/a
45–49	n/a	n/a	n/a	n/a	n/a	n/a	n/a	n/a	n/a	n/a	n/a	n/a
50–54	n/a	n/a	n/a	n/a	n/a	n/a	n/a	n/a	n/a	n/a	n/a	n/a
55–59	n/a	n/a	n/a	n/a	n/a	n/a	n/a	n/a	n/a	n/a	n/a	n/a
60–64	706	23.4	24.9	26.1	27.5	28.8	30.0	31.0	32.3	33.6	35.4	38.0
65–69	1399	23.4	25.0	27.0	28.5	30.0	31.0	33.0	34.0	35.0	38.0	40.0
70–74	1183	23.0	25.0	27.0	29.0	30.0	31.0	32.1	34.0	35.0	38.0	40.0
75–79	1279	22.2	24.8	27.0	28.1	30.0	31.0	32.0	34.0	35.0	37.0	40.0
80–84	1237	22.2	24.6	27.0	28.0	29.0	30.0	31.0	32.7	34.0	36.0	40.0
85–89	1011	21.2	24.0	26.0	27.0	28.0	29.0	30.0	31.0	33.0	36.0	38.0
90–94	1171	21.0	23.0	25.0	26.0	27.0	29.0	30.0	31.0	32.0	35.0	38.0
95–99	785	19.0	21.0	23.0	25.0	26.0	27.0	28.0	30.0	30.0	33.0	36.0
100+	2088	19.0	20.0	23.0	24.0	25.0	26.0	27.0	28.0	30.0	32.0	34.0
**Males**												
20–24	n/a	n/a	n/a	n/a	n/a	n/a	n/a	n/a	n/a	n/a	n/a	n/a
25–29	n/a	n/a	n/a	n/a	n/a	n/a	n/a	n/a	n/a	n/a	n/a	n/a
30–34	n/a	n/a	n/a	n/a	n/a	n/a	n/a	n/a	n/a	n/a	n/a	n/a
35–39	n/a	n/a	n/a	n/a	n/a	n/a	n/a	n/a	n/a	n/a	n/a	n/a
40–44	n/a	n/a	n/a	n/a	n/a	n/a	n/a	n/a	n/a	n/a	n/a	n/a
45–49	n/a	n/a	n/a	n/a	n/a	n/a	n/a	n/a	n/a	n/a	n/a	n/a
50–54	n/a	n/a	n/a	n/a	n/a	n/a	n/a	n/a	n/a	n/a	n/a	n/a
55–59	n/a	n/a	n/a	n/a	n/a	n/a	n/a	n/a	n/a	n/a	n/a	n/a
60–64	485	25.2	26.4	28.0	29.0	30.1	31.0	32.0	33.0	34.5	36.1	38.0
65–69	1330	25.6	27.0	29.0	30.0	31.4	33.0	34.0	35.0	36.8	39.0	40.0
70–74	1296	25.0	27.0	29.0	30.0	31.7	33.0	34.0	35.0	37.0	39.0	41.0
75–79	1211	24.0	26.3	29.0	30.0	32.0	33.0	34.0	35.0	36.0	39.0	41.0
80–84	1179	23.0	26.0	28.0	30.0	31.0	32.0	33.0	34.8	36.0	38.0	40.0
85–89	879	24.0	26.0	28.0	30.0	30.0	32.0	33.0	34.0	36.0	39.0	40.0
90–94	989	23.0	25.0	27.3	29.0	30.0	31.0	32.0	33.0	35.0	37.0	40.0
95–99	563	23.0	25.0	27.0	28.0	30.0	30.0	32.0	33.0	35.0	37.0	40.0
100+	674	22.0	24.0	26.0	28.0	29.0	30.0	31.0	32.0	34.0	37.0	40.0

Abbreviations: CLHLS = Chinese Longitudinal Health and Longevity Survey; LASI DAD = Longitudinal Aging Study in India‐Diagnostic Assessment of Dementia.

For males (*n* = 8606), reference values for calf circumference were calculated for age groups between 60 and 100+ years. The largest calf circumference was found among all groups between the 65–69‐year‐olds and 75–79‐year‐olds (5th percentile = 25.6 cm; 50th percentile = 33.0 cm; 95th percentile = 41.0 cm; Table 6 and Supplementary file 99). The lowest calf circumference was found among the 100+‐year‐olds (5th percentile = 22.0 cm; 50th percentile = 30.0 cm; 95th percentile = 40.0 cm).

Compared with females, males had larger calf circumference in 96 and equal in 3 out of the 99 percentile‐by‐age comparisons, with the differences between the respective percentiles ranging up to 6 cm.

Country‐specific reference values for calf circumference in China and India are available in Supplementary files 100–103.

## 4 Discussion

This study provided reference values for handgrip strength, gait speed, FTSST performance, skeletal muscle mass and calf circumference in the Asian population. The established reference values are generally more favourable among younger age groups, compared with older age groups, and they also vary across Asian regions. There are differences in the established reference values between sexes, typically indicating greater handgrip strength, higher gait speed, better performance on the FTSST, more skeletal muscle mass and larger calf circumference among males, compared with females. The reference values proposed in this study can be used as Asian‐specific benchmarks for clinical, public health and research purposes.

A recent study established international reference values for handgrip strength using data from 69 countries and regions, including 22 in Asia [8]. While this study provided international reference values, no data were reported for Asia specifically. Compared to the international reference values, the Asian‐specific values established in the current study are considerably lower across nearly all percentiles, with the differences reaching up to 7.4 kg for females and 13.6 kg for males. Similar differences are observed when comparing with reference values established for 27 European countries [22]. This comparison suggests that Asian populations tend to have lower normative handgrip strength, underscoring the need for Asian‐specific reference values. Our reference values also differ from those reported in a recent pooled analysis of eight cohorts from Japan, Malaysia and Taiwan [17]. In this study, the lower percentiles are up to 8.5 and 10 kg smaller for females and males, respectively, while the differences between the upper percentiles ranged between −2 and 6 kg. These discrepancies likely reflect differences in the study samples. The previous analysis included only data from East and Southeast Asia and had a much smaller sample size (*n* = 16 730) [17]. Additionally, their oldest age group was defined as 80 years and older.

Region‐specific reference values in this study revealed that both males and females from Eastern and Western Asia exhibit higher handgrip strength compared to those in South and Southeast Asia. Handgrip strength is influenced by several factors, such as height, body weight, body mass index, waist circumference, upper arm circumference, nutritional status and ethnicity [23, 24] (S18). Handgrip reference values for China were consistently among the highest, whereas those for Singapore were among the lowest, despite approximately 70% of Singapore's population being of Chinese descent (Supplementary file 104; S18). This difference persists even when excluding the Malay and Indian populations from Singapore's data, with comparisons drawn solely between ethnically Chinese individuals from Singapore and those from China, even though Singapore data are available only for those 60+ years of age (S18). It is unlikely that a single factor, such as ethnicity, could fully explain the differences in handgrip strength across regions of Asia.

Only one previous pooled analysis of gait speed in Asia has been conducted, including data from Japan, Malaysia and Taiwan [17]. Their reference values showed an age‐related difference in gait speed, with the lowest speeds observed in the oldest age groups. However, they reported that gait speed peaks at ages 50–54 years, which contrasts with our findings, where gait speed was the highest among individuals of both sexes in their 20s. It is important to note that this observation pertains to the 4‐m test, as the youngest age group for the 2.5‐m test in our data was 50–54‐year‐olds. Several factors may explain these differences. The previous analysis pooled data from multiple test distances (4‐m, 5‐m, 6‐m and 10‐m), whereas our study pooled only data from cohorts using the same test distance. Additionally, the earlier study lacked data for participants aged 20–29 years and had very limited data for those aged 30–39 years (only 9 males and 12 females), resulting in a sample with an over‐representation of older age groups [17].

Although the FTSST is easy to administer and relatively inexpensive (requiring only a chair and a stopwatch), it was administered in only four of the included cohorts. In a recent pooled analysis of eight Asian cohorts, the reported reference values were generally lower (i.e., faster completion times) than those observed herein [17]. Across most overlapping age groups and percentiles, differences of 1–2 s were noted, albeit some were even larger (e.g., 5 s at the 95th percentile for males aged 70–74 years). A key distinction between the two analyses lies in the countries represented. The second‐largest cohort in the previous analysis was from Japan (*n* = 7205), whereas the data for the present study came from cohort studies in China, Indonesia, Israel and Singapore. This is important, as a study comparing FTSST performance between native Japanese and Caucasian women in the United States found that the Japanese group generally completed the test 3–4 s faster [25]. The authors attributed this to lifestyle factors, such as frequent squatting, the use of tatami mats and futon bedding, among the Japanese cohort [25]. The higher performance of Japanese participants and their large representation in the earlier analysis may partly explain the observed differences between the two studies [17].

When comparing the reference values presented in this study with those from Western countries, it is evident that the Asian population generally performs better in the FTSST. For example, studies using data from the Canadian Longitudinal Study on Ageing and 14 European countries included in SHARE generally reported higher percentile values (worse performance) than those observed herein [10, 26]. Performance on the FTSST is influenced by various factors, including visual contrast sensitivity and lower limb proprioception [27]. Future research should consider investigating whether and how much factors underpinning FTSST test performance differ between Asian and Western populations.

Data on skeletal muscle mass were available in four cohorts only, which may not be surprising given the inherent challenges associated with its measurement. Many national cohorts rely on field‐based data collection, where practical limitations restrict the use of certain body composition assessment tools. While BIA offers portable options suitable for field settings, DXA generally requires participants to visit a clinical facility, potentially creating logistical hurdles. This likely explains the limited use of DXA in large‐scale cohort studies. In the calculation of reference values, we combined fat‐free mass (measured by BIA) and lean body mass (measured by DXA) under the unified term ‘skeletal muscle mass’, as they represent similar body components [28].

The reference values indicate that skeletal muscle mass remains relatively stable until approximately 50 years of age, after which it begins to decline more markedly. For example, among males in Singapore, the difference in reference values for muscle mass in each decade of life from 60 to 80 years of age ranges from 1 to 7 kg. This pattern aligns with the higher sarcopenia prevalence with age [29]. These findings suggest that early intervention (i.e., before significant muscle mass loss occurs) could help reduce the risk of muscle‐related conditions, such as sarcopenia. While the Asian Working Group for Sarcopenia recommends assessing skeletal muscle mass, their guidelines focus specifically on height‐adjusted appendicular muscle mass, for which threshold values are provided [6, 7]. In this study, reference values for appendicular muscle mass were available only from one cohort (KNHANES), which used DXA (2008–2011) and BIA (2022–2023). Given these limited data, future studies are needed to establish more comprehensive reference values for appendicular muscle mass in Asia. Additionally, there are limitations in combining data from BIA and DXA, given that these methods provide somewhat different measures. Hence, while our pan‐Asian reference values for skeletal muscle mass may serve as a general guide, using the country‐ and device‐specific reference values may enable a more accurate interpretation.

While assessing skeletal muscle mass in large‐scale cohort studies may present logistical challenges, calf circumference offers a relatively simple surrogate measure. Previous studies have shown that calf circumference is strongly correlated with appendicular lean mass [30]. Cutoffs for calf circumference have been established when screening for low muscle mass (< 34 cm in men and < 33 cm in women) [6, 7]. Despite growing recognition of its relevance for muscle health, calf circumference was assessed in only two of the 20 cohorts included in this study. However, these cohorts represent China and India, which together account for over 50% of the Asian population. Notably, the reference values for calf circumference were generally higher in the Chinese cohort than in the Indian cohort. This finding aligns with existing data on sarcopenia prevalence. The pooled estimates indicate a lower prevalence of sarcopenia in China (10.0%; 95% confidence interval = 9.6%, 10.4%) compared to India (14.2%; 95% confidence interval = 13.8%, 14.6%) [31]. One potential contributor to this difference is a higher prevalence of insufficient physical activity in India, compared with China [32]. In both countries, calf circumference data were only available for adults aged 60+ years, underscoring the need for future studies that would include younger age groups across a broader range of Asian countries.

## 5 Practical Applications

In practice, the reference values can be used to support health screening, monitoring and intervention planning. They can help identify people with low values and may be used to guide interventions. At the population level, they allow evaluation of performance trends across demographics and help define cutoffs for low values. Although no universal thresholds exist, one proposed system classifies values as ‘low’ (< 20th percentile), ‘somewhat low’ (20–39th percentile), ‘moderate’ (40–59th percentile), ‘somewhat high’ (60–79th percentile) and ‘high’ (≥ 80th percentile) and may be used when interpreting the reference values [8]. Indeed, the 20th percentile is commonly used as a cutoff for low values [7]. In the 2025 consensus update from the Asian Working Group on Sarcopenia, cutoffs for handgrip strength were 34 kg (male) and 20 kg (female) for the 50–64‐year‐olds and 28 kg (male) and 18 kg (female) for the 65+ year‐olds [7]. If the 20th percentile is applied as a cutoff in our pan‐Asian data, the values would vary from 7.2 to 20 kg for females and from 12.5 to 34 kg for males, depending on the age group. The data generated herein may help inform future revisions of cutoff values, which could be developed for different groups by sex, age, region and country. From a practical standpoint, the pan‐Asian reference values offer a broad framework for comparisons with the whole Asian population, while the region‐ and country‐specific reference values are expected to provide greater accuracy for specific populations. If the region‐ and country‐specific reference values are not available, the pan‐Asian reference values may represent a suitable alternative.

## 6 Strengths and Limitations

The key strength of this study is the comprehensive and systematic search that was conducted to identify the included national cohorts. Additional strengths are the large sample sizes for some of the analysed reference values and coverage of a broad range of age groups. Despite these strengths, there are limitations that also need to be mentioned. One limitation is that there were no national cohorts from Central Asia; therefore, future research is needed in this region. Another limitation is the use of different protocols for evaluating the outcomes. For example, the handgrip strength assessment was performed: using electrical or mechanical dynamometers; in a standing or seated position; and with the elbow flexed or extended. While all these factors can influence handgrip strength assessments [33], the associated variation tends to be relatively small (i.e., within the range of 1 to 4 kg) [33]. Additionally, although only national cohorts were included, not all provided sampling weights, necessitating the use of unweighted percentiles in the analyses. Another limitation is that the data collection periods across different cohorts ranged from 2004 to 2024. This should be considered when using the reference values, as secular trends have been reported for some of the analysed outcomes. For example, handgrip strength in Japan improved on average by 1.4 kg from 1998 to 2017, with other studies reporting a decline over time [34, 35, 36]. As secular changes are generally considered negligible to small [34, 35, 36], this limitation likely had only minor influence on the reference values, since many percentile differences exceed the magnitude of typical secular changes. Finally, the small number of cohorts for some outcomes, such as calf circumference, also needs to be highlighted, reinforcing the need for future research.

## 7 Conclusion

Using data from 20 national cohorts from Asia, this study established reference values for handgrip strength, gait speed, FTSST performance, skeletal muscle mass and calf circumference. For all analysed variables, the age gradient in percentiles was evident, with younger age groups generally having better test results than older age groups. Regional differences in reference values were also found. Notable sex differences were also observed, with males typically having greater handgrip strength, faster gait speed, better performance on the FTSST, more skeletal muscle mass and larger calf circumference compared to females. The sex‐, age‐ and region‐specific reference values for Asian populations provided in this study offer a valuable resource for clinical, public health and research applications.

## Funding

This research was funded by Abbott Nutrition.

## Ethics Statement

Ethical approval was noted in all of the included cohorts:

China Health and Retirement Longitudinal Study (CHARLS): Peking University Biomedical Ethics Review Committee (ref: IRB00001052‐11014 and IRB00001052‐11015).

Chinese Longitudinal Health and Longevity Survey (CLHLS): Biomedical Ethics Committee of Peking University (ref: IRB00001052‐13074).

Indonesian Family Life Survey (IFLS): Institutional Review Board at the RAND Corporation in the United States and Universitas Gadjah Mada (ref: s0064‐06‐CR01).

Korea National Health and Nutrition Examination Survey (KNHANES): Institutional Review Board of the Korean Centres for Disease Control and Prevention.

Korean Longitudinal Study of Ageing (KLoSA): Institutional Review Board of the Korea Centers for Disease Control and Prevention.

Lebanon Study on Ageing and HeAlth (LSAHA): Institutional Review Board at the American University of Beirut.

Longitudinal Ageing Study in India (LASI): Institutional Review Board at the Indian Council of Medical Research (ICMR), Delhi; Institutional Review Board at the International Institute for Population Sciences (IIPS), Mumbai; Institutional Review Board at the Harvard T.H. Chan School of Public Health (HSPH), Boston; Institutional Review Board at the University of Southern California (USC), Los Angeles; Institutional Review Board at the ICMR‐National AIDS Research Institute (NARI), Pune; and Institutional Review Board at the Regional Geriatric Centres (RGCs), MoHFW.

Longitudinal Study of Ageing and Health in the Philippines (LSAHP): University of the Philippines Manila Research Ethics Board Review Panel 2.

Malaysia Ageing and Retirement Survey (MARS): University of Malaya Research Ethics Committee (UMREC) (reference number: UM.TNC2/UMREC_2408) and the Medical Research and Ethics Committee (MREC) (ref: NMRR ID‐23‐01044‐OQD).

Mongolia Population‐Based Study (MPBS): Medical Research Ethical Committee at the Ministry of Health, Mongolia (ref: 2018‐38).

National Survey of the Japanese Elderly (NSJE): Institutional Review Board of the Tokyo Metropolitan Institute of Gerontology and the University of Michigan.

Nihon University Japanese Longitudinal Study of Aging (NUJLSA): Nihon University Institutional Review Board.

Panel on Health and Ageing of Singaporean Elderly (PHASE): National University of Singapore‐ Institutional Review Board (NUS IRB; 10‐441, L11‐020E, B‐14‐235).

Philippine Study on Aging (PSA): Ethical Review Board of the College of Social Sciences and Philosophy in the University of the Philippines and from the Nihon University Institutional Review Board.

PopulatION HEalth and Eye Disease PRofilE in Elderly Singaporeans Study (PIONEER): SingHealth Centralized Institutional Review Board (CIRB, Reference #2016/3089).

Sri Lanka Health and Ageing Study (SLHAS): Sri Lanka Medical Association Ethical Review Committee (ERC/18‐022).

Study on Global AGEing and Adult Health (SAGE; China): Ethics Review Committee, World Health Organization, Geneva, Switzerland and the Ethics Committee, Shanghai Municipal Centre for Disease Control and Prevention, Shanghai, China.

Study on Global AGEing and Adult Health (SAGE; India): Ethics Review Committee of the World Health Organization, Geneva, Switzerland and the Institutional Review Board, International Institute of Population Sciences, Mumbai, India.

Survey of Health, Ageing and Retirement in Europe (SHARE): Ethics Council of the Max Planck Society as well as relevant national ethics committees in participating countries.

Well‐being of the Singapore Elderly (WiSE): National Healthcare Group Domain Specific Review Board and the SingHealth Centralized Institutional Review Board.

## Conflicts of Interest

S.L.T., D.T.T.H. and Y.L.L. are employees of Abbott Nutrition but did not have access to individual data and were not involved in the data analysis. The other authors declare no conflicts of interest.

## Patient and public involvement

Patients and/or the public were not involved in the design, conduct, reporting or dissemination plans of this research.

## Supporting information


**Supplementary file 1.** Search syntax used in PubMed/MEDLINE and Scopus.
**Supplementary file 2.** Characteristics of the 20 included cohorts across 12 countries.
**Supplementary file 3**. Summary of the protocols used to evaluate handgrip strength.
**Supplementary file 4.** Summary of the protocols used to evaluate gait speed.
**Supplementary file 5.** Summary of the protocols used to evaluate Five‐Times‐Sit‐to‐Stand Test (FTSST) performance.
**Supplementary file 6.** Summary of the devices used to evaluate skeletal muscle mass (fat‐free mass and lean body mass).
**Supplementary file 7.** Summary of the protocols used to evaluate calf circumference.
**Supplementary file 8.** Percentile curves for handgrip strength for females in Asia (CHARLS, IFLS, KLoSA, KNHANES, LASI, LSAHA, LSAHP, MARS, NSJE, NUJLSA, PHASE, PIONEER, PSA, SAGE, SHARE, SLHAS, WiSE; pooled *n* = 151,731).
**Supplementary file 9.** Percentile curves for handgrip strength for males in Asia (CHARLS, IFLS, KLoSA, KNHANES, LASI, LSAHA, LSAHP, MARS, NSJE, NUJLSA, PHASE, PIONEER, PSA, SAGE, SHARE, SLHAS, WiSE; pooled *n* = 126,190).
**Supplementary file 10.** Reference values for handgrip strength for females in East Asia (CHARLS, KLoSA, KNHANES, NSJE, NUJLSA, SAGE; pooled *n* = 50,335).
**Supplementary file 11.** Reference values for handgrip strength for males in East Asia (CHARLS, KLoSA, KNHANES, NSJE, NUJLSA, SAGE; pooled *n* = 42,742).
**Supplementary file 12.** Reference values for handgrip strength for females in South Asia (LASI, SAGE, SLHAS; pooled *n* = 70,115).
**Supplementary file 13.** Reference values for handgrip strength for males in South Asia (LASI, SAGE, SLHAS; pooled *n* = 57,507).
**Supplementary file 14.** Reference values for handgrip strength for females in Southeast Asia (IFLS, LSAHP, MARS, PHASE, PIONEER, PSA, WiSE; pooled *n* = 20,124).
**Supplementary file 15.** Reference values for handgrip strength for males in Southeast Asia (IFLS, LSAHP, MARS, PHASE, PIONEER, PSA, WiSE; pooled *n* = 15,935).
**Supplementary file 16.** Reference values for handgrip strength for females in West Asia (LSAHA, SHARE; pooled *n* = 3,337).
**Supplementary file 17.** Reference values for handgrip strength for males in West Asia (LSAH, SHARE; pooled *n* = 2,958).
**Supplementary file 18.** Reference values for handgrip strength for females in China (CHARLS, SAGE; pooled *n* = 20,810).
**Supplementary file 19.** Reference values for handgrip strength for males in China (CHARLS, SAGE; pooled *n* = 18,944).
**Supplementary file 20.** Reference values for handgrip strength for females in India (LASI, SAGE; pooled *n* = 66,953).
**Supplementary file 21.** Reference values for handgrip strength for males in India (LASI, SAGE; pooled *n* = 54,525).
**Supplementary file 22.** Reference values for handgrip strength for females in Indonesia (IFLS; pooled *n* = 13,518).
**Supplementary file 23.** Reference values for handgrip strength for males in Indonesia (IFLS; pooled *n* = 12,132).
**Supplementary file 24.** Reference values for handgrip strength for females in Israel (SHARE; pooled *n* = 2,684).
**Supplementary file 25.** Reference values for handgrip strength for males in Israel (SHARE; pooled *n* = 2,414).
**Supplementary file 26.** Reference values for handgrip strength for females in Japan (NSJE, NUJLSA; pooled *n* = 2,232).
**Supplementary file 27.** Reference values for handgrip strength for males in Japan (NSJE, NUJLSA; pooled *n* = 1,985).
**Supplementary file 28.** Reference values for handgrip strength for females in Lebanon (LSAHA; pooled *n* = 1,241).
**Supplementary file 29.** Reference values for handgrip strength for males in Lebanon (LSAHA; pooled *n* = 758).
**Supplementary file 30.** Reference values for handgrip strength for females in Malaysia (MARS; pooled *n* = 3,004).
**Supplementary file 31.** Reference values for handgrip strength for males in Malaysia (MARS; pooled *n* = 2,402).
**Supplementary file 32.** Reference values for handgrip strength for females in Philippines (LSAHP, PSA; pooled *n* = 5,125).
**Supplementary file 33.** Reference values for handgrip strength for males in Philippines (LSAHP, PSA; pooled *n* = 3,210).
**Supplementary file 34.** Reference values for handgrip strength for females in the Republic of Korea (KLoSA, KNHANES; pooled *n* = 27,293).
**Supplementary file 35.** Reference values for handgrip strength for males in the Republic of Korea (KLoSA, KNHANES; pooled *n* = 21,808).
**Supplementary file 36.** Reference values for handgrip strength for females in Singapore (PIONEER, SIHLS, WiSE; *n* = 6,150).
**Supplementary file 37.** Reference values for handgrip strength for males in Singapore (PIONEER, SIHLS, and WiSE; *n* = 5,101).
**Supplementary file 38.** Reference values for handgrip strength for females in Sri Lanka (SLHAS; pooled *n* = 3,156).
**Supplementary file 39.** Reference values for handgrip strength for males in Sri Lanka (SLHAS; pooled *n* = 3,047).
**Supplementary file 40.** Percentile curves for 2.5 m gait speed for females in Asia (CHARLS, NSJE, PHASE, SHARE; pooled *n* = 6,792).
**Supplementary file 41.** Percentile curves for 2.5 m gait speed for males in Asia (CHARLS, NSJE, PHASE, SHARE; pooled *n* = 6,682).
**Supplementary file 42.** Reference values for 2.5 m gait speed for females in East Asia (CHARLS, NSJE; pooled *n* = 4,989).
**Supplementary file 43.** Reference values for 2.5 m gait speed for males in East Asia (CHARLS, NSJE; pooled *n* = 5,159).
**Supplementary file 44.** Reference values for 2.5 m gait speed for females in China (CHARLS; pooled *n* = 5,526).
**Supplementary file 45.** Reference values for 2.5 m gait speed for males in China (CHARLS; pooled *n* = 5,515).
**Supplementary file 46.** Reference values for 2.5 m gait speed for females in Israel (SHARE; pooled *n* = 176).
**Supplementary file 47.** Reference values for 2.5 m gait speed for males in Israel (SHARE; pooled *n* = 110).
**Supplementary file 48.** Reference values for 2.5 m gait speed for females in Japan (NSJE; pooled *n* = 983).
**Supplementary file 49.** Reference values for 2.5 m gait speed for males in Japan (NSJE; pooled *n* = 927).
**Supplementary file 50.** Reference values for 2.5 m gait speed for females in Singapore (PHASE; pooled *n* = 651).
**Supplementary file 51.** Reference values for 2.5 m gait speed for males in Singapore (PHASE; pooled *n* = 492).
**Supplementary file 52.** Percentile curves for 4 m gait speed for females in Asia (LASI, PIONEER, SAGE, SLHAS; pooled *n* = 76,443).
**Supplementary file 53.** Percentile curves for 4 m gait speed for males in Asia (LASI, PIONEER, SAGE, SLHAS; pooled *n* = 62,678).
**Supplementary file 54.** Reference values for 4 m gait speed for females in South Asia (LASI, SAGE, SLHAS; pooled *n* = 70,377).
**Supplementary file 55.** Reference values for 4 m gait speed for males in South Asia (LASI, SAGE, SLHAS; pooled *n* = 57,375).
**Supplementary file 56.** Reference values for 4 m gait speed for females in China (SAGE; pooled *n* = 4,660).
**Supplementary file 57.** Reference values for 4 m gait speed for males in China (SAGE; pooled *n* = 4,055).
**Supplementary file 58.** Reference values for 4 m gait speed for females in India (LASI, SAGE; pooled *n* = 67,181).
**Supplementary file 59.** Reference values for 4 m gait speed for males in India (LASI, SAGE; pooled *n* = 54,358).
**Supplementary file 60.** Reference values for 4 m gait speed for females in Singapore (PIONEER; pooled *n* = 1,354).
**Supplementary file 61.** Reference values for 4 m gait speed for males in Singapore (PIONEER; pooled *n* = 1,138).
**Supplementary file 62.** Reference values for 4 m gait speed for females in Sri Lanka (SLHAS; pooled *n* = 3,192).
**Supplementary file 63.** Reference values for 4 m gait speed for males in Sri Lanka (SLHAS; pooled *n* = 3,074).
**Supplementary file 64.** Reference values for 3 m gait speed for females in Lebanon (LSAHA; pooled *n* = 1,238).
**Supplementary file 65.** Reference values for 3 m gait speed for males in Lebanon (LSAHA; pooled *n* = 735).
**Supplementary file 66.** Reference values for 10 m gait speed for females in Singapore (WISE; pooled *n* = 2,058).
**Supplementary file 67.** Reference values for 10 m gait speed for males in Singapore (WISE; pooled *n* = 1,796).
**Supplementary file 68.** Percentile curves for the Five‐Times‐Sit‐to‐Stand Test (FTSST) performance for females in Asia (CHARLS, IFLS, PHASE, SHARE; pooled *n* = 28,826).
**Supplementary file 69.** Percentile curves for the Five‐Times‐Sit‐to‐Stand Test (FTSST) performance for males in Asia (CHARLS, IFLS, PHASE, SHARE; pooled *n* = 26,409).
**Supplementary file 70.** Reference values for the Five‐Times‐Sit‐to‐Stand Test (FTSST) performance for females in Southeast Asia (IFLS, PHASE; pooled *n* = 1,661).
**Supplementary file 71.** Reference values for the Five‐Times‐Sit‐to‐Stand Test (FTSST) performance for males in Southeast Asia (IFLS, PHASE; pooled *n* = 1,405).
**Supplementary file 72.** Reference values for the Five‐Times‐Sit‐to‐Stand Test (FTSST) performance for females in China (CHARLS; pooled *n* = 13,582).
**Supplementary file 73.** Reference values for the Five‐Times‐Sit‐to‐Stand Test (FTSST) performance for males in China (CHARLS; pooled *n* = 12,604).
**Supplementary file 74.** Reference values for the Five‐Times‐Sit‐to‐Stand Test (FTSST) performance for females in Indonesia (IFLS; pooled *n* = 13,106).
**Supplementary file 75.** Reference values for the Five‐Times‐Sit‐to‐Stand Test (FTSST) performance for males in Indonesia (IFLS; pooled *n* = 11,983).
**Supplementary file 76.** Reference values for the Five‐Times‐Sit‐to‐Stand Test (FTSST) performance for females in Israel (SHARE; pooled *n* = 1,536).
**Supplementary file 77.** Reference values for the Five‐Times‐Sit‐to‐Stand Test (FTSST) performance for males in Israel (SHARE; pooled *n* = 1,319).
**Supplementary file 78.** Reference values for the Five‐Times‐Sit‐to‐Stand Test (FTSST) performance for females in Singapore (PHASE; pooled *n* = 534).
**Supplementary file 79.** Reference values for the Five‐Times‐Sit‐to‐Stand Test (FTSST) performance for males in Singapore (PHASE; pooled *n* = 451).
**Supplementary file 80.** Percentile curves for skeletal muscle mass (fat‐free mass and lean body mass) for females in Asia (KNHANES, MPBS, PIONEER, SLHAS; pooled *n* = 27,894).
**Supplementary file 81.** Percentile curves for skeletal muscle mass (fat‐free mass and lean body mass) for males in Asia (KNHANES, MPBS, PIONEER, SLHAS; pooled *n* = 21,415).
**Supplementary file 82.** Reference values for skeletal muscle mass for females in East Asia (MPBS, KNHANES; pooled *n* = 18,003).
**Supplementary file 83.** Reference values for skeletal muscle mass for males in East Asia (MPBS, KNHANES; pooled *n* = 13,390).
**Supplementary file 84.** Reference values for fat‐free mass (bioelectrical impedance analysis) for females in Mongolia (MPBS; pooled *n* = 1,913).
**Supplementary file 85.** Reference values for fat‐free mass (bioelectrical impedance analysis) for males in Mongolia (MPBS; pooled *n* = 1,231).
**Supplementary file 86.** Reference values for fat‐free mass (bioelectrical impedance analysis) for females in the Republic of Korea (KNHANES; pooled *n* = 5,378).
**Supplementary file 87.** Reference values for fat‐free mass (bioelectrical impedance analysis) for males in the Republic of Korea (KNHANES; pooled *n* = 4,122).
**Supplementary file 88.** Reference values for lean body mass (dual‐energy x‐ray absorptiometry) for females in the Republic of Korea (KNHANES; pooled *n* = 10,694).
**Supplementary file 89.** Reference values for lean body mass (dual‐energy x‐ray absorptiometry) for males in the Republic of Korea (KNHANES; pooled *n* = 8,012).
**Supplementary file 90.** Reference values for lean body mass (dual‐energy x‐ray absorptiometry) for females in Singapore (PIONEER; pooled *n* = 1,277).
**Supplementary file 91.** Reference values for lean body mass (dual‐energy x‐ray absorptiometry) for males in Singapore (PIONEER; pooled *n* = 1,038).
**Supplementary file 92.** Reference values for fat‐free mass (bioelectrical impedance analysis) for females in Sri Lanka (SLHAS; pooled *n* = 3,211).
**Supplementary file 93.** Reference values for fat‐free mass (bioelectrical impedance analysis) for males in Sri Lanka (SLHAS; pooled *n* = 2,851).
**Supplementary file 94.** Reference values for height‐adjusted appendicular muscle mass (bioelectrical impedance analysis) for females in the Republic of Korea (KNHANES; pooled *n* = 5,319).
**Supplementary file 95.** Reference values for height‐adjusted appendicular muscle mass (bioelectrical impedance analysis) for males in the Republic of Korea (KNHANES; pooled *n* = 4,102).
**Supplementary file 96.** Reference values for height‐adjusted appendicular muscle mass (dual‐energy x‐ray absorptiometry) for females in the Republic of Korea (KNHANES; pooled *n* = 8,659).
**Supplementary file 97.** Reference values for height‐adjusted appendicular muscle mass (dual‐energy x‐ray absorptiometry) for males in the Republic of Korea (KNHANES; pooled *n* = 6,542).
**Supplementary file 98.** Percentile curves for calf circumference for females in Asia (CLHLS, LASI DAD; pooled *n* = 10,859).
**Supplementary file 99.** Percentile curves for calf circumference for males in Asia (CLHLS, LASI DAD; pooled *n* = 8,606).
**Supplementary file 100.** Reference values for calf circumference for females in China (CLHLS; pooled *n* = 8,685).
**Supplementary file 101.** Reference values for calf circumference for males in China (CLHLS; pooled *n* = 6,744).
**Supplementary file 102.** Reference values for calf circumference for females in India (LASI DAD; pooled *n* = 2,154).
**Supplementary file 103.** Reference values for calf circumference for males in India (LASI DAD; pooled *n* = 1,853).
**Supplementary file 104.** Country‐specific 50^th^ percentile for handgrip strength (kg).

## Data Availability

The data analysed in this study can be accessed upon reasonable request and with approval from the authors and the respective data custodians. We are not authorized to share these datasets without explicit consent from the data custodians.
