# Supplementary material for: Asian Reference Values for Handgrip Strength, Gait Speed, Five‐Times‐Sit‐to‐Stand Test, Muscle Mass and Calf Circumference
Source: J Cachexia Sarcopenia Muscle. 2026 Feb 11;17(1):e70216. doi: 10.1002/jcsm.70216 (PMC12894420; doi:10.1002/jcsm.70216)
Supplement: Supplementary file 1 — Search syntax used in PubMed/MEDLINE and Scopus. Supplementary file 2. Characteristics of the 20 included cohorts across 12 countries. Supplementary file 3. Summary of the protocols used to evaluate handgrip strength. Supplementary file 4. Summary of the protocols used to evaluate gait speed. Supplementary file 5. Summary of the protocols used to evaluate Five‐Times‐Sit‐to‐Stand Test (FTSST) performance. Supplementary file 6. Summary of the devices used to evaluate skeletal muscle mass (fat‐free mass and lean body mass). Supplementary file 7. Summary of the protocols used to evaluate calf circumference. Supplementary file 8. Percentile curves for handgrip strength for females in Asia (CHARLS, IFLS, KLoSA, KNHANES, LASI, LSAHA, LSAHP, MARS, NSJE, NUJLSA, PHASE, PIONEER, PSA, SAGE, SHARE, SLHAS, WiSE; pooled n = 151,731). Supplementary file 9. Percentile curves for handgrip strength for males in Asia (CHARLS, IFLS, KLoSA, KNHANES, LASI, LSAHA, LSAHP, MARS, NSJE, NUJLSA, PHASE, PIONEER, PSA, SAGE, SHARE, SLHAS, WiSE; pooled n = 126,190). Supplementary file 10. Reference values for handgrip strength for females in East Asia (CHARLS, KLoSA, KNHANES, NSJE, NUJLSA, SAGE; pooled n = 50,335). Supplementary file 11. Reference values for handgrip strength for males in East Asia (CHARLS, KLoSA, KNHANES, NSJE, NUJLSA, SAGE; pooled n = 42,742). Supplementary file 12. Reference values for handgrip strength for females in South Asia (LASI, SAGE, SLHAS; pooled n = 70,115). Supplementary file 13. Reference values for handgrip strength for males in South Asia (LASI, SAGE, SLHAS; pooled n = 57,507). Supplementary file 14. Reference values for handgrip strength for females in Southeast Asia (IFLS, LSAHP, MARS, PHASE, PIONEER, PSA, WiSE; pooled n = 20,124). Supplementary file 15. Reference values for handgrip strength for males in Southeast Asia (IFLS, LSAHP, MARS, PHASE, PIONEER, PSA, WiSE; pooled n = 15,935). Supplementary file 16. Reference values for handgrip st [file JCSM-17-e70216-s001.docx]

**Electronic Supplementary Material**

**Asian reference values for handgrip strength, gait speed, Five-Times-Sit-to-Stand Test, muscle mass, and calf circumference**

Jozo Grgic^1,2*^, Siew Ling Tey^3^, Dieu Thi Thu Huynh^3^, Yen Ling Low^3^, Zeljko Pedisic^4^, Nina Schaller^5^, Vanessa Kristina Wazny^1^, Weilan Wang^1^, Yasuhiko Saito^6,7^, Ravindra P. Rannan-Eliya^8^, Hala Ghattas^9^, Monique Chaaya^10^, Carlos Mendes de Leon^11^, Preeti Gupta^12,13^, Ecosse L. Lamoureux^12,13,14^, Mythily Subramaniam^15,16^, Edimansyah Abdin^15^, Rahul Malhotra^17,18^, Angelique Chan^17,18^, Bayasgalan Tumenbayar^19^, Oyunbileg Luvsandavaajav^20^, Bolormaa Enkhtuvshin^21^, Norma Mansor^22^, Halimah Awang^22^, Andrea B. Maier^1,23,24*^

^1^NUS Academy for Healthy Longevity, Yong Loo Lin School of Medicine, National University of Singapore, Singapore, Singapore

^2^Department of Sports Science and Physical Education, The Chinese University of Hong Kong, Hong Kong, China

^3^Abbott Nutrition Research and Development, Asia-Pacific Centre, Singapore 138668, Singapore

^4^School of Public Health, Li Ka Shing Faculty of Medicine, The University of Hong Kong, Hong Kong, China

^5^Department for Preventive Sports Medicine and Sports Cardiology, TUM School of Medicine and Health, TUM University Hospital, Technical University of Munich (TUM), Munich, Germany

^6^College of Economics, Nihon University, Chiyoda, Japan

^7^Economic Research Institute for ASEAN and East Asia, Jakarta, Indonesia

^8^Institute for Health Policy, 72 Park Street, Colombo 00200, Sri Lanka

^9^Department of Health Promotion, Education, and Behavior, Arnold School of Public Health, University of South Carolina, Columbia, South Carolina, USA

^10^Department of Epidemiology and Population Health, Faculty of Health Sciences, American University of Beirut, Beirut, Lebanon

^11^Department of Global Health, Georgetown University School of Health, Washington, District of Columbia, USA

^12^Singapore Eye Research Institute, Singapore National Eye Centre, Singapore, Singapore

^13^Duke-NUS Medical School, Singapore, Singapore

^14^Department of Ophthalmology, The University of Melbourne, Melbourne, Victoria, Australia

^15^Research Division, Institute of Mental Health, Singapore, Singapore

^16^Saw Swee Hock School of Public Health, National University Singapore, Singapore, Singapore

^17^Centre for Ageing Research & Education (CARE), Duke-NUS Medical School, Singapore, Singapore

^18^Programme in Health Services and Systems Research (HSSR), Duke-NUS Medical School, Singapore, Singapore

^19^Postgraduate Training Institute, Mongolian National University of Medical Sciences, Ulaanbaatar, Mongolia

^20^Graduate School of Public Health, Inje University, Republic of Korea

^21^Intermed Hospital, Ulaanbaatar, Mongolia

^22^Social Wellbeing Research Centre, Universiti Malaya, Kuala Lumpur, Malaysia.

^23^Healthy Longevity Translational Research Program, Yong Loo Lin School of Medicine, National University of Singapore, Singapore

^24^Department of Human Movement Sciences, @AgeAmsterdam, Faculty of Behavioural and Movement Sciences, Vrije Universiteit Amsterdam, Amsterdam Movement Sciences, Amsterdam, The Netherlands

**Supplementary file 1.** Search syntax used in PubMed/MEDLINE and Scopus

(norms OR "normative reference" OR "normative study" OR "normative data" OR "normative value*" OR "normative range*" OR "normative curve*" OR "reference data" OR "reference value*" OR "reference range*" OR "cross sectional study" OR "cross-sectional cohort" OR "longitudinal study" OR "longitudinal cohort" OR "prospective study" OR "prospective cohort" OR "population study" OR "population cohort" OR "cohort study" OR "study cohort" OR "national study" OR "national cohort" OR "national survey" OR "health study" OR "health survey" OR "aging study" OR "ageing study" OR "study of ageing" OR "study of aging" OR "study on ageing" OR "study on aging" OR "aging cohort" OR "ageing cohort" OR "longevity survey" OR "retirement survey" OR "retirement study" OR "retirement cohort" OR "nationally representative" OR "nationally-representative") AND (handgrip OR "hand-grip" OR "hand grip" OR "grip strength" OR dynamometer OR "isometric strength" OR "muscle strength" OR "muscular strength" OR "body composition" OR "skeletal muscle" OR "muscle mass" OR "lean body mass" OR "lean mass" OR "fat free mass" OR "fat mass" OR "calf circumference" OR calves OR "five-repetition chair stand test" OR "sit-to-stand" OR "sit to stand" OR "5 times chair stand" OR "five times chair stand" OR gait OR "walk speed" OR "Short Physical Performance Battery" OR SPPB) AND (Asia* OR Kazakhstan OR Kyrgyzstan OR Tajikistan OR Turkmenistan OR Uzbekistan OR China OR "Hong Kong" OR Macao OR Taiwan OR Korea OR Japan OR Mongolia OR Brunei OR Cambodia OR Indonesia OR Lao OR Laos OR Malaysia OR Myanmar OR Philippines OR Singapore OR Thailand OR "Timor-Leste" OR "Viet Nam" OR Vietnam OR Afghanistan OR Bangladesh OR Bhutan OR India OR Iran OR Maldives OR Nepal OR Pakistan OR "Sri Lanka" OR Armenia OR Azerbaijan OR Bahrain OR Cyprus OR Georgia OR Iraq OR Israel OR Jordan OR Kuwait OR Lebanon OR Oman OR Qatar OR "Saudi Arabia" OR Palestine OR Gaza OR "West Bank" OR "Syrian Arab Republic" OR Syria OR Turkey OR "United Arab Emirates" OR UAE OR Yemen)

**Supplementary file 2.** Characteristics of the 20 included cohorts across 12 countries

| **Country** | **Cohort** | **Data collection period** | **Analyzed outcomes and sample size** | **Excluded samples with negative, zero, or missing values** |
| --- | --- | --- | --- | --- |
| China | China Health and Retirement Longitudinal Study (CHARLS) [S1] | Harmonized dataset including waves 1-3 (2011, 2013, 2015) | Handgrip strength (*n* = 31,442), 2.5 m gait speed (*n* = 11,041), and FTSST (*n* = 26,186) | Handgrip strength (*n* = 11,157), 2.5 m gait speed (*n* = 31,558), and FTSST (*n* = 16,413) |
|  | Chinese Longitudinal Health and Longevity Survey (CLHLS) [S2] | Wave 8 (2018) | Calf circumference (*n* = 15,429) | Calf circumference (*n* = 445) |
|  | Study on Global AGEing and Adult Health (SAGE) [S3] | Wave 1 (2007-2010) | Handgrip strength (*n* = 8,606) and 4 m gait speed (*n* = 8,715) | Handgrip strength (*n* = 822) and 4 m gait speed (*n* = 713) |
| India | Longitudinal Ageing Study in India (LASI) [S4] | Wave 1 (2017-2018) | Handgrip strength (*n* = 110,990), 4 m gait speed (*n* = 110,963), and calf circumference (LASI DAD; *n* = 4,007) | Handgrip strength (*n* = 12,570), 4 m gait speed (*n* = 12,597), and calf circumference (LASI DAD; *n* = 89) |
|  | Study on Global AGEing and Adult Health (SAGE) [S3] | Wave 1 (2007-2010) | Handgrip strength (*n* = 10,777) and 4 m gait speed (*n* = 10,846) | Handgrip strength (*n* = 1,421) and 4 m gait speed (*n* = 1,325) |
| Indonesia | Indonesian Family Life Survey (IFLS) [S5] | Wave 5 (2014-2015) | Handgrip strength (*n* = 25,650) and FTSST (*n* = 25,089) | Handgrip strength (*n* = 22,356) and FTSST (*n* = 22,917) |
| Israel | Survey of Health, Ageing and Retirement in Europe (SHARE) [S6] | Harmonized dataset including waves 1, 2, 5, 6, 8 and 9 (2004, 2006, 2013, 2015, 2019-2020, 2021-2022) | Handgrip strength (*n* = 5,098), 2.5 m gait speed (*n* = 286), and FTSST (*n* = 2,855) | Handgrip strength (*n* = 1,263), 2.5 m gait speed (*n* = 6,075), and FTSST (*n* = 3,506) |
| Japan | National Survey of the Japanese Elderly (NSJE) [S7] | Wave 8 (2012) | Handgrip strength (*n* = 2,268) and 2.5 m gait speed (*n* = 1,910) | Handgrip strength (*n* = 232) and 2.5 m gait speed (*n* = 590) |
|  | Nihon University Japanese Longitudinal Study of Aging (NUJLSA) [S8] | Wave 5 (2009) | Handgrip strength (*n* = 1,967) | Handgrip strength (*n* = 569) |
| Lebanon | Lebanon Study on Aging and HeAlth (LSAHA) [S9] | Wave 1 (2023-2024) | Handgrip strength (*n* = 1,999) and 3 m gait speed (*n* = 1,973) | Handgrip strength (*n* = 134) and 3 m gait speed (*n* = 160) |
| Malaysia | Malaysia Ageing and Retirement Survey (MARS) [S10] | Wave 1 (2018-2019) | Handgrip strength (*n* = 5,406) | Handgrip strength (*n* = 207) |
| Mongolia | Mongolia Population-Based Study (MPBS) [S11] | Wave 1 (2019) | Fat-free mass (*n* = 3,144) | Fat-free mass (*n* = 95) |
| Philippines | Longitudinal Study of Ageing and Health in the Philippines (LSAHP) [S12] | Wave 1 (2018) | Handgrip strength (*n* = 5,323) | Handgrip strength (*n* = 662) |
|  | Philippine Study on Aging (PSA) [S13] | Wave 1 (2007) | Handgrip strength (*n* = 3,041) | Handgrip strength (*n* = 64) |
| Republic of Korea | Korea National Health and Nutrition Examination Survey (KNHANES) [S14] | Waves 5-8 (2008-2011) for DXA assessments; waves 19-20 (2022-2023) for BIA assessments; waves 11-16 (2014-2019) and 19-20 (2022-2023) for handgrip strength | Handgrip strength (*n* = 44,508), lean body mass (*n* = 18,706), fat-free mass (*n* = 9,500), and appendicular muscle mass (*n* = 9,421 for BIA; *n* = 15,201 for DXA) | Handgrip strength (*n* = 15,995), lean body mass (*n* = 2,597), fat-free mass (*n* = 3,694), and appendicular muscle mass (*n* = 3,773 for BIA; *n* = 6,102 for DXA) |
|  | Korean Longitudinal Study of Aging (KLoSA) [S15] | Wave 8 (2020) | Handgrip strength (*n* = 9,436) | Handgrip strength (*n* = 818) |
| Singapore | Panel on Health and Ageing of Singaporean Elderly (PHASE) [S16] | Wave 1 (2009) for handgrip strength and wave 3 (2015) for gait speed and FTSST | Handgrip strength (*n* = 4,488), 2.5 m gait speed (*n* = 1,143), and FTSST (*n* = 985) | Handgrip strength (*n* = 503), 2.5 m gait speed (*n* = 429), and FTSST (*n* = 587) |
|  | PopulatION HEalth and Eye Disease PRofilE in Elderly Singaporeans Study (PIONEER) [S17] | Wave 1 (2017-2022) | Handgrip strength (*n* = 2,550), 4 m gait speed (*n* = 2,492), and lean body mass (*n* = 2,315) | Handgrip strength (*n* = 93), 4 m gait speed (*n* = 151), and lean body mass (*n* = 328) |
|  | Well-being of the Singapore Elderly (WiSE) [S18] | Wave 1 and 2 (2013 and 2023) | Handgrip strength (*n* = 4,220) and 10 m gait speed (*n* = 3,854) | Handgrip strength (*n* = 348) and 10 m gait speed (*n* = 714) |
| Sri Lanka | Sri Lanka Health and Ageing Study (SLHAS) [S19] | Wave 1 (2018-2019) | Handgrip strength (*n* = 6,203), fat-free mass (*n* = 6,062), and 4 m gait speed (*n* = 6,266) | Handgrip strength (*n* = 469), fat-free mass (*n* = 406), and 4 m gait speed (*n* = 610) |
| BIA = bioelectrical impedance analysis; DXA = dual-energy X-ray absorptiometry; FTSST = Five-Times-Sit-to-Stand Test; LASI DAD = Longitudinal Aging Study in India-Diagnostic Assessment of Dementia is a subset of participants from LASI | | | | |

**Supplementary file 3**. Summary of the protocols used to evaluate handgrip strength

| **Cohort** | **Protocol description** |
| --- | --- |
| China Health and Retirement Longitudinal Study (CHARLS) | Handgrip strength was assessed using a mechanical dynamometer with the participant in a standing (preferred), seated, or lying position. The arm was flexed at 90° (handshake position), and two attempts were made with both hands. |
| Study on Global AGEing and Adult Health (SAGE) – China | Handgrip strength was assessed using a mechanical dynamometer with the participant in a seated position. The arm was flexed at 90° (handshake position), and two attempts were made with both hands. |
| Longitudinal Ageing Study in India (LASI) | Handgrip strength was assessed using a mechanical dynamometer with the participant in a standing (preferred), seated, or lying position. The arm was flexed at 90° (handshake position), and two attempts were made with both hands. |
| Study on Global AGEing and Adult Health (SAGE) – India | Handgrip strength was assessed using a mechanical dynamometer with the participant in a seated position. The arm was flexed at 90° (handshake position), and two attempts were made with both hands. |
| Indonesian Family Life Survey (IFLS) | Handgrip strength was assessed using a mechanical dynamometer with the participant in a standing position. The arm was flexed at 90° (handshake position), and three attempts were made with both hands. |
| Survey of Health, Ageing and Retirement in Europe (SHARE) | Handgrip strength was assessed using a mechanical dynamometer with the participant in a standing (preferred), seated, or lying position. The arm was flexed at 90° (handshake position), and two attempts were made with both hands. |
| National Survey of the Japanese Elderly (NSJE) | Handgrip strength was assessed using a mechanical dynamometer with the participant in a standing position. The arm was extended at the elbow joint, and two attempts were made with both hands. |
| Nihon University Japanese Longitudinal Study of Aging (NUJLSA) | Handgrip strength was assessed using a mechanical dynamometer with the participant in a standing position. The arm was extended at the elbow joint, and two attempts were made with both hands. |
| Lebanon Study on Aging and HeAlth (LSAHA) | Handgrip strength was assessed using a mechanical dynamometer with the participant in a standing or seated position. The arm was flexed at 90° (handshake position), and two attempts were made with both hands. |
| Malaysia Ageing and Retirement Survey (MARS) | Handgrip strength was assessed using an electronic dynamometer with the participant in a standing position. The arm was flexed at 90° (handshake position), and one attempt was made with both hands. |
| Longitudinal Study of Ageing and Health in the Philippines (LSAHP) | Handgrip strength was assessed using a mechanical dynamometer with the participant in a standing position. The arm was extended at the elbow joint, and three attempts were made with both hands. |
| Philippine Study on Aging (PSA) | Not specified |
| Korea National Health and Nutrition Examination Survey (KNHANES) | Handgrip strength was assessed using an electronic dynamometer with the participant in a standing position. The arm was extended at the elbow joint, and three attempts were made with both hands. |
| Korean Longitudinal Study of Aging (KLoSA) | Handgrip strength was assessed using an electronic dynamometer with the participant in a seated position. The arm was flexed at 90° (handshake position), and two attempts were made with both hands. |
| Panel on Health and Ageing of Singaporean Elderly (PHASE) | Handgrip strength was assessed using a mechanical dynamometer with the participant in a standing (preferred), seated, or lying position. The arm was extended at the elbow joint, and two attempts were made with both hands. |
| PopulatION HEalth and Eye Disease PRofilE in Elderly Singaporeans Study (PIONEER) | Handgrip strength was assessed using an electronic dynamometer with the participant in a seated position. The arm was flexed at 90° (handshake position), and three attempts were made with both hands. |
| Well-being of the Singapore Elderly (WiSE) | Handgrip strength was assessed using an electronic dynamometer with the participant in a seated position. The arm was flexed at 90° (handshake position), and two attempts were made with both hands. |
| Sri Lanka Health and Ageing Study (SLHAS) | Handgrip strength was assessed using an electronic dynamometer with the participant in a standing position. The arm was extended at the elbow joint, and two attempts were made with both hands. |

**Supplementary file** **4.** Summary of the protocols used to evaluate gait speed

| **Cohort** | **Protocol description** |
| --- | --- |
| China Health and Retirement Longitudinal Study (CHARLS) | The test included walking a distance of 2.5 m at normal speed. Two attempts were performed on a floor that was predominantly classified as being linoleum/tile/wood or concrete. |
| Study on Global AGEing and Adult Health (SAGE) – China | The test included walking a distance of 4 m at normal speed. One attempt was performed on flat ground. |
| Longitudinal Ageing Study in India (LASI) | The test included walking a distance of 4 m at normal speed. Two attempts were performed on a non-carpeted area. |
| Study on Global AGEing and Adult Health (SAGE) – India | The test included walking a distance of 4 m at normal speed. One attempt was performed on flat ground. |
| Survey of Health, Ageing and Retirement in Europe (SHARE) | The test included walking a distance of 2.5 m at normal speed. Two attempts were performed and the floor was predominantly classified as being linoleum/tile/wood or low-pile carpet. |
| National Survey of the Japanese Elderly (NSJE) | The test included walking a distance of 2.5 m at normal speed. Three attempts were performed on flat ground. |
| Lebanon Study on Aging and HeAlth (LSAHA) | The test included walking a distance of 3 m at normal speed. Two attempts were performed on flat ground. |
| Panel on Health and Ageing of Singaporean Elderly (PHASE) | The test included walking a distance of 2.5 m at normal speed. One attempt was performed on a non-carpeted area. |
| PopulatION HEalth and Eye Disease PRofilE in Elderly Singaporeans Study (PIONEER) | The test included walking a distance of 4 m at normal speed. One attempt was performed on flat ground. |
| Well-being of the Singapore Elderly (WiSE) | The test included walking a distance of 10 m at normal speed. One attempt was performed on flat ground. |
| Sri Lanka Health and Ageing Study (SLHAS) | The test included walking a distance of 4 m at normal speed. One attempt was performed on flat ground. |

**Supplementary file** **5.** Summary of the protocols used to evaluate Five-Times-Sit-to-Stand Test (FTSST) performance

| **Cohort** | **Protocol description** |
| --- | --- |
| China Health and Retirement Longitudinal Study (CHARLS) | Testing was performed on an armless chair (height = 47 cm). The test was completed on the fifth stand. Only one attempt was performed. |
| Indonesian Family Life Survey (IFLS) | No chair details were provided. The final test position was not specified. Only one attempt was performed. |
| Survey of Health, Ageing and Retirement in Europe (SHARE) | No chair details were provided. The test was completed on the fifth stand. Only one attempt was performed. |
| Social Isolation, Health and Lifestyles Survey (SIHLS) | Testing was performed on a chair that had a straight back, no armrests, a hard seat, and no wheels or rocking motion. The test was completed on the fifth stand. Only one attempt was performed. |

**Supplementary file** **6.** Summary of the devices used to evaluate skeletal muscle mass (fat-free mass and lean body mass)

| **Cohort** | **Protocol description** |
| --- | --- |
| Mongolia Population-Based Study (MPBS) | The TANITA® BC-541 (Tanita Corporation, Japan) bioelectrical impedance analysis device was used to evaluate fat-free mass. |
| Korea National Health and Nutrition Examination Survey (KNHANES) | The Hologic Discovery DXA scanner (Discovery QDR 4500 W, Hologic, Inc., Denver, CO, USA) was used to evaluate lean body mass. The INBODY 770 device (InBody Co. Ltd., Seoul, Korea) was used to evaluate fat-free mass. |
| PopulatION HEalth and Eye Disease PRofilE in Elderly Singaporeans Study (PIONEER) | The Hologic Discovery DXA scanner (Discovery QDR 4500 W, Hologic, Inc., Denver, CO, USA) was used to evaluate lean body mass. |
| Sri Lanka Health and Ageing Study (SLHAS) | The OMRON BF511 Body Composition Monitor (Omron Corporation, Japan) bioelectrical impedance analysis device was used to evaluate fat-free mass. |

**Supplementary file 7.** Summary of the protocols used to evaluate calf circumference

| **Cohort** | **Protocol description** |
| --- | --- |
| Chinese Longitudinal Health and Longevity Survey (CLHLS) | Assessment was performed in a seated position. A measuring tape was applied to the widest point of the right calf. |
| Longitudinal Aging Study in India-Diagnostic Assessment of Dementia (LASI DAD) | Assessment was performed in a seated position. A measuring tape was applied to the widest point of the right calf. |

**Supplementary file 8.** Percentile curves for handgrip strength for females in Asia (CHARLS, IFLS, KLoSA, KNHANES, LASI, LSAHA, LSAHP, MARS, NSJE, NUJLSA, PHASE, PIONEER, PSA, SAGE, SHARE, SLHAS, WiSE; pooled *n* = 151,731)


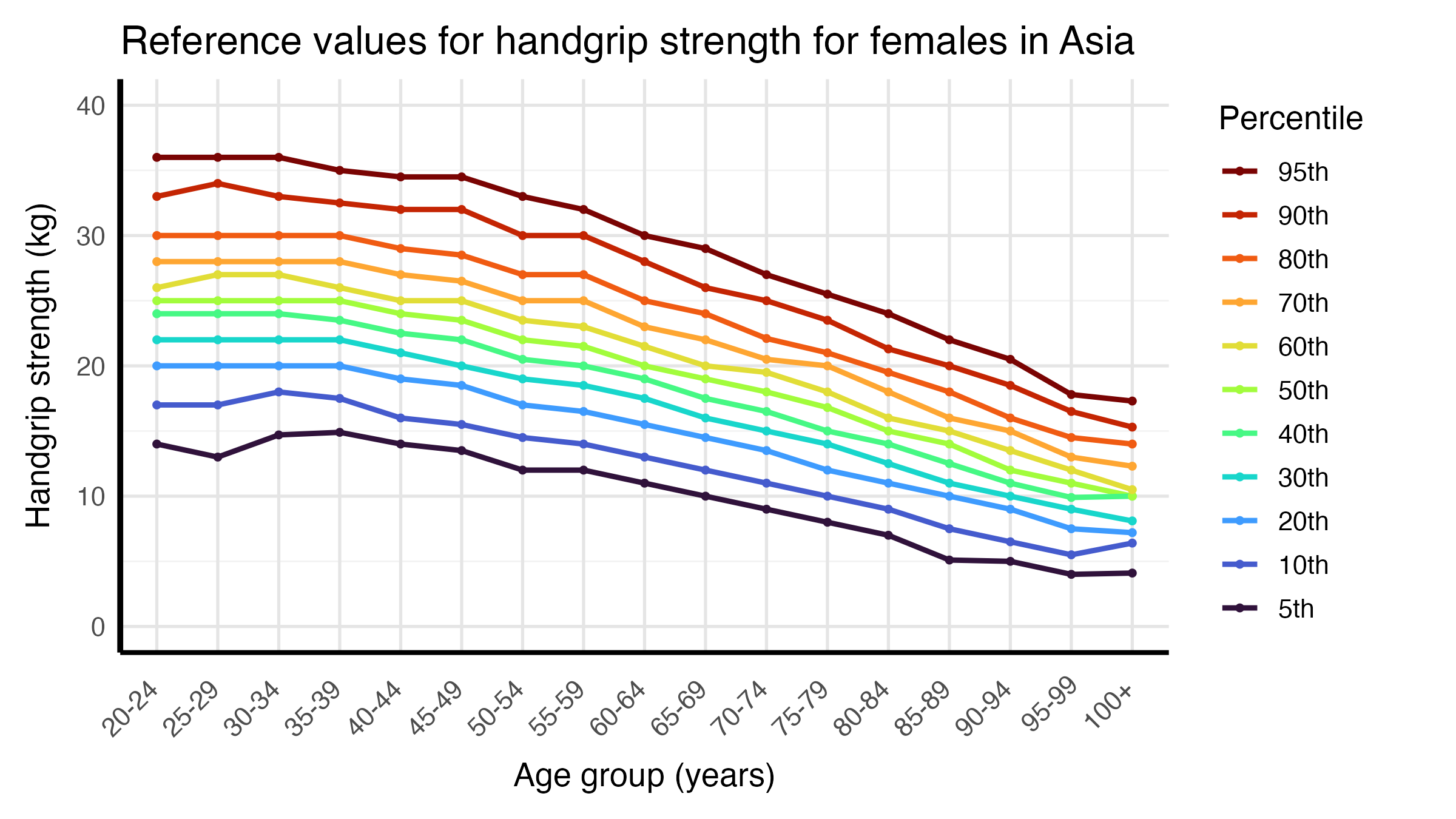


**Supplementary file 9.** Percentile curves for handgrip strength for males in Asia (CHARLS, IFLS, KLoSA, KNHANES, LASI, LSAHA, LSAHP, MARS, NSJE, NUJLSA, PHASE, PIONEER, PSA, SAGE, SHARE, SLHAS, WiSE; pooled *n* = 126,190)


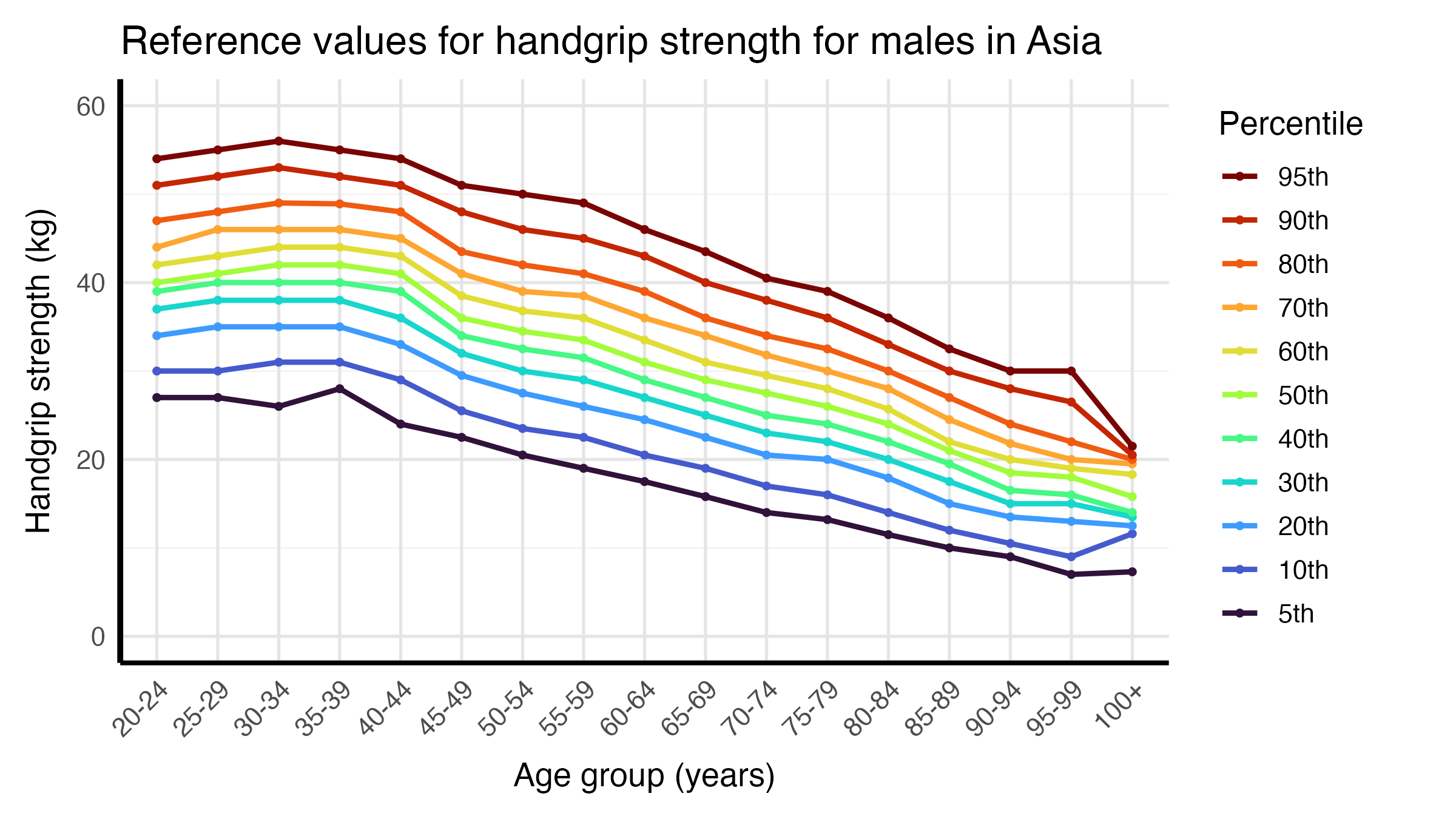


**Supplementary file 10.** Reference values for handgrip strength for females in East Asia (CHARLS, KLoSA, KNHANES, NSJE, NUJLSA, SAGE; pooled *n* = 50,335)

| **Age (years)** | ***n*** | **Percentile (kg)** | | | | | | | | | | |
| --- | --- | --- | --- | --- | --- | --- | --- | --- | --- | --- | --- | --- |
|  |  | **5^th^** | **10^th^** | **20^th^** | **30^th^** | **40^th^** | **50^th^** | **60^th^** | **70^th^** | **80^th^** | **90^th^** | **95^th^** |
| 20-24 | 1,200 | 17.0 | 19.0 | 21.0 | 22.0 | 23.0 | 24.0 | 25.0 | 27.0 | 28.0 | 31.0 | 33.0 |
| 25-29 | 1,193 | 16.5 | 18.0 | 21.0 | 22.0 | 24.0 | 25.0 | 26.0 | 27.0 | 29.0 | 31.0 | 33.0 |
| 30-34 | 1,489 | 18.0 | 20.0 | 22.0 | 23.0 | 24.0 | 26.0 | 27.0 | 28.0 | 30.0 | 32.0 | 34.0 |
| 35-39 | 2,125 | 18.0 | 20.0 | 22.0 | 24.0 | 25.0 | 26.0 | 28.0 | 29.0 | 31.0 | 33.0 | 35.0 |
| 40-44 | 3,353 | 18.0 | 20.0 | 23.0 | 24.0 | 26.0 | 27.0 | 28.3 | 30.0 | 32.0 | 35.0 | 37.3 |
| 45-49 | 6,549 | 18.0 | 20.0 | 22.0 | 24.0 | 25.0 | 27.0 | 28.6 | 30.0 | 32.0 | 35.0 | 38.0 |
| 50-54 | 6,319 | 15.0 | 18.0 | 20.4 | 22.0 | 24.0 | 25.0 | 27.0 | 28.7 | 30.0 | 33.4 | 36.0 |
| 55-59 | 6,578 | 14.1 | 18.0 | 20.0 | 22.0 | 23.0 | 25.0 | 26.0 | 28.0 | 30.0 | 32.0 | 35.0 |
| 60-64 | 6,105 | 14.0 | 16.0 | 19.0 | 21.0 | 22.0 | 24.0 | 25.0 | 26.0 | 28.0 | 30.5 | 33.0 |
| 65-69 | 4,951 | 12.0 | 15.0 | 18.0 | 19.4 | 20.5 | 22.0 | 23.0 | 25.0 | 26.0 | 29.0 | 31.0 |
| 70-74 | 4,127 | 12.0 | 14.0 | 16.6 | 18.0 | 20.0 | 20.6 | 22.0 | 23.0 | 25.0 | 27.0 | 29.4 |
| 75-79 | 3,313 | 10.0 | 12.0 | 15.0 | 16.0 | 18.0 | 19.0 | 20.0 | 21.5 | 23.0 | 25.0 | 27.0 |
| 80-84 | 2,240 | 9.0 | 10.5 | 13.0 | 15.0 | 16.0 | 17.0 | 18.0 | 19.5 | 21.0 | 23.0 | 25.0 |
| 85-89 | 621 | 8.0 | 10.0 | 11.5 | 13.0 | 14.5 | 16.0 | 17.0 | 18.5 | 19.8 | 21.5 | 24.0 |
| 90-94 | 172 | 8.0 | 9.5 | 10.6 | 12.0 | 13.0 | 14.0 | 15.4 | 16.9 | 18.2 | 21.0 | 22.2 |
| 95-99 | n/a | n/a | n/a | n/a | n/a | n/a | n/a | n/a | n/a | n/a | n/a | n/a |
| 100+ | n/a | n/a | n/a | n/a | n/a | n/a | n/a | n/a | n/a | n/a | n/a | n/a |
| CHARLS = China Health and Retirement Longitudinal Study; KLoSA = Korean Longitudinal Study of Aging; KNHANES = Korea National Health and Nutrition Examination Survey; NSJE = National Survey of the Japanese Elderly; NUJLSA = Nihon University Japanese Longitudinal Study of Aging; SAGE = Study on Global AGEing and Adult Health | | | | | | | | | | | | |

**Supplementary file 11.** Reference values for handgrip strength for males in East Asia (CHARLS, KLoSA, KNHANES, NSJE, NUJLSA, SAGE; pooled *n* = 42,742)

| **Age (years)** | ***n*** | **Percentile (kg)** | | | | | | | | | | |
| --- | --- | --- | --- | --- | --- | --- | --- | --- | --- | --- | --- | --- |
|  |  | **5^th^** | **10^th^** | **20^th^** | **30^th^** | **40^th^** | **50^th^** | **60^th^** | **70^th^** | **80^th^** | **90^th^** | **95^th^** |
| 20-24 | 1,046 | 29.0 | 31.5 | 35.0 | 38.0 | 40.0 | 41.0 | 43.0 | 45.0 | 47.0 | 51.0 | 55.0 |
| 25-29 | 1,034 | 30.0 | 33.0 | 36.0 | 38.0 | 40.0 | 42.0 | 44.0 | 46.0 | 49.0 | 52.0 | 55.0 |
| 30-34 | 1,194 | 30.0 | 34.0 | 37.0 | 40.0 | 42.0 | 44.0 | 46.0 | 48.0 | 50.0 | 54.0 | 57.0 |
| 35-39 | 1,535 | 33.0 | 36.0 | 39.0 | 41.0 | 43.0 | 44.0 | 46.0 | 48.0 | 51.0 | 54.0 | 57.0 |
| 40-44 | 2,169 | 31.0 | 34.0 | 38.0 | 40.0 | 42.0 | 44.0 | 45.5 | 47.3 | 50.0 | 53.0 | 56.0 |
| 45-49 | 5,137 | 30.4 | 33.0 | 36.1 | 39.0 | 40.9 | 43.0 | 44.6 | 46.0 | 49.0 | 52.5 | 56.0 |
| 50-54 | 5,178 | 27.0 | 30.0 | 34.0 | 37.0 | 39.0 | 40.0 | 42.0 | 44.5 | 47.0 | 50.2 | 54.0 |
| 55-59 | 5,661 | 25.4 | 29.0 | 32.0 | 35.0 | 37.0 | 39.0 | 41.0 | 43.0 | 45.0 | 49.0 | 52.0 |
| 60-64 | 5,575 | 24.0 | 27.0 | 31.0 | 33.5 | 35.0 | 37.4 | 39.0 | 41.0 | 43.5 | 47.0 | 50.0 |
| 65-69 | 4,888 | 22.0 | 25.0 | 29.0 | 31.0 | 33.0 | 35.0 | 37.0 | 39.0 | 41.0 | 44.0 | 47.0 |
| 70-74 | 3,942 | 20.0 | 23.0 | 26.5 | 29.0 | 30.1 | 32.0 | 34.0 | 36.0 | 38.0 | 41.0 | 43.5 |
| 75-79 | 3,044 | 18.0 | 20.9 | 24.0 | 26.5 | 29.0 | 30.0 | 32.0 | 33.5 | 36.0 | 39.0 | 41.0 |
| 80-84 | 1,821 | 14.0 | 18.0 | 21.0 | 23.5 | 25.0 | 27.0 | 29.0 | 30.0 | 32.0 | 35.0 | 37.2 |
| 85-89 | 430 | 12.0 | 15.5 | 20.0 | 21.0 | 22.8 | 24.5 | 26.0 | 28.0 | 29.5 | 31.0 | 35.0 |
| 90-94 | 88 | 11.0 | 15.0 | 17.0 | 19.2 | 20.0 | 22.0 | 23.1 | 24.5 | 26.3 | 28.4 | 30.0 |
| 95-99 | n/a | n/a | n/a | n/a | n/a | n/a | n/a | n/a | n/a | n/a | n/a | n/a |
| 100+ | n/a | n/a | n/a | n/a | n/a | n/a | n/a | n/a | n/a | n/a | n/a | n/a |
| CHARLS = China Health and Retirement Longitudinal Study; KLoSA = Korean Longitudinal Study of Aging; KNHANES = Korea National Health and Nutrition Examination Survey; NSJE = National Survey of the Japanese Elderly; NUJLSA = Nihon University Japanese Longitudinal Study of Aging; SAGE = Study on Global AGEing and Adult Health | | | | | | | | | | | | |

**Supplementary file 12.** Reference values for handgrip strength for females in South Asia (LASI, SAGE, SLHAS; pooled *n* = 70,115)

| **Age (years)** | ***n*** | **Percentile (kg)** | | | | | | | | | | |
| --- | --- | --- | --- | --- | --- | --- | --- | --- | --- | --- | --- | --- |
|  |  | **5^th^** | **10^th^** | **20^th^** | **30^th^** | **40^th^** | **50^th^** | **60^th^** | **70^th^** | **80^th^** | **90^th^** | **95^th^** |
| 20-24 | 725 | 13.6 | 16.0 | 19.0 | 20.0 | 22.0 | 23.0 | 25.0 | 27.0 | 29.0 | 32.0 | 34.0 |
| 25-29 | 894 | 14.0 | 16.0 | 19.0 | 20.7 | 22.0 | 23.0 | 24.5 | 26.0 | 28.5 | 31.0 | 33.0 |
| 30-34 | 1,465 | 15.0 | 17.5 | 19.5 | 21.0 | 22.0 | 23.5 | 25.0 | 26.5 | 28.5 | 30.5 | 32.5 |
| 35-39 | 3,949 | 14.5 | 16.5 | 19.0 | 20.5 | 22.0 | 23.0 | 24.5 | 26.0 | 27.9 | 30.0 | 32.5 |
| 40-44 | 8,313 | 14.0 | 16.0 | 18.0 | 20.0 | 21.5 | 22.5 | 24.0 | 25.0 | 26.6 | 29.0 | 31.0 |
| 45-49 | 11,995 | 13.0 | 15.0 | 17.0 | 19.0 | 20.5 | 21.5 | 23.0 | 24.5 | 26.0 | 28.0 | 30.5 |
| 50-54 | 10,078 | 12.0 | 14.0 | 16.0 | 18.0 | 19.5 | 20.5 | 21.5 | 23.0 | 25.0 | 27.0 | 29.5 |
| 55-59 | 9,360 | 11.5 | 13.0 | 15.0 | 17.0 | 18.5 | 19.5 | 21.0 | 22.0 | 24.0 | 26.0 | 28.0 |
| 60-64 | 8,800 | 10.5 | 12.0 | 14.5 | 16.0 | 17.0 | 18.5 | 20.0 | 21.0 | 22.5 | 25.0 | 27.0 |
| 65-69 | 6,711 | 9.5 | 11.5 | 13.5 | 15.0 | 16.0 | 17.0 | 18.5 | 20.0 | 21.5 | 23.5 | 25.5 |
| 70-74 | 3,911 | 8.0 | 10.0 | 12.0 | 13.5 | 14.5 | 15.5 | 17.0 | 18.5 | 20.0 | 22.0 | 24.5 |
| 75-79 | 2,153 | 7.5 | 9.0 | 11.0 | 12.0 | 13.5 | 14.5 | 15.5 | 17.0 | 18.5 | 21.0 | 22.5 |
| 80-84 | 1,080 | 6.0 | 7.5 | 10.0 | 11.0 | 12.0 | 13.5 | 14.5 | 15.5 | 17.0 | 19.5 | 20.9 |
| 85-89 | 440 | 5.0 | 7.0 | 9.0 | 10.5 | 12.0 | 13.0 | 14.0 | 15.0 | 16.1 | 19.0 | 21.0 |
| 90-94 | 171 | 5.0 | 6.0 | 8.0 | 9.5 | 10.5 | 11.0 | 12.0 | 13.5 | 14.5 | 17.5 | 19.3 |
| 95-99 | 70 | 5.2 | 5.5 | 7.5 | 8.5 | 9.5 | 11.0 | 12.0 | 13.0 | 14.7 | 16.0 | 16.5 |
| 100+ | n/a | n/a | n/a | n/a | n/a | n/a | n/a | n/a | n/a | n/a | n/a | n/a |
| LASI = Longitudinal Ageing Study in India; SAGE = Study on Global AGEing and Adult Health; SLHAS = Sri Lanka Health and Ageing Study | | | | | | | | | | | | |

**Supplementary file 13.** Reference values for handgrip strength for males in South Asia (LASI, SAGE, SLHAS; pooled *n* = 57,507)

| **Age (years)** | ***n*** | **Percentile (kg)** | | | | | | | | | | |
| --- | --- | --- | --- | --- | --- | --- | --- | --- | --- | --- | --- | --- |
|  |  | **5^th^** | **10^th^** | **20^th^** | **30^th^** | **40^th^** | **50^th^** | **60^th^** | **70^th^** | **80^th^** | **90^th^** | **95^th^** |
| 20-24 | 301 | 25.7 | 27.3 | 30.0 | 31.8 | 33.3 | 36.0 | 37.3 | 39.0 | 41.3 | 46.0 | 50.0 |
| 25-29 | 299 | 25.2 | 27.7 | 30.8 | 32.3 | 34.7 | 36.6 | 38.0 | 40.0 | 42.8 | 46.0 | 49.0 |
| 30-34 | 324 | 21.7 | 26.0 | 30.0 | 32.0 | 34.0 | 36.0 | 38.0 | 40.0 | 41.9 | 44.0 | 47.1 |
| 35-39 | 612 | 24.0 | 26.9 | 30.0 | 32.0 | 33.1 | 35.0 | 37.5 | 40.0 | 41.5 | 45.0 | 47.5 |
| 40-44 | 604 | 23.9 | 25.7 | 29.0 | 31.0 | 32.5 | 34.8 | 36.0 | 38.5 | 41.0 | 44.0 | 47.5 |
| 45-49 | 10,606 | 21.5 | 24.5 | 27.5 | 30.0 | 32.0 | 33.5 | 35.5 | 37.0 | 39.5 | 43.0 | 45.5 |
| 50-54 | 9,443 | 20.0 | 22.5 | 25.5 | 28.0 | 30.0 | 31.5 | 33.5 | 35.5 | 37.5 | 40.9 | 44.0 |
| 55-59 | 8,493 | 18.2 | 21.0 | 24.0 | 26.0 | 28.5 | 30.0 | 32.0 | 33.5 | 36.0 | 39.5 | 42.0 |
| 60-64 | 8,658 | 16.0 | 19.0 | 22.0 | 24.5 | 26.0 | 28.0 | 30.0 | 31.5 | 33.5 | 37.0 | 39.5 |
| 65-69 | 7,885 | 14.5 | 17.5 | 20.5 | 22.5 | 24.5 | 26.0 | 28.0 | 29.7 | 32.0 | 35.0 | 38.0 |
| 70-74 | 5,161 | 13.0 | 15.5 | 18.0 | 20.0 | 22.0 | 24.0 | 25.5 | 27.5 | 30.0 | 33.0 | 35.5 |
| 75-79 | 2,872 | 12.0 | 14.0 | 17.0 | 19.0 | 21.0 | 22.5 | 24.5 | 26.0 | 28.4 | 31.0 | 33.5 |
| 80-84 | 1,470 | 10.5 | 12.5 | 15.0 | 17.0 | 19.0 | 20.5 | 22.5 | 24.5 | 26.1 | 30.0 | 32.1 |
| 85-89 | 578 | 9.5 | 11.0 | 13.5 | 15.0 | 17.5 | 18.8 | 20.2 | 21.5 | 23.5 | 27.0 | 30.0 |
| 90-94 | 201 | 9.0 | 10.0 | 12.5 | 14.0 | 15.5 | 17.0 | 18.5 | 20.0 | 22.0 | 25.5 | 28.5 |
| 95-99 | n/a | n/a | n/a | n/a | n/a | n/a | n/a | n/a | n/a | n/a | n/a | n/a |
| 100+ | n/a | n/a | n/a | n/a | n/a | n/a | n/a | n/a | n/a | n/a | n/a | n/a |
| LASI = Longitudinal Ageing Study in India; SAGE = Study on Global AGEing and Adult Health; SLHAS = Sri Lanka Health and Ageing Study | | | | | | | | | | | | |

**Supplementary file 14.** Reference values for handgrip strength for females in Southeast Asia (IFLS, LSAHP, MARS, PHASE, PIONEER, PSA, WiSE; pooled *n* = 20,124)

| **Age (years)** | ***n*** | **Percentile (kg)** | | | | | | | | | | |
| --- | --- | --- | --- | --- | --- | --- | --- | --- | --- | --- | --- | --- |
|  |  | **5^th^** | **10^th^** | **20^th^** | **30^th^** | **40^th^** | **50^th^** | **60^th^** | **70^th^** | **80^th^** | **90^th^** | **95^th^** |
| 20-24 | n/a | n/a | n/a | n/a | n/a | n/a | n/a | n/a | n/a | n/a | n/a | n/a |
| 25-29 | n/a | n/a | n/a | n/a | n/a | n/a | n/a | n/a | n/a | n/a | n/a | n/a |
| 30-34 | n/a | n/a | n/a | n/a | n/a | n/a | n/a | n/a | n/a | n/a | n/a | n/a |
| 35-39 | n/a | n/a | n/a | n/a | n/a | n/a | n/a | n/a | n/a | n/a | n/a | n/a |
| 40-44 | 1,706 | 10.0 | 12.9 | 17.6 | 20.0 | 22.0 | 24.0 | 26.0 | 28.0 | 30.0 | 34.0 | 36.0 |
| 45-49 | 1,608 | 8.3 | 11.7 | 16.0 | 19.0 | 21.0 | 23.0 | 25.0 | 27.0 | 30.0 | 32.0 | 35.0 |
| 50-54 | 1,427 | 8.0 | 10.6 | 14.0 | 16.5 | 18.9 | 20.0 | 22.0 | 24.0 | 26.0 | 30.0 | 33.0 |
| 55-59 | 1,160 | 8.0 | 11.1 | 14.0 | 16.0 | 18.0 | 20.0 | 21.5 | 23.0 | 25.0 | 28.2 | 32.0 |
| 60-64 | 3,275 | 10.0 | 12.0 | 15.0 | 16.2 | 18.0 | 19.0 | 20.0 | 21.3 | 23.0 | 25.0 | 27.5 |
| 65-69 | 3,076 | 9.0 | 11.0 | 14.0 | 15.0 | 16.8 | 18.0 | 19.0 | 20.0 | 22.0 | 24.0 | 26.0 |
| 70-74 | 2,745 | 9.0 | 11.0 | 13.0 | 14.5 | 16.0 | 17.0 | 18.0 | 19.0 | 20.0 | 22.0 | 24.0 |
| 75-79 | 2,202 | 8.0 | 10.0 | 12.0 | 13.0 | 14.0 | 15.0 | 16.5 | 18.0 | 19.0 | 21.0 | 23.0 |
| 80-84 | 1,750 | 7.0 | 8.9 | 11.0 | 12.0 | 13.0 | 14.0 | 15.0 | 16.0 | 18.0 | 19.9 | 21.0 |
| 85-89 | 858 | 5.0 | 6.0 | 9.0 | 10.0 | 12.0 | 12.8 | 14.0 | 15.0 | 16.2 | 18.5 | 20.0 |
| 90-94 | 264 | 5.0 | 6.0 | 8.3 | 10.0 | 10.1 | 11.8 | 13.0 | 14.0 | 15.3 | 17.0 | 18.9 |
| 95-99 | 53 | 3.0 | 4.0 | 6.0 | 8.0 | 9.0 | 10.0 | 11.0 | 13.0 | 13.8 | 14.9 | 17.5 |
| 100+ | n/a | n/a | n/a | n/a | n/a | n/a | n/a | n/a | n/a | n/a | n/a | n/a |
| IFLS = Indonesian Family Life Survey; LSAHP = Longitudinal Study of Ageing and Health in the Philippines; MARS = Malaysia Ageing and Retirement Survey; PHASE = Panel on Health and Ageing of Singaporean Elderly; PIONEER = PopulatION HEalth and Eye Disease PRofilE in Elderly Singaporeans Study; PSA = Philippine Study on Aging; WiSE = Well-being of the Singapore Elderly | | | | | | | | | | | | |

**Supplementary file 15.** Reference values for handgrip strength for males in Southeast Asia (IFLS, LSAHP, MARS, PHASE, PIONEER, PSA, WiSE; pooled *n* = 15,935)

| **Age (years)** | ***n*** | **Percentile (kg)** | | | | | | | | | | |
| --- | --- | --- | --- | --- | --- | --- | --- | --- | --- | --- | --- | --- |
|  |  | **5^th^** | **10^th^** | **20^th^** | **30^th^** | **40^th^** | **50^th^** | **60^th^** | **70^th^** | **80^th^** | **90^th^** | **95^th^** |
| 20-24 | n/a | n/a | n/a | n/a | n/a | n/a | n/a | n/a | n/a | n/a | n/a | n/a |
| 25-29 | n/a | n/a | n/a | n/a | n/a | n/a | n/a | n/a | n/a | n/a | n/a | n/a |
| 30-34 | n/a | n/a | n/a | n/a | n/a | n/a | n/a | n/a | n/a | n/a | n/a | n/a |
| 35-39 | n/a | n/a | n/a | n/a | n/a | n/a | n/a | n/a | n/a | n/a | n/a | n/a |
| 40-44 | 1,466 | 16.9 | 23.3 | 30.0 | 34.0 | 36.8 | 39.0 | 40.6 | 42.0 | 46.0 | 50.0 | 52.0 |
| 45-49 | 1,398 | 16.0 | 20.8 | 27.8 | 31.0 | 34.0 | 37.0 | 39.0 | 41.0 | 43.0 | 48.0 | 50.0 |
| 50-54 | 1,222 | 14.8 | 19.3 | 25.0 | 29.0 | 32.0 | 34.0 | 37.0 | 39.6 | 42.0 | 45.0 | 48.0 |
| 55-59 | 1,035 | 12.0 | 16.1 | 23.0 | 26.7 | 30.0 | 32.0 | 35.5 | 38.0 | 40.0 | 43.7 | 48.0 |
| 60-64 | 2,842 | 16.0 | 20.0 | 23.5 | 26.0 | 28.0 | 30.0 | 31.0 | 33.0 | 35.8 | 39.0 | 42.0 |
| 65-69 | 2,522 | 15.8 | 19.0 | 22.0 | 25.0 | 27.0 | 28.0 | 30.0 | 31.5 | 34.0 | 37.0 | 40.0 |
| 70-74 | 2,080 | 13.5 | 17.0 | 20.0 | 22.0 | 24.0 | 25.5 | 27.2 | 29.0 | 31.0 | 34.0 | 37.0 |
| 75-79 | 1,522 | 13.0 | 16.0 | 19.0 | 20.5 | 22.0 | 24.0 | 25.0 | 27.0 | 29.0 | 32.0 | 34.9 |
| 80-84 | 1,116 | 10.0 | 14.0 | 17.0 | 19.0 | 20.1 | 22.0 | 23.3 | 25.0 | 27.0 | 30.0 | 32.0 |
| 85-89 | 570 | 10.0 | 12.0 | 15.0 | 17.0 | 18.5 | 20.0 | 21.0 | 22.5 | 25.0 | 28.0 | 31.1 |
| 90-94 | 134 | 7.6 | 9.8 | 13.0 | 15.0 | 16.0 | 17.0 | 19.0 | 20.4 | 23.0 | 26.3 | 31.4 |
| 95-99 | 28 | 11.0 | 11.7 | 14.4 | 16.0 | 16.0 | 18.0 | 19.2 | 20.5 | 22.3 | 27.9 | 31.3 |
| 100+ | n/a | n/a | n/a | n/a | n/a | n/a | n/a | n/a | n/a | n/a | n/a | n/a |
| IFLS = Indonesian Family Life Survey; LSAHP = Longitudinal Study of Ageing and Health in the Philippines; MARS = Malaysia Ageing and Retirement Survey; PHASE = Panel on Health and Ageing of Singaporean Elderly; PIONEER = PopulatION HEalth and Eye Disease PRofilE in Elderly Singaporeans Study; PSA = Philippine Study on Aging; WiSE = Well-being of the Singapore Elderly | | | | | | | | | | | | |

**Supplementary file 16.** Reference values for handgrip strength for females in West Asia (LSAHA, SHARE; pooled *n* = 3,337)

| **Age (years)** | ***n*** | **Percentile (kg)** | | | | | | | | | | |
| --- | --- | --- | --- | --- | --- | --- | --- | --- | --- | --- | --- | --- |
|  |  | **5^th^** | **10^th^** | **20^th^** | **30^th^** | **40^th^** | **50^th^** | **60^th^** | **70^th^** | **80^th^** | **90^th^** | **95^th^** |
| 20-24 | n/a | n/a | n/a | n/a | n/a | n/a | n/a | n/a | n/a | n/a | n/a | n/a |
| 25-29 | n/a | n/a | n/a | n/a | n/a | n/a | n/a | n/a | n/a | n/a | n/a | n/a |
| 30-34 | n/a | n/a | n/a | n/a | n/a | n/a | n/a | n/a | n/a | n/a | n/a | n/a |
| 35-39 | n/a | n/a | n/a | n/a | n/a | n/a | n/a | n/a | n/a | n/a | n/a | n/a |
| 40-44 | n/a | n/a | n/a | n/a | n/a | n/a | n/a | n/a | n/a | n/a | n/a | n/a |
| 45-49 | n/a | n/a | n/a | n/a | n/a | n/a | n/a | n/a | n/a | n/a | n/a | n/a |
| 50-54 | n/a | n/a | n/a | n/a | n/a | n/a | n/a | n/a | n/a | n/a | n/a | n/a |
| 55-59 | 630 | 13.0 | 17.0 | 20.0 | 22.0 | 24.0 | 25.0 | 27.0 | 29.0 | 30.0 | 34.0 | 35.0 |
| 60-64 | 844 | 12.0 | 15.0 | 19.0 | 20.0 | 22.0 | 23.0 | 25.0 | 27.0 | 29.0 | 31.0 | 34.0 |
| 65-69 | 694 | 10.7 | 12.0 | 16.0 | 18.0 | 20.0 | 21.0 | 22.0 | 25.0 | 27.0 | 30.0 | 32.0 |
| 70-74 | 521 | 10.0 | 12.0 | 15.0 | 17.0 | 19.0 | 20.0 | 21.0 | 24.0 | 25.0 | 29.0 | 30.0 |
| 75-79 | 379 | 10.0 | 11.0 | 13.0 | 15.0 | 17.0 | 18.0 | 20.0 | 20.0 | 23.0 | 25.0 | 27.0 |
| 80-84 | 230 | 7.0 | 10.0 | 11.0 | 13.0 | 15.6 | 17.0 | 19.0 | 20.0 | 22.0 | 25.0 | 27.0 |
| 85-89 | 39 | 8.4 | 9.0 | 10.0 | 12.0 | 14.0 | 16.0 | 18.0 | 20.0 | 20.0 | 23.0 | 24.0 |
| 90-94 | n/a | n/a | n/a | n/a | n/a | n/a | n/a | n/a | n/a | n/a | n/a | n/a |
| 95-99 | n/a | n/a | n/a | n/a | n/a | n/a | n/a | n/a | n/a | n/a | n/a | n/a |
| 100+ | n/a | n/a | n/a | n/a | n/a | n/a | n/a | n/a | n/a | n/a | n/a | n/a |
| LSAHA = Lebanon Study on Aging and HeAlth; SHARE = Survey of Health, Ageing and Retirement in Europe | | | | | | | | | | | | |

**Supplementary file 17.** Reference values for handgrip strength for males in West Asia (LSAH, SHARE; pooled *n* = 2,958)

| **Age (years)** | ***n*** | **Percentile (kg)** | | | | | | | | | | |
| --- | --- | --- | --- | --- | --- | --- | --- | --- | --- | --- | --- | --- |
|  |  | **5^th^** | **10^th^** | **20^th^** | **30^th^** | **40^th^** | **50^th^** | **60^th^** | **70^th^** | **80^th^** | **90^th^** | **95^th^** |
| 20-24 | n/a | n/a | n/a | n/a | n/a | n/a | n/a | n/a | n/a | n/a | n/a | n/a |
| 25-29 | n/a | n/a | n/a | n/a | n/a | n/a | n/a | n/a | n/a | n/a | n/a | n/a |
| 30-34 | n/a | n/a | n/a | n/a | n/a | n/a | n/a | n/a | n/a | n/a | n/a | n/a |
| 35-39 | n/a | n/a | n/a | n/a | n/a | n/a | n/a | n/a | n/a | n/a | n/a | n/a |
| 40-44 | n/a | n/a | n/a | n/a | n/a | n/a | n/a | n/a | n/a | n/a | n/a | n/a |
| 45-49 | n/a | n/a | n/a | n/a | n/a | n/a | n/a | n/a | n/a | n/a | n/a | n/a |
| 50-54 | n/a | n/a | n/a | n/a | n/a | n/a | n/a | n/a | n/a | n/a | n/a | n/a |
| 55-59 | 515 | 26.7 | 30.0 | 35.0 | 39.0 | 40.0 | 43.0 | 45.0 | 48.0 | 50.0 | 55.0 | 58.0 |
| 60-64 | 619 | 23.0 | 28.0 | 32.6 | 36.0 | 39.0 | 40.0 | 42.8 | 45.0 | 48.0 | 52.0 | 55.0 |
| 65-69 | 565 | 20.0 | 24.0 | 28.0 | 31.0 | 34.0 | 36.0 | 39.0 | 41.0 | 44.0 | 48.0 | 51.0 |
| 70-74 | 532 | 17.0 | 21.0 | 26.0 | 30.0 | 32.0 | 34.0 | 37.0 | 39.0 | 41.0 | 45.9 | 49.5 |
| 75-79 | 367 | 18.0 | 20.0 | 24.0 | 27.0 | 29.0 | 30.0 | 33.0 | 35.0 | 38.0 | 42.0 | 47.0 |
| 80-84 | 241 | 17.0 | 19.0 | 21.0 | 25.0 | 27.0 | 29.0 | 30.0 | 33.0 | 35.0 | 39.0 | 42.0 |
| 85-89 | 94 | 12.0 | 16.3 | 20.0 | 22.0 | 24.0 | 26.0 | 28.8 | 30.0 | 32.0 | 36.7 | 43.0 |
| 90-94 | 25 | 15.2 | 16.0 | 16.8 | 21.2 | 22.0 | 24.0 | 26.4 | 28.0 | 29.0 | 30.0 | 31.6 |
| 95-99 | n/a | n/a | n/a | n/a | n/a | n/a | n/a | n/a | n/a | n/a | n/a | n/a |
| 100+ | n/a | n/a | n/a | n/a | n/a | n/a | n/a | n/a | n/a | n/a | n/a | n/a |
| LSAH = Lebanon Study on Aging and Health; SHARE = Survey of Health, Ageing and Retirement in Europe | | | | | | | | | | | | |

**Supplementary file 18.** Reference values for handgrip strength for females in China (CHARLS, SAGE; pooled *n* = 20,810)

| **Age (years)** | ***n*** | **Percentile (kg)** | | | | | | | | | | |
| --- | --- | --- | --- | --- | --- | --- | --- | --- | --- | --- | --- | --- |
|  |  | **5^th^** | **10^th^** | **20^th^** | **30^th^** | **40^th^** | **50^th^** | **60^th^** | **70^th^** | **80^th^** | **90^th^** | **95^th^** |
| 20-24 | 50 | 16.5 | 17.9 | 22.0 | 23.0 | 24.0 | 27.0 | 30.0 | 32.0 | 33.6 | 38.0 | 38.0 |
| 25-29 | 54 | 14.0 | 18.0 | 20.0 | 23.9 | 26.0 | 28.5 | 30.0 | 32.0 | 33.0 | 36.9 | 40.7 |
| 30-34 | 73 | 9.2 | 12.0 | 16.0 | 20.6 | 24.0 | 26.0 | 28.0 | 30.0 | 32.0 | 37.6 | 38.0 |
| 35-39 | 247 | 15.7 | 20.0 | 23.1 | 26.0 | 28.0 | 29.9 | 31.0 | 33.0 | 35.2 | 38.1 | 40.0 |
| 40-44 | 1,419 | 18.1 | 21.0 | 24.0 | 26.0 | 28.0 | 29.5 | 30.7 | 32.5 | 35.0 | 37.9 | 40.0 |
| 45-49 | 3,573 | 18.0 | 20.5 | 24.0 | 26.0 | 27.5 | 29.0 | 30.5 | 32.1 | 34.5 | 37.2 | 40.0 |
| 50-54 | 3,483 | 13.0 | 17.8 | 21.0 | 23.0 | 25.0 | 27.0 | 28.8 | 30.0 | 32.0 | 35.1 | 38.0 |
| 55-59 | 3,745 | 12.1 | 16.0 | 20.0 | 22.0 | 24.0 | 26.0 | 27.5 | 29.5 | 31.0 | 34.5 | 38.0 |
| 60-64 | 3,236 | 12.0 | 15.1 | 19.0 | 21.0 | 23.0 | 24.1 | 26.0 | 28.0 | 30.0 | 32.2 | 36.0 |
| 65-69 | 2,024 | 10.0 | 13.5 | 17.0 | 19.0 | 20.5 | 22.1 | 24.0 | 26.0 | 28.0 | 31.0 | 34.0 |
| 70-74 | 1,379 | 10.0 | 12.0 | 16.0 | 18.0 | 20.0 | 21.0 | 22.1 | 24.0 | 26.0 | 30.0 | 32.0 |
| 75-79 | 932 | 6.0 | 10.0 | 12.5 | 15.0 | 18.0 | 19.5 | 20.5 | 22.0 | 24.6 | 28.0 | 30.0 |
| 80-84 | 419 | 7.0 | 10.0 | 12.0 | 13.5 | 15.5 | 17.2 | 19.0 | 20.0 | 22.5 | 27.1 | 34.0 |
| 85-89 | 146 | 5.6 | 8.0 | 10.5 | 12.0 | 13.0 | 15.2 | 17.2 | 20.0 | 22.0 | 28.8 | 31.5 |
| 90-94 | 30 | 6.9 | 8.0 | 10.8 | 11.7 | 13.9 | 15.5 | 16.7 | 21.3 | 25.2 | 28.2 | 30.0 |
| 95-99 | n/a | n/a | n/a | n/a | n/a | n/a | n/a | n/a | n/a | n/a | n/a | n/a |
| 100+ | n/a | n/a | n/a | n/a | n/a | n/a | n/a | n/a | n/a | n/a | n/a | n/a |
| CHARLS = China Health and Retirement Longitudinal Study; SAGE = Study on Global AGEing and Adult Health | | | | | | | | | | | | |

**Supplementary file 19.** Reference values for handgrip strength for males in China (CHARLS, SAGE; pooled *n* = 18,944)

| **Age (years)** | ***n*** | **Percentile (kg)** | | | | | | | | | | |
| --- | --- | --- | --- | --- | --- | --- | --- | --- | --- | --- | --- | --- |
|  |  | **5^th^** | **10^th^** | **20^th^** | **30^th^** | **40^th^** | **50^th^** | **60^th^** | **70^th^** | **80^th^** | **90^th^** | **95^th^** |
| 20-24 | 38 | 25.9 | 30.0 | 33.0 | 36.0 | 40.0 | 40.0 | 42.0 | 44.0 | 45.0 | 50.0 | 51.2 |
| 25-29 | 50 | 18.0 | 21.8 | 29.6 | 33.7 | 38.0 | 41.5 | 42.4 | 46.0 | 50.0 | 52.2 | 55.1 |
| 30-34 | 55 | 14.1 | 19.2 | 28.8 | 34.2 | 37.6 | 42.0 | 46.0 | 47.8 | 50.0 | 54.6 | 56.6 |
| 35-39 | 60 | 15.9 | 25.8 | 31.6 | 36.7 | 41.0 | 42.0 | 44.2 | 48.0 | 50.0 | 54.2 | 60.0 |
| 40-44 | 642 | 29.2 | 33.6 | 37.0 | 40.0 | 42.0 | 44.0 | 45.9 | 48.0 | 51.0 | 54.0 | 57.9 |
| 45-49 | 2,934 | 30.0 | 33.0 | 37.5 | 40.0 | 42.0 | 44.0 | 45.5 | 48.0 | 50.0 | 54.0 | 57.5 |
| 50-54 | 3,052 | 25.0 | 30.0 | 34.0 | 36.5 | 39.0 | 41.0 | 43.0 | 45.0 | 48.1 | 52.0 | 55.0 |
| 55-59 | 3,344 | 23.5 | 28.0 | 31.5 | 34.5 | 37.0 | 39.2 | 41.0 | 43.0 | 46.0 | 50.0 | 52.9 |
| 60-64 | 3,231 | 22.0 | 26.0 | 30.0 | 33.0 | 35.0 | 37.2 | 39.5 | 41.5 | 44.0 | 48.0 | 51.0 |
| 65-69 | 2,290 | 20.0 | 23.5 | 28.0 | 30.0 | 32.5 | 35.0 | 37.0 | 39.0 | 42.0 | 45.0 | 48.0 |
| 70-74 | 1,575 | 17.0 | 20.5 | 25.0 | 28.0 | 30.0 | 32.0 | 34.0 | 35.8 | 38.5 | 41.9 | 44.5 |
| 75-79 | 1,074 | 14.4 | 18.0 | 22.0 | 25.0 | 27.1 | 29.8 | 31.0 | 33.0 | 36.0 | 39.5 | 41.5 |
| 80-84 | 445 | 11.1 | 14.0 | 19.0 | 22.0 | 24.0 | 26.6 | 29.0 | 30.0 | 32.5 | 37.0 | 40.0 |
| 85-89 | 125 | 9.2 | 12.0 | 14.9 | 20.0 | 22.0 | 24.0 | 25.8 | 28.0 | 30.0 | 34.4 | 37.9 |
| 90-94 | 29 | 8.8 | 10.0 | 12.0 | 16.0 | 18.6 | 20.0 | 22.0 | 25.3 | 29.1 | 31.6 | 36.4 |
| 95-99 | n/a | n/a | n/a | n/a | n/a | n/a | n/a | n/a | n/a | n/a | n/a | n/a |
| 100+ | n/a | n/a | n/a | n/a | n/a | n/a | n/a | n/a | n/a | n/a | n/a | n/a |
| CHARLS = China Health and Retirement Longitudinal Study; SAGE = Study on Global AGEing and Adult Health | | | | | | | | | | | | |

**Supplementary file 20.** Reference values for handgrip strength for females in India (LASI, SAGE; pooled *n* = 66,953)

| **Age (years)** | ***n*** | **Percentile (kg)** | | | | | | | | | | |
| --- | --- | --- | --- | --- | --- | --- | --- | --- | --- | --- | --- | --- |
|  |  | **5^th^** | **10^th^** | **20^th^** | **30^th^** | **40^th^** | **50^th^** | **60^th^** | **70^th^** | **80^th^** | **90^th^** | **95^th^** |
| 20-24 | 535 | 14.0 | 18.0 | 20.0 | 22.0 | 22.0 | 24.0 | 26.0 | 28.0 | 30.0 | 32.0 | 36.0 |
| 25-29 | 638 | 14.0 | 16.5 | 20.0 | 21.0 | 22.0 | 24.0 | 25.0 | 27.5 | 29.5 | 32.0 | 34.0 |
| 30-34 | 1,264 | 15.0 | 17.5 | 20.0 | 21.0 | 22.0 | 24.0 | 25.0 | 27.0 | 28.5 | 31.0 | 33.0 |
| 35-39 | 3,602 | 14.5 | 16.5 | 19.0 | 20.5 | 22.0 | 23.5 | 25.0 | 26.0 | 28.0 | 30.0 | 32.5 |
| 40-44 | 8,033 | 14.0 | 16.0 | 18.0 | 20.0 | 21.5 | 22.5 | 24.0 | 25.0 | 27.0 | 29.5 | 31.0 |
| 45-49 | 11,708 | 13.0 | 15.0 | 17.0 | 19.0 | 20.5 | 21.5 | 23.0 | 24.5 | 26.0 | 28.0 | 30.5 |
| 50-54 | 9,820 | 12.0 | 14.0 | 16.0 | 18.0 | 19.5 | 20.5 | 22.0 | 23.0 | 25.0 | 27.0 | 29.5 |
| 55-59 | 9,076 | 11.5 | 13.0 | 15.0 | 17.0 | 18.5 | 19.5 | 21.0 | 22.0 | 24.0 | 26.0 | 28.0 |
| 60-64 | 8,526 | 10.5 | 12.0 | 14.5 | 16.0 | 17.0 | 18.5 | 20.0 | 21.0 | 22.5 | 25.0 | 27.0 |
| 65-69 | 6,438 | 9.5 | 11.5 | 13.5 | 14.5 | 16.0 | 17.0 | 18.5 | 20.0 | 21.5 | 23.7 | 26.0 |
| 70-74 | 3,627 | 8.0 | 10.0 | 12.0 | 13.0 | 14.5 | 15.5 | 16.5 | 18.0 | 20.0 | 22.0 | 24.0 |
| 75-79 | 2,015 | 7.0 | 9.0 | 11.0 | 12.0 | 13.5 | 14.5 | 15.5 | 17.0 | 18.5 | 21.0 | 22.5 |
| 80-84 | 1,016 | 6.0 | 7.5 | 9.5 | 11.0 | 12.0 | 13.5 | 14.5 | 15.5 | 17.0 | 19.5 | 20.6 |
| 85-89 | 420 | 5.0 | 7.0 | 9.0 | 10.5 | 12.0 | 13.0 | 14.0 | 15.0 | 16.0 | 19.0 | 21.0 |
| 90-94 | 166 | 5.0 | 6.0 | 8.0 | 9.5 | 10.5 | 11.3 | 12.0 | 13.5 | 14.5 | 17.8 | 19.4 |
| 95-99 | 69 | 5.2 | 5.5 | 7.5 | 8.5 | 9.5 | 11.0 | 12.0 | 12.8 | 14.7 | 16.0 | 16.5 |
| 100+ | n/a | n/a | n/a | n/a | n/a | n/a | n/a | n/a | n/a | n/a | n/a | n/a |
| LASI = Longitudinal Ageing Study in India; SAGE = Study on Global AGEing and Adult Health | | | | | | | | | | | | |

**Supplementary file 21.** Reference values for handgrip strength for males in India (LASI, SAGE; pooled *n* = 54,525)

| **Age (years)** | ***n*** | **Percentile (kg)** | | | | | | | | | | |
| --- | --- | --- | --- | --- | --- | --- | --- | --- | --- | --- | --- | --- |
|  |  | **5^th^** | **10^th^** | **20^th^** | **30^th^** | **40^th^** | **50^th^** | **60^th^** | **70^th^** | **80^th^** | **90^th^** | **95^th^** |
| 20-24 | 127 | 26.3 | 30.0 | 31.2 | 33.0 | 36.0 | 37.0 | 40.0 | 42.0 | 45.6 | 49.4 | 53.7 |
| 25-29 | 125 | 24.0 | 28.0 | 30.0 | 33.0 | 36.0 | 38.0 | 39.0 | 40.8 | 44.0 | 48.0 | 51.6 |
| 30-34 | 138 | 20.0 | 25.5 | 30.0 | 32.0 | 34.0 | 36.0 | 38.6 | 40.0 | 42.0 | 45.0 | 49.2 |
| 35-39 | 246 | 24.0 | 27.3 | 30.0 | 32.0 | 34.0 | 36.0 | 39.0 | 40.0 | 42.0 | 46.0 | 49.0 |
| 40-44 | 318 | 23.9 | 25.4 | 28.0 | 31.0 | 32.0 | 35.0 | 37.0 | 40.0 | 42.0 | 46.5 | 48.1 |
| 45-49 | 10,313 | 21.5 | 24.5 | 27.5 | 30.0 | 32.0 | 33.5 | 35.5 | 37.0 | 39.5 | 43.0 | 45.5 |
| 50-54 | 9,176 | 20.0 | 22.5 | 25.5 | 28.0 | 30.0 | 31.5 | 33.5 | 35.5 | 37.5 | 41.0 | 44.0 |
| 55-59 | 8,198 | 18.5 | 21.0 | 24.0 | 26.0 | 28.5 | 30.0 | 32.0 | 33.5 | 36.0 | 39.5 | 42.0 |
| 60-64 | 8,360 | 16.0 | 19.0 | 22.0 | 24.5 | 26.0 | 28.0 | 30.0 | 31.5 | 34.0 | 37.0 | 39.5 |
| 65-69 | 7,632 | 14.3 | 17.0 | 20.5 | 22.5 | 24.5 | 26.0 | 28.0 | 29.5 | 32.0 | 35.0 | 38.0 |
| 70-74 | 4,923 | 13.0 | 15.0 | 18.0 | 20.0 | 22.0 | 24.0 | 25.5 | 27.5 | 30.0 | 33.0 | 35.5 |
| 75-79 | 2,720 | 12.0 | 14.0 | 16.5 | 19.0 | 20.5 | 22.5 | 24.5 | 26.0 | 28.5 | 31.5 | 33.5 |
| 80-84 | 1,405 | 10.5 | 12.5 | 15.0 | 17.0 | 19.0 | 20.5 | 22.5 | 24.5 | 26.5 | 30.0 | 32.5 |
| 85-89 | 560 | 9.5 | 11.0 | 13.5 | 15.0 | 17.0 | 18.5 | 20.0 | 21.5 | 23.5 | 27.0 | 30.0 |
| 90-94 | 200 | 8.9 | 10.0 | 12.5 | 14.0 | 15.5 | 17.0 | 18.5 | 20.0 | 22.0 | 25.5 | 28.5 |
| 95-99 | 60 | 6.0 | 7.0 | 11.9 | 14.4 | 15.3 | 16.5 | 18.0 | 18.5 | 20.0 | 22.0 | 23.6 |
| 100+ | 24 | 10.2 | 11.8 | 12.8 | 13.5 | 15.3 | 16.5 | 19.5 | 19.5 | 20.2 | 20.5 | 20.9 |
| LASI = Longitudinal Ageing Study in India; SAGE = Study on Global AGEing and Adult Health | | | | | | | | | | | | |

**Supplementary file 22.** Reference values for handgrip strength for females in Indonesia (IFLS; pooled *n* = 13,518)

| **Age (years)** | ***n*** | **Percentile (kg)** | | | | | | | | | | |
| --- | --- | --- | --- | --- | --- | --- | --- | --- | --- | --- | --- | --- |
|  |  | **5^th^** | **10^th^** | **20^th^** | **30^th^** | **40^th^** | **50^th^** | **60^th^** | **70^th^** | **80^th^** | **90^th^** | **95^th^** |
| 20-24 | 2,065 | 11.0 | 16.0 | 20.0 | 23.0 | 24.0 | 26.0 | 28.0 | 30.0 | 32.0 | 34.0 | 36.0 |
| 25-29 | 2,318 | 10.0 | 15.0 | 20.0 | 23.0 | 25.0 | 26.0 | 28.0 | 30.0 | 32.0 | 36.0 | 38.0 |
| 30-34 | 1,778 | 12.0 | 16.0 | 20.0 | 23.0 | 25.0 | 26.0 | 28.0 | 30.0 | 32.0 | 36.0 | 38.0 |
| 35-39 | 1,551 | 11.0 | 17.0 | 21.0 | 23.0 | 25.0 | 27.0 | 28.0 | 30.0 | 32.0 | 36.0 | 38.0 |
| 40-44 | 1,285 | 10.0 | 16.0 | 20.0 | 22.0 | 24.0 | 26.0 | 28.0 | 30.0 | 32.0 | 35.0 | 37.0 |
| 45-49 | 1,173 | 8.6 | 12.0 | 18.0 | 20.0 | 23.0 | 24.0 | 26.0 | 28.0 | 30.0 | 34.0 | 36.0 |
| 50-54 | 929 | 8.0 | 12.0 | 16.0 | 19.0 | 20.0 | 22.0 | 24.0 | 26.0 | 28.0 | 31.0 | 34.0 |
| 55-59 | 669 | 10.0 | 12.0 | 16.0 | 18.0 | 20.0 | 22.0 | 23.0 | 25.0 | 27.0 | 30.0 | 33.0 |
| 60-64 | 527 | 6.0 | 10.0 | 14.0 | 16.0 | 18.0 | 20.0 | 22.0 | 23.0 | 25.0 | 28.0 | 30.0 |
| 65-69 | 526 | 6.0 | 9.0 | 12.0 | 14.5 | 16.0 | 18.0 | 20.0 | 22.0 | 24.0 | 26.0 | 28.0 |
| 70-74 | 314 | 2.7 | 6.6 | 10.0 | 12.0 | 15.0 | 17.0 | 19.0 | 20.0 | 21.4 | 24.0 | 26.0 |
| 75-79 | 210 | 4.0 | 8.0 | 10.0 | 12.0 | 13.0 | 14.0 | 16.0 | 18.0 | 20.0 | 23.1 | 26.6 |
| 80-84 | 126 | 5.3 | 8.0 | 10.0 | 12.0 | 12.0 | 14.5 | 16.0 | 18.0 | 20.0 | 24.0 | 25.8 |
| 85-89 | 47 | 4.0 | 4.0 | 6.0 | 10.0 | 12.0 | 12.0 | 14.0 | 17.0 | 19.0 | 22.0 | 22.0 |
| 90-94 | n/a | n/a | n/a | n/a | n/a | n/a | n/a | n/a | n/a | n/a | n/a | n/a |
| 95-99 | n/a | n/a | n/a | n/a | n/a | n/a | n/a | n/a | n/a | n/a | n/a | n/a |
| 100+ | n/a | n/a | n/a | n/a | n/a | n/a | n/a | n/a | n/a | n/a | n/a | n/a |
| IFLS = Indonesian Family Life Survey | | | | | | | | | | | | |

**Supplementary file 23.** Reference values for handgrip strength for males in Indonesia (IFLS; pooled *n* = 12,132)

| **Age (years)** | ***n*** | **Percentile (kg)** | | | | | | | | | | |
| --- | --- | --- | --- | --- | --- | --- | --- | --- | --- | --- | --- | --- |
|  |  | **5^th^** | **10^th^** | **20^th^** | **30^th^** | **40^th^** | **50^th^** | **60^th^** | **70^th^** | **80^th^** | **90^th^** | **95^th^** |
| 20-24 | 1,591 | 26.0 | 30.0 | 34.0 | 38.0 | 40.0 | 41.0 | 43.0 | 45.0 | 48.0 | 52.0 | 54.0 |
| 25-29 | 2,025 | 26.0 | 30.0 | 35.0 | 38.0 | 40.0 | 42.0 | 43.0 | 46.0 | 49.0 | 52.0 | 56.0 |
| 30-34 | 1,750 | 26.0 | 30.0 | 35.0 | 38.0 | 40.0 | 42.0 | 43.0 | 46.0 | 48.0 | 52.0 | 56.0 |
| 35-39 | 1,577 | 26.0 | 30.0 | 35.0 | 38.0 | 40.0 | 41.0 | 43.0 | 45.2 | 48.0 | 52.0 | 54.0 |
| 40-44 | 1,189 | 24.0 | 30.0 | 33.0 | 36.0 | 38.0 | 40.0 | 42.0 | 44.0 | 46.0 | 50.0 | 53.0 |
| 45-49 | 1,054 | 20.7 | 26.0 | 30.0 | 34.0 | 37.0 | 38.0 | 40.0 | 42.0 | 44.0 | 48.0 | 50.0 |
| 50-54 | 820 | 18.0 | 23.9 | 29.0 | 32.0 | 34.0 | 36.0 | 38.0 | 40.0 | 42.0 | 46.0 | 49.1 |
| 55-59 | 656 | 13.0 | 20.0 | 26.0 | 30.0 | 32.0 | 35.0 | 37.0 | 39.0 | 41.0 | 44.0 | 48.0 |
| 60-64 | 443 | 12.0 | 16.0 | 22.0 | 26.0 | 29.0 | 30.0 | 32.0 | 35.0 | 38.0 | 41.0 | 44.0 |
| 65-69 | 448 | 10.4 | 15.7 | 21.0 | 24.0 | 26.0 | 29.0 | 31.0 | 32.0 | 36.0 | 38.0 | 41.0 |
| 70-74 | 267 | 9.0 | 12.0 | 18.0 | 21.0 | 23.0 | 26.0 | 28.0 | 30.0 | 32.0 | 37.0 | 40.0 |
| 75-79 | 159 | 8.0 | 10.8 | 15.0 | 19.0 | 20.0 | 22.0 | 25.0 | 28.0 | 30.4 | 33.2 | 35.0 |
| 80-84 | 101 | 5.0 | 8.0 | 10.0 | 16.0 | 20.0 | 20.0 | 22.0 | 25.0 | 27.0 | 31.0 | 34.0 |
| 85-89 | 52 | 6.2 | 10.0 | 12.0 | 13.3 | 18.8 | 20.0 | 22.0 | 23.7 | 28.0 | 32.9 | 35.0 |
| 90-94 | n/a | n/a | n/a | n/a | n/a | n/a | n/a | n/a | n/a | n/a | n/a | n/a |
| 95-99 | n/a | n/a | n/a | n/a | n/a | n/a | n/a | n/a | n/a | n/a | n/a | n/a |
| 100+ | n/a | n/a | n/a | n/a | n/a | n/a | n/a | n/a | n/a | n/a | n/a | n/a |
| IFLS = Indonesian Family Life Survey | | | | | | | | | | | | |

**Supplementary file 24.** Reference values for handgrip strength for females in Israel (SHARE; pooled *n* = 2,684)

| **Age (years)** | ***n*** | **Percentile (kg)** | | | | | | | | | | |
| --- | --- | --- | --- | --- | --- | --- | --- | --- | --- | --- | --- | --- |
|  |  | **5^th^** | **10^th^** | **20^th^** | **30^th^** | **40^th^** | **50^th^** | **60^th^** | **70^th^** | **80^th^** | **90^th^** | **95^th^** |
| 20-24 | n/a | n/a | n/a | n/a | n/a | n/a | n/a | n/a | n/a | n/a | n/a | n/a |
| 25-29 | n/a | n/a | n/a | n/a | n/a | n/a | n/a | n/a | n/a | n/a | n/a | n/a |
| 30-34 | n/a | n/a | n/a | n/a | n/a | n/a | n/a | n/a | n/a | n/a | n/a | n/a |
| 35-39 | n/a | n/a | n/a | n/a | n/a | n/a | n/a | n/a | n/a | n/a | n/a | n/a |
| 40-44 | 22 | 23.1 | 24.1 | 28.0 | 29.3 | 31.4 | 32.0 | 33.0 | 33.0 | 34.8 | 39.8 | 40.0 |
| 45-49 | 119 | 17.0 | 20.0 | 23.0 | 25.0 | 26.2 | 28.0 | 29.8 | 31.0 | 33.4 | 35.2 | 40.0 |
| 50-54 | 417 | 15.8 | 18.0 | 20.0 | 22.0 | 24.0 | 25.0 | 27.0 | 29.0 | 30.0 | 35.0 | 36.0 |
| 55-59 | 609 | 13.0 | 17.0 | 20.0 | 23.0 | 24.0 | 25.0 | 27.0 | 29.0 | 30.0 | 33.2 | 35.0 |
| 60-64 | 475 | 14.0 | 16.0 | 20.0 | 22.0 | 23.0 | 25.0 | 26.0 | 28.0 | 30.0 | 32.0 | 34.3 |
| 65-69 | 365 | 11.2 | 15.0 | 17.0 | 20.0 | 21.0 | 22.0 | 25.0 | 25.0 | 28.0 | 30.0 | 32.0 |
| 70-74 | 291 | 11.0 | 14.0 | 16.0 | 18.0 | 20.0 | 22.0 | 23.0 | 25.0 | 27.0 | 29.0 | 30.0 |
| 75-79 | 216 | 10.0 | 12.0 | 14.0 | 15.0 | 17.0 | 19.0 | 20.0 | 21.0 | 24.0 | 25.0 | 27.0 |
| 80-84 | 130 | 5.0 | 7.9 | 11.8 | 14.0 | 16.0 | 18.0 | 19.4 | 21.0 | 22.0 | 25.0 | 26.6 |
| 85-89 | 40 | 7.0 | 8.9 | 10.0 | 10.7 | 14.0 | 15.0 | 16.4 | 19.3 | 21.0 | 24.0 | 24.1 |
| 90-94 | n/a | n/a | n/a | n/a | n/a | n/a | n/a | n/a | n/a | n/a | n/a | n/a |
| 95-99 | n/a | n/a | n/a | n/a | n/a | n/a | n/a | n/a | n/a | n/a | n/a | n/a |
| 100+ | n/a | n/a | n/a | n/a | n/a | n/a | n/a | n/a | n/a | n/a | n/a | n/a |
| SHARE = Survey of Health, Ageing and Retirement in Europe | | | | | | | | | | | | |

**Supplementary file 25.** Reference values for handgrip strength for males in Israel (SHARE; pooled *n* = 2,414)

| **Age (years)** | ***n*** | **Percentile (kg)** | | | | | | | | | | |
| --- | --- | --- | --- | --- | --- | --- | --- | --- | --- | --- | --- | --- |
|  |  | **5^th^** | **10^th^** | **20^th^** | **30^th^** | **40^th^** | **50^th^** | **60^th^** | **70^th^** | **80^th^** | **90^th^** | **95^th^** |
| 20-24 | n/a | n/a | n/a | n/a | n/a | n/a | n/a | n/a | n/a | n/a | n/a | n/a |
| 25-29 | n/a | n/a | n/a | n/a | n/a | n/a | n/a | n/a | n/a | n/a | n/a | n/a |
| 30-34 | n/a | n/a | n/a | n/a | n/a | n/a | n/a | n/a | n/a | n/a | n/a | n/a |
| 35-39 | n/a | n/a | n/a | n/a | n/a | n/a | n/a | n/a | n/a | n/a | n/a | n/a |
| 40-44 | n/a | n/a | n/a | n/a | n/a | n/a | n/a | n/a | n/a | n/a | n/a | n/a |
| 45-49 | n/a | n/a | n/a | n/a | n/a | n/a | n/a | n/a | n/a | n/a | n/a | n/a |
| 50-54 | 251 | 25.0 | 32.0 | 38.0 | 40.0 | 44.0 | 47.0 | 50.0 | 50.0 | 54.0 | 55.0 | 58.0 |
| 55-59 | 503 | 26.1 | 30.0 | 35.0 | 39.0 | 40.0 | 44.0 | 45.0 | 48.0 | 50.0 | 55.0 | 58.0 |
| 60-64 | 445 | 26.0 | 30.0 | 35.0 | 38.0 | 40.0 | 42.0 | 44.0 | 46.0 | 50.0 | 53.6 | 55.0 |
| 65-69 | 374 | 20.0 | 25.0 | 30.0 | 33.0 | 35.0 | 38.0 | 40.0 | 43.0 | 46.0 | 50.0 | 52.0 |
| 70-74 | 365 | 17.2 | 23.0 | 28.0 | 31.0 | 34.0 | 36.0 | 39.0 | 40.0 | 43.2 | 46.6 | 50.0 |
| 75-79 | 255 | 18.0 | 20.0 | 24.0 | 27.0 | 28.6 | 30.0 | 33.4 | 35.0 | 40.0 | 44.0 | 47.0 |
| 80-84 | 155 | 15.7 | 18.0 | 21.8 | 25.0 | 28.0 | 30.0 | 32.0 | 34.0 | 36.0 | 40.0 | 43.0 |
| 85-89 | 66 | 12.8 | 17.0 | 20.0 | 22.5 | 26.0 | 27.0 | 30.0 | 31.5 | 35.0 | 40.0 | 43.0 |
| 90-94 | n/a | n/a | n/a | n/a | n/a | n/a | n/a | n/a | n/a | n/a | n/a | n/a |
| 95-99 | n/a | n/a | n/a | n/a | n/a | n/a | n/a | n/a | n/a | n/a | n/a | n/a |
| 100+ | n/a | n/a | n/a | n/a | n/a | n/a | n/a | n/a | n/a | n/a | n/a | n/a |
| SHARE = Survey of Health, Ageing and Retirement in Europe | | | | | | | | | | | | |

**Supplementary file 26.** Reference values for handgrip strength for females in Japan (NSJE, NUJLSA; pooled *n* = 2,232)

| **Age (years)** | ***n*** | **Percentile (kg)** | | | | | | | | | | |
| --- | --- | --- | --- | --- | --- | --- | --- | --- | --- | --- | --- | --- |
|  |  | **5^th^** | **10^th^** | **20^th^** | **30^th^** | **40^th^** | **50^th^** | **60^th^** | **70^th^** | **80^th^** | **90^th^** | **95^th^** |
| 20-24 | n/a | n/a | n/a | n/a | n/a | n/a | n/a | n/a | n/a | n/a | n/a | n/a |
| 25-29 | n/a | n/a | n/a | n/a | n/a | n/a | n/a | n/a | n/a | n/a | n/a | n/a |
| 30-34 | n/a | n/a | n/a | n/a | n/a | n/a | n/a | n/a | n/a | n/a | n/a | n/a |
| 35-39 | n/a | n/a | n/a | n/a | n/a | n/a | n/a | n/a | n/a | n/a | n/a | n/a |
| 40-44 | n/a | n/a | n/a | n/a | n/a | n/a | n/a | n/a | n/a | n/a | n/a | n/a |
| 45-49 | n/a | n/a | n/a | n/a | n/a | n/a | n/a | n/a | n/a | n/a | n/a | n/a |
| 50-54 | n/a | n/a | n/a | n/a | n/a | n/a | n/a | n/a | n/a | n/a | n/a | n/a |
| 55-59 | n/a | n/a | n/a | n/a | n/a | n/a | n/a | n/a | n/a | n/a | n/a | n/a |
| 60-64 | 141 | 17.5 | 19.0 | 21.0 | 21.5 | 23.0 | 24.0 | 25.0 | 25.5 | 26.5 | 28.0 | 29.5 |
| 65-69 | 121 | 16.5 | 18.5 | 20.3 | 21.5 | 22.5 | 23.0 | 24.0 | 25.0 | 26.0 | 27.5 | 28.5 |
| 70-74 | 454 | 15.0 | 16.5 | 18.0 | 19.5 | 20.2 | 21.0 | 22.0 | 23.2 | 24.5 | 26.0 | 27.0 |
| 75-79 | 497 | 12.5 | 15.0 | 17.0 | 18.5 | 19.5 | 20.0 | 21.0 | 21.6 | 22.5 | 24.5 | 26.0 |
| 80-84 | 493 | 11.5 | 13.0 | 15.0 | 16.0 | 17.5 | 18.5 | 19.5 | 20.0 | 21.0 | 23.0 | 25.0 |
| 85-89 | 411 | 9.7 | 10.5 | 12.5 | 14.0 | 15.0 | 16.0 | 17.0 | 18.5 | 19.5 | 21.0 | 22.0 |
| 90-94 | 115 | 9.0 | 10.0 | 11.0 | 12.3 | 13.1 | 14.1 | 15.5 | 16.5 | 18.0 | 19.7 | 21.2 |
| 95-99 | n/a | n/a | n/a | n/a | n/a | n/a | n/a | n/a | n/a | n/a | n/a | n/a |
| 100+ | n/a | n/a | n/a | n/a | n/a | n/a | n/a | n/a | n/a | n/a | n/a | n/a |
| NSJE = National Survey of the Japanese Elderly; NUJLSA = Nihon University Japanese Longitudinal Study of Aging | | | | | | | | | | | | |

**Supplementary file 27.** Reference values for handgrip strength for males in Japan (NSJE, NUJLSA; pooled *n* = 1,985)

| **Age (years)** | ***n*** | **Percentile (kg)** | | | | | | | | | | |
| --- | --- | --- | --- | --- | --- | --- | --- | --- | --- | --- | --- | --- |
|  |  | **5^th^** | **10^th^** | **20^th^** | **30^th^** | **40^th^** | **50^th^** | **60^th^** | **70^th^** | **80^th^** | **90^th^** | **95^th^** |
| 20-24 | n/a | n/a | n/a | n/a | n/a | n/a | n/a | n/a | n/a | n/a | n/a | n/a |
| 25-29 | n/a | n/a | n/a | n/a | n/a | n/a | n/a | n/a | n/a | n/a | n/a | n/a |
| 30-34 | n/a | n/a | n/a | n/a | n/a | n/a | n/a | n/a | n/a | n/a | n/a | n/a |
| 35-39 | n/a | n/a | n/a | n/a | n/a | n/a | n/a | n/a | n/a | n/a | n/a | n/a |
| 40-44 | n/a | n/a | n/a | n/a | n/a | n/a | n/a | n/a | n/a | n/a | n/a | n/a |
| 45-49 | n/a | n/a | n/a | n/a | n/a | n/a | n/a | n/a | n/a | n/a | n/a | n/a |
| 50-54 | n/a | n/a | n/a | n/a | n/a | n/a | n/a | n/a | n/a | n/a | n/a | n/a |
| 55-59 | n/a | n/a | n/a | n/a | n/a | n/a | n/a | n/a | n/a | n/a | n/a | n/a |
| 60-64 | 141 | 28.0 | 30.0 | 33.5 | 35.0 | 36.5 | 38.0 | 39.5 | 40.0 | 42.0 | 45.0 | 46.5 |
| 65-69 | 148 | 25.0 | 28.4 | 31.5 | 32.5 | 34.0 | 35.0 | 36.5 | 38.9 | 40.0 | 42.1 | 43.8 |
| 70-74 | 434 | 23.8 | 25.1 | 28.0 | 29.7 | 31.0 | 32.0 | 34.0 | 35.1 | 37.3 | 40.0 | 41.5 |
| 75-79 | 514 | 21.0 | 23.0 | 25.8 | 27.5 | 29.5 | 30.5 | 31.9 | 33.0 | 35.0 | 38.0 | 39.7 |
| 80-84 | 427 | 18.8 | 21.0 | 22.5 | 24.5 | 25.5 | 27.1 | 28.5 | 30.0 | 31.5 | 34.0 | 36.4 |
| 85-89 | 267 | 17.2 | 18.7 | 20.5 | 22.0 | 23.0 | 25.0 | 26.0 | 28.0 | 29.0 | 30.5 | 33.4 |
| 90-94 | 54 | 15.7 | 16.7 | 18.8 | 20.0 | 20.6 | 22.0 | 23.0 | 24.1 | 25.7 | 27.0 | 28.0 |
| 95-99 | n/a | n/a | n/a | n/a | n/a | n/a | n/a | n/a | n/a | n/a | n/a | n/a |
| 100+ | n/a | n/a | n/a | n/a | n/a | n/a | n/a | n/a | n/a | n/a | n/a | n/a |
| NSJE = National Survey of the Japanese Elderly; NUJLSA = Nihon University Japanese Longitudinal Study of Aging | | | | | | | | | | | | |

**Supplementary file 28.** Reference values for handgrip strength for females in Lebanon (LSAHA; pooled *n* = 1,241)

| **Age (years)** | ***n*** | **Percentile (kg)** | | | | | | | | | | |
| --- | --- | --- | --- | --- | --- | --- | --- | --- | --- | --- | --- | --- |
|  |  | **5^th^** | **10^th^** | **20^th^** | **30^th^** | **40^th^** | **50^th^** | **60^th^** | **70^th^** | **80^th^** | **90^th^** | **95^th^** |
| 20-24 | n/a | n/a | n/a | n/a | n/a | n/a | n/a | n/a | n/a | n/a | n/a | n/a |
| 25-29 | n/a | n/a | n/a | n/a | n/a | n/a | n/a | n/a | n/a | n/a | n/a | n/a |
| 30-34 | n/a | n/a | n/a | n/a | n/a | n/a | n/a | n/a | n/a | n/a | n/a | n/a |
| 35-39 | n/a | n/a | n/a | n/a | n/a | n/a | n/a | n/a | n/a | n/a | n/a | n/a |
| 40-44 | n/a | n/a | n/a | n/a | n/a | n/a | n/a | n/a | n/a | n/a | n/a | n/a |
| 45-49 | n/a | n/a | n/a | n/a | n/a | n/a | n/a | n/a | n/a | n/a | n/a | n/a |
| 50-54 | n/a | n/a | n/a | n/a | n/a | n/a | n/a | n/a | n/a | n/a | n/a | n/a |
| 55-59 | 21 | 14.0 | 16.0 | 18.0 | 18.0 | 20.0 | 21.0 | 23.0 | 26.0 | 30.0 | 38.0 | 55.0 |
| 60-64 | 369 | 11.0 | 14.0 | 18.0 | 20.0 | 20.0 | 21.0 | 22.0 | 25.0 | 28.0 | 30.0 | 34.0 |
| 65-69 | 329 | 10.0 | 12.0 | 14.0 | 16.4 | 19.0 | 20.0 | 20.0 | 22.0 | 25.0 | 30.0 | 33.6 |
| 70-74 | 230 | 10.0 | 10.5 | 12.0 | 15.7 | 18.0 | 19.0 | 20.0 | 20.0 | 22.2 | 28.0 | 30.0 |
| 75-79 | 163 | 10.0 | 10.0 | 12.0 | 14.0 | 15.8 | 18.0 | 19.0 | 20.0 | 20.0 | 24.0 | 26.0 |
| 80-84 | 100 | 10.0 | 10.0 | 11.0 | 12.0 | 15.0 | 16.0 | 19.0 | 20.0 | 21.0 | 25.1 | 30.0 |
| 85-89 | 29 | 10.0 | 10.0 | 10.6 | 12.0 | 14.0 | 18.0 | 18.0 | 20.0 | 20.0 | 20.0 | 20.0 |
| 90-94 | n/a | n/a | n/a | n/a | n/a | n/a | n/a | n/a | n/a | n/a | n/a | n/a |
| 95-99 | n/a | n/a | n/a | n/a | n/a | n/a | n/a | n/a | n/a | n/a | n/a | n/a |
| 100+ | n/a | n/a | n/a | n/a | n/a | n/a | n/a | n/a | n/a | n/a | n/a | n/a |
| LSAHA = Lebanon Study on Aging and HeAlth | | | | | | | | | | | | |

**Supplementary file 29.** Reference values for handgrip strength for males in Lebanon (LSAHA; pooled *n* = 758)

| **Age (years)** | ***n*** | **Percentile (kg)** | | | | | | | | | | |
| --- | --- | --- | --- | --- | --- | --- | --- | --- | --- | --- | --- | --- |
|  |  | **5^th^** | **10^th^** | **20^th^** | **30^th^** | **40^th^** | **50^th^** | **60^th^** | **70^th^** | **80^th^** | **90^th^** | **95^th^** |
| 20-24 | n/a | n/a | n/a | n/a | n/a | n/a | n/a | n/a | n/a | n/a | n/a | n/a |
| 25-29 | n/a | n/a | n/a | n/a | n/a | n/a | n/a | n/a | n/a | n/a | n/a | n/a |
| 30-34 | n/a | n/a | n/a | n/a | n/a | n/a | n/a | n/a | n/a | n/a | n/a | n/a |
| 35-39 | n/a | n/a | n/a | n/a | n/a | n/a | n/a | n/a | n/a | n/a | n/a | n/a |
| 40-44 | n/a | n/a | n/a | n/a | n/a | n/a | n/a | n/a | n/a | n/a | n/a | n/a |
| 45-49 | n/a | n/a | n/a | n/a | n/a | n/a | n/a | n/a | n/a | n/a | n/a | n/a |
| 50-54 | n/a | n/a | n/a | n/a | n/a | n/a | n/a | n/a | n/a | n/a | n/a | n/a |
| 55-59 | n/a | n/a | n/a | n/a | n/a | n/a | n/a | n/a | n/a | n/a | n/a | n/a |
| 60-64 | 174 | 19.5 | 20.0 | 25.0 | 29.0 | 30.0 | 33.0 | 36.0 | 39.0 | 40.0 | 45.0 | 49.0 |
| 65-69 | 191 | 16.0 | 20.0 | 25.0 | 28.0 | 30.0 | 30.0 | 33.0 | 36.0 | 39.0 | 40.0 | 44.5 |
| 70-74 | 167 | 14.0 | 18.0 | 21.0 | 24.0 | 27.4 | 29.0 | 30.0 | 30.7 | 32.0 | 38.0 | 40.0 |
| 75-79 | 112 | 15.5 | 19.0 | 20.0 | 22.0 | 26.6 | 28.0 | 30.0 | 31.0 | 34.0 | 38.2 | 40.0 |
| 80-84 | 86 | 13.1 | 16.4 | 20.0 | 20.6 | 22.0 | 25.0 | 28.0 | 29.4 | 30.0 | 34.0 | 36.9 |
| 85-89 | 28 | 11.0 | 11.0 | 14.0 | 18.1 | 20.0 | 22.0 | 24.4 | 27.8 | 29.0 | 30.0 | 30.0 |
| 90-94 | n/a | n/a | n/a | n/a | n/a | n/a | n/a | n/a | n/a | n/a | n/a | n/a |
| 95-99 | n/a | n/a | n/a | n/a | n/a | n/a | n/a | n/a | n/a | n/a | n/a | n/a |
| 100+ | n/a | n/a | n/a | n/a | n/a | n/a | n/a | n/a | n/a | n/a | n/a | n/a |
| LSAHA = Lebanon Study on Aging and HeAlth | | | | | | | | | | | | |

**Supplementary file 30.** Reference values for handgrip strength for females in Malaysia (MARS; pooled *n* = 3,004)

| **Age (years)** | ***n*** | **Percentile (kg)** | | | | | | | | | | |
| --- | --- | --- | --- | --- | --- | --- | --- | --- | --- | --- | --- | --- |
|  |  | **5^th^** | **10^th^** | **20^th^** | **30^th^** | **40^th^** | **50^th^** | **60^th^** | **70^th^** | **80^th^** | **90^th^** | **95^th^** |
| 20-24 | n/a | n/a | n/a | n/a | n/a | n/a | n/a | n/a | n/a | n/a | n/a | n/a |
| 25-29 | n/a | n/a | n/a | n/a | n/a | n/a | n/a | n/a | n/a | n/a | n/a | n/a |
| 30-34 | n/a | n/a | n/a | n/a | n/a | n/a | n/a | n/a | n/a | n/a | n/a | n/a |
| 35-39 | n/a | n/a | n/a | n/a | n/a | n/a | n/a | n/a | n/a | n/a | n/a | n/a |
| 40-44 | 421 | 8.5 | 10.4 | 12.9 | 14.9 | 16.9 | 18.6 | 20.3 | 22.3 | 24.2 | 26.2 | 27.6 |
| 45-49 | 435 | 8.3 | 10.3 | 12.9 | 15.2 | 17.0 | 18.5 | 20.6 | 22.5 | 24.9 | 27.9 | 29.9 |
| 50-54 | 498 | 7.2 | 9.4 | 12.3 | 14.2 | 16.0 | 17.2 | 18.9 | 20.8 | 23.2 | 26.2 | 28.7 |
| 55-59 | 489 | 7.5 | 9.8 | 13.0 | 14.5 | 16.1 | 17.6 | 18.9 | 20.7 | 22.5 | 25.1 | 27.2 |
| 60-64 | 430 | 6.7 | 8.9 | 12.3 | 14.5 | 16.2 | 17.9 | 19.4 | 21.0 | 23.0 | 26.0 | 29.7 |
| 65-69 | 313 | 6.3 | 8.7 | 11.3 | 13.3 | 15.1 | 16.8 | 18.5 | 20.3 | 22.4 | 24.6 | 26.9 |
| 70-74 | 190 | 7.1 | 8.8 | 11.5 | 13.4 | 14.7 | 16.4 | 18.5 | 19.8 | 21.6 | 24.1 | 27.9 |
| 75-79 | 125 | 8.7 | 10.4 | 13.5 | 15.0 | 15.9 | 16.9 | 19.1 | 20.2 | 22.1 | 26.2 | 28.7 |
| 80-84 | 83 | 7.7 | 9.6 | 13.4 | 14.8 | 16.5 | 16.9 | 18.4 | 20.0 | 21.5 | 23.7 | 25.7 |
| 85-89 | 20 | 10.5 | 13.1 | 15.0 | 15.5 | 17.7 | 18.3 | 19.0 | 20.3 | 22.3 | 23.8 | 24.5 |
| 90-94 | n/a | n/a | n/a | n/a | n/a | n/a | n/a | n/a | n/a | n/a | n/a | n/a |
| 95-99 | n/a | n/a | n/a | n/a | n/a | n/a | n/a | n/a | n/a | n/a | n/a | n/a |
| 100+ | n/a | n/a | n/a | n/a | n/a | n/a | n/a | n/a | n/a | n/a | n/a | n/a |
| MARS = Malaysia Ageing and Retirement Survey | | | | | | | | | | | | |

**Supplementary file 31.** Reference values for handgrip strength for males in Malaysia (MARS; pooled *n* = 2,402)

| **Age (years)** | ***n*** | **Percentile (kg)** | | | | | | | | | | |
| --- | --- | --- | --- | --- | --- | --- | --- | --- | --- | --- | --- | --- |
|  |  | **5^th^** | **10^th^** | **20^th^** | **30^th^** | **40^th^** | **50^th^** | **60^th^** | **70^th^** | **80^th^** | **90^th^** | **95^th^** |
| 20-24 | n/a | n/a | n/a | n/a | n/a | n/a | n/a | n/a | n/a | n/a | n/a | n/a |
| 25-29 | n/a | n/a | n/a | n/a | n/a | n/a | n/a | n/a | n/a | n/a | n/a | n/a |
| 30-34 | n/a | n/a | n/a | n/a | n/a | n/a | n/a | n/a | n/a | n/a | n/a | n/a |
| 35-39 | n/a | n/a | n/a | n/a | n/a | n/a | n/a | n/a | n/a | n/a | n/a | n/a |
| 40-44 | 277 | 11.7 | 14.2 | 17.5 | 22.5 | 27.0 | 29.3 | 32.6 | 35.3 | 38.5 | 41.8 | 44.9 |
| 45-49 | 344 | 12.2 | 15.1 | 18.7 | 22.9 | 26.7 | 29.2 | 32.0 | 34.4 | 37.8 | 41.4 | 44.5 |
| 50-54 | 402 | 12.6 | 15.6 | 20.2 | 23.7 | 26.7 | 28.9 | 31.5 | 35.1 | 38.4 | 42.9 | 45.4 |
| 55-59 | 379 | 9.6 | 13.7 | 19.6 | 23.0 | 25.7 | 28.3 | 31.0 | 34.3 | 37.3 | 40.9 | 45.0 |
| 60-64 | 394 | 11.2 | 14.1 | 18.3 | 22.5 | 25.3 | 28.1 | 30.2 | 32.8 | 36.4 | 41.1 | 47.2 |
| 65-69 | 264 | 12.0 | 15.8 | 20.0 | 23.2 | 26.6 | 28.3 | 30.3 | 32.8 | 35.2 | 39.6 | 44.3 |
| 70-74 | 197 | 8.8 | 12.1 | 17.1 | 20.8 | 23.0 | 25.0 | 27.5 | 30.6 | 33.6 | 36.7 | 40.3 |
| 75-79 | 100 | 11.0 | 13.4 | 18.3 | 22.4 | 24.0 | 26.7 | 31.2 | 34.0 | 36.4 | 40.2 | 45.1 |
| 80-84 | 45 | 10.7 | 13.7 | 17.2 | 20.0 | 23.2 | 24.5 | 26.4 | 29.0 | 35.0 | 43.0 | 45.6 |
| 85-89 | n/a | n/a | n/a | n/a | n/a | n/a | n/a | n/a | n/a | n/a | n/a | n/a |
| 90-94 | n/a | n/a | n/a | n/a | n/a | n/a | n/a | n/a | n/a | n/a | n/a | n/a |
| 95-99 | n/a | n/a | n/a | n/a | n/a | n/a | n/a | n/a | n/a | n/a | n/a | n/a |
| 100+ | n/a | n/a | n/a | n/a | n/a | n/a | n/a | n/a | n/a | n/a | n/a | n/a |
| MARS = Malaysia Ageing and Retirement Survey | | | | | | | | | | | | |

**Supplementary file 32.** Reference values for handgrip strength for females in Philippines (LSAHP, PSA; pooled *n* = 5,125)

| **Age (years)** | ***n*** | **Percentile (kg)** | | | | | | | | | | |
| --- | --- | --- | --- | --- | --- | --- | --- | --- | --- | --- | --- | --- |
|  |  | **5^th^** | **10^th^** | **20^th^** | **30^th^** | **40^th^** | **50^th^** | **60^th^** | **70^th^** | **80^th^** | **90^th^** | **95^th^** |
| 20-24 | n/a | n/a | n/a | n/a | n/a | n/a | n/a | n/a | n/a | n/a | n/a | n/a |
| 25-29 | n/a | n/a | n/a | n/a | n/a | n/a | n/a | n/a | n/a | n/a | n/a | n/a |
| 30-34 | n/a | n/a | n/a | n/a | n/a | n/a | n/a | n/a | n/a | n/a | n/a | n/a |
| 35-39 | n/a | n/a | n/a | n/a | n/a | n/a | n/a | n/a | n/a | n/a | n/a | n/a |
| 40-44 | n/a | n/a | n/a | n/a | n/a | n/a | n/a | n/a | n/a | n/a | n/a | n/a |
| 45-49 | n/a | n/a | n/a | n/a | n/a | n/a | n/a | n/a | n/a | n/a | n/a | n/a |
| 50-54 | n/a | n/a | n/a | n/a | n/a | n/a | n/a | n/a | n/a | n/a | n/a | n/a |
| 55-59 | n/a | n/a | n/a | n/a | n/a | n/a | n/a | n/a | n/a | n/a | n/a | n/a |
| 60-64 | 1,077 | 12.0 | 14.0 | 15.5 | 17.0 | 18.0 | 19.0 | 20.0 | 21.0 | 22.0 | 24.0 | 25.0 |
| 65-69 | 960 | 10.5 | 12.0 | 14.5 | 15.9 | 17.0 | 18.0 | 19.0 | 20.0 | 21.0 | 22.0 | 25.0 |
| 70-74 | 1,178 | 10.0 | 11.5 | 13.0 | 15.0 | 16.0 | 17.0 | 18.0 | 19.0 | 20.0 | 21.0 | 23.0 |
| 75-79 | 812 | 9.0 | 11.0 | 12.0 | 14.0 | 15.0 | 15.9 | 16.9 | 18.0 | 19.0 | 20.0 | 22.0 |
| 80-84 | 692 | 7.0 | 9.0 | 11.0 | 12.0 | 13.0 | 14.0 | 15.0 | 16.0 | 17.0 | 19.0 | 20.0 |
| 85-89 | 313 | 5.3 | 8.0 | 10.0 | 11.0 | 12.0 | 13.0 | 14.0 | 15.0 | 16.0 | 18.0 | 20.0 |
| 90-94 | 93 | 6.0 | 6.6 | 9.0 | 10.0 | 11.2 | 12.0 | 13.0 | 14.4 | 15.9 | 17.0 | 18.4 |
| 95-99 | n/a | n/a | n/a | n/a | n/a | n/a | n/a | n/a | n/a | n/a | n/a | n/a |
| 100+ | n/a | n/a | n/a | n/a | n/a | n/a | n/a | n/a | n/a | n/a | n/a | n/a |
| LSAHP = Longitudinal Study of Ageing and Health in the Philippines; PSA = Philippine Study on Aging | | | | | | | | | | | | |

**Supplementary file 33.** Reference values for handgrip strength for males in Philippines (LSAHP, PSA; pooled *n* = 3,210)

| **Age (years)** | ***n*** | **Percentile (kg)** | | | | | | | | | | |
| --- | --- | --- | --- | --- | --- | --- | --- | --- | --- | --- | --- | --- |
|  |  | **5^th^** | **10^th^** | **20^th^** | **30^th^** | **40^th^** | **50^th^** | **60^th^** | **70^th^** | **80^th^** | **90^th^** | **95^th^** |
| 20-24 | n/a | n/a | n/a | n/a | n/a | n/a | n/a | n/a | n/a | n/a | n/a | n/a |
| 25-29 | n/a | n/a | n/a | n/a | n/a | n/a | n/a | n/a | n/a | n/a | n/a | n/a |
| 30-34 | n/a | n/a | n/a | n/a | n/a | n/a | n/a | n/a | n/a | n/a | n/a | n/a |
| 35-39 | n/a | n/a | n/a | n/a | n/a | n/a | n/a | n/a | n/a | n/a | n/a | n/a |
| 40-44 | n/a | n/a | n/a | n/a | n/a | n/a | n/a | n/a | n/a | n/a | n/a | n/a |
| 45-49 | n/a | n/a | n/a | n/a | n/a | n/a | n/a | n/a | n/a | n/a | n/a | n/a |
| 50-54 | n/a | n/a | n/a | n/a | n/a | n/a | n/a | n/a | n/a | n/a | n/a | n/a |
| 55-59 | n/a | n/a | n/a | n/a | n/a | n/a | n/a | n/a | n/a | n/a | n/a | n/a |
| 60-64 | 881 | 18.5 | 20.5 | 24.0 | 25.3 | 27.0 | 28.0 | 29.5 | 30.5 | 32.0 | 34.0 | 36.0 |
| 65-69 | 685 | 16.5 | 19.0 | 21.5 | 23.3 | 25.0 | 26.0 | 28.0 | 29.0 | 30.0 | 32.5 | 34.0 |
| 70-74 | 766 | 15.0 | 17.5 | 20.0 | 21.0 | 23.0 | 24.0 | 26.0 | 27.0 | 29.0 | 31.0 | 32.0 |
| 75-79 | 418 | 13.0 | 15.0 | 18.0 | 20.0 | 21.0 | 22.0 | 24.0 | 25.0 | 27.0 | 29.0 | 30.0 |
| 80-84 | 294 | 10.0 | 12.0 | 15.0 | 17.0 | 19.0 | 20.0 | 21.0 | 22.0 | 24.0 | 27.0 | 28.0 |
| 85-89 | 140 | 9.0 | 10.9 | 14.0 | 16.0 | 18.0 | 19.0 | 20.0 | 21.0 | 22.0 | 24.0 | 26.0 |
| 90-94 | 26 | 7.5 | 9.0 | 11.0 | 14.4 | 15.0 | 15.0 | 16.0 | 17.0 | 18.0 | 19.0 | 19.0 |
| 95-99 | n/a | n/a | n/a | n/a | n/a | n/a | n/a | n/a | n/a | n/a | n/a | n/a |
| 100+ | n/a | n/a | n/a | n/a | n/a | n/a | n/a | n/a | n/a | n/a | n/a | n/a |
| LSAHP = Longitudinal Study of Ageing and Health in the Philippines; PSA = Philippine Study on Aging | | | | | | | | | | | | |

**Supplementary file 34.** Reference values for handgrip strength for females in the Republic of Korea (KLoSA, KNHANES; pooled *n* = 27,293)

| **Age (years)** | ***n*** | **Percentile (kg)** | | | | | | | | | | |
| --- | --- | --- | --- | --- | --- | --- | --- | --- | --- | --- | --- | --- |
|  |  | **5^th^** | **10^th^** | **20^th^** | **30^th^** | **40^th^** | **50^th^** | **60^th^** | **70^th^** | **80^th^** | **90^th^** | **95^th^** |
| 20-24 | 1,150 | 17.0 | 19.0 | 20.0 | 22.0 | 23.0 | 24.0 | 25.0 | 27.0 | 28.0 | 31.0 | 33.0 |
| 25-29 | 1,139 | 17.0 | 18.0 | 21.0 | 22.0 | 24.0 | 25.0 | 26.0 | 27.0 | 29.0 | 30.0 | 32.0 |
| 30-34 | 1,416 | 18.0 | 20.0 | 22.0 | 23.0 | 24.0 | 26.0 | 27.0 | 28.0 | 30.0 | 32.0 | 34.0 |
| 35-39 | 1,878 | 18.0 | 20.0 | 22.0 | 24.0 | 25.0 | 26.0 | 27.0 | 29.0 | 30.0 | 32.0 | 34.0 |
| 40-44 | 1,934 | 18.0 | 20.0 | 22.0 | 24.0 | 25.0 | 26.0 | 27.0 | 28.0 | 30.0 | 32.0 | 34.0 |
| 45-49 | 2,976 | 18.0 | 20.0 | 21.0 | 23.0 | 24.0 | 25.0 | 26.0 | 28.0 | 29.0 | 31.0 | 33.0 |
| 50-54 | 2,836 | 17.0 | 19.0 | 20.0 | 22.0 | 23.0 | 24.0 | 25.0 | 27.0 | 28.0 | 30.0 | 32.0 |
| 55-59 | 2,833 | 16.0 | 19.0 | 20.0 | 21.5 | 23.0 | 24.0 | 25.0 | 26.0 | 28.0 | 29.0 | 31.0 |
| 60-64 | 2,728 | 15.0 | 17.0 | 19.0 | 20.0 | 22.0 | 23.0 | 24.0 | 25.0 | 27.0 | 29.0 | 30.0 |
| 65-69 | 2,806 | 14.0 | 16.0 | 18.0 | 19.3 | 20.0 | 22.0 | 23.0 | 24.0 | 25.1 | 27.0 | 29.0 |
| 70-74 | 2,294 | 12.0 | 14.0 | 17.0 | 18.0 | 19.0 | 20.0 | 21.0 | 23.0 | 24.0 | 26.0 | 27.9 |
| 75-79 | 1,884 | 11.0 | 12.0 | 15.0 | 16.0 | 18.0 | 19.0 | 20.0 | 21.0 | 22.0 | 24.0 | 26.0 |
| 80-84 | 1,328 | 9.0 | 10.0 | 13.0 | 14.0 | 15.0 | 16.0 | 18.0 | 19.0 | 20.0 | 22.0 | 24.0 |
| 85-89 | 64 | 5.2 | 8.0 | 10.0 | 11.0 | 12.0 | 13.0 | 15.0 | 16.0 | 17.0 | 19.0 | 20.0 |
| 90-94 | 27 | 7.3 | 8.6 | 10.0 | 10.8 | 12.0 | 13.0 | 14.0 | 14.2 | 16.6 | 18.8 | 20.0 |
| 95-99 | n/a | n/a | n/a | n/a | n/a | n/a | n/a | n/a | n/a | n/a | n/a | n/a |
| 100+ | n/a | n/a | n/a | n/a | n/a | n/a | n/a | n/a | n/a | n/a | n/a | n/a |
| KLoSA = Korean Longitudinal Study of Aging; KNHANES = Korea National Health and Nutrition Examination Survey | | | | | | | | | | | | |

**Supplementary file 35.** Reference values for handgrip strength for males in the Republic of Korea (KLoSA, KNHANES; pooled *n* = 21,808)

| **Age (years)** | ***n*** | **Percentile (kg)** | | | | | | | | | | |
| --- | --- | --- | --- | --- | --- | --- | --- | --- | --- | --- | --- | --- |
|  |  | **5^th^** | **10^th^** | **20^th^** | **30^th^** | **40^th^** | **50^th^** | **60^th^** | **70^th^** | **80^th^** | **90^th^** | **95^th^** |
| 20-24 | 1,008 | 29.0 | 32.0 | 35.0 | 38.0 | 39.0 | 41.0 | 43.0 | 45.0 | 47.0 | 51.0 | 55.0 |
| 25-29 | 984 | 30.0 | 33.0 | 36.0 | 38.9 | 40.0 | 42.0 | 44.0 | 46.0 | 48.0 | 52.0 | 55.0 |
| 30-34 | 1,139 | 31.0 | 34.0 | 38.0 | 40.0 | 42.0 | 44.0 | 46.0 | 48.0 | 50.0 | 54.0 | 57.0 |
| 35-39 | 1,475 | 33.0 | 36.0 | 39.0 | 41.0 | 43.0 | 44.0 | 46.0 | 48.0 | 51.0 | 54.0 | 56.3 |
| 40-44 | 1,527 | 32.0 | 35.0 | 38.0 | 40.0 | 42.0 | 44.0 | 45.0 | 47.0 | 50.0 | 53.0 | 55.0 |
| 45-49 | 2,203 | 31.0 | 33.0 | 36.0 | 38.0 | 40.0 | 41.0 | 43.0 | 45.0 | 47.0 | 50.0 | 53.0 |
| 50-54 | 2,126 | 29.0 | 32.0 | 35.0 | 37.0 | 39.0 | 40.0 | 42.0 | 43.0 | 46.0 | 49.0 | 51.8 |
| 55-59 | 2,317 | 28.0 | 31.0 | 34.0 | 36.0 | 38.0 | 39.0 | 41.0 | 42.0 | 45.0 | 48.0 | 50.0 |
| 60-64 | 2,203 | 26.0 | 29.0 | 32.0 | 34.0 | 36.0 | 37.0 | 39.0 | 40.0 | 43.0 | 46.0 | 48.0 |
| 65-69 | 2,450 | 24.0 | 27.0 | 30.0 | 32.0 | 34.0 | 35.0 | 37.0 | 38.0 | 40.0 | 43.0 | 45.0 |
| 70-74 | 1,933 | 21.0 | 24.0 | 27.0 | 29.0 | 31.0 | 33.0 | 34.0 | 36.0 | 38.0 | 40.6 | 43.0 |
| 75-79 | 1,456 | 19.0 | 22.0 | 25.0 | 27.0 | 29.0 | 31.0 | 32.0 | 34.0 | 36.0 | 39.0 | 41.0 |
| 80-84 | 949 | 14.0 | 18.0 | 21.5 | 24.0 | 26.0 | 27.0 | 29.0 | 30.0 | 32.0 | 35.0 | 37.0 |
| 85-89 | 38 | 15.0 | 17.1 | 20.0 | 20.0 | 21.0 | 24.0 | 25.0 | 25.0 | 29.2 | 32.0 | 38.2 |
| 90-94 | n/a | n/a | n/a | n/a | n/a | n/a | n/a | n/a | n/a | n/a | n/a | n/a |
| 95-99 | n/a | n/a | n/a | n/a | n/a | n/a | n/a | n/a | n/a | n/a | n/a | n/a |
| 100+ | n/a | n/a | n/a | n/a | n/a | n/a | n/a | n/a | n/a | n/a | n/a | n/a |
| KLoSA = Korean Longitudinal Study of Aging; KNHANES = Korea National Health and Nutrition Examination Survey | | | | | | | | | | | | |

**Supplementary file 36.** Reference values for handgrip strength for females in Singapore (PIONEER, SIHLS, WiSE; *n* = 6,150)

| **Age (years)** | ***n*** | **Percentile (kg)** | | | | | | | | | | |
| --- | --- | --- | --- | --- | --- | --- | --- | --- | --- | --- | --- | --- |
|  |  | **5^th^** | **10^th^** | **20^th^** | **30^th^** | **40^th^** | **50^th^** | **60^th^** | **70^th^** | **80^th^** | **90^th^** | **95^th^** |
| 20-24 | n/a | n/a | n/a | n/a | n/a | n/a | n/a | n/a | n/a | n/a | n/a | n/a |
| 25-29 | n/a | n/a | n/a | n/a | n/a | n/a | n/a | n/a | n/a | n/a | n/a | n/a |
| 30-34 | n/a | n/a | n/a | n/a | n/a | n/a | n/a | n/a | n/a | n/a | n/a | n/a |
| 35-39 | n/a | n/a | n/a | n/a | n/a | n/a | n/a | n/a | n/a | n/a | n/a | n/a |
| 40-44 | n/a | n/a | n/a | n/a | n/a | n/a | n/a | n/a | n/a | n/a | n/a | n/a |
| 45-49 | n/a | n/a | n/a | n/a | n/a | n/a | n/a | n/a | n/a | n/a | n/a | n/a |
| 50-54 | n/a | n/a | n/a | n/a | n/a | n/a | n/a | n/a | n/a | n/a | n/a | n/a |
| 55-59 | n/a | n/a | n/a | n/a | n/a | n/a | n/a | n/a | n/a | n/a | n/a | n/a |
| 60-64 | 1,241 | 11.0 | 13.0 | 15.0 | 16.5 | 18.0 | 19.0 | 20.0 | 21.0 | 23.0 | 25.0 | 27.0 |
| 65-69 | 1,277 | 10.0 | 12.0 | 14.0 | 15.7 | 17.0 | 18.0 | 19.1 | 20.0 | 21.9 | 24.0 | 25.8 |
| 70-74 | 1,063 | 9.0 | 11.0 | 13.0 | 15.0 | 16.0 | 17.0 | 18.0 | 19.2 | 20.6 | 22.9 | 24.2 |
| 75-79 | 1,055 | 7.0 | 9.0 | 11.0 | 12.5 | 14.0 | 15.0 | 16.0 | 17.2 | 19.0 | 20.8 | 22.0 |
| 80-84 | 849 | 6.5 | 8.0 | 10.5 | 12.0 | 13.0 | 14.0 | 15.0 | 16.0 | 17.5 | 19.2 | 20.8 |
| 85-89 | 478 | 5.0 | 6.0 | 8.0 | 10.0 | 11.0 | 12.0 | 13.5 | 14.8 | 16.0 | 18.0 | 20.0 |
| 90-94 | 152 | 5.0 | 5.1 | 7.2 | 9.0 | 10.0 | 11.0 | 12.0 | 13.8 | 15.0 | 16.3 | 17.6 |
| 95-99 | 35 | 2.3 | 4.3 | 6.0 | 8.0 | 9.0 | 9.3 | 10.0 | 11.1 | 13.0 | 14.4 | 16.4 |
| 100+ | n/a | n/a | n/a | n/a | n/a | n/a | n/a | n/a | n/a | n/a | n/a | n/a |
| PHASE = Panel on Health and Ageing of Singaporean Elderly; PIONEER = PopulatION HEalth and Eye Disease PRofilE in Elderly Singaporeans Study; WiSE = Well-being of the Singapore Elderly | | | | | | | | | | | | |

**Supplementary file 37.** Reference values for handgrip strength for males in Singapore (PIONEER, SIHLS, and WiSE; *n* = 5,101)

| **Age (years)** | ***n*** | **Percentile (kg)** | | | | | | | | | | |
| --- | --- | --- | --- | --- | --- | --- | --- | --- | --- | --- | --- | --- |
|  |  | **5^th^** | **10^th^** | **20^th^** | **30^th^** | **40^th^** | **50^th^** | **60^th^** | **70^th^** | **80^th^** | **90^th^** | **95^th^** |
| 20-24 | n/a | n/a | n/a | n/a | n/a | n/a | n/a | n/a | n/a | n/a | n/a | n/a |
| 25-29 | n/a | n/a | n/a | n/a | n/a | n/a | n/a | n/a | n/a | n/a | n/a | n/a |
| 30-34 | n/a | n/a | n/a | n/a | n/a | n/a | n/a | n/a | n/a | n/a | n/a | n/a |
| 35-39 | n/a | n/a | n/a | n/a | n/a | n/a | n/a | n/a | n/a | n/a | n/a | n/a |
| 40-44 | n/a | n/a | n/a | n/a | n/a | n/a | n/a | n/a | n/a | n/a | n/a | n/a |
| 45-49 | n/a | n/a | n/a | n/a | n/a | n/a | n/a | n/a | n/a | n/a | n/a | n/a |
| 50-54 | n/a | n/a | n/a | n/a | n/a | n/a | n/a | n/a | n/a | n/a | n/a | n/a |
| 55-59 | n/a | n/a | n/a | n/a | n/a | n/a | n/a | n/a | n/a | n/a | n/a | n/a |
| 60-64 | 1,124 | 19.0 | 21.0 | 25.0 | 27.5 | 29.4 | 31.0 | 33.0 | 35.0 | 37.0 | 40.9 | 43.0 |
| 65-69 | 1,125 | 18.0 | 21.0 | 24.3 | 26.0 | 28.0 | 30.0 | 31.0 | 33.0 | 35.0 | 38.0 | 40.0 |
| 70-74 | 850 | 17.0 | 19.0 | 22.0 | 23.5 | 25.0 | 27.0 | 29.0 | 30.0 | 32.1 | 35.0 | 37.0 |
| 75-79 | 845 | 15.0 | 17.0 | 20.0 | 21.0 | 23.0 | 25.0 | 26.0 | 27.5 | 29.4 | 32.0 | 34.3 |
| 80-84 | 676 | 13.0 | 16.0 | 18.0 | 20.0 | 21.5 | 23.0 | 24.0 | 26.0 | 27.6 | 30.0 | 32.0 |
| 85-89 | 367 | 11.0 | 13.0 | 16.0 | 17.5 | 19.0 | 20.0 | 21.9 | 23.0 | 25.0 | 28.8 | 31.1 |
| 90-94 | 94 | 8.0 | 10.0 | 13.0 | 15.0 | 16.0 | 17.0 | 20.0 | 20.5 | 22.4 | 25.3 | 29.4 |
| 95-99 | 20 | 11.0 | 11.9 | 15.6 | 16.0 | 16.5 | 18.0 | 19.0 | 20.2 | 21.2 | 23.0 | 27.3 |
| 100+ | n/a | n/a | n/a | n/a | n/a | n/a | n/a | n/a | n/a | n/a | n/a | n/a |
| PHASE = Panel on Health and Ageing of Singaporean Elderly; PIONEER = PopulatION HEalth and Eye Disease PRofilE in Elderly Singaporeans Study; WiSE = Well-being of the Singapore Elderly | | | | | | | | | | | | |

**Supplementary file 38.** Reference values for handgrip strength for females in Sri Lanka (SLHAS; pooled *n* = 3,156)

| **Age (years)** | ***n*** | **Percentile (kg)** | | | | | | | | | | |
| --- | --- | --- | --- | --- | --- | --- | --- | --- | --- | --- | --- | --- |
|  |  | **5^th^** | **10^th^** | **20^th^** | **30^th^** | **40^th^** | **50^th^** | **60^th^** | **70^th^** | **80^th^** | **90^th^** | **95^th^** |
| 20-24 | 190 | 12.7 | 14.0 | 16.6 | 18.2 | 19.7 | 20.4 | 21.3 | 22.4 | 24.1 | 26.9 | 28.5 |
| 25-29 | 256 | 13.8 | 15.1 | 18.0 | 19.4 | 21.3 | 22.4 | 23.7 | 24.8 | 26.1 | 28.1 | 30.1 |
| 30-34 | 201 | 15.0 | 16.5 | 18.4 | 20.1 | 21.3 | 22.2 | 23.3 | 24.8 | 27.3 | 29.0 | 30.4 |
| 35-39 | 347 | 13.4 | 15.0 | 17.8 | 19.4 | 20.8 | 22.1 | 22.9 | 24.5 | 26.3 | 28.3 | 30.1 |
| 40-44 | 280 | 13.5 | 15.1 | 17.5 | 19.4 | 20.4 | 21.7 | 22.9 | 24.4 | 26.1 | 27.5 | 28.2 |
| 45-49 | 287 | 12.9 | 14.7 | 17.2 | 18.7 | 20.1 | 21.3 | 22.8 | 24.1 | 25.7 | 27.1 | 28.4 |
| 50-54 | 258 | 12.4 | 13.9 | 15.8 | 17.7 | 19.1 | 19.9 | 20.7 | 22.0 | 23.3 | 24.9 | 25.8 |
| 55-59 | 284 | 11.6 | 13.5 | 15.6 | 17.5 | 18.7 | 19.4 | 20.7 | 21.4 | 23.0 | 25.1 | 26.9 |
| 60-64 | 274 | 11.6 | 12.9 | 15.0 | 16.6 | 17.6 | 18.5 | 19.4 | 20.6 | 22.2 | 24.1 | 25.2 |
| 65-69 | 273 | 11.0 | 12.2 | 14.1 | 15.4 | 16.3 | 17.8 | 19.1 | 19.9 | 21.1 | 22.8 | 24.8 |
| 70-74 | 284 | 10.4 | 11.8 | 14.2 | 15.2 | 16.6 | 17.7 | 18.8 | 20.1 | 21.6 | 23.2 | 25.1 |
| 75-79 | 138 | 10.3 | 11.6 | 13.3 | 14.2 | 14.9 | 16.1 | 17.0 | 18.2 | 19.1 | 20.3 | 21.8 |
| 80-84 | 64 | 7.8 | 8.7 | 10.7 | 12.1 | 13.1 | 14.6 | 15.4 | 16.3 | 17.4 | 19.9 | 20.9 |
| 85-89 | 20 | 8.8 | 8.9 | 11.2 | 12.4 | 14.1 | 15.8 | 16.2 | 16.8 | 17.3 | 17.6 | 18.5 |
| 90-94 | n/a | n/a | n/a | n/a | n/a | n/a | n/a | n/a | n/a | n/a | n/a | n/a |
| 95-99 | n/a | n/a | n/a | n/a | n/a | n/a | n/a | n/a | n/a | n/a | n/a | n/a |
| 100+ | n/a | n/a | n/a | n/a | n/a | n/a | n/a | n/a | n/a | n/a | n/a | n/a |
| SLHAS = Sri Lanka Health and Ageing Study | | | | | | | | | | | | |

**Supplementary file 39.** Reference values for handgrip strength for males in Sri Lanka (SLHAS; pooled *n* = 3,047)

| **Age (years)** | ***n*** | **Percentile (kg)** | | | | | | | | | | |
| --- | --- | --- | --- | --- | --- | --- | --- | --- | --- | --- | --- | --- |
|  |  | **5^th^** | **10^th^** | **20^th^** | **30^th^** | **40^th^** | **50^th^** | **60^th^** | **70^th^** | **80^th^** | **90^th^** | **95^th^** |
| 20-24 | 174 | 24.9 | 26.6 | 28.9 | 31.0 | 32.4 | 34.5 | 36.3 | 37.8 | 39.6 | 41.5 | 45.0 |
| 25-29 | 174 | 26.1 | 27.6 | 30.9 | 32.3 | 34.0 | 36.1 | 37.5 | 39.6 | 41.7 | 44.3 | 47.1 |
| 30-34 | 186 | 23.0 | 26.5 | 30.3 | 31.9 | 33.6 | 35.7 | 37.8 | 39.1 | 40.7 | 43.8 | 45.3 |
| 35-39 | 366 | 24.3 | 26.8 | 29.3 | 31.6 | 32.7 | 34.4 | 36.6 | 39.0 | 40.9 | 43.9 | 47.0 |
| 40-44 | 286 | 24.0 | 26.1 | 29.1 | 31.0 | 32.6 | 34.2 | 35.4 | 37.6 | 40.0 | 42.7 | 45.4 |
| 45-49 | 293 | 22.3 | 25.2 | 27.4 | 29.9 | 31.6 | 32.8 | 34.4 | 36.8 | 38.8 | 41.6 | 44.9 |
| 50-54 | 267 | 22.1 | 23.5 | 26.6 | 28.7 | 30.1 | 31.6 | 33.0 | 34.6 | 36.0 | 38.4 | 40.5 |
| 55-59 | 295 | 17.7 | 19.9 | 23.1 | 25.5 | 27.4 | 29.0 | 30.4 | 32.1 | 33.8 | 36.8 | 38.8 |
| 60-64 | 298 | 16.0 | 19.1 | 22.8 | 24.9 | 26.3 | 27.8 | 28.8 | 30.1 | 31.8 | 34.6 | 37.1 |
| 65-69 | 253 | 18.5 | 20.5 | 22.4 | 23.8 | 25.1 | 26.6 | 28.1 | 29.9 | 32.7 | 35.1 | 37.5 |
| 70-74 | 238 | 15.1 | 18.1 | 20.4 | 22.2 | 23.3 | 24.6 | 25.9 | 27.6 | 29.3 | 31.7 | 33.7 |
| 75-79 | 152 | 15.3 | 16.9 | 18.5 | 20.4 | 21.8 | 23.1 | 24.6 | 25.8 | 27.8 | 29.7 | 31.5 |
| 80-84 | 65 | 11.5 | 13.8 | 16.4 | 18.1 | 19.4 | 21.6 | 22.9 | 23.8 | 24.8 | 26.2 | 28.5 |
| 85-89 | n/a | n/a | n/a | n/a | n/a | n/a | n/a | n/a | n/a | n/a | n/a | n/a |
| 90-94 | n/a | n/a | n/a | n/a | n/a | n/a | n/a | n/a | n/a | n/a | n/a | n/a |
| 95-99 | n/a | n/a | n/a | n/a | n/a | n/a | n/a | n/a | n/a | n/a | n/a | n/a |
| 100+ | n/a | n/a | n/a | n/a | n/a | n/a | n/a | n/a | n/a | n/a | n/a | n/a |
| SLHAS = Sri Lanka Health and Ageing Study | | | | | | | | | | | | |

**Supplementary file 40.** Percentile curves for 2.5 m gait speed for females in Asia (CHARLS, NSJE, PHASE, SHARE; pooled *n* = 6,792)


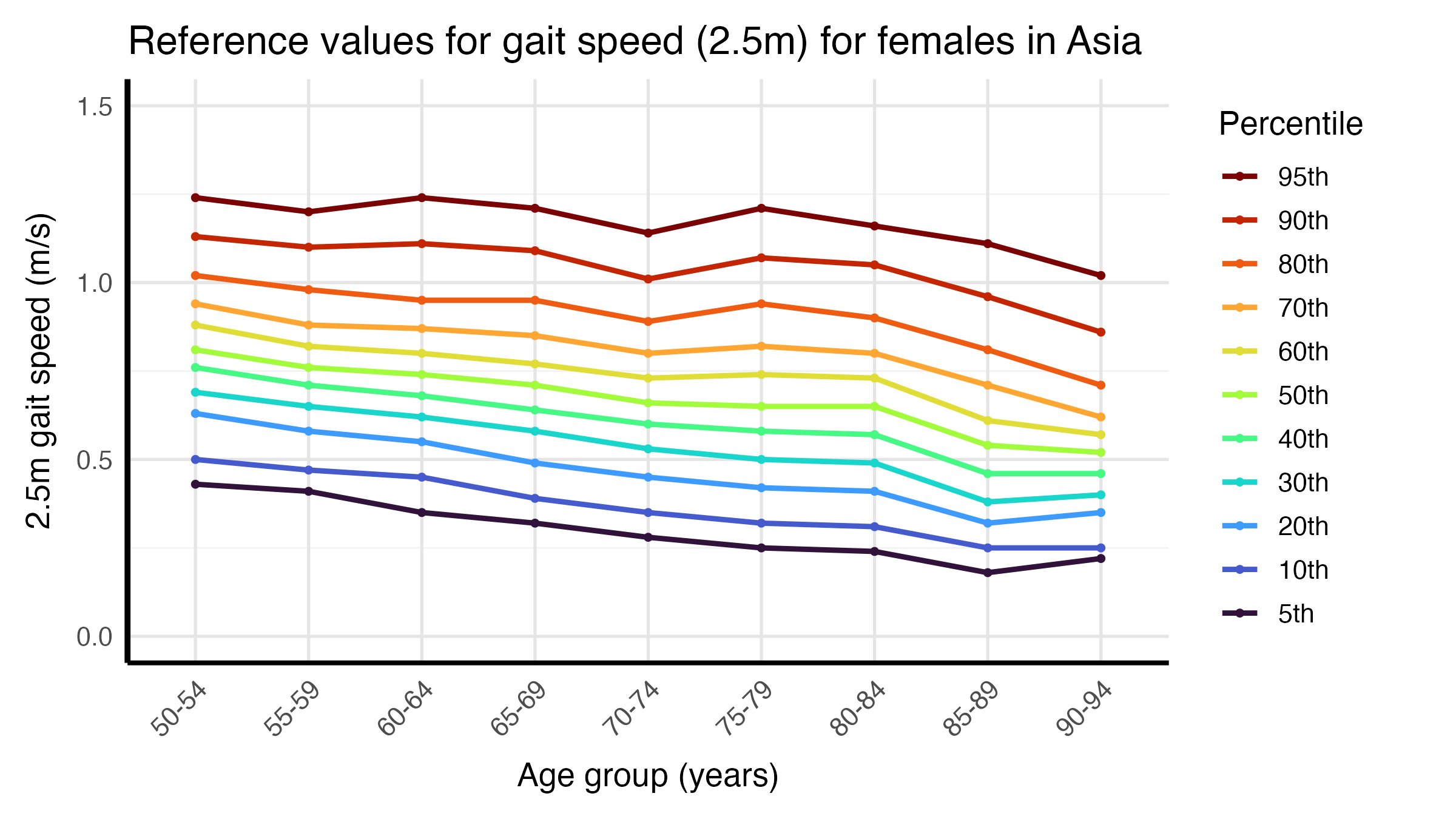


**Supplementary file 41.** Percentile curves for 2.5 m gait speed for males in Asia (CHARLS, NSJE, PHASE, SHARE; pooled *n* = 6,682)


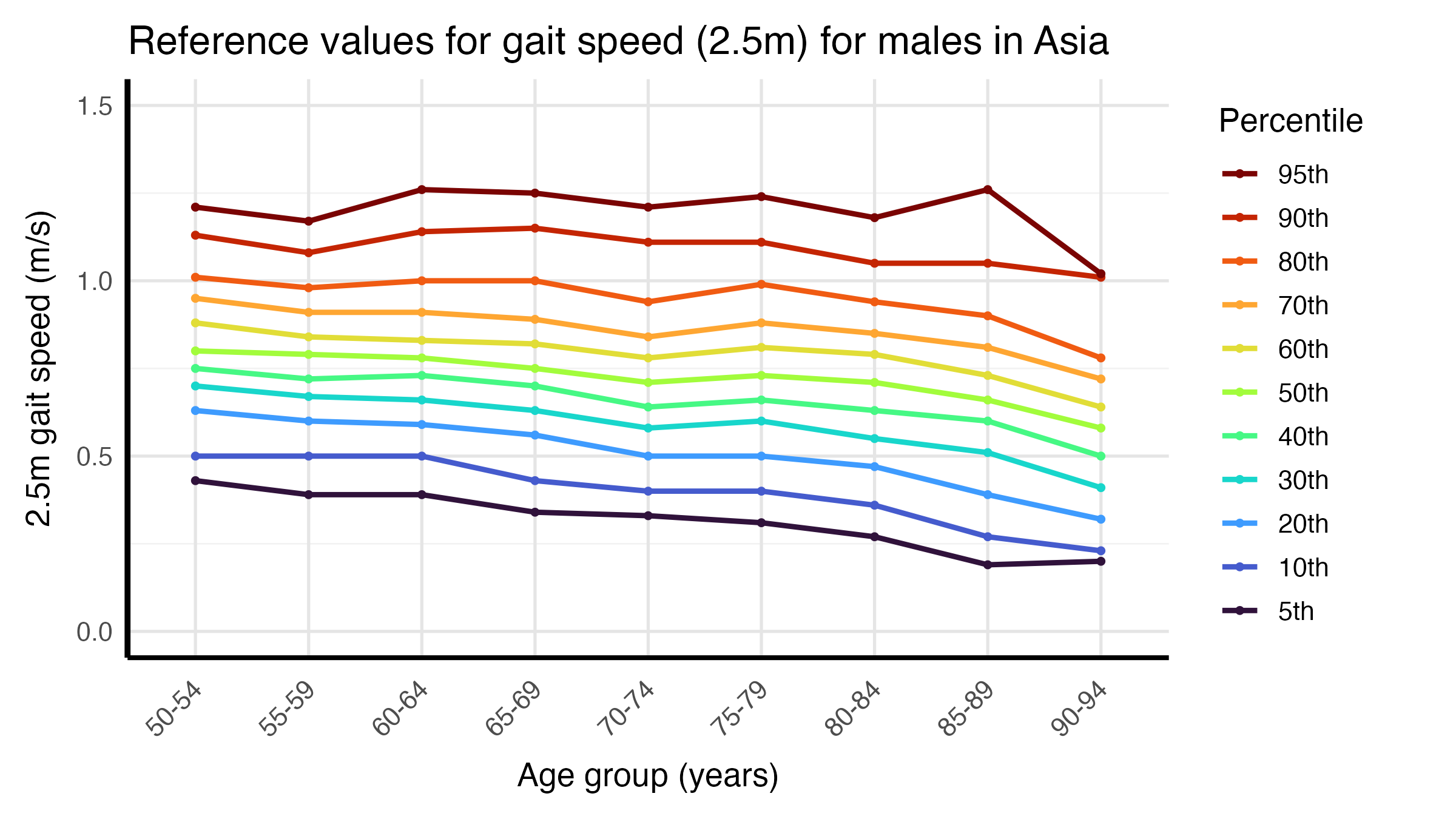


**Supplementary file 42.** Reference values for 2.5 m gait speed for females in East Asia (CHARLS, NSJE; pooled *n* = 4,989)

| **Age (years)** | ***n*** | **Percentile (m/s)** | | | | | | | | | | |
| --- | --- | --- | --- | --- | --- | --- | --- | --- | --- | --- | --- | --- |
|  |  | **5^th^** | **10^th^** | **20^th^** | **30^th^** | **40^th^** | **50^th^** | **60^th^** | **70^th^** | **80^th^** | **90^th^** | **95^th^** |
| 20-24 | n/a | n/a | n/a | n/a | n/a | n/a | n/a | n/a | n/a | n/a | n/a | n/a |
| 25-29 | n/a | n/a | n/a | n/a | n/a | n/a | n/a | n/a | n/a | n/a | n/a | n/a |
| 30-34 | n/a | n/a | n/a | n/a | n/a | n/a | n/a | n/a | n/a | n/a | n/a | n/a |
| 35-39 | n/a | n/a | n/a | n/a | n/a | n/a | n/a | n/a | n/a | n/a | n/a | n/a |
| 40-44 | n/a | n/a | n/a | n/a | n/a | n/a | n/a | n/a | n/a | n/a | n/a | n/a |
| 45-49 | n/a | n/a | n/a | n/a | n/a | n/a | n/a | n/a | n/a | n/a | n/a | n/a |
| 50-54 | n/a | n/a | n/a | n/a | n/a | n/a | n/a | n/a | n/a | n/a | n/a | n/a |
| 55-59 | n/a | n/a | n/a | n/a | n/a | n/a | n/a | n/a | n/a | n/a | n/a | n/a |
| 60-64 | 1,734 | 0.35 | 0.45 | 0.55 | 0.62 | 0.68 | 0.74 | 0.80 | 0.87 | 0.95 | 1.11 | 1.24 |
| 65-69 | 1,172 | 0.33 | 0.41 | 0.51 | 0.59 | 0.66 | 0.72 | 0.78 | 0.86 | 0.96 | 1.10 | 1.21 |
| 70-74 | 761 | 0.31 | 0.38 | 0.48 | 0.54 | 0.62 | 0.68 | 0.75 | 0.82 | 0.91 | 1.04 | 1.18 |
| 75-79 | 624 | 0.29 | 0.36 | 0.46 | 0.53 | 0.60 | 0.67 | 0.76 | 0.84 | 0.96 | 1.10 | 1.24 |
| 80-84 | 403 | 0.25 | 0.34 | 0.47 | 0.56 | 0.64 | 0.70 | 0.77 | 0.85 | 0.94 | 1.08 | 1.20 |
| 85-89 | 227 | 0.23 | 0.30 | 0.36 | 0.45 | 0.54 | 0.60 | 0.68 | 0.77 | 0.85 | 1.00 | 1.15 |
| 90-94 | 68 | 0.25 | 0.29 | 0.39 | 0.42 | 0.51 | 0.54 | 0.58 | 0.64 | 0.73 | 0.84 | 0.97 |
| 95-99 | n/a | n/a | n/a | n/a | n/a | n/a | n/a | n/a | n/a | n/a | n/a | n/a |
| 100+ | n/a | n/a | n/a | n/a | n/a | n/a | n/a | n/a | n/a | n/a | n/a | n/a |
| CHARLS = China Health and Retirement Longitudinal Study; NSJE = National Survey of the Japanese Elderly; | | | | | | | | | | | | |

**Supplementary file 43.** Reference values for 2.5 m gait speed for males in East Asia (CHARLS, NSJE; pooled *n* = 5,159)

| **Age (years)** | ***n*** | **Percentile (m/s)** | | | | | | | | | | |
| --- | --- | --- | --- | --- | --- | --- | --- | --- | --- | --- | --- | --- |
|  |  | **5^th^** | **10^th^** | **20^th^** | **30^th^** | **40^th^** | **50^th^** | **60^th^** | **70^th^** | **80^th^** | **90^th^** | **95^th^** |
| 20-24 | n/a | n/a | n/a | n/a | n/a | n/a | n/a | n/a | n/a | n/a | n/a | n/a |
| 25-29 | n/a | n/a | n/a | n/a | n/a | n/a | n/a | n/a | n/a | n/a | n/a | n/a |
| 30-34 | n/a | n/a | n/a | n/a | n/a | n/a | n/a | n/a | n/a | n/a | n/a | n/a |
| 35-39 | n/a | n/a | n/a | n/a | n/a | n/a | n/a | n/a | n/a | n/a | n/a | n/a |
| 40-44 | n/a | n/a | n/a | n/a | n/a | n/a | n/a | n/a | n/a | n/a | n/a | n/a |
| 45-49 | n/a | n/a | n/a | n/a | n/a | n/a | n/a | n/a | n/a | n/a | n/a | n/a |
| 50-54 | n/a | n/a | n/a | n/a | n/a | n/a | n/a | n/a | n/a | n/a | n/a | n/a |
| 55-59 | n/a | n/a | n/a | n/a | n/a | n/a | n/a | n/a | n/a | n/a | n/a | n/a |
| 60-64 | 1,766 | 0.39 | 0.50 | 0.59 | 0.66 | 0.73 | 0.78 | 0.83 | 0.91 | 1.00 | 1.14 | 1.26 |
| 65-69 | 1,236 | 0.36 | 0.46 | 0.57 | 0.64 | 0.70 | 0.76 | 0.82 | 0.89 | 1.00 | 1.15 | 1.25 |
| 70-74 | 868 | 0.33 | 0.41 | 0.51 | 0.59 | 0.65 | 0.73 | 0.79 | 0.86 | 0.95 | 1.11 | 1.21 |
| 75-79 | 699 | 0.32 | 0.42 | 0.53 | 0.62 | 0.70 | 0.75 | 0.83 | 0.90 | 1.01 | 1.13 | 1.25 |
| 80-84 | 401 | 0.31 | 0.42 | 0.53 | 0.60 | 0.69 | 0.76 | 0.83 | 0.89 | 0.98 | 1.13 | 1.23 |
| 85-89 | 158 | 0.23 | 0.37 | 0.50 | 0.59 | 0.66 | 0.72 | 0.81 | 0.87 | 0.99 | 1.13 | 1.29 |
| 90-94 | 31 | 0.33 | 0.39 | 0.50 | 0.54 | 0.58 | 0.63 | 0.72 | 0.72 | 0.79 | 1.01 | 1.02 |
| 95-99 | n/a | n/a | n/a | n/a | n/a | n/a | n/a | n/a | n/a | n/a | n/a | n/a |
| 100+ | n/a | n/a | n/a | n/a | n/a | n/a | n/a | n/a | n/a | n/a | n/a | n/a |
| CHARLS = China Health and Retirement Longitudinal Study; NSJE = National Survey of the Japanese Elderly; | | | | | | | | | | | | |

**Supplementary file 44.** Reference values for 2.5 m gait speed for females in China (CHARLS; pooled *n* = 5,526)

| **Age (years)** | ***n*** | **Percentile (m/s)** | | | | | | | | | | |
| --- | --- | --- | --- | --- | --- | --- | --- | --- | --- | --- | --- | --- |
|  |  | **5^th^** | **10^th^** | **20^th^** | **30^th^** | **40^th^** | **50^th^** | **60^th^** | **70^th^** | **80^th^** | **90^th^** | **95^th^** |
| 20-24 | n/a | n/a | n/a | n/a | n/a | n/a | n/a | n/a | n/a | n/a | n/a | n/a |
| 25-29 | n/a | n/a | n/a | n/a | n/a | n/a | n/a | n/a | n/a | n/a | n/a | n/a |
| 30-34 | n/a | n/a | n/a | n/a | n/a | n/a | n/a | n/a | n/a | n/a | n/a | n/a |
| 35-39 | 24 | 0.56 | 0.59 | 0.65 | 0.69 | 0.81 | 0.88 | 0.94 | 0.95 | 1.00 | 1.03 | 1.07 |
| 40-44 | 194 | 0.45 | 0.52 | 0.61 | 0.68 | 0.74 | 0.80 | 0.87 | 0.94 | 0.99 | 1.11 | 1.23 |
| 45-49 | 390 | 0.43 | 0.51 | 0.63 | 0.71 | 0.78 | 0.83 | 0.89 | 0.96 | 1.02 | 1.11 | 1.20 |
| 50-54 | 447 | 0.43 | 0.51 | 0.63 | 0.69 | 0.76 | 0.81 | 0.88 | 0.94 | 1.02 | 1.14 | 1.24 |
| 55-59 | 511 | 0.41 | 0.47 | 0.58 | 0.65 | 0.71 | 0.76 | 0.82 | 0.88 | 0.97 | 1.10 | 1.18 |
| 60-64 | 1,608 | 0.34 | 0.44 | 0.54 | 0.61 | 0.67 | 0.71 | 0.78 | 0.84 | 0.92 | 1.03 | 1.14 |
| 65-69 | 1,062 | 0.32 | 0.39 | 0.49 | 0.57 | 0.63 | 0.69 | 0.75 | 0.81 | 0.90 | 1.01 | 1.11 |
| 70-74 | 658 | 0.29 | 0.36 | 0.46 | 0.52 | 0.59 | 0.64 | 0.71 | 0.76 | 0.83 | 0.98 | 1.07 |
| 75-79 | 407 | 0.24 | 0.32 | 0.39 | 0.46 | 0.52 | 0.57 | 0.62 | 0.68 | 0.77 | 0.88 | 0.97 |
| 80-84 | 167 | 0.19 | 0.24 | 0.34 | 0.41 | 0.46 | 0.51 | 0.58 | 0.63 | 0.71 | 0.89 | 1.02 |
| 85-89 | 58 | 0.17 | 0.20 | 0.27 | 0.31 | 0.34 | 0.37 | 0.41 | 0.50 | 0.58 | 0.70 | 0.82 |
| 90-94 | n/a | n/a | n/a | n/a | n/a | n/a | n/a | n/a | n/a | n/a | n/a | n/a |
| 95-99 | n/a | n/a | n/a | n/a | n/a | n/a | n/a | n/a | n/a | n/a | n/a | n/a |
| 100+ | n/a | n/a | n/a | n/a | n/a | n/a | n/a | n/a | n/a | n/a | n/a | n/a |
| CHARLS = China Health and Retirement Longitudinal Study | | | | | | | | | | | | |

**Supplementary file 45.** Reference values for 2.5 m gait speed for males in China (CHARLS; pooled *n* = 5,515)

| **Age (years)** | ***n*** | **Percentile (m/s)** | | | | | | | | | | |
| --- | --- | --- | --- | --- | --- | --- | --- | --- | --- | --- | --- | --- |
|  |  | **5^th^** | **10^th^** | **20^th^** | **30^th^** | **40^th^** | **50^th^** | **60^th^** | **70^th^** | **80^th^** | **90^th^** | **95^th^** |
| 20-24 | n/a | n/a | n/a | n/a | n/a | n/a | n/a | n/a | n/a | n/a | n/a | n/a |
| 25-29 | n/a | n/a | n/a | n/a | n/a | n/a | n/a | n/a | n/a | n/a | n/a | n/a |
| 30-34 | n/a | n/a | n/a | n/a | n/a | n/a | n/a | n/a | n/a | n/a | n/a | n/a |
| 35-39 | n/a | n/a | n/a | n/a | n/a | n/a | n/a | n/a | n/a | n/a | n/a | n/a |
| 40-44 | 96 | 0.38 | 0.49 | 0.58 | 0.66 | 0.72 | 0.77 | 0.80 | 0.85 | 0.91 | 1.00 | 1.13 |
| 45-49 | 340 | 0.42 | 0.53 | 0.62 | 0.69 | 0.74 | 0.80 | 0.86 | 0.94 | 1.01 | 1.11 | 1.21 |
| 50-54 | 401 | 0.43 | 0.50 | 0.63 | 0.69 | 0.75 | 0.80 | 0.88 | 0.94 | 1.01 | 1.13 | 1.21 |
| 55-59 | 477 | 0.39 | 0.50 | 0.60 | 0.67 | 0.72 | 0.79 | 0.84 | 0.91 | 0.98 | 1.08 | 1.17 |
| 60-64 | 1,641 | 0.38 | 0.48 | 0.58 | 0.65 | 0.71 | 0.76 | 0.82 | 0.88 | 0.97 | 1.08 | 1.21 |
| 65-69 | 1,098 | 0.34 | 0.43 | 0.56 | 0.62 | 0.68 | 0.73 | 0.79 | 0.85 | 0.93 | 1.05 | 1.18 |
| 70-74 | 762 | 0.32 | 0.39 | 0.50 | 0.56 | 0.63 | 0.69 | 0.75 | 0.81 | 0.89 | 1.00 | 1.14 |
| 75-79 | 473 | 0.28 | 0.37 | 0.48 | 0.56 | 0.62 | 0.68 | 0.73 | 0.79 | 0.87 | 1.00 | 1.11 |
| 80-84 | 179 | 0.24 | 0.31 | 0.43 | 0.50 | 0.56 | 0.59 | 0.66 | 0.73 | 0.80 | 0.90 | 1.00 |
| 85-89 | 48 | 0.18 | 0.29 | 0.39 | 0.47 | 0.58 | 0.61 | 0.67 | 0.75 | 0.79 | 0.90 | 0.95 |
| 90-94 | n/a | n/a | n/a | n/a | n/a | n/a | n/a | n/a | n/a | n/a | n/a | n/a |
| 95-99 | n/a | n/a | n/a | n/a | n/a | n/a | n/a | n/a | n/a | n/a | n/a | n/a |
| 100+ | n/a | n/a | n/a | n/a | n/a | n/a | n/a | n/a | n/a | n/a | n/a | n/a |
| CHARLS = China Health and Retirement Longitudinal Study | | | | | | | | | | | | |

**Supplementary file 46.** Reference values for 2.5 m gait speed for females in Israel (SHARE; pooled *n* = 176)

| **Age (years)** | ***n*** | **Percentile (m/s)** | | | | | | | | | | |
| --- | --- | --- | --- | --- | --- | --- | --- | --- | --- | --- | --- | --- |
|  |  | **5^th^** | **10^th^** | **20^th^** | **30^th^** | **40^th^** | **50^th^** | **60^th^** | **70^th^** | **80^th^** | **90^th^** | **95^th^** |
| 20-24 | n/a | n/a | n/a | n/a | n/a | n/a | n/a | n/a | n/a | n/a | n/a | n/a |
| 25-29 | n/a | n/a | n/a | n/a | n/a | n/a | n/a | n/a | n/a | n/a | n/a | n/a |
| 30-34 | n/a | n/a | n/a | n/a | n/a | n/a | n/a | n/a | n/a | n/a | n/a | n/a |
| 35-39 | n/a | n/a | n/a | n/a | n/a | n/a | n/a | n/a | n/a | n/a | n/a | n/a |
| 40-44 | n/a | n/a | n/a | n/a | n/a | n/a | n/a | n/a | n/a | n/a | n/a | n/a |
| 45-49 | n/a | n/a | n/a | n/a | n/a | n/a | n/a | n/a | n/a | n/a | n/a | n/a |
| 50-54 | n/a | n/a | n/a | n/a | n/a | n/a | n/a | n/a | n/a | n/a | n/a | n/a |
| 55-59 | n/a | n/a | n/a | n/a | n/a | n/a | n/a | n/a | n/a | n/a | n/a | n/a |
| 60-64 | n/a | n/a | n/a | n/a | n/a | n/a | n/a | n/a | n/a | n/a | n/a | n/a |
| 65-69 | n/a | n/a | n/a | n/a | n/a | n/a | n/a | n/a | n/a | n/a | n/a | n/a |
| 70-74 | n/a | n/a | n/a | n/a | n/a | n/a | n/a | n/a | n/a | n/a | n/a | n/a |
| 75-79 | 97 | 0.25 | 0.29 | 0.38 | 0.47 | 0.60 | 0.67 | 0.81 | 0.85 | 1.00 | 1.08 | 1.16 |
| 80-84 | 58 | 0.24 | 0.31 | 0.40 | 0.47 | 0.57 | 0.66 | 0.70 | 0.81 | 0.86 | 1.06 | 1.17 |
| 85-89 | 21 | 0.23 | 0.25 | 0.36 | 0.36 | 0.40 | 0.42 | 0.50 | 0.52 | 0.58 | 0.78 | 1.05 |
| 90-94 | n/a | n/a | n/a | n/a | n/a | n/a | n/a | n/a | n/a | n/a | n/a | n/a |
| 95-99 | n/a | n/a | n/a | n/a | n/a | n/a | n/a | n/a | n/a | n/a | n/a | n/a |
| 100+ | n/a | n/a | n/a | n/a | n/a | n/a | n/a | n/a | n/a | n/a | n/a | n/a |
| SHARE = Survey of Health, Ageing and Retirement in Europe | | | | | | | | | | | | |

**Supplementary file 47.** Reference values for 2.5 m gait speed for males in Israel (SHARE; pooled *n* = 110)

| **Age (years)** | ***n*** | **Percentile (m/s)** | | | | | | | | | | |
| --- | --- | --- | --- | --- | --- | --- | --- | --- | --- | --- | --- | --- |
|  |  | **5^th^** | **10^th^** | **20^th^** | **30^th^** | **40^th^** | **50^th^** | **60^th^** | **70^th^** | **80^th^** | **90^th^** | **95^th^** |
| 20-24 | n/a | n/a | n/a | n/a | n/a | n/a | n/a | n/a | n/a | n/a | n/a | n/a |
| 25-29 | n/a | n/a | n/a | n/a | n/a | n/a | n/a | n/a | n/a | n/a | n/a | n/a |
| 30-34 | n/a | n/a | n/a | n/a | n/a | n/a | n/a | n/a | n/a | n/a | n/a | n/a |
| 35-39 | n/a | n/a | n/a | n/a | n/a | n/a | n/a | n/a | n/a | n/a | n/a | n/a |
| 40-44 | n/a | n/a | n/a | n/a | n/a | n/a | n/a | n/a | n/a | n/a | n/a | n/a |
| 45-49 | n/a | n/a | n/a | n/a | n/a | n/a | n/a | n/a | n/a | n/a | n/a | n/a |
| 50-54 | n/a | n/a | n/a | n/a | n/a | n/a | n/a | n/a | n/a | n/a | n/a | n/a |
| 55-59 | n/a | n/a | n/a | n/a | n/a | n/a | n/a | n/a | n/a | n/a | n/a | n/a |
| 60-64 | n/a | n/a | n/a | n/a | n/a | n/a | n/a | n/a | n/a | n/a | n/a | n/a |
| 65-69 | n/a | n/a | n/a | n/a | n/a | n/a | n/a | n/a | n/a | n/a | n/a | n/a |
| 70-74 | n/a | n/a | n/a | n/a | n/a | n/a | n/a | n/a | n/a | n/a | n/a | n/a |
| 75-79 | 63 | 0.26 | 0.42 | 0.49 | 0.57 | 0.63 | 0.70 | 0.83 | 0.92 | 1.03 | 1.17 | 1.24 |
| 80-84 | 47 | 0.38 | 0.44 | 0.54 | 0.59 | 0.63 | 0.71 | 0.83 | 0.92 | 1.02 | 1.10 | 1.18 |
| 85-89 | n/a | n/a | n/a | n/a | n/a | n/a | n/a | n/a | n/a | n/a | n/a | n/a |
| 90-94 | n/a | n/a | n/a | n/a | n/a | n/a | n/a | n/a | n/a | n/a | n/a | n/a |
| 95-99 | n/a | n/a | n/a | n/a | n/a | n/a | n/a | n/a | n/a | n/a | n/a | n/a |
| 100+ | n/a | n/a | n/a | n/a | n/a | n/a | n/a | n/a | n/a | n/a | n/a | n/a |
| SHARE = Survey of Health, Ageing and Retirement in Europe | | | | | | | | | | | | |

**Supplementary file 48.** Reference values for 2.5 m gait speed for females in Japan (NSJE; pooled *n* = 983)

| **Age (years)** | ***n*** | **Percentile (m/s)** | | | | | | | | | | |
| --- | --- | --- | --- | --- | --- | --- | --- | --- | --- | --- | --- | --- |
|  |  | **5^th^** | **10^th^** | **20^th^** | **30^th^** | **40^th^** | **50^th^** | **60^th^** | **70^th^** | **80^th^** | **90^th^** | **95^th^** |
| 20-24 | n/a | n/a | n/a | n/a | n/a | n/a | n/a | n/a | n/a | n/a | n/a | n/a |
| 25-29 | n/a | n/a | n/a | n/a | n/a | n/a | n/a | n/a | n/a | n/a | n/a | n/a |
| 30-34 | n/a | n/a | n/a | n/a | n/a | n/a | n/a | n/a | n/a | n/a | n/a | n/a |
| 35-39 | n/a | n/a | n/a | n/a | n/a | n/a | n/a | n/a | n/a | n/a | n/a | n/a |
| 40-44 | n/a | n/a | n/a | n/a | n/a | n/a | n/a | n/a | n/a | n/a | n/a | n/a |
| 45-49 | n/a | n/a | n/a | n/a | n/a | n/a | n/a | n/a | n/a | n/a | n/a | n/a |
| 50-54 | n/a | n/a | n/a | n/a | n/a | n/a | n/a | n/a | n/a | n/a | n/a | n/a |
| 55-59 | n/a | n/a | n/a | n/a | n/a | n/a | n/a | n/a | n/a | n/a | n/a | n/a |
| 60-64 | 96 | 0.72 | 0.86 | 0.94 | 0.99 | 1.08 | 1.12 | 1.18 | 1.24 | 1.34 | 1.50 | 1.64 |
| 65-69 | 110 | 0.70 | 0.81 | 0.94 | 1.00 | 1.06 | 1.11 | 1.16 | 1.22 | 1.34 | 1.41 | 1.56 |
| 70-74 | 103 | 0.63 | 0.72 | 0.80 | 0.87 | 0.90 | 0.97 | 1.01 | 1.10 | 1.20 | 1.24 | 1.33 |
| 75-79 | 217 | 0.53 | 0.61 | 0.74 | 0.81 | 0.85 | 0.94 | 0.98 | 1.05 | 1.15 | 1.28 | 1.41 |
| 80-84 | 236 | 0.46 | 0.54 | 0.65 | 0.70 | 0.77 | 0.81 | 0.87 | 0.93 | 1.00 | 1.12 | 1.26 |
| 85-89 | 169 | 0.30 | 0.35 | 0.45 | 0.55 | 0.60 | 0.66 | 0.77 | 0.82 | 0.91 | 1.05 | 1.16 |
| 90-94 | 52 | 0.32 | 0.36 | 0.41 | 0.45 | 0.52 | 0.56 | 0.58 | 0.65 | 0.74 | 0.89 | 1.01 |
| 95-99 | n/a | n/a | n/a | n/a | n/a | n/a | n/a | n/a | n/a | n/a | n/a | n/a |
| 100+ | n/a | n/a | n/a | n/a | n/a | n/a | n/a | n/a | n/a | n/a | n/a | n/a |
| NSJE = National Survey of the Japanese Elderly | | | | | | | | | | | | |

**Supplementary file 49.** Reference values for 2.5 m gait speed for males in Japan (NSJE; pooled *n* = 927)

| **Age (years)** | ***n*** | **Percentile (m/s)** | | | | | | | | | | |
| --- | --- | --- | --- | --- | --- | --- | --- | --- | --- | --- | --- | --- |
|  |  | **5^th^** | **10^th^** | **20^th^** | **30^th^** | **40^th^** | **50^th^** | **60^th^** | **70^th^** | **80^th^** | **90^th^** | **95^th^** |
| 20-24 | n/a | n/a | n/a | n/a | n/a | n/a | n/a | n/a | n/a | n/a | n/a | n/a |
| 25-29 | n/a | n/a | n/a | n/a | n/a | n/a | n/a | n/a | n/a | n/a | n/a | n/a |
| 30-34 | n/a | n/a | n/a | n/a | n/a | n/a | n/a | n/a | n/a | n/a | n/a | n/a |
| 35-39 | n/a | n/a | n/a | n/a | n/a | n/a | n/a | n/a | n/a | n/a | n/a | n/a |
| 40-44 | n/a | n/a | n/a | n/a | n/a | n/a | n/a | n/a | n/a | n/a | n/a | n/a |
| 45-49 | n/a | n/a | n/a | n/a | n/a | n/a | n/a | n/a | n/a | n/a | n/a | n/a |
| 50-54 | n/a | n/a | n/a | n/a | n/a | n/a | n/a | n/a | n/a | n/a | n/a | n/a |
| 55-59 | n/a | n/a | n/a | n/a | n/a | n/a | n/a | n/a | n/a | n/a | n/a | n/a |
| 60-64 | 125 | 0.75 | 0.82 | 0.87 | 0.98 | 1.03 | 1.11 | 1.16 | 1.21 | 1.26 | 1.36 | 1.44 |
| 65-69 | 138 | 0.71 | 0.75 | 0.84 | 0.97 | 1.02 | 1.09 | 1.16 | 1.20 | 1.26 | 1.46 | 1.61 |
| 70-74 | 106 | 0.65 | 0.80 | 0.88 | 0.91 | 1.00 | 1.05 | 1.11 | 1.17 | 1.22 | 1.39 | 1.47 |
| 75-79 | 226 | 0.55 | 0.67 | 0.74 | 0.83 | 0.88 | 0.97 | 1.02 | 1.08 | 1.15 | 1.27 | 1.37 |
| 80-84 | 222 | 0.46 | 0.55 | 0.70 | 0.76 | 0.82 | 0.87 | 0.93 | 1.00 | 1.05 | 1.20 | 1.35 |
| 85-89 | 110 | 0.29 | 0.39 | 0.57 | 0.66 | 0.71 | 0.81 | 0.84 | 0.96 | 1.04 | 1.20 | 1.39 |
| 90-94 | n/a | n/a | n/a | n/a | n/a | n/a | n/a | n/a | n/a | n/a | n/a | n/a |
| 95-99 | n/a | n/a | n/a | n/a | n/a | n/a | n/a | n/a | n/a | n/a | n/a | n/a |
| 100+ | n/a | n/a | n/a | n/a | n/a | n/a | n/a | n/a | n/a | n/a | n/a | n/a |
| NSJE = National Survey of the Japanese Elderly | | | | | | | | | | | | |

**Supplementary file 50.** Reference values for 2.5 m gait speed for females in Singapore (PHASE; pooled *n* = 651)

| **Age (years)** | ***n*** | **Percentile (m/s)** | | | | | | | | | | |
| --- | --- | --- | --- | --- | --- | --- | --- | --- | --- | --- | --- | --- |
|  |  | **5^th^** | **10^th^** | **20^th^** | **30^th^** | **40^th^** | **50^th^** | **60^th^** | **70^th^** | **80^th^** | **90^th^** | **95^th^** |
| 20-24 | n/a | n/a | n/a | n/a | n/a | n/a | n/a | n/a | n/a | n/a | n/a | n/a |
| 25-29 | n/a | n/a | n/a | n/a | n/a | n/a | n/a | n/a | n/a | n/a | n/a | n/a |
| 30-34 | n/a | n/a | n/a | n/a | n/a | n/a | n/a | n/a | n/a | n/a | n/a | n/a |
| 35-39 | n/a | n/a | n/a | n/a | n/a | n/a | n/a | n/a | n/a | n/a | n/a | n/a |
| 40-44 | n/a | n/a | n/a | n/a | n/a | n/a | n/a | n/a | n/a | n/a | n/a | n/a |
| 45-49 | n/a | n/a | n/a | n/a | n/a | n/a | n/a | n/a | n/a | n/a | n/a | n/a |
| 50-54 | n/a | n/a | n/a | n/a | n/a | n/a | n/a | n/a | n/a | n/a | n/a | n/a |
| 55-59 | n/a | n/a | n/a | n/a | n/a | n/a | n/a | n/a | n/a | n/a | n/a | n/a |
| 60-64 | n/a | n/a | n/a | n/a | n/a | n/a | n/a | n/a | n/a | n/a | n/a | n/a |
| 65-69 | 150 | 0.23 | 0.31 | 0.41 | 0.49 | 0.53 | 0.61 | 0.72 | 0.82 | 0.92 | 1.04 | 1.11 |
| 70-74 | 184 | 0.22 | 0.28 | 0.37 | 0.45 | 0.53 | 0.58 | 0.66 | 0.73 | 0.82 | 0.92 | 1.00 |
| 75-79 | 137 | 0.22 | 0.26 | 0.32 | 0.40 | 0.46 | 0.51 | 0.60 | 0.68 | 0.78 | 0.85 | 0.98 |
| 80-84 | 103 | 0.17 | 0.25 | 0.31 | 0.36 | 0.41 | 0.45 | 0.49 | 0.57 | 0.67 | 0.76 | 0.91 |
| 85-89 | 57 | 0.11 | 0.13 | 0.20 | 0.26 | 0.31 | 0.36 | 0.42 | 0.49 | 0.55 | 0.66 | 0.83 |
| 90-94 | 20 | 0.16 | 0.21 | 0.27 | 0.31 | 0.35 | 0.37 | 0.40 | 0.54 | 0.66 | 0.72 | 0.89 |
| 95-99 | n/a | n/a | n/a | n/a | n/a | n/a | n/a | n/a | n/a | n/a | n/a | n/a |
| 100+ | n/a | n/a | n/a | n/a | n/a | n/a | n/a | n/a | n/a | n/a | n/a | n/a |
| PHASE = Panel on Health and Ageing of Singaporean Elderly | | | | | | | | | | | | |

**Supplementary file 51.** Reference values for 2.5 m gait speed for males in Singapore (PHASE; pooled *n* = 492)

| **Age (years)** | ***n*** | **Percentile (m/s)** | | | | | | | | | | |
| --- | --- | --- | --- | --- | --- | --- | --- | --- | --- | --- | --- | --- |
|  |  | **5^th^** | **10^th^** | **20^th^** | **30^th^** | **40^th^** | **50^th^** | **60^th^** | **70^th^** | **80^th^** | **90^th^** | **95^th^** |
| 20-24 | n/a | n/a | n/a | n/a | n/a | n/a | n/a | n/a | n/a | n/a | n/a | n/a |
| 25-29 | n/a | n/a | n/a | n/a | n/a | n/a | n/a | n/a | n/a | n/a | n/a | n/a |
| 30-34 | n/a | n/a | n/a | n/a | n/a | n/a | n/a | n/a | n/a | n/a | n/a | n/a |
| 35-39 | n/a | n/a | n/a | n/a | n/a | n/a | n/a | n/a | n/a | n/a | n/a | n/a |
| 40-44 | n/a | n/a | n/a | n/a | n/a | n/a | n/a | n/a | n/a | n/a | n/a | n/a |
| 45-49 | n/a | n/a | n/a | n/a | n/a | n/a | n/a | n/a | n/a | n/a | n/a | n/a |
| 50-54 | n/a | n/a | n/a | n/a | n/a | n/a | n/a | n/a | n/a | n/a | n/a | n/a |
| 55-59 | n/a | n/a | n/a | n/a | n/a | n/a | n/a | n/a | n/a | n/a | n/a | n/a |
| 60-64 | n/a | n/a | n/a | n/a | n/a | n/a | n/a | n/a | n/a | n/a | n/a | n/a |
| 65-69 | 114 | 0.21 | 0.31 | 0.42 | 0.50 | 0.63 | 0.71 | 0.81 | 0.90 | 1.00 | 1.15 | 1.25 |
| 70-74 | 139 | 0.32 | 0.36 | 0.45 | 0.53 | 0.58 | 0.65 | 0.69 | 0.79 | 0.90 | 1.04 | 1.18 |
| 75-79 | 106 | 0.30 | 0.33 | 0.39 | 0.45 | 0.50 | 0.60 | 0.65 | 0.71 | 0.82 | 0.98 | 1.11 |
| 80-84 | 84 | 0.19 | 0.23 | 0.31 | 0.35 | 0.42 | 0.49 | 0.55 | 0.63 | 0.69 | 0.81 | 0.85 |
| 85-89 | 49 | 0.17 | 0.19 | 0.27 | 0.31 | 0.34 | 0.43 | 0.50 | 0.62 | 0.70 | 0.78 | 0.82 |
| 90-94 | n/a | n/a | n/a | n/a | n/a | n/a | n/a | n/a | n/a | n/a | n/a | n/a |
| 95-99 | n/a | n/a | n/a | n/a | n/a | n/a | n/a | n/a | n/a | n/a | n/a | n/a |
| 100+ | n/a | n/a | n/a | n/a | n/a | n/a | n/a | n/a | n/a | n/a | n/a | n/a |
| PHASE = Panel on Health and Ageing of Singaporean Elderly | | | | | | | | | | | | |

**Supplementary file 52.** Percentile curves for 4 m gait speed for females in Asia (LASI, PIONEER, SAGE, SLHAS; pooled *n* = 76,443)


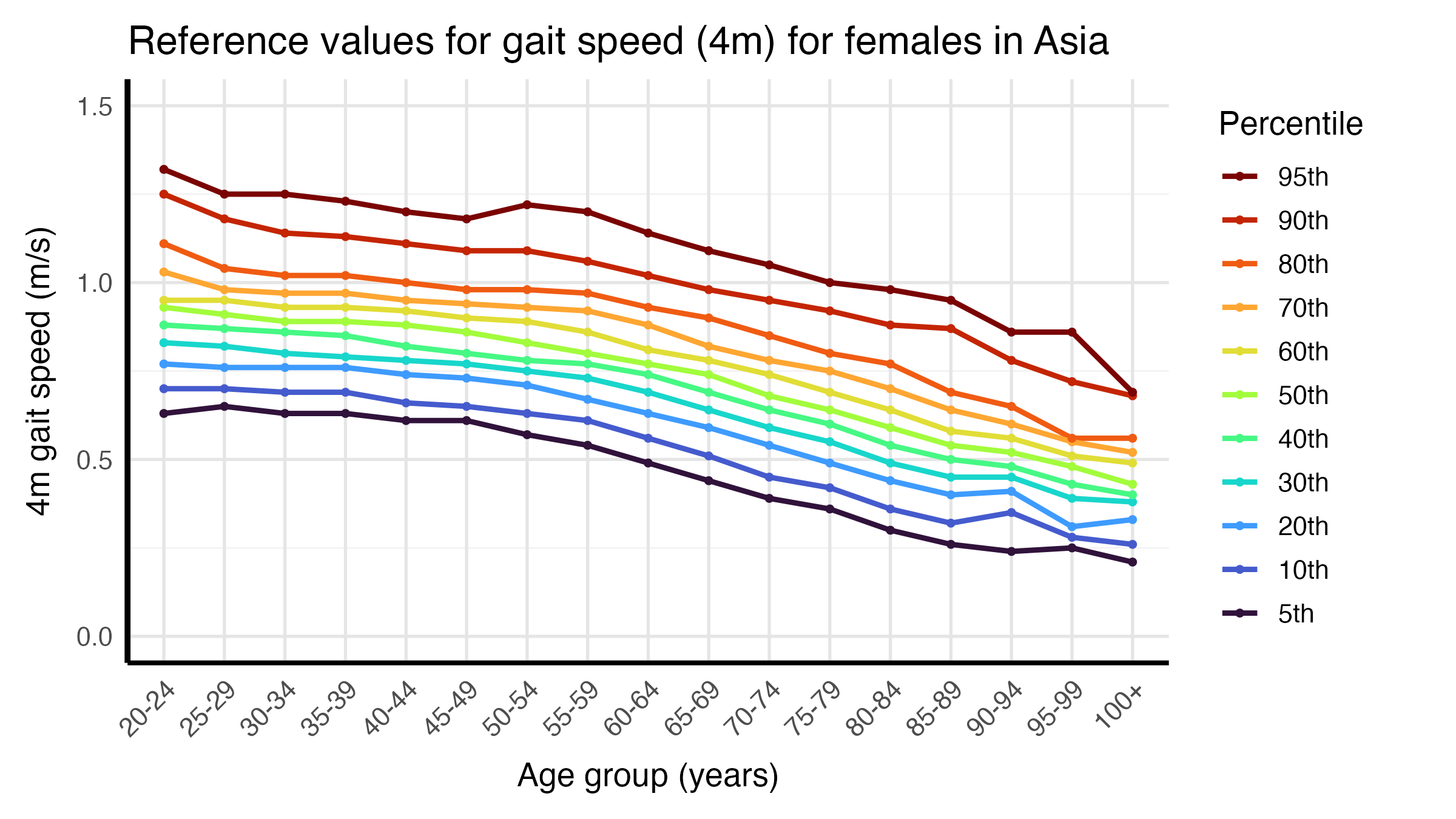


**Supplementary file 53.** Percentile curves for 4 m gait speed for males in Asia (LASI, PIONEER, SAGE, SLHAS; pooled *n* = 62,678)


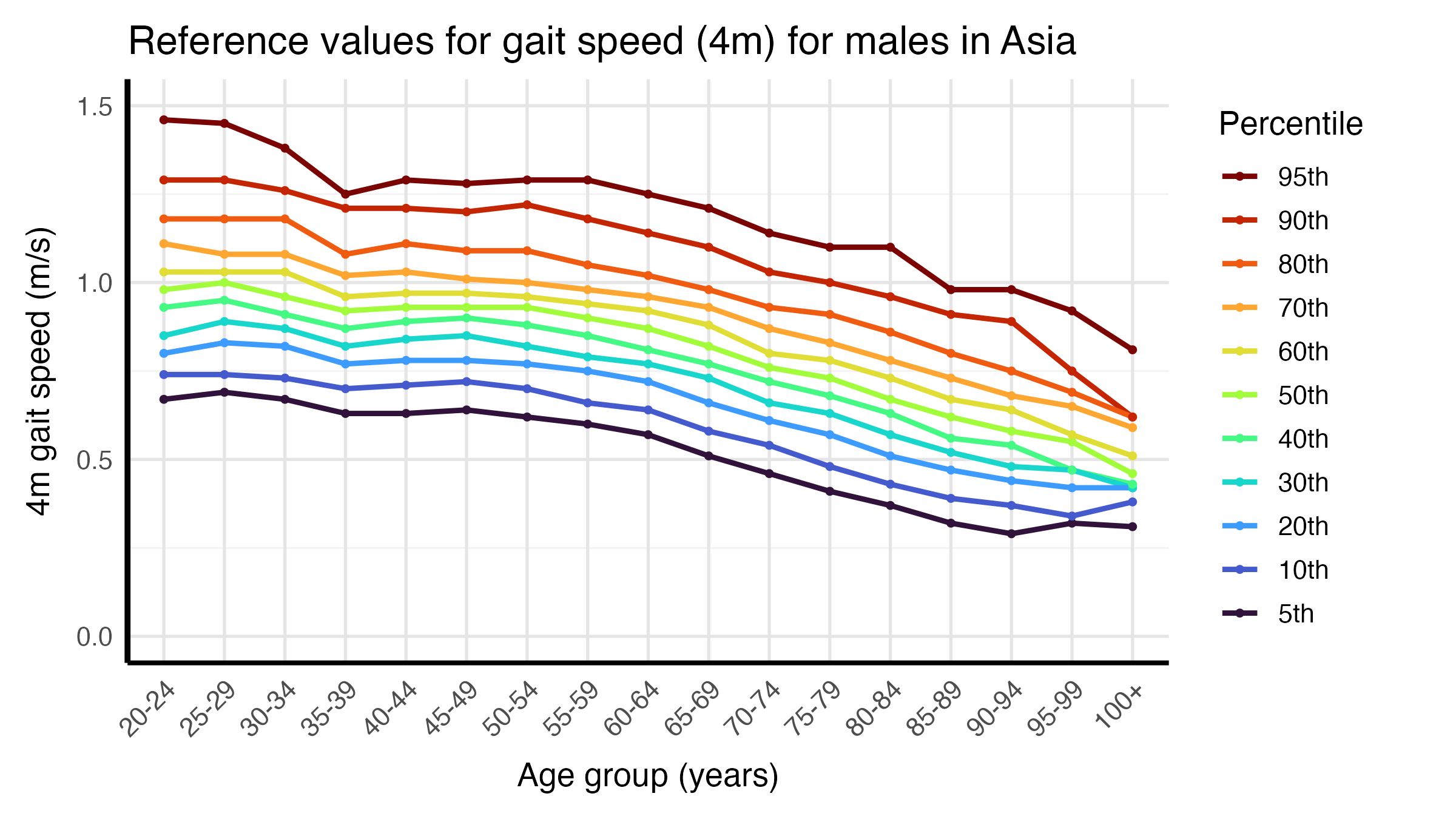


**Supplementary file 54.** Reference values for 4 m gait speed for females in South Asia (LASI, SAGE, SLHAS; pooled *n* = 70,377)

| **Age (years)** | ***n*** | **Percentile (m/s)** | | | | | | | | | | |
| --- | --- | --- | --- | --- | --- | --- | --- | --- | --- | --- | --- | --- |
|  |  | **5^th^** | **10^th^** | **20^th^** | **30^th^** | **40^th^** | **50^th^** | **60^th^** | **70^th^** | **80^th^** | **90^th^** | **95^th^** |
| 20-24 | 721 | 0.62 | 0.70 | 0.77 | 0.82 | 0.87 | 0.93 | 0.95 | 1.00 | 1.08 | 1.20 | 1.29 |
| 25-29 | 900 | 0.65 | 0.69 | 0.75 | 0.81 | 0.86 | 0.89 | 0.93 | 0.98 | 1.01 | 1.14 | 1.24 |
| 30-34 | 1,465 | 0.62 | 0.68 | 0.75 | 0.80 | 0.85 | 0.89 | 0.93 | 0.96 | 1.00 | 1.12 | 1.22 |
| 35-39 | 3,954 | 0.63 | 0.69 | 0.75 | 0.79 | 0.85 | 0.89 | 0.93 | 0.97 | 1.01 | 1.12 | 1.22 |
| 40-44 | 8,360 | 0.61 | 0.66 | 0.74 | 0.78 | 0.82 | 0.88 | 0.92 | 0.95 | 0.99 | 1.11 | 1.19 |
| 45-49 | 12,093 | 0.60 | 0.65 | 0.73 | 0.77 | 0.80 | 0.86 | 0.90 | 0.94 | 0.98 | 1.08 | 1.17 |
| 50-54 | 10,175 | 0.57 | 0.63 | 0.70 | 0.74 | 0.78 | 0.82 | 0.87 | 0.92 | 0.95 | 1.04 | 1.15 |
| 55-59 | 9,437 | 0.54 | 0.60 | 0.66 | 0.72 | 0.76 | 0.78 | 0.83 | 0.89 | 0.94 | 1.00 | 1.10 |
| 60-64 | 8,843 | 0.49 | 0.55 | 0.63 | 0.67 | 0.72 | 0.76 | 0.78 | 0.84 | 0.90 | 0.97 | 1.04 |
| 65-69 | 6,728 | 0.43 | 0.51 | 0.58 | 0.63 | 0.68 | 0.72 | 0.76 | 0.80 | 0.87 | 0.95 | 1.00 |
| 70-74 | 3,894 | 0.38 | 0.44 | 0.52 | 0.57 | 0.62 | 0.66 | 0.71 | 0.76 | 0.80 | 0.90 | 0.97 |
| 75-79 | 2,124 | 0.34 | 0.40 | 0.47 | 0.52 | 0.57 | 0.62 | 0.65 | 0.70 | 0.75 | 0.83 | 0.91 |
| 80-84 | 1,046 | 0.29 | 0.35 | 0.42 | 0.47 | 0.52 | 0.55 | 0.61 | 0.65 | 0.71 | 0.78 | 0.87 |
| 85-89 | 419 | 0.25 | 0.31 | 0.38 | 0.44 | 0.49 | 0.53 | 0.56 | 0.61 | 0.66 | 0.75 | 0.90 |
| 90-94 | 154 | 0.24 | 0.35 | 0.40 | 0.44 | 0.48 | 0.52 | 0.55 | 0.59 | 0.64 | 0.73 | 0.82 |
| 95-99 | 64 | 0.25 | 0.28 | 0.31 | 0.37 | 0.41 | 0.47 | 0.50 | 0.54 | 0.56 | 0.68 | 0.75 |
| 100+ | n/a | n/a | n/a | n/a | n/a | n/a | n/a | n/a | n/a | n/a | n/a | n/a |
| LASI = Longitudinal Ageing Study in India; SAGE = Study on Global AGEing and Adult Health; SLHAS = Sri Lanka Health and Ageing Study | | | | | | | | | | | | |

**Supplementary file 55.** Reference values for 4 m gait speed for males in South Asia (LASI, SAGE, SLHAS; pooled *n* = 57,375)

| **Age (years)** | ***n*** | **Percentile (m/s)** | | | | | | | | | | |
| --- | --- | --- | --- | --- | --- | --- | --- | --- | --- | --- | --- | --- |
|  |  | **5^th^** | **10^th^** | **20^th^** | **30^th^** | **40^th^** | **50^th^** | **60^th^** | **70^th^** | **80^th^** | **90^th^** | **95^th^** |
| 20-24 | 299 | 0.66 | 0.73 | 0.78 | 0.85 | 0.89 | 0.95 | 1.00 | 1.05 | 1.14 | 1.25 | 1.29 |
| 25-29 | 301 | 0.67 | 0.74 | 0.81 | 0.87 | 0.93 | 0.97 | 1.02 | 1.05 | 1.11 | 1.25 | 1.33 |
| 30-34 | 327 | 0.63 | 0.71 | 0.81 | 0.86 | 0.89 | 0.93 | 0.98 | 1.03 | 1.10 | 1.25 | 1.29 |
| 35-39 | 613 | 0.62 | 0.69 | 0.76 | 0.81 | 0.86 | 0.90 | 0.95 | 1.00 | 1.07 | 1.18 | 1.25 |
| 40-44 | 607 | 0.62 | 0.70 | 0.77 | 0.83 | 0.88 | 0.93 | 0.95 | 1.00 | 1.07 | 1.18 | 1.25 |
| 45-49 | 10,621 | 0.64 | 0.72 | 0.78 | 0.85 | 0.89 | 0.93 | 0.97 | 1.01 | 1.09 | 1.20 | 1.27 |
| 50-54 | 9,475 | 0.62 | 0.70 | 0.77 | 0.82 | 0.87 | 0.92 | 0.95 | 0.99 | 1.06 | 1.18 | 1.25 |
| 55-59 | 8,495 | 0.59 | 0.65 | 0.74 | 0.78 | 0.84 | 0.89 | 0.93 | 0.97 | 1.03 | 1.15 | 1.24 |
| 60-64 | 8,669 | 0.56 | 0.63 | 0.71 | 0.76 | 0.80 | 0.85 | 0.90 | 0.95 | 0.99 | 1.11 | 1.19 |
| 65-69 | 7,867 | 0.51 | 0.58 | 0.65 | 0.72 | 0.76 | 0.80 | 0.87 | 0.92 | 0.96 | 1.05 | 1.16 |
| 70-74 | 5,083 | 0.46 | 0.53 | 0.61 | 0.65 | 0.71 | 0.75 | 0.78 | 0.85 | 0.92 | 0.99 | 1.08 |
| 75-79 | 2,843 | 0.41 | 0.48 | 0.56 | 0.62 | 0.66 | 0.71 | 0.76 | 0.80 | 0.87 | 0.95 | 1.03 |
| 80-84 | 1,438 | 0.36 | 0.43 | 0.50 | 0.56 | 0.61 | 0.64 | 0.70 | 0.75 | 0.82 | 0.92 | 1.01 |
| 85-89 | 548 | 0.29 | 0.38 | 0.46 | 0.51 | 0.55 | 0.60 | 0.65 | 0.71 | 0.77 | 0.88 | 0.93 |
| 90-94 | 189 | 0.29 | 0.36 | 0.44 | 0.48 | 0.53 | 0.56 | 0.63 | 0.66 | 0.72 | 0.88 | 0.97 |
| 95-99 | n/a | n/a | n/a | n/a | n/a | n/a | n/a | n/a | n/a | n/a | n/a | n/a |
| 100+ | n/a | n/a | n/a | n/a | n/a | n/a | n/a | n/a | n/a | n/a | n/a | n/a |
| LASI = Longitudinal Ageing Study in India; SAGE = Study on Global AGEing and Adult Health; SLHAS = Sri Lanka Health and Ageing Study | | | | | | | | | | | | |

**Supplementary file 56.** Reference values for 4 m gait speed for females in China (SAGE; pooled *n* = 4,660)

| **Age (years)** | ***n*** | **Percentile (m/s)** | | | | | | | | | | |
| --- | --- | --- | --- | --- | --- | --- | --- | --- | --- | --- | --- | --- |
|  |  | **5^th^** | **10^th^** | **20^th^** | **30^th^** | **40^th^** | **50^th^** | **60^th^** | **70^th^** | **80^th^** | **90^th^** | **95^th^** |
| 20-24 | 45 | 0.81 | 0.88 | 0.95 | 1.05 | 1.10 | 1.18 | 1.25 | 1.33 | 1.38 | 1.81 | 1.98 |
| 25-29 | 51 | 0.84 | 0.95 | 1.00 | 1.00 | 1.11 | 1.14 | 1.25 | 1.29 | 1.38 | 1.48 | 1.51 |
| 30-34 | 48 | 0.85 | 0.89 | 0.98 | 1.00 | 1.00 | 1.04 | 1.16 | 1.25 | 1.27 | 1.41 | 1.62 |
| 35-39 | 73 | 0.82 | 0.91 | 0.95 | 1.00 | 1.05 | 1.14 | 1.18 | 1.29 | 1.29 | 1.33 | 1.43 |
| 40-44 | 83 | 0.80 | 0.84 | 0.93 | 1.02 | 1.03 | 1.11 | 1.14 | 1.25 | 1.32 | 1.38 | 1.59 |
| 45-49 | 138 | 0.80 | 0.82 | 0.96 | 1.00 | 1.05 | 1.11 | 1.18 | 1.25 | 1.29 | 1.33 | 1.43 |
| 50-54 | 957 | 0.75 | 0.80 | 0.89 | 0.95 | 1.00 | 1.03 | 1.11 | 1.21 | 1.33 | 1.33 | 1.48 |
| 55-59 | 1040 | 0.69 | 0.80 | 0.87 | 0.95 | 1.00 | 1.03 | 1.08 | 1.18 | 1.29 | 1.33 | 1.43 |
| 60-64 | 729 | 0.67 | 0.77 | 0.82 | 0.91 | 0.95 | 1.00 | 1.05 | 1.14 | 1.25 | 1.33 | 1.33 |
| 65-69 | 438 | 0.62 | 0.70 | 0.80 | 0.85 | 0.93 | 0.99 | 1.00 | 1.08 | 1.18 | 1.33 | 1.33 |
| 70-74 | 389 | 0.56 | 0.59 | 0.68 | 0.77 | 0.83 | 0.91 | 0.95 | 1.00 | 1.08 | 1.25 | 1.33 |
| 75-79 | 399 | 0.45 | 0.53 | 0.62 | 0.69 | 0.77 | 0.80 | 0.87 | 0.95 | 1.00 | 1.14 | 1.29 |
| 80-84 | 204 | 0.40 | 0.42 | 0.51 | 0.58 | 0.65 | 0.73 | 0.80 | 0.85 | 0.98 | 1.03 | 1.11 |
| 85-89 | 66 | 0.40 | 0.40 | 0.46 | 0.57 | 0.66 | 0.69 | 0.80 | 0.89 | 0.98 | 1.01 | 1.10 |
| 90-94 | n/a | n/a | n/a | n/a | n/a | n/a | n/a | n/a | n/a | n/a | n/a | n/a |
| 95-99 | n/a | n/a | n/a | n/a | n/a | n/a | n/a | n/a | n/a | n/a | n/a | n/a |
| 100+ | n/a | n/a | n/a | n/a | n/a | n/a | n/a | n/a | n/a | n/a | n/a | n/a |
| SAGE = Study on Global AGEing and Adult Health | | | | | | | | | | | | |

**Supplementary file 57.** Reference values for 4 m gait speed for males in China (SAGE; pooled *n* = 4,055)

| **Age (years)** | ***n*** | **Percentile (m/s)** | | | | | | | | | | |
| --- | --- | --- | --- | --- | --- | --- | --- | --- | --- | --- | --- | --- |
|  |  | **5^th^** | **10^th^** | **20^th^** | **30^th^** | **40^th^** | **50^th^** | **60^th^** | **70^th^** | **80^th^** | **90^th^** | **95^th^** |
| 20-24 | 38 | 0.94 | 1.00 | 1.03 | 1.06 | 1.14 | 1.25 | 1.30 | 1.42 | 1.64 | 1.76 | 1.83 |
| 25-29 | 53 | 0.82 | 0.84 | 1.00 | 1.05 | 1.11 | 1.21 | 1.25 | 1.31 | 1.33 | 1.52 | 1.81 |
| 30-34 | 56 | 0.87 | 0.94 | 1.05 | 1.08 | 1.14 | 1.16 | 1.25 | 1.29 | 1.38 | 1.54 | 1.67 |
| 35-39 | 46 | 0.87 | 0.91 | 0.95 | 1.00 | 1.03 | 1.07 | 1.21 | 1.25 | 1.33 | 1.51 | 1.72 |
| 40-44 | 82 | 0.74 | 0.80 | 0.95 | 1.00 | 1.04 | 1.08 | 1.16 | 1.21 | 1.33 | 1.42 | 1.48 |
| 45-49 | 113 | 0.80 | 0.84 | 0.95 | 1.00 | 1.03 | 1.05 | 1.14 | 1.25 | 1.33 | 1.43 | 1.67 |
| 50-54 | 746 | 0.72 | 0.82 | 0.95 | 1.00 | 1.05 | 1.14 | 1.25 | 1.29 | 1.33 | 1.38 | 1.54 |
| 55-59 | 822 | 0.73 | 0.80 | 0.89 | 0.98 | 1.00 | 1.05 | 1.14 | 1.25 | 1.33 | 1.38 | 1.54 |
| 60-64 | 716 | 0.67 | 0.80 | 0.87 | 0.95 | 1.00 | 1.05 | 1.14 | 1.21 | 1.29 | 1.33 | 1.48 |
| 65-69 | 463 | 0.65 | 0.77 | 0.83 | 0.93 | 1.00 | 1.03 | 1.08 | 1.18 | 1.29 | 1.33 | 1.48 |
| 70-74 | 366 | 0.57 | 0.67 | 0.77 | 0.82 | 0.89 | 0.93 | 1.00 | 1.08 | 1.18 | 1.33 | 1.37 |
| 75-79 | 334 | 0.54 | 0.61 | 0.73 | 0.80 | 0.87 | 0.93 | 0.98 | 1.00 | 1.08 | 1.25 | 1.33 |
| 80-84 | 159 | 0.45 | 0.51 | 0.62 | 0.68 | 0.74 | 0.80 | 0.83 | 0.98 | 1.03 | 1.29 | 1.33 |
| 85-89 | 61 | 0.40 | 0.41 | 0.50 | 0.58 | 0.67 | 0.71 | 0.80 | 0.85 | 0.93 | 1.00 | 1.05 |
| 90-94 | n/a | n/a | n/a | n/a | n/a | n/a | n/a | n/a | n/a | n/a | n/a | n/a |
| 95-99 | n/a | n/a | n/a | n/a | n/a | n/a | n/a | n/a | n/a | n/a | n/a | n/a |
| 100+ | n/a | n/a | n/a | n/a | n/a | n/a | n/a | n/a | n/a | n/a | n/a | n/a |
| SAGE = Study on Global AGEing and Adult Health | | | | | | | | | | | | |

**Supplementary file 58.** Reference values for 4 m gait speed for females in India (LASI, SAGE; pooled *n* = 67,181)

| **Age (years)** | ***n*** | **Percentile (m/s)** | | | | | | | | | | |
| --- | --- | --- | --- | --- | --- | --- | --- | --- | --- | --- | --- | --- |
|  |  | **5^th^** | **10^th^** | **20^th^** | **30^th^** | **40^th^** | **50^th^** | **60^th^** | **70^th^** | **80^th^** | **90^th^** | **95^th^** |
| 20-24 | 533 | 0.68 | 0.74 | 0.80 | 0.87 | 0.93 | 0.95 | 0.98 | 1.03 | 1.14 | 1.25 | 1.29 |
| 25-29 | 644 | 0.67 | 0.71 | 0.77 | 0.85 | 0.89 | 0.93 | 0.95 | 0.99 | 1.05 | 1.18 | 1.25 |
| 30-34 | 1,261 | 0.64 | 0.71 | 0.77 | 0.82 | 0.87 | 0.90 | 0.95 | 0.97 | 1.03 | 1.14 | 1.25 |
| 35-39 | 3,601 | 0.65 | 0.71 | 0.77 | 0.80 | 0.86 | 0.90 | 0.94 | 0.97 | 1.02 | 1.13 | 1.23 |
| 40-44 | 8,072 | 0.62 | 0.67 | 0.74 | 0.78 | 0.82 | 0.88 | 0.92 | 0.95 | 1.00 | 1.11 | 1.19 |
| 45-49 | 11,805 | 0.61 | 0.66 | 0.73 | 0.77 | 0.80 | 0.86 | 0.90 | 0.94 | 0.98 | 1.08 | 1.18 |
| 50-54 | 9,910 | 0.57 | 0.63 | 0.70 | 0.75 | 0.78 | 0.82 | 0.87 | 0.92 | 0.96 | 1.04 | 1.15 |
| 55-59 | 9,147 | 0.54 | 0.61 | 0.67 | 0.73 | 0.76 | 0.79 | 0.84 | 0.89 | 0.94 | 1.00 | 1.11 |
| 60-64 | 8,561 | 0.49 | 0.56 | 0.63 | 0.68 | 0.73 | 0.76 | 0.79 | 0.85 | 0.91 | 0.97 | 1.04 |
| 65-69 | 6,453 | 0.44 | 0.51 | 0.59 | 0.64 | 0.68 | 0.73 | 0.76 | 0.80 | 0.87 | 0.95 | 1.00 |
| 70-74 | 3,600 | 0.39 | 0.45 | 0.53 | 0.58 | 0.63 | 0.66 | 0.72 | 0.76 | 0.80 | 0.91 | 0.97 |
| 75-79 | 1,980 | 0.35 | 0.41 | 0.48 | 0.53 | 0.58 | 0.62 | 0.65 | 0.71 | 0.76 | 0.84 | 0.92 |
| 80-84 | 981 | 0.29 | 0.36 | 0.43 | 0.48 | 0.53 | 0.56 | 0.61 | 0.65 | 0.72 | 0.78 | 0.88 |
| 85-89 | 400 | 0.25 | 0.31 | 0.39 | 0.44 | 0.49 | 0.54 | 0.56 | 0.62 | 0.66 | 0.77 | 0.91 |
| 90-94 | 149 | 0.25 | 0.36 | 0.41 | 0.44 | 0.48 | 0.53 | 0.56 | 0.60 | 0.64 | 0.74 | 0.82 |
| 95-99 | 63 | 0.25 | 0.28 | 0.31 | 0.38 | 0.42 | 0.47 | 0.50 | 0.54 | 0.56 | 0.68 | 0.75 |
| 100+ | 21 | 0.20 | 0.25 | 0.32 | 0.38 | 0.39 | 0.43 | 0.49 | 0.51 | 0.53 | 0.65 | 0.68 |
| LASI = Longitudinal Ageing Study in India; SAGE = Study on Global AGEing and Adult Health | | | | | | | | | | | | |

**Supplementary file 59.** Reference values for 4 m gait speed for males in India (LASI, SAGE; pooled *n* = 54,358)

| **Age (years)** | ***n*** | **Percentile (m/s)** | | | | | | | | | | |
| --- | --- | --- | --- | --- | --- | --- | --- | --- | --- | --- | --- | --- |
|  |  | **5^th^** | **10^th^** | **20^th^** | **30^th^** | **40^th^** | **50^th^** | **60^th^** | **70^th^** | **80^th^** | **90^th^** | **95^th^** |
| 20-24 | 127 | 0.75 | 0.84 | 0.91 | 0.95 | 1.01 | 1.05 | 1.11 | 1.18 | 1.25 | 1.29 | 1.41 |
| 25-29 | 126 | 0.77 | 0.86 | 0.93 | 0.95 | 1.03 | 1.05 | 1.08 | 1.18 | 1.25 | 1.36 | 1.58 |
| 30-34 | 139 | 0.70 | 0.80 | 0.87 | 0.93 | 0.98 | 1.03 | 1.08 | 1.16 | 1.25 | 1.29 | 1.38 |
| 35-39 | 247 | 0.66 | 0.75 | 0.83 | 0.89 | 0.95 | 1.00 | 1.05 | 1.11 | 1.18 | 1.25 | 1.29 |
| 40-44 | 318 | 0.70 | 0.75 | 0.83 | 0.89 | 0.93 | 0.95 | 0.99 | 1.05 | 1.12 | 1.25 | 1.29 |
| 45-49 | 10,327 | 0.64 | 0.72 | 0.78 | 0.85 | 0.90 | 0.93 | 0.97 | 1.01 | 1.09 | 1.20 | 1.27 |
| 50-54 | 9,203 | 0.63 | 0.70 | 0.77 | 0.82 | 0.88 | 0.92 | 0.95 | 0.99 | 1.07 | 1.18 | 1.25 |
| 55-59 | 8,194 | 0.60 | 0.66 | 0.74 | 0.78 | 0.84 | 0.89 | 0.93 | 0.97 | 1.03 | 1.15 | 1.24 |
| 60-64 | 8,367 | 0.57 | 0.64 | 0.71 | 0.76 | 0.80 | 0.85 | 0.91 | 0.95 | 1.00 | 1.11 | 1.19 |
| 65-69 | 7,609 | 0.51 | 0.58 | 0.66 | 0.73 | 0.77 | 0.81 | 0.87 | 0.92 | 0.96 | 1.05 | 1.17 |
| 70-74 | 4,841 | 0.46 | 0.53 | 0.61 | 0.66 | 0.71 | 0.75 | 0.79 | 0.86 | 0.92 | 0.99 | 1.08 |
| 75-79 | 2,693 | 0.41 | 0.48 | 0.56 | 0.63 | 0.66 | 0.72 | 0.76 | 0.80 | 0.87 | 0.96 | 1.03 |
| 80-84 | 1,373 | 0.37 | 0.43 | 0.50 | 0.56 | 0.62 | 0.65 | 0.71 | 0.76 | 0.82 | 0.93 | 1.02 |
| 85-89 | 531 | 0.29 | 0.39 | 0.47 | 0.51 | 0.55 | 0.61 | 0.65 | 0.71 | 0.77 | 0.89 | 0.93 |
| 90-94 | 188 | 0.29 | 0.36 | 0.44 | 0.48 | 0.53 | 0.56 | 0.63 | 0.66 | 0.72 | 0.88 | 0.97 |
| 95-99 | 54 | 0.31 | 0.34 | 0.42 | 0.47 | 0.47 | 0.55 | 0.56 | 0.63 | 0.68 | 0.73 | 0.77 |
| 100+ | 21 | 0.31 | 0.37 | 0.42 | 0.42 | 0.44 | 0.46 | 0.54 | 0.61 | 0.62 | 0.62 | 0.82 |
| LASI = Longitudinal Ageing Study in India; SAGE = Study on Global AGEing and Adult Health | | | | | | | | | | | | |

**Supplementary file 60.** Reference values for 4 m gait speed for females in Singapore (PIONEER; pooled *n* = 1,354)

| **Age (years)** | ***n*** | **Percentile (m/s)** | | | | | | | | | | |
| --- | --- | --- | --- | --- | --- | --- | --- | --- | --- | --- | --- | --- |
|  |  | **5^th^** | **10^th^** | **20^th^** | **30^th^** | **40^th^** | **50^th^** | **60^th^** | **70^th^** | **80^th^** | **90^th^** | **95^th^** |
| 20-24 | n/a | n/a | n/a | n/a | n/a | n/a | n/a | n/a | n/a | n/a | n/a | n/a |
| 25-29 | n/a | n/a | n/a | n/a | n/a | n/a | n/a | n/a | n/a | n/a | n/a | n/a |
| 30-34 | n/a | n/a | n/a | n/a | n/a | n/a | n/a | n/a | n/a | n/a | n/a | n/a |
| 35-39 | n/a | n/a | n/a | n/a | n/a | n/a | n/a | n/a | n/a | n/a | n/a | n/a |
| 40-44 | n/a | n/a | n/a | n/a | n/a | n/a | n/a | n/a | n/a | n/a | n/a | n/a |
| 45-49 | n/a | n/a | n/a | n/a | n/a | n/a | n/a | n/a | n/a | n/a | n/a | n/a |
| 50-54 | n/a | n/a | n/a | n/a | n/a | n/a | n/a | n/a | n/a | n/a | n/a | n/a |
| 55-59 | n/a | n/a | n/a | n/a | n/a | n/a | n/a | n/a | n/a | n/a | n/a | n/a |
| 60-64 | 242 | 0.65 | 0.72 | 0.84 | 0.89 | 0.93 | 0.99 | 1.03 | 1.08 | 1.14 | 1.24 | 1.29 |
| 65-69 | 260 | 0.54 | 0.65 | 0.75 | 0.82 | 0.87 | 0.91 | 0.95 | 1.00 | 1.07 | 1.14 | 1.23 |
| 70-74 | 300 | 0.45 | 0.54 | 0.64 | 0.73 | 0.79 | 0.83 | 0.89 | 0.93 | 1.00 | 1.09 | 1.15 |
| 75-79 | 162 | 0.37 | 0.47 | 0.59 | 0.65 | 0.72 | 0.76 | 0.82 | 0.87 | 0.94 | 1.03 | 1.11 |
| 80-84 | 259 | 0.31 | 0.38 | 0.50 | 0.57 | 0.63 | 0.68 | 0.74 | 0.79 | 0.84 | 0.94 | 0.98 |
| 85-89 | 107 | 0.26 | 0.32 | 0.40 | 0.46 | 0.51 | 0.54 | 0.59 | 0.67 | 0.75 | 0.88 | 0.93 |
| 90-94 | 24 | 0.25 | 0.37 | 0.41 | 0.46 | 0.49 | 0.52 | 0.56 | 0.63 | 0.79 | 0.93 | 0.99 |
| 95-99 | n/a | n/a | n/a | n/a | n/a | n/a | n/a | n/a | n/a | n/a | n/a | n/a |
| 100+ | n/a | n/a | n/a | n/a | n/a | n/a | n/a | n/a | n/a | n/a | n/a | n/a |
| PIONEER = PopulatION HEalth and Eye Disease PRofilE in Elderly Singaporeans Study | | | | | | | | | | | | |

**Supplementary file 61.** Reference values for 4 m gait speed for males in Singapore (PIONEER; pooled *n* = 1,138)

| **Age (years)** | ***n*** | **Percentile (m/s)** | | | | | | | | | | |
| --- | --- | --- | --- | --- | --- | --- | --- | --- | --- | --- | --- | --- |
|  |  | **5^th^** | **10^th^** | **20^th^** | **30^th^** | **40^th^** | **50^th^** | **60^th^** | **70^th^** | **80^th^** | **90^th^** | **95^th^** |
| 20-24 | n/a | n/a | n/a | n/a | n/a | n/a | n/a | n/a | n/a | n/a | n/a | n/a |
| 25-29 | n/a | n/a | n/a | n/a | n/a | n/a | n/a | n/a | n/a | n/a | n/a | n/a |
| 30-34 | n/a | n/a | n/a | n/a | n/a | n/a | n/a | n/a | n/a | n/a | n/a | n/a |
| 35-39 | n/a | n/a | n/a | n/a | n/a | n/a | n/a | n/a | n/a | n/a | n/a | n/a |
| 40-44 | n/a | n/a | n/a | n/a | n/a | n/a | n/a | n/a | n/a | n/a | n/a | n/a |
| 45-49 | n/a | n/a | n/a | n/a | n/a | n/a | n/a | n/a | n/a | n/a | n/a | n/a |
| 50-54 | n/a | n/a | n/a | n/a | n/a | n/a | n/a | n/a | n/a | n/a | n/a | n/a |
| 55-59 | n/a | n/a | n/a | n/a | n/a | n/a | n/a | n/a | n/a | n/a | n/a | n/a |
| 60-64 | 222 | 0.65 | 0.77 | 0.86 | 0.91 | 0.96 | 1.03 | 1.08 | 1.11 | 1.19 | 1.25 | 1.33 |
| 65-69 | 231 | 0.67 | 0.73 | 0.83 | 0.88 | 0.94 | 0.97 | 1.02 | 1.10 | 1.15 | 1.27 | 1.40 |
| 70-74 | 217 | 0.52 | 0.56 | 0.70 | 0.76 | 0.83 | 0.89 | 0.93 | 1.00 | 1.05 | 1.18 | 1.28 |
| 75-79 | 144 | 0.46 | 0.53 | 0.62 | 0.70 | 0.78 | 0.83 | 0.86 | 0.91 | 0.98 | 1.06 | 1.11 |
| 80-84 | 219 | 0.44 | 0.49 | 0.59 | 0.67 | 0.73 | 0.77 | 0.81 | 0.88 | 0.93 | 1.05 | 1.16 |
| 85-89 | 105 | 0.38 | 0.43 | 0.52 | 0.58 | 0.63 | 0.68 | 0.74 | 0.82 | 0.89 | 0.98 | 1.03 |
| 90-94 | n/a | n/a | n/a | n/a | n/a | n/a | n/a | n/a | n/a | n/a | n/a | n/a |
| 95-99 | n/a | n/a | n/a | n/a | n/a | n/a | n/a | n/a | n/a | n/a | n/a | n/a |
| 100+ | n/a | n/a | n/a | n/a | n/a | n/a | n/a | n/a | n/a | n/a | n/a | n/a |
| PIONEER = PopulatION HEalth and Eye Disease PRofilE in Elderly Singaporeans Study | | | | | | | | | | | | |

**Supplementary file 62.** Reference values for 4 m gait speed for females in Sri Lanka (SLHAS; pooled *n* = 3,192)

| **Age (years)** | ***n*** | **Percentile (m/s)** | | | | | | | | | | |
| --- | --- | --- | --- | --- | --- | --- | --- | --- | --- | --- | --- | --- |
|  |  | **5^th^** | **10^th^** | **20^th^** | **30^th^** | **40^th^** | **50^th^** | **60^th^** | **70^th^** | **80^th^** | **90^th^** | **95^th^** |
| 20-24 | 188 | 0.56 | 0.62 | 0.70 | 0.75 | 0.78 | 0.82 | 0.86 | 0.89 | 0.94 | 1.00 | 1.09 |
| 25-29 | 256 | 0.58 | 0.65 | 0.71 | 0.75 | 0.78 | 0.82 | 0.85 | 0.88 | 0.94 | 1.00 | 1.08 |
| 30-34 | 204 | 0.57 | 0.61 | 0.68 | 0.72 | 0.76 | 0.79 | 0.83 | 0.87 | 0.92 | 0.98 | 1.03 |
| 35-39 | 353 | 0.53 | 0.60 | 0.65 | 0.69 | 0.74 | 0.78 | 0.81 | 0.85 | 0.90 | 1.00 | 1.06 |
| 40-44 | 288 | 0.51 | 0.57 | 0.64 | 0.68 | 0.72 | 0.75 | 0.78 | 0.84 | 0.90 | 0.95 | 1.01 |
| 45-49 | 288 | 0.48 | 0.56 | 0.63 | 0.67 | 0.71 | 0.75 | 0.78 | 0.81 | 0.86 | 0.94 | 1.00 |
| 50-54 | 265 | 0.47 | 0.51 | 0.57 | 0.60 | 0.66 | 0.70 | 0.75 | 0.80 | 0.84 | 0.91 | 0.95 |
| 55-59 | 290 | 0.44 | 0.49 | 0.57 | 0.63 | 0.66 | 0.70 | 0.74 | 0.78 | 0.83 | 0.90 | 0.97 |
| 60-64 | 282 | 0.40 | 0.46 | 0.51 | 0.56 | 0.61 | 0.65 | 0.68 | 0.72 | 0.76 | 0.84 | 0.92 |
| 65-69 | 275 | 0.35 | 0.39 | 0.46 | 0.53 | 0.57 | 0.61 | 0.65 | 0.69 | 0.74 | 0.84 | 0.91 |
| 70-74 | 294 | 0.32 | 0.35 | 0.42 | 0.48 | 0.53 | 0.57 | 0.61 | 0.65 | 0.70 | 0.78 | 0.85 |
| 75-79 | 144 | 0.28 | 0.34 | 0.38 | 0.42 | 0.46 | 0.51 | 0.54 | 0.61 | 0.66 | 0.73 | 0.83 |
| 80-84 | 65 | 0.27 | 0.28 | 0.32 | 0.37 | 0.42 | 0.46 | 0.53 | 0.58 | 0.62 | 0.71 | 0.75 |
| 85-89 | n/a | n/a | n/a | n/a | n/a | n/a | n/a | n/a | n/a | n/a | n/a | n/a |
| 90-94 | n/a | n/a | n/a | n/a | n/a | n/a | n/a | n/a | n/a | n/a | n/a | n/a |
| 95-99 | n/a | n/a | n/a | n/a | n/a | n/a | n/a | n/a | n/a | n/a | n/a | n/a |
| 100+ | n/a | n/a | n/a | n/a | n/a | n/a | n/a | n/a | n/a | n/a | n/a | n/a |
| SLHAS = Sri Lanka Health and Ageing Study | | | | | | | | | | | | |

**Supplementary file 63.** Reference values for 4 m gait speed for males in Sri Lanka (SLHAS; pooled *n* = 3,074)

| **Age (years)** | ***n*** | **Percentile (m/s)** | | | | | | | | | | |
| --- | --- | --- | --- | --- | --- | --- | --- | --- | --- | --- | --- | --- |
|  |  | **5^th^** | **10^th^** | **20^th^** | **30^th^** | **40^th^** | **50^th^** | **60^th^** | **70^th^** | **80^th^** | **90^th^** | **95^th^** |
| 20-24 | 172 | 0.64 | 0.71 | 0.74 | 0.79 | 0.83 | 0.86 | 0.92 | 0.97 | 1.02 | 1.10 | 1.18 |
| 25-29 | 175 | 0.64 | 0.70 | 0.76 | 0.82 | 0.86 | 0.91 | 0.97 | 0.99 | 1.03 | 1.09 | 1.17 |
| 30-34 | 188 | 0.61 | 0.70 | 0.77 | 0.82 | 0.86 | 0.89 | 0.91 | 0.95 | 1.02 | 1.09 | 1.16 |
| 35-39 | 366 | 0.59 | 0.67 | 0.73 | 0.78 | 0.82 | 0.86 | 0.90 | 0.93 | 0.98 | 1.06 | 1.14 |
| 40-44 | 289 | 0.56 | 0.67 | 0.74 | 0.78 | 0.82 | 0.86 | 0.90 | 0.95 | 1.00 | 1.08 | 1.16 |
| 45-49 | 294 | 0.55 | 0.62 | 0.71 | 0.75 | 0.82 | 0.86 | 0.88 | 0.93 | 0.98 | 1.07 | 1.15 |
| 50-54 | 272 | 0.55 | 0.60 | 0.68 | 0.72 | 0.77 | 0.81 | 0.85 | 0.89 | 0.93 | 1.02 | 1.09 |
| 55-59 | 301 | 0.49 | 0.53 | 0.61 | 0.69 | 0.73 | 0.78 | 0.84 | 0.87 | 0.93 | 1.01 | 1.10 |
| 60-64 | 302 | 0.45 | 0.50 | 0.59 | 0.64 | 0.71 | 0.76 | 0.80 | 0.85 | 0.91 | 0.97 | 1.05 |
| 65-69 | 258 | 0.42 | 0.47 | 0.54 | 0.61 | 0.67 | 0.70 | 0.76 | 0.80 | 0.86 | 0.93 | 0.98 |
| 70-74 | 242 | 0.41 | 0.46 | 0.53 | 0.58 | 0.62 | 0.66 | 0.69 | 0.74 | 0.79 | 0.86 | 0.91 |
| 75-79 | 150 | 0.35 | 0.39 | 0.48 | 0.52 | 0.59 | 0.64 | 0.71 | 0.77 | 0.83 | 0.92 | 0.94 |
| 80-84 | 65 | 0.32 | 0.37 | 0.45 | 0.49 | 0.51 | 0.55 | 0.59 | 0.63 | 0.65 | 0.70 | 0.73 |
| 85-89 | n/a | n/a | n/a | n/a | n/a | n/a | n/a | n/a | n/a | n/a | n/a | n/a |
| 90-94 | n/a | n/a | n/a | n/a | n/a | n/a | n/a | n/a | n/a | n/a | n/a | n/a |
| 95-99 | n/a | n/a | n/a | n/a | n/a | n/a | n/a | n/a | n/a | n/a | n/a | n/a |
| 100+ | n/a | n/a | n/a | n/a | n/a | n/a | n/a | n/a | n/a | n/a | n/a | n/a |
| SLHAS = Sri Lanka Health and Ageing Study | | | | | | | | | | | | |

**Supplementary file 64.** Reference values for 3 m gait speed for females in Lebanon (LSAHA; pooled *n* = 1,238)

| **Age (years)** | ***n*** | **Percentile (m/s)** | | | | | | | | | | |
| --- | --- | --- | --- | --- | --- | --- | --- | --- | --- | --- | --- | --- |
|  |  | **5^th^** | **10^th^** | **20^th^** | **30^th^** | **40^th^** | **50^th^** | **60^th^** | **70^th^** | **80^th^** | **90^th^** | **95^th^** |
| 20-24 | n/a | n/a | n/a | n/a | n/a | n/a | n/a | n/a | n/a | n/a | n/a | n/a |
| 25-29 | n/a | n/a | n/a | n/a | n/a | n/a | n/a | n/a | n/a | n/a | n/a | n/a |
| 30-34 | n/a | n/a | n/a | n/a | n/a | n/a | n/a | n/a | n/a | n/a | n/a | n/a |
| 35-39 | n/a | n/a | n/a | n/a | n/a | n/a | n/a | n/a | n/a | n/a | n/a | n/a |
| 40-44 | n/a | n/a | n/a | n/a | n/a | n/a | n/a | n/a | n/a | n/a | n/a | n/a |
| 45-49 | n/a | n/a | n/a | n/a | n/a | n/a | n/a | n/a | n/a | n/a | n/a | n/a |
| 50-54 | n/a | n/a | n/a | n/a | n/a | n/a | n/a | n/a | n/a | n/a | n/a | n/a |
| 55-59 | 21 | 0.38 | 0.43 | 0.50 | 0.60 | 0.60 | 0.75 | 0.75 | 1.00 | 1.00 | 1.00 | 1.00 |
| 60-64 | 371 | 0.30 | 0.38 | 0.50 | 0.60 | 0.60 | 0.75 | 0.75 | 1.00 | 1.00 | 1.00 | 1.50 |
| 65-69 | 327 | 0.30 | 0.38 | 0.50 | 0.60 | 0.60 | 0.75 | 0.75 | 0.75 | 1.00 | 1.00 | 1.50 |
| 70-74 | 235 | 0.27 | 0.33 | 0.43 | 0.50 | 0.60 | 0.60 | 0.75 | 0.75 | 0.75 | 1.00 | 1.00 |
| 75-79 | 160 | 0.27 | 0.30 | 0.38 | 0.43 | 0.50 | 0.55 | 0.60 | 0.75 | 0.75 | 1.00 | 1.00 |
| 80-84 | 94 | 0.18 | 0.21 | 0.30 | 0.38 | 0.43 | 0.50 | 0.60 | 0.60 | 0.75 | 0.75 | 1.00 |
| 85-89 | 30 | 0.25 | 0.25 | 0.29 | 0.38 | 0.47 | 0.50 | 0.54 | 0.60 | 0.75 | 0.75 | 0.89 |
| 90-94 | n/a | n/a | n/a | n/a | n/a | n/a | n/a | n/a | n/a | n/a | n/a | n/a |
| 95-99 | n/a | n/a | n/a | n/a | n/a | n/a | n/a | n/a | n/a | n/a | n/a | n/a |
| 100+ | n/a | n/a | n/a | n/a | n/a | n/a | n/a | n/a | n/a | n/a | n/a | n/a |
| LSAHA = Lebanon Study on Aging and HeAlth | | | | | | | | | | | | |

**Supplementary file 65.** Reference values for 3 m gait speed for males in Lebanon (LSAHA; pooled *n* = 735)

| **Age (years)** | ***n*** | **Percentile (m/s)** | | | | | | | | | | |
| --- | --- | --- | --- | --- | --- | --- | --- | --- | --- | --- | --- | --- |
|  |  | **5^th^** | **10^th^** | **20^th^** | **30^th^** | **40^th^** | **50^th^** | **60^th^** | **70^th^** | **80^th^** | **90^th^** | **95^th^** |
| 20-24 | n/a | n/a | n/a | n/a | n/a | n/a | n/a | n/a | n/a | n/a | n/a | n/a |
| 25-29 | n/a | n/a | n/a | n/a | n/a | n/a | n/a | n/a | n/a | n/a | n/a | n/a |
| 30-34 | n/a | n/a | n/a | n/a | n/a | n/a | n/a | n/a | n/a | n/a | n/a | n/a |
| 35-39 | n/a | n/a | n/a | n/a | n/a | n/a | n/a | n/a | n/a | n/a | n/a | n/a |
| 40-44 | n/a | n/a | n/a | n/a | n/a | n/a | n/a | n/a | n/a | n/a | n/a | n/a |
| 45-49 | n/a | n/a | n/a | n/a | n/a | n/a | n/a | n/a | n/a | n/a | n/a | n/a |
| 50-54 | n/a | n/a | n/a | n/a | n/a | n/a | n/a | n/a | n/a | n/a | n/a | n/a |
| 55-59 | n/a | n/a | n/a | n/a | n/a | n/a | n/a | n/a | n/a | n/a | n/a | n/a |
| 60-64 | 170 | 0.33 | 0.43 | 0.60 | 0.60 | 0.75 | 0.75 | 1.00 | 1.00 | 1.00 | 1.50 | 1.50 |
| 65-69 | 186 | 0.38 | 0.43 | 0.50 | 0.60 | 0.75 | 0.75 | 0.75 | 1.00 | 1.00 | 1.00 | 1.50 |
| 70-74 | 161 | 0.30 | 0.38 | 0.50 | 0.60 | 0.60 | 0.75 | 0.75 | 1.00 | 1.00 | 1.00 | 1.50 |
| 75-79 | 109 | 0.30 | 0.38 | 0.43 | 0.50 | 0.60 | 0.60 | 0.75 | 0.75 | 0.85 | 1.00 | 1.00 |
| 80-84 | 83 | 0.30 | 0.34 | 0.43 | 0.50 | 0.60 | 0.60 | 0.75 | 0.75 | 1.00 | 1.00 | 1.00 |
| 85-89 | 26 | 0.33 | 0.33 | 0.38 | 0.50 | 0.50 | 0.60 | 0.60 | 0.75 | 0.75 | 1.00 | 1.00 |
| 90-94 | n/a | n/a | n/a | n/a | n/a | n/a | n/a | n/a | n/a | n/a | n/a | n/a |
| 95-99 | n/a | n/a | n/a | n/a | n/a | n/a | n/a | n/a | n/a | n/a | n/a | n/a |
| 100+ | n/a | n/a | n/a | n/a | n/a | n/a | n/a | n/a | n/a | n/a | n/a | n/a |
| LSAHA = Lebanon Study on Aging and HeAlth | | | | | | | | | | | | |

**Supplementary file 66.** Reference values for 10 m gait speed for females in Singapore (WISE; pooled *n* = 2,058)

| **Age (years)** | ***n*** | **Percentile (m/s)** | | | | | | | | | | |
| --- | --- | --- | --- | --- | --- | --- | --- | --- | --- | --- | --- | --- |
|  |  | **5^th^** | **10^th^** | **20^th^** | **30^th^** | **40^th^** | **50^th^** | **60^th^** | **70^th^** | **80^th^** | **90^th^** | **95^th^** |
| 20-24 | n/a | n/a | n/a | n/a | n/a | n/a | n/a | n/a | n/a | n/a | n/a | n/a |
| 25-29 | n/a | n/a | n/a | n/a | n/a | n/a | n/a | n/a | n/a | n/a | n/a | n/a |
| 30-34 | n/a | n/a | n/a | n/a | n/a | n/a | n/a | n/a | n/a | n/a | n/a | n/a |
| 35-39 | n/a | n/a | n/a | n/a | n/a | n/a | n/a | n/a | n/a | n/a | n/a | n/a |
| 40-44 | n/a | n/a | n/a | n/a | n/a | n/a | n/a | n/a | n/a | n/a | n/a | n/a |
| 45-49 | n/a | n/a | n/a | n/a | n/a | n/a | n/a | n/a | n/a | n/a | n/a | n/a |
| 50-54 | n/a | n/a | n/a | n/a | n/a | n/a | n/a | n/a | n/a | n/a | n/a | n/a |
| 55-59 | n/a | n/a | n/a | n/a | n/a | n/a | n/a | n/a | n/a | n/a | n/a | n/a |
| 60-64 | 508 | 0.45 | 0.53 | 0.63 | 0.67 | 0.71 | 0.77 | 0.83 | 0.83 | 0.91 | 1.00 | 1.11 |
| 65-69 | 449 | 0.44 | 0.56 | 0.63 | 0.67 | 0.71 | 0.77 | 0.83 | 0.91 | 0.91 | 1.00 | 1.11 |
| 70-74 | 337 | 0.33 | 0.42 | 0.50 | 0.59 | 0.63 | 0.67 | 0.71 | 0.77 | 0.83 | 0.91 | 1.00 |
| 75-79 | 335 | 0.29 | 0.34 | 0.42 | 0.48 | 0.56 | 0.59 | 0.63 | 0.67 | 0.71 | 0.83 | 0.91 |
| 80-84 | 220 | 0.25 | 0.30 | 0.37 | 0.40 | 0.45 | 0.50 | 0.56 | 0.59 | 0.67 | 0.77 | 0.83 |
| 85-89 | 158 | 0.23 | 0.25 | 0.31 | 0.38 | 0.42 | 0.43 | 0.50 | 0.55 | 0.61 | 0.67 | 0.71 |
| 90-94 | 51 | 0.18 | 0.21 | 0.30 | 0.33 | 0.36 | 0.42 | 0.43 | 0.45 | 0.50 | 0.56 | 0.63 |
| 95-99 | n/a | n/a | n/a | n/a | n/a | n/a | n/a | n/a | n/a | n/a | n/a | n/a |
| 100+ | n/a | n/a | n/a | n/a | n/a | n/a | n/a | n/a | n/a | n/a | n/a | n/a |
| WISE = Well-being of the Singapore Elderly | | | | | | | | | | | | |

**Supplementary file 67.** Reference values for 10 m gait speed for males in Singapore (WISE; pooled *n* = 1,796)

| **Age (years)** | ***n*** | **Percentile (m/s)** | | | | | | | | | | |
| --- | --- | --- | --- | --- | --- | --- | --- | --- | --- | --- | --- | --- |
|  |  | **5^th^** | **10^th^** | **20^th^** | **30^th^** | **40^th^** | **50^th^** | **60^th^** | **70^th^** | **80^th^** | **90^th^** | **95^th^** |
| 20-24 | n/a | n/a | n/a | n/a | n/a | n/a | n/a | n/a | n/a | n/a | n/a | n/a |
| 25-29 | n/a | n/a | n/a | n/a | n/a | n/a | n/a | n/a | n/a | n/a | n/a | n/a |
| 30-34 | n/a | n/a | n/a | n/a | n/a | n/a | n/a | n/a | n/a | n/a | n/a | n/a |
| 35-39 | n/a | n/a | n/a | n/a | n/a | n/a | n/a | n/a | n/a | n/a | n/a | n/a |
| 40-44 | n/a | n/a | n/a | n/a | n/a | n/a | n/a | n/a | n/a | n/a | n/a | n/a |
| 45-49 | n/a | n/a | n/a | n/a | n/a | n/a | n/a | n/a | n/a | n/a | n/a | n/a |
| 50-54 | n/a | n/a | n/a | n/a | n/a | n/a | n/a | n/a | n/a | n/a | n/a | n/a |
| 55-59 | n/a | n/a | n/a | n/a | n/a | n/a | n/a | n/a | n/a | n/a | n/a | n/a |
| 60-64 | 463 | 0.56 | 0.63 | 0.71 | 0.77 | 0.83 | 0.83 | 0.91 | 1.00 | 1.00 | 1.11 | 1.25 |
| 65-69 | 427 | 0.50 | 0.59 | 0.63 | 0.67 | 0.71 | 0.77 | 0.83 | 0.85 | 0.91 | 1.00 | 1.11 |
| 70-74 | 253 | 0.40 | 0.48 | 0.56 | 0.63 | 0.67 | 0.71 | 0.77 | 0.83 | 0.83 | 0.91 | 1.00 |
| 75-79 | 282 | 0.32 | 0.42 | 0.50 | 0.56 | 0.63 | 0.67 | 0.71 | 0.77 | 0.83 | 0.91 | 1.00 |
| 80-84 | 191 | 0.28 | 0.36 | 0.43 | 0.50 | 0.56 | 0.59 | 0.63 | 0.67 | 0.77 | 0.83 | 0.91 |
| 85-89 | 137 | 0.21 | 0.29 | 0.35 | 0.42 | 0.48 | 0.53 | 0.59 | 0.63 | 0.71 | 0.77 | 0.85 |
| 90-94 | 43 | 0.23 | 0.25 | 0.31 | 0.33 | 0.40 | 0.45 | 0.50 | 0.54 | 0.59 | 0.71 | 0.71 |
| 95-99 | n/a | n/a | n/a | n/a | n/a | n/a | n/a | n/a | n/a | n/a | n/a | n/a |
| 100+ | n/a | n/a | n/a | n/a | n/a | n/a | n/a | n/a | n/a | n/a | n/a | n/a |
| WISE = Well-being of the Singapore Elderly | | | | | | | | | | | | |

**Supplementary file 68.** Percentile curves for the Five-Times-Sit-to-Stand Test (FTSST) performance for females in Asia (CHARLS, IFLS, PHASE, SHARE; pooled *n* = 28,826)


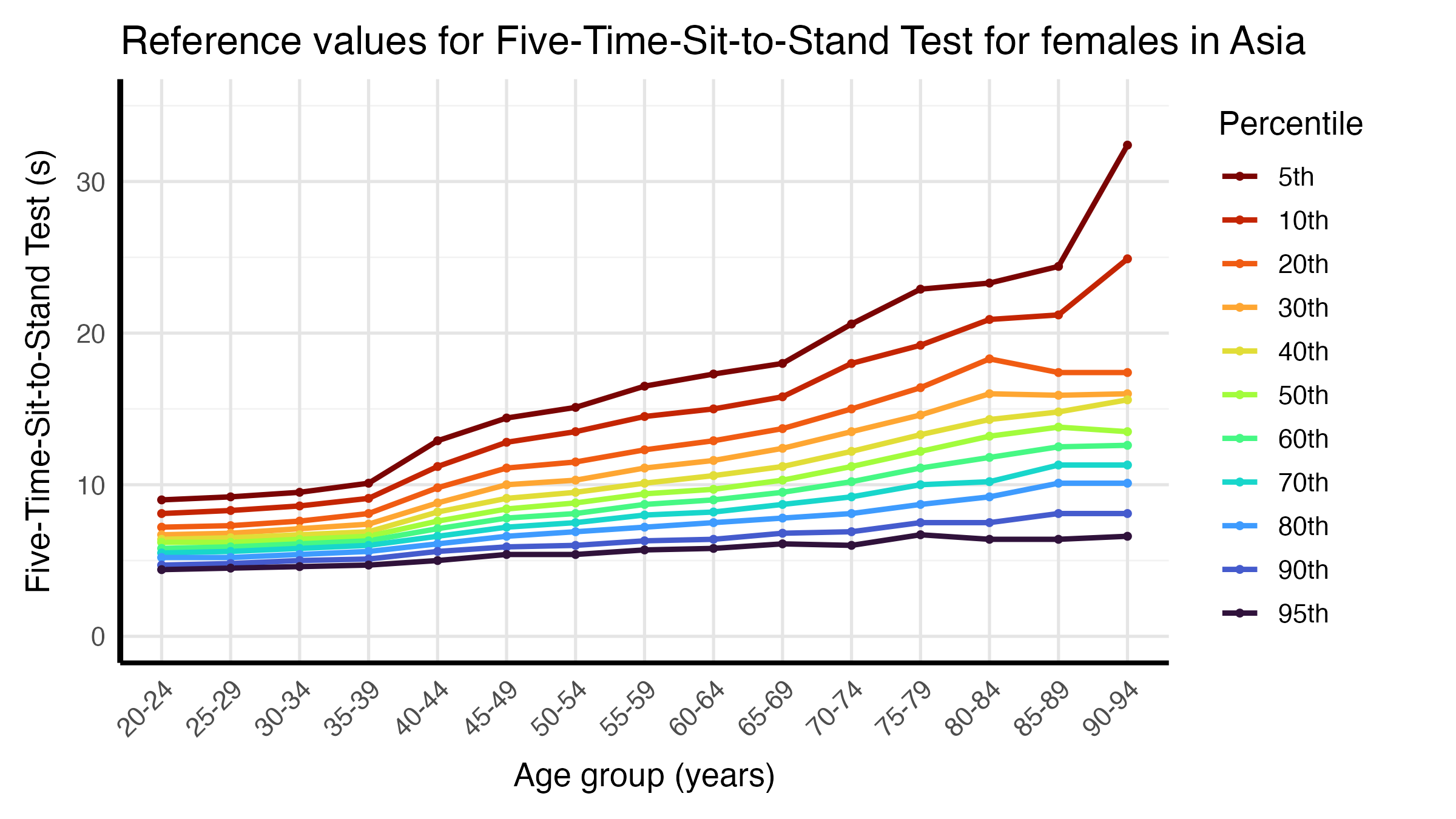


**Supplementary file 69.** Percentile curves for the Five-Times-Sit-to-Stand Test (FTSST) performance for males in Asia (CHARLS, IFLS, PHASE, SHARE; pooled *n* = 26,409)


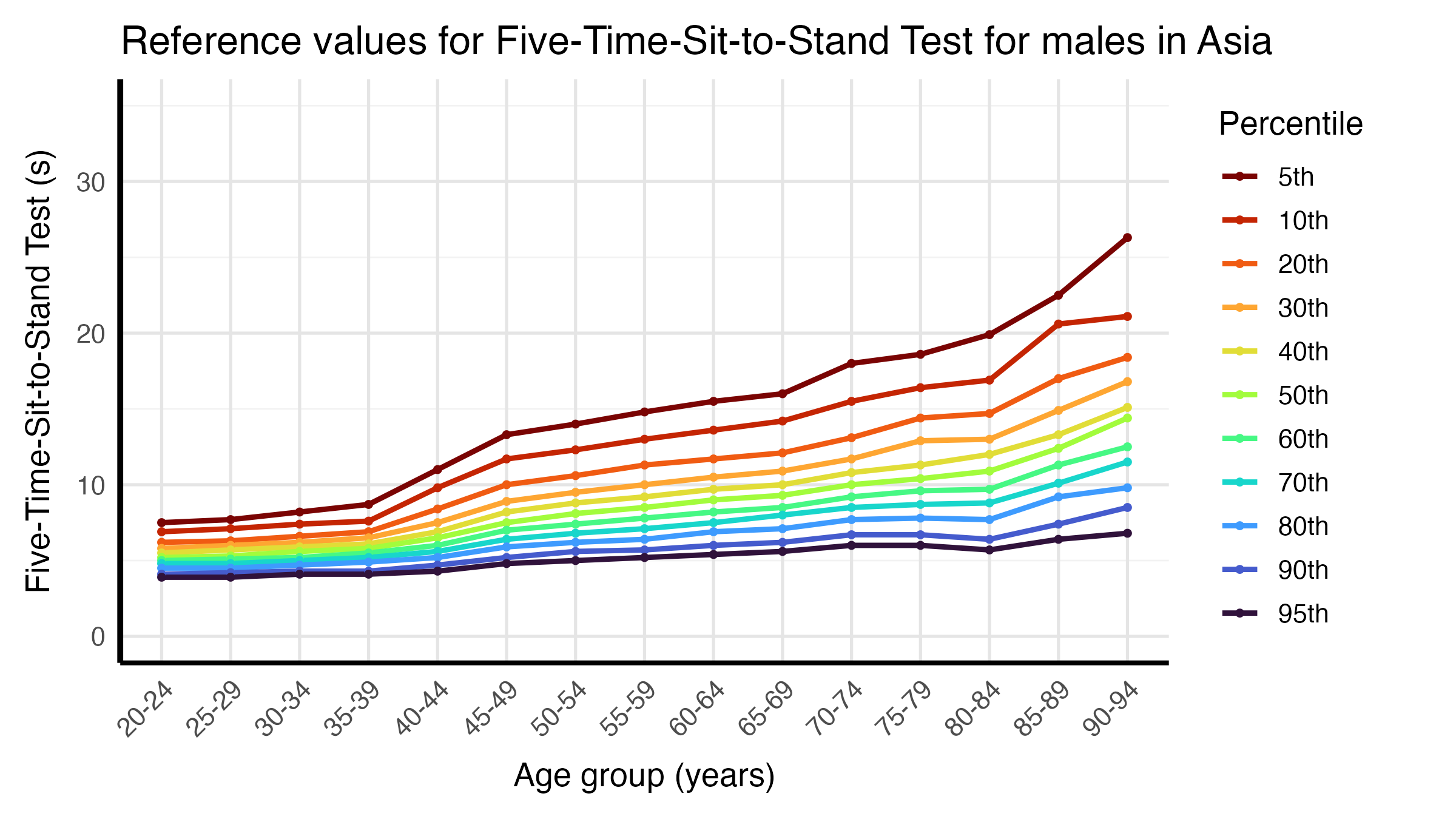


**Supplementary file 70.** Reference values for the Five-Times-Sit-to-Stand Test (FTSST) performance for females in Southeast Asia (IFLS, PHASE; pooled *n* = 1,661)

| **Age (years)** | ***n*** | **Percentile (s)** | | | | | | | | | | |
| --- | --- | --- | --- | --- | --- | --- | --- | --- | --- | --- | --- | --- |
|  |  | **5^th^** | **10^th^** | **20^th^** | **30^th^** | **40^th^** | **50^th^** | **60^th^** | **70^th^** | **80^th^** | **90^th^** | **95^th^** |
| 20-24 | n/a | n/a | n/a | n/a | n/a | n/a | n/a | n/a | n/a | n/a | n/a | n/a |
| 25-29 | n/a | n/a | n/a | n/a | n/a | n/a | n/a | n/a | n/a | n/a | n/a | n/a |
| 30-34 | n/a | n/a | n/a | n/a | n/a | n/a | n/a | n/a | n/a | n/a | n/a | n/a |
| 35-39 | n/a | n/a | n/a | n/a | n/a | n/a | n/a | n/a | n/a | n/a | n/a | n/a |
| 40-44 | n/a | n/a | n/a | n/a | n/a | n/a | n/a | n/a | n/a | n/a | n/a | n/a |
| 45-49 | n/a | n/a | n/a | n/a | n/a | n/a | n/a | n/a | n/a | n/a | n/a | n/a |
| 50-54 | n/a | n/a | n/a | n/a | n/a | n/a | n/a | n/a | n/a | n/a | n/a | n/a |
| 55-59 | n/a | n/a | n/a | n/a | n/a | n/a | n/a | n/a | n/a | n/a | n/a | n/a |
| 60-64 | n/a | n/a | n/a | n/a | n/a | n/a | n/a | n/a | n/a | n/a | n/a | n/a |
| 65-69 | 623 | 16.5 | 15.0 | 13.2 | 12.3 | 10.9 | 10.1 | 9.4 | 8.6 | 7.9 | 6.8 | 6.4 |
| 70-74 | 461 | 19.2 | 17.3 | 15.1 | 13.7 | 12.4 | 11.6 | 10.5 | 9.7 | 8.7 | 7.2 | 6.3 |
| 75-79 | 298 | 21.1 | 19.0 | 16.6 | 14.5 | 13.4 | 12.4 | 11.3 | 10.2 | 9.2 | 8.1 | 6.9 |
| 80-84 | 184 | 21.8 | 20.3 | 17.4 | 15.8 | 14.5 | 13.4 | 12.0 | 10.8 | 9.9 | 8.2 | 7.6 |
| 85-89 | 73 | 21.4 | 20.0 | 17.4 | 15.8 | 14.5 | 13.7 | 12.3 | 11.3 | 10.2 | 9.2 | 6.5 |
| 90-94 | 22 | 26.2 | 24.5 | 16.4 | 15.8 | 15.4 | 13.9 | 13.1 | 12.3 | 10.4 | 8.3 | 7.0 |
| 95-99 | n/a | n/a | n/a | n/a | n/a | n/a | n/a | n/a | n/a | n/a | n/a | n/a |
| 100+ | n/a | n/a | n/a | n/a | n/a | n/a | n/a | n/a | n/a | n/a | n/a | n/a |
| IFLS = The Indonesian Family Life Survey; PHASE = Panel on Health and Ageing of Singaporean Elderly | | | | | | | | | | | | |

**Supplementary file 71.** Reference values for the Five-Times-Sit-to-Stand Test (FTSST) performance for males in Southeast Asia (IFLS, PHASE; pooled *n* = 1,405)

| **Age (years)** | ***n*** | **Percentile (s)** | | | | | | | | | | |
| --- | --- | --- | --- | --- | --- | --- | --- | --- | --- | --- | --- | --- |
|  |  | **5^th^** | **10^th^** | **20^th^** | **30^th^** | **40^th^** | **50^th^** | **60^th^** | **70^th^** | **80^th^** | **90^th^** | **95^th^** |
| 20-24 | n/a | n/a | n/a | n/a | n/a | n/a | n/a | n/a | n/a | n/a | n/a | n/a |
| 25-29 | n/a | n/a | n/a | n/a | n/a | n/a | n/a | n/a | n/a | n/a | n/a | n/a |
| 30-34 | n/a | n/a | n/a | n/a | n/a | n/a | n/a | n/a | n/a | n/a | n/a | n/a |
| 35-39 | n/a | n/a | n/a | n/a | n/a | n/a | n/a | n/a | n/a | n/a | n/a | n/a |
| 40-44 | n/a | n/a | n/a | n/a | n/a | n/a | n/a | n/a | n/a | n/a | n/a | n/a |
| 45-49 | n/a | n/a | n/a | n/a | n/a | n/a | n/a | n/a | n/a | n/a | n/a | n/a |
| 50-54 | n/a | n/a | n/a | n/a | n/a | n/a | n/a | n/a | n/a | n/a | n/a | n/a |
| 55-59 | n/a | n/a | n/a | n/a | n/a | n/a | n/a | n/a | n/a | n/a | n/a | n/a |
| 60-64 | n/a | n/a | n/a | n/a | n/a | n/a | n/a | n/a | n/a | n/a | n/a | n/a |
| 65-69 | 542 | 15.2 | 13.5 | 11.1 | 9.9 | 9.1 | 8.4 | 8.0 | 7.3 | 6.7 | 6.0 | 5.4 |
| 70-74 | 381 | 16.0 | 14.7 | 12.4 | 11.3 | 10.6 | 9.8 | 9.1 | 8.4 | 7.8 | 6.9 | 6.2 |
| 75-79 | 237 | 17.2 | 16.0 | 14.0 | 12.3 | 10.9 | 10.2 | 9.6 | 8.6 | 7.8 | 6.7 | 5.8 |
| 80-84 | 160 | 17.4 | 15.4 | 14.3 | 13.0 | 12.0 | 10.9 | 10.1 | 9.3 | 8.2 | 6.5 | 5.6 |
| 85-89 | 85 | 21.9 | 19.8 | 16.2 | 14.6 | 12.8 | 12.0 | 10.8 | 9.8 | 9.2 | 7.3 | 6.6 |
| 90-94 | n/a | n/a | n/a | n/a | n/a | n/a | n/a | n/a | n/a | n/a | n/a | n/a |
| 95-99 | n/a | n/a | n/a | n/a | n/a | n/a | n/a | n/a | n/a | n/a | n/a | n/a |
| 100+ | n/a | n/a | n/a | n/a | n/a | n/a | n/a | n/a | n/a | n/a | n/a | n/a |
| IFLS = The Indonesian Family Life Survey; PHASE = Panel on Health and Ageing of Singaporean Elderly | | | | | | | | | | | | |

**Supplementary file 72.** Reference values for the Five-Times-Sit-to-Stand Test (FTSST) performance for females in China (CHARLS; pooled *n* = 13,582)

| **Age (years)** | ***n*** | **Percentile (s)** | | | | | | | | | | |
| --- | --- | --- | --- | --- | --- | --- | --- | --- | --- | --- | --- | --- |
|  |  | **5^th^** | **10^th^** | **20^th^** | **30^th^** | **40^th^** | **50^th^** | **60^th^** | **70^th^** | **80^th^** | **90^th^** | **95^th^** |
| 20-24 | n/a | n/a | n/a | n/a | n/a | n/a | n/a | n/a | n/a | n/a | n/a | n/a |
| 25-29 | n/a | n/a | n/a | n/a | n/a | n/a | n/a | n/a | n/a | n/a | n/a | n/a |
| 30-34 | 25 | 14.4 | 12.9 | 11.0 | 9.2 | 8.4 | 7.8 | 7.4 | 6.8 | 6.4 | 5.9 | 5.9 |
| 35-39 | 150 | 16.4 | 12.6 | 10.7 | 9.7 | 8.9 | 8.0 | 7.3 | 6.9 | 6.3 | 5.4 | 5.4 |
| 40-44 | 1,169 | 14.4 | 12.8 | 11.0 | 10.0 | 9.2 | 8.5 | 7.9 | 7.4 | 6.7 | 6.0 | 6.0 |
| 45-49 | 3,052 | 15.0 | 13.4 | 11.6 | 10.6 | 9.8 | 9.0 | 8.4 | 7.7 | 7.0 | 6.2 | 6.2 |
| 50-54 | 2,125 | 15.8 | 14.1 | 12.2 | 11.0 | 10.1 | 9.3 | 8.6 | 7.9 | 7.1 | 6.2 | 6.2 |
| 55-59 | 2,323 | 17.2 | 15.1 | 12.8 | 11.5 | 10.5 | 9.8 | 9.1 | 8.3 | 7.5 | 6.3 | 6.3 |
| 60-64 | 2,040 | 17.4 | 15.2 | 12.9 | 11.6 | 10.6 | 9.8 | 9.1 | 8.3 | 7.5 | 6.4 | 6.4 |
| 65-69 | 1,284 | 18.4 | 16.0 | 13.7 | 12.5 | 11.3 | 10.4 | 9.5 | 8.7 | 7.7 | 6.7 | 6.7 |
| 70-74 | 792 | 21.2 | 18.1 | 14.8 | 13.1 | 11.9 | 10.7 | 9.8 | 8.8 | 7.8 | 6.6 | 6.6 |
| 75-79 | 416 | 24.5 | 19.6 | 16.6 | 14.7 | 13.3 | 12.0 | 11.0 | 9.7 | 8.4 | 7.3 | 7.3 |
| 80-84 | 165 | 25.3 | 21.6 | 18.9 | 16.6 | 14.3 | 12.9 | 10.5 | 9.5 | 8.4 | 6.8 | 6.8 |
| 85-89 | 41 | 26.1 | 24.2 | 17.8 | 15.8 | 15.0 | 14.3 | 13.4 | 10.8 | 10.0 | 7.0 | 7.0 |
| 90-94 | n/a | n/a | n/a | n/a | n/a | n/a | n/a | n/a | n/a | n/a | n/a | n/a |
| 95-99 | n/a | n/a | n/a | n/a | n/a | n/a | n/a | n/a | n/a | n/a | n/a | n/a |
| 100+ | n/a | n/a | n/a | n/a | n/a | n/a | n/a | n/a | n/a | n/a | n/a | n/a |
| CHARLS = China Health and Retirement Longitudinal Study | | | | | | | | | | | | |

**Supplementary file 73.** Reference values for the Five-Times-Sit-to-Stand Test (FTSST) performance for males in China (CHARLS; pooled *n* = 12,604)

| **Age (years)** | ***n*** | **Percentile (s)** | | | | | | | | | | |
| --- | --- | --- | --- | --- | --- | --- | --- | --- | --- | --- | --- | --- |
|  |  | **5^th^** | **10^th^** | **20^th^** | **30^th^** | **40^th^** | **50^th^** | **60^th^** | **70^th^** | **80^th^** | **90^th^** | **95^th^** |
| 20-24 | n/a | n/a | n/a | n/a | n/a | n/a | n/a | n/a | n/a | n/a | n/a | n/a |
| 25-29 | n/a | n/a | n/a | n/a | n/a | n/a | n/a | n/a | n/a | n/a | n/a | n/a |
| 30-34 | n/a | n/a | n/a | n/a | n/a | n/a | n/a | n/a | n/a | n/a | n/a | n/a |
| 35-39 | n/a | n/a | n/a | n/a | n/a | n/a | n/a | n/a | n/a | n/a | n/a | n/a |
| 40-44 | 525 | 13.5 | 11.8 | 10.3 | 9.5 | 8.6 | 8.1 | 7.5 | 6.9 | 6.2 | 5.5 | 4.8 |
| 45-49 | 2,598 | 14.1 | 12.4 | 10.7 | 9.6 | 8.8 | 8.2 | 7.6 | 7.0 | 6.4 | 5.7 | 5.0 |
| 50-54 | 1,992 | 14.7 | 13.1 | 11.4 | 10.3 | 9.4 | 8.7 | 8.0 | 7.4 | 6.7 | 5.9 | 5.3 |
| 55-59 | 2,163 | 15.4 | 13.7 | 11.9 | 10.6 | 9.7 | 9.0 | 8.4 | 7.7 | 6.8 | 6.0 | 5.4 |
| 60-64 | 2,085 | 15.9 | 13.8 | 12.0 | 10.8 | 10.0 | 9.3 | 8.6 | 7.9 | 7.2 | 6.3 | 5.6 |
| 65-69 | 1,440 | 15.8 | 14.2 | 12.3 | 11.0 | 10.2 | 9.5 | 8.8 | 8.2 | 7.4 | 6.4 | 5.7 |
| 70-74 | 958 | 18.1 | 15.7 | 13.2 | 11.8 | 10.8 | 10.0 | 9.3 | 8.5 | 7.7 | 6.7 | 6.0 |
| 75-79 | 567 | 19.1 | 16.4 | 14.5 | 13.0 | 11.3 | 10.4 | 9.5 | 8.7 | 7.7 | 6.6 | 6.0 |
| 80-84 | 221 | 22.7 | 18.2 | 15.3 | 13.0 | 11.8 | 10.5 | 9.3 | 8.4 | 7.5 | 6.3 | 5.7 |
| 85-89 | 55 | 22.8 | 20.5 | 17.7 | 16.2 | 13.4 | 12.3 | 11.4 | 10.1 | 8.7 | 7.4 | 6.2 |
| 90-94 | n/a | n/a | n/a | n/a | n/a | n/a | n/a | n/a | n/a | n/a | n/a | n/a |
| 95-99 | n/a | n/a | n/a | n/a | n/a | n/a | n/a | n/a | n/a | n/a | n/a | n/a |
| 100+ | n/a | n/a | n/a | n/a | n/a | n/a | n/a | n/a | n/a | n/a | n/a | n/a |
| CHARLS = China Health and Retirement Longitudinal Study | | | | | | | | | | | | |

**Supplementary file 74.** Reference values for the Five-Times-Sit-to-Stand Test (FTSST) performance for females in Indonesia (IFLS; pooled *n* = 13,106)

| **Age (years)** | ***n*** | **Percentile (s)** | | | | | | | | | | |
| --- | --- | --- | --- | --- | --- | --- | --- | --- | --- | --- | --- | --- |
|  |  | **5^th^** | **10^th^** | **20^th^** | **30^th^** | **40^th^** | **50^th^** | **60^th^** | **70^th^** | **80^th^** | **90^th^** | **95^th^** |
| 20-24 | 1,968 | 8.9 | 8.1 | 7.2 | 6.7 | 6.4 | 6.2 | 5.8 | 5.5 | 5.2 | 4.7 | 4.4 |
| 25-29 | 2,231 | 9.2 | 8.2 | 7.3 | 6.8 | 6.5 | 6.2 | 5.9 | 5.6 | 5.2 | 4.8 | 4.5 |
| 30-34 | 1,734 | 9.5 | 8.5 | 7.6 | 7.1 | 6.7 | 6.4 | 6.1 | 5.8 | 5.4 | 5.0 | 4.6 |
| 35-39 | 1,523 | 9.6 | 8.7 | 7.8 | 7.2 | 6.8 | 6.5 | 6.2 | 5.9 | 5.6 | 5.1 | 4.7 |
| 40-44 | 1,282 | 10.3 | 9.4 | 8.5 | 7.9 | 7.3 | 6.9 | 6.6 | 6.2 | 5.8 | 5.2 | 4.8 |
| 45-49 | 1,167 | 11.3 | 10.0 | 8.9 | 8.2 | 7.7 | 7.2 | 6.8 | 6.4 | 5.9 | 5.4 | 5.0 |
| 50-54 | 918 | 12.3 | 11.0 | 9.8 | 9.0 | 8.4 | 8.0 | 7.4 | 6.9 | 6.4 | 5.7 | 5.3 |
| 55-59 | 662 | 14.0 | 12.1 | 10.3 | 9.7 | 8.9 | 8.3 | 7.7 | 7.2 | 6.7 | 6.1 | 5.6 |
| 60-64 | 517 | 15.5 | 13.9 | 12.2 | 10.9 | 10.1 | 9.4 | 8.9 | 8.2 | 7.6 | 6.5 | 5.9 |
| 65-69 | 489 | 15.9 | 14.3 | 12.6 | 11.5 | 10.5 | 9.8 | 9.2 | 8.5 | 7.8 | 6.9 | 6.4 |
| 70-74 | 298 | 18.3 | 16.8 | 14.6 | 13.4 | 12.1 | 11.0 | 10.2 | 9.5 | 8.5 | 7.2 | 6.4 |
| 75-79 | 181 | 21.1 | 18.9 | 15.3 | 13.4 | 12.5 | 11.4 | 10.3 | 9.3 | 8.7 | 7.6 | 6.7 |
| 80-84 | 101 | 20.3 | 18.7 | 16.1 | 14.7 | 13.8 | 12.1 | 10.8 | 9.9 | 9.2 | 7.8 | 7.1 |
| 85-89 | 35 | 20.1 | 18.0 | 16.0 | 15.1 | 13.9 | 12.5 | 11.8 | 10.5 | 9.7 | 9.2 | 8.0 |
| 90-94 | n/a | n/a | n/a | n/a | n/a | n/a | n/a | n/a | n/a | n/a | n/a | n/a |
| 95-99 | n/a | n/a | n/a | n/a | n/a | n/a | n/a | n/a | n/a | n/a | n/a | n/a |
| 100+ | n/a | n/a | n/a | n/a | n/a | n/a | n/a | n/a | n/a | n/a | n/a | n/a |
| IFLS = The Indonesian Family Life Survey | | | | | | | | | | | | |

**Supplementary file 75.** Reference values for the Five-Times-Sit-to-Stand Test (FTSST) performance for males in Indonesia (IFLS; pooled *n* = 11,983)

| **Age (years)** | ***n*** | **Percentile (s)** | | | | | | | | | | |
| --- | --- | --- | --- | --- | --- | --- | --- | --- | --- | --- | --- | --- |
|  |  | **5^th^** | **10^th^** | **20^th^** | **30^th^** | **40^th^** | **50^th^** | **60^th^** | **70^th^** | **80^th^** | **90^th^** | **95^th^** |
| 20-24 | 1,583 | 7.5 | 6.9 | 6.2 | 5.8 | 5.5 | 5.2 | 5.0 | 4.8 | 4.5 | 4.1 | 3.9 |
| 25-29 | 2,018 | 7.7 | 7.1 | 6.3 | 6.0 | 5.7 | 5.3 | 5.1 | 4.8 | 4.5 | 4.2 | 3.9 |
| 30-34 | 1,747 | 8.2 | 7.4 | 6.6 | 6.2 | 5.9 | 5.6 | 5.2 | 5.0 | 4.7 | 4.3 | 4.1 |
| 35-39 | 1,565 | 8.5 | 7.6 | 6.9 | 6.5 | 6.1 | 5.8 | 5.4 | 5.2 | 4.8 | 4.3 | 4.1 |
| 40-44 | 1,184 | 8.9 | 8.2 | 7.3 | 6.8 | 6.4 | 6.0 | 5.7 | 5.3 | 5.0 | 4.6 | 4.2 |
| 45-49 | 1,050 | 9.5 | 8.6 | 7.6 | 7.1 | 6.7 | 6.3 | 5.9 | 5.5 | 5.2 | 4.8 | 4.4 |
| 50-54 | 804 | 10.2 | 9.4 | 8.5 | 7.8 | 7.3 | 6.8 | 6.3 | 6.0 | 5.6 | 5.1 | 4.7 |
| 55-59 | 649 | 11.8 | 10.2 | 9.0 | 8.2 | 7.5 | 7.1 | 6.7 | 6.2 | 5.7 | 5.2 | 4.8 |
| 60-64 | 429 | 13.0 | 11.6 | 9.8 | 8.9 | 8.2 | 7.5 | 7.0 | 6.5 | 6.0 | 5.4 | 5.1 |
| 65-69 | 433 | 13.8 | 12.3 | 10.2 | 9.6 | 8.8 | 8.2 | 7.7 | 7.2 | 6.5 | 6.0 | 5.4 |
| 70-74 | 248 | 15.0 | 13.0 | 11.7 | 10.9 | 10.2 | 9.3 | 8.7 | 8.2 | 7.6 | 6.8 | 6.0 |
| 75-79 | 138 | 16.6 | 14.8 | 12.8 | 11.3 | 10.3 | 9.7 | 9.1 | 8.4 | 7.8 | 6.9 | 6.5 |
| 80-84 | 89 | 15.3 | 14.3 | 13.1 | 12.0 | 11.3 | 10.7 | 9.4 | 8.9 | 8.0 | 6.1 | 5.6 |
| 85-89 | 46 | 18.9 | 16.8 | 14.3 | 12.2 | 10.8 | 10.1 | 9.8 | 9.3 | 7.5 | 7.1 | 6.6 |
| 90-94 | n/a | n/a | n/a | n/a | n/a | n/a | n/a | n/a | n/a | n/a | n/a | n/a |
| 95-99 | n/a | n/a | n/a | n/a | n/a | n/a | n/a | n/a | n/a | n/a | n/a | n/a |
| 100+ | n/a | n/a | n/a | n/a | n/a | n/a | n/a | n/a | n/a | n/a | n/a | n/a |
| IFLS = The Indonesian Family Life Survey | | | | | | | | | | | | |

**Supplementary file 76.** Reference values for the Five-Times-Sit-to-Stand Test (FTSST) performance for females in Israel (SHARE; pooled *n* = 1,536)

| **Age (years)** | ***n*** | **Percentile (s)** | | | | | | | | | | |
| --- | --- | --- | --- | --- | --- | --- | --- | --- | --- | --- | --- | --- |
|  |  | **5^th^** | **10^th^** | **20^th^** | **30^th^** | **40^th^** | **50^th^** | **60^th^** | **70^th^** | **80^th^** | **90^th^** | **95^th^** |
| 20-24 | n/a | n/a | n/a | n/a | n/a | n/a | n/a | n/a | n/a | n/a | n/a | n/a |
| 25-29 | n/a | n/a | n/a | n/a | n/a | n/a | n/a | n/a | n/a | n/a | n/a | n/a |
| 30-34 | n/a | n/a | n/a | n/a | n/a | n/a | n/a | n/a | n/a | n/a | n/a | n/a |
| 35-39 | n/a | n/a | n/a | n/a | n/a | n/a | n/a | n/a | n/a | n/a | n/a | n/a |
| 40-44 | n/a | n/a | n/a | n/a | n/a | n/a | n/a | n/a | n/a | n/a | n/a | n/a |
| 45-49 | 56 | 17.8 | 17.0 | 12.5 | 11.6 | 9.0 | 8.0 | 8.0 | 7.0 | 6.9 | 6.0 | 6.0 |
| 50-54 | 192 | 15.2 | 14.9 | 12.0 | 10.2 | 10.0 | 9.0 | 8.0 | 7.3 | 7.0 | 6.0 | 6.0 |
| 55-59 | 416 | 16.1 | 14.0 | 12.0 | 11.0 | 10.0 | 9.0 | 8.8 | 8.0 | 7.2 | 6.5 | 6.0 |
| 60-64 | 396 | 18.0 | 15.5 | 13.4 | 12.0 | 11.0 | 9.6 | 8.7 | 8.0 | 7.0 | 6.0 | 5.5 |
| 65-69 | 243 | 19.2 | 17.0 | 14.9 | 13.0 | 12.0 | 11.0 | 10.0 | 9.0 | 8.0 | 7.0 | 6.2 |
| 70-74 | 184 | 20.9 | 18.2 | 15.8 | 14.1 | 13.0 | 12.0 | 11.0 | 9.8 | 8.5 | 7.3 | 6.8 |
| 75-79 | 49 | 20.8 | 17.2 | 15.0 | 13.6 | 13.0 | 12.0 | 12.0 | 11.0 | 9.0 | 7.8 | 6.6 |
| 80-84 | n/a | n/a | n/a | n/a | n/a | n/a | n/a | n/a | n/a | n/a | n/a | n/a |
| 85-89 | n/a | n/a | n/a | n/a | n/a | n/a | n/a | n/a | n/a | n/a | n/a | n/a |
| 90-94 | n/a | n/a | n/a | n/a | n/a | n/a | n/a | n/a | n/a | n/a | n/a | n/a |
| 95-99 | n/a | n/a | n/a | n/a | n/a | n/a | n/a | n/a | n/a | n/a | n/a | n/a |
| 100+ | n/a | n/a | n/a | n/a | n/a | n/a | n/a | n/a | n/a | n/a | n/a | n/a |
| SHARE = Survey of Health, Ageing and Retirement in Europe | | | | | | | | | | | | |

**Supplementary file 77.** Reference values for the Five-Times-Sit-to-Stand Test (FTSST) performance for males in Israel (SHARE; pooled *n* = 1,319)

| **Age (years)** | ***n*** | **Percentile (s)** | | | | | | | | | | |
| --- | --- | --- | --- | --- | --- | --- | --- | --- | --- | --- | --- | --- |
|  |  | **5^th^** | **10^th^** | **20^th^** | **30^th^** | **40^th^** | **50^th^** | **60^th^** | **70^th^** | **80^th^** | **90^th^** | **95^th^** |
| 20-24 | n/a | n/a | n/a | n/a | n/a | n/a | n/a | n/a | n/a | n/a | n/a | n/a |
| 25-29 | n/a | n/a | n/a | n/a | n/a | n/a | n/a | n/a | n/a | n/a | n/a | n/a |
| 30-34 | n/a | n/a | n/a | n/a | n/a | n/a | n/a | n/a | n/a | n/a | n/a | n/a |
| 35-39 | n/a | n/a | n/a | n/a | n/a | n/a | n/a | n/a | n/a | n/a | n/a | n/a |
| 40-44 | n/a | n/a | n/a | n/a | n/a | n/a | n/a | n/a | n/a | n/a | n/a | n/a |
| 45-49 | n/a | n/a | n/a | n/a | n/a | n/a | n/a | n/a | n/a | n/a | n/a | n/a |
| 50-54 | 95 | 14.0 | 12.0 | 11.0 | 10.0 | 9.0 | 8.1 | 7.0 | 7.0 | 6.0 | 6.0 | 6.0 |
| 55-59 | 345 | 14.0 | 12.5 | 11.3 | 10.0 | 9.0 | 8.0 | 7.6 | 7.0 | 6.3 | 6.0 | 5.3 |
| 60-64 | 324 | 16.1 | 14.0 | 12.0 | 10.4 | 9.1 | 8.4 | 8.0 | 7.0 | 6.3 | 5.8 | 5.0 |
| 65-69 | 240 | 17.0 | 16.0 | 13.3 | 12.0 | 11.0 | 9.8 | 9.0 | 8.0 | 7.0 | 6.0 | 5.0 |
| 70-74 | 208 | 21.0 | 16.1 | 14.0 | 12.6 | 11.3 | 10.3 | 9.5 | 8.8 | 7.3 | 6.0 | 5.7 |
| 75-79 | 78 | 20.0 | 17.0 | 15.0 | 13.0 | 13.0 | 12.0 | 10.3 | 9.0 | 8.0 | 7.0 | 6.2 |
| 80-84 | 29 | 16.0 | 14.4 | 14.0 | 14.0 | 12.0 | 12.0 | 11.0 | 10.4 | 9.0 | 7.0 | 6.9 |
| 85-89 | n/a | n/a | n/a | n/a | n/a | n/a | n/a | n/a | n/a | n/a | n/a | n/a |
| 90-94 | n/a | n/a | n/a | n/a | n/a | n/a | n/a | n/a | n/a | n/a | n/a | n/a |
| 95-99 | n/a | n/a | n/a | n/a | n/a | n/a | n/a | n/a | n/a | n/a | n/a | n/a |
| 100+ | n/a | n/a | n/a | n/a | n/a | n/a | n/a | n/a | n/a | n/a | n/a | n/a |
| SHARE = Survey of Health, Ageing and Retirement in Europe | | | | | | | | | | | | |

**Supplementary file 78.** Reference values for the Five-Times-Sit-to-Stand Test (FTSST) performance for females in Singapore (PHASE; pooled *n* = 534)

| **Age (years)** | ***n*** | **Percentile (s)** | | | | | | | | | | |
| --- | --- | --- | --- | --- | --- | --- | --- | --- | --- | --- | --- | --- |
|  |  | **5^th^** | **10^th^** | **20^th^** | **30^th^** | **40^th^** | **50^th^** | **60^th^** | **70^th^** | **80^th^** | **90^th^** | **95^th^** |
| 20-24 | n/a | n/a | n/a | n/a | n/a | n/a | n/a | n/a | n/a | n/a | n/a | n/a |
| 25-29 | n/a | n/a | n/a | n/a | n/a | n/a | n/a | n/a | n/a | n/a | n/a | n/a |
| 30-34 | n/a | n/a | n/a | n/a | n/a | n/a | n/a | n/a | n/a | n/a | n/a | n/a |
| 35-39 | n/a | n/a | n/a | n/a | n/a | n/a | n/a | n/a | n/a | n/a | n/a | n/a |
| 40-44 | n/a | n/a | n/a | n/a | n/a | n/a | n/a | n/a | n/a | n/a | n/a | n/a |
| 45-49 | n/a | n/a | n/a | n/a | n/a | n/a | n/a | n/a | n/a | n/a | n/a | n/a |
| 50-54 | n/a | n/a | n/a | n/a | n/a | n/a | n/a | n/a | n/a | n/a | n/a | n/a |
| 55-59 | n/a | n/a | n/a | n/a | n/a | n/a | n/a | n/a | n/a | n/a | n/a | n/a |
| 60-64 | n/a | n/a | n/a | n/a | n/a | n/a | n/a | n/a | n/a | n/a | n/a | n/a |
| 65-69 | 134 | 18.1 | 16.7 | 14.9 | 13.6 | 12.7 | 12.0 | 10.5 | 9.7 | 8.7 | 6.6 | 6.0 |
| 70-74 | 163 | 22.0 | 18.2 | 15.8 | 14.3 | 13.2 | 12.2 | 11.4 | 10.4 | 9.2 | 7.0 | 6.0 |
| 75-79 | 117 | 22.9 | 19.8 | 17.7 | 15.8 | 14.5 | 13.8 | 12.5 | 11.7 | 10.8 | 9.2 | 7.5 |
| 80-84 | 83 | 22.9 | 21.5 | 19.4 | 17.0 | 15.8 | 14.5 | 13.3 | 12.1 | 11.1 | 9.9 | 9.4 |
| 85-89 | 37 | 24.1 | 21.4 | 18.5 | 16.5 | 15.4 | 14.4 | 13.7 | 12.3 | 11.2 | 10.1 | 7.8 |
| 90-94 | n/a | n/a | n/a | n/a | n/a | n/a | n/a | n/a | n/a | n/a | n/a | n/a |
| 95-99 | n/a | n/a | n/a | n/a | n/a | n/a | n/a | n/a | n/a | n/a | n/a | n/a |
| 100+ | n/a | n/a | n/a | n/a | n/a | n/a | n/a | n/a | n/a | n/a | n/a | n/a |
| PHASE = Panel on Health and Ageing of Singaporean Elderly | | | | | | | | | | | | |

**Supplementary file 79.** Reference values for the Five-Times-Sit-to-Stand Test (FTSST) performance for males in Singapore (PHASE; pooled *n* = 451)

| **Age (years)** | ***n*** | **Percentile (s)** | | | | | | | | | | |
| --- | --- | --- | --- | --- | --- | --- | --- | --- | --- | --- | --- | --- |
|  |  | **5^th^** | **10^th^** | **20^th^** | **30^th^** | **40^th^** | **50^th^** | **60^th^** | **70^th^** | **80^th^** | **90^th^** | **95^th^** |
| 20-24 | n/a | n/a | n/a | n/a | n/a | n/a | n/a | n/a | n/a | n/a | n/a | n/a |
| 25-29 | n/a | n/a | n/a | n/a | n/a | n/a | n/a | n/a | n/a | n/a | n/a | n/a |
| 30-34 | n/a | n/a | n/a | n/a | n/a | n/a | n/a | n/a | n/a | n/a | n/a | n/a |
| 35-39 | n/a | n/a | n/a | n/a | n/a | n/a | n/a | n/a | n/a | n/a | n/a | n/a |
| 40-44 | n/a | n/a | n/a | n/a | n/a | n/a | n/a | n/a | n/a | n/a | n/a | n/a |
| 45-49 | n/a | n/a | n/a | n/a | n/a | n/a | n/a | n/a | n/a | n/a | n/a | n/a |
| 50-54 | n/a | n/a | n/a | n/a | n/a | n/a | n/a | n/a | n/a | n/a | n/a | n/a |
| 55-59 | n/a | n/a | n/a | n/a | n/a | n/a | n/a | n/a | n/a | n/a | n/a | n/a |
| 60-64 | n/a | n/a | n/a | n/a | n/a | n/a | n/a | n/a | n/a | n/a | n/a | n/a |
| 65-69 | 109 | 16.9 | 16.0 | 14.4 | 12.5 | 11.0 | 10.3 | 9.2 | 8.3 | 7.4 | 6.3 | 5.5 |
| 70-74 | 133 | 17.4 | 15.9 | 14.4 | 12.6 | 11.2 | 10.7 | 10.0 | 9.2 | 8.5 | 7.5 | 6.7 |
| 75-79 | 99 | 18.1 | 16.7 | 15.0 | 13.6 | 12.1 | 10.9 | 10.1 | 9.8 | 7.7 | 6.0 | 3.0 |
| 80-84 | 71 | 19.8 | 17.4 | 15.2 | 14.5 | 13.0 | 12.1 | 10.9 | 9.8 | 9.4 | 7.0 | 5.8 |
| 85-89 | 39 | 23.6 | 22.1 | 19.3 | 16.1 | 14.8 | 14.2 | 12.9 | 12.5 | 11.3 | 9.2 | 7.4 |
| 90-94 | n/a | n/a | n/a | n/a | n/a | n/a | n/a | n/a | n/a | n/a | n/a | n/a |
| 95-99 | n/a | n/a | n/a | n/a | n/a | n/a | n/a | n/a | n/a | n/a | n/a | n/a |
| 100+ | n/a | n/a | n/a | n/a | n/a | n/a | n/a | n/a | n/a | n/a | n/a | n/a |
| PHASE = Panel on Health and Ageing of Singaporean Elderly | | | | | | | | | | | | |

**Supplementary file 80.** Percentile curves for skeletal muscle mass (fat-free mass and lean body mass) for females in Asia (KNHANES, MPBS, PIONEER, SLHAS; pooled *n* = 27,894)


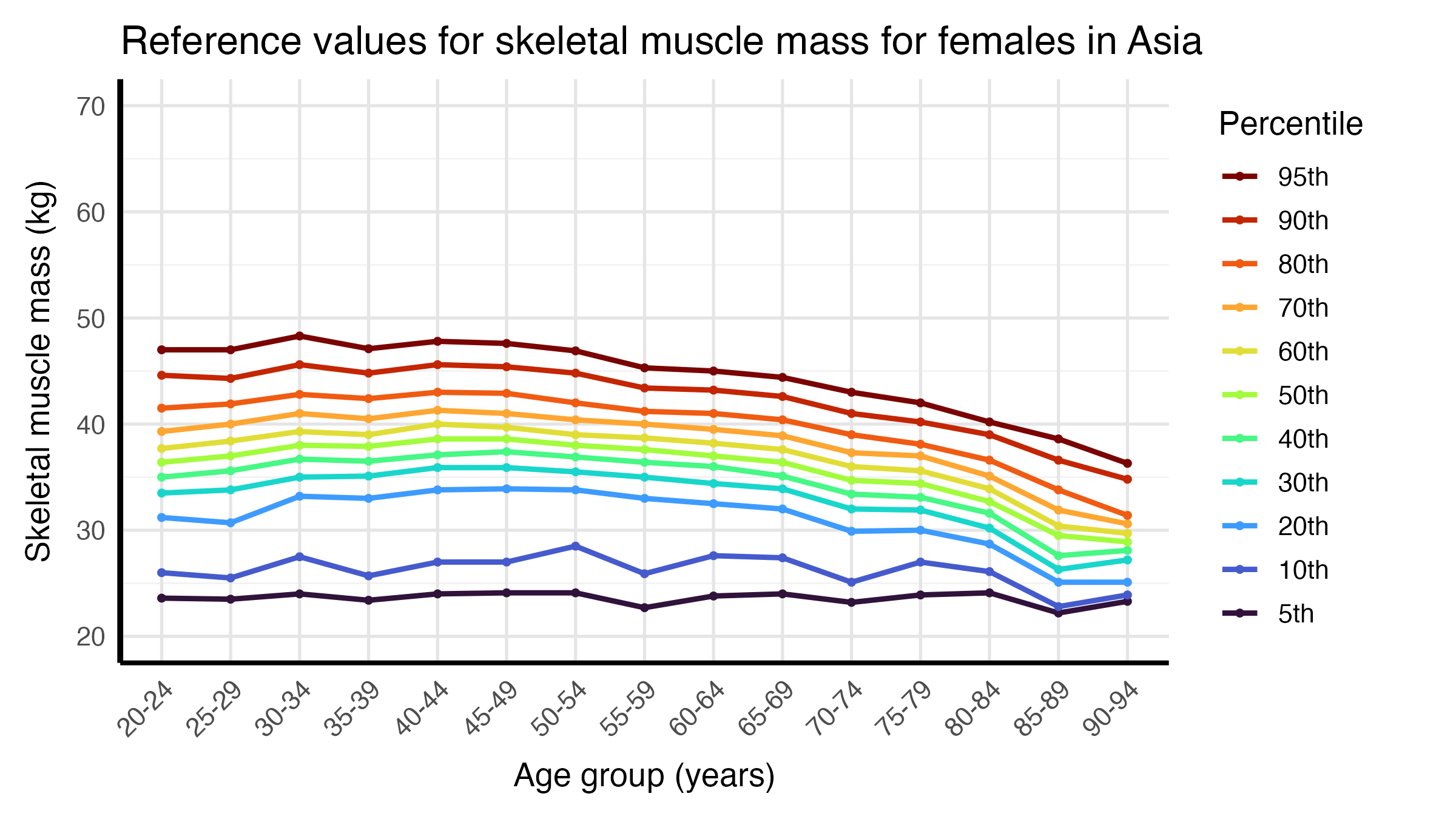


**Supplementary file 81.** Percentile curves for skeletal muscle mass (fat-free mass and lean body mass) for males in Asia (KNHANES, MPBS, PIONEER, SLHAS; pooled *n* = 21,415)


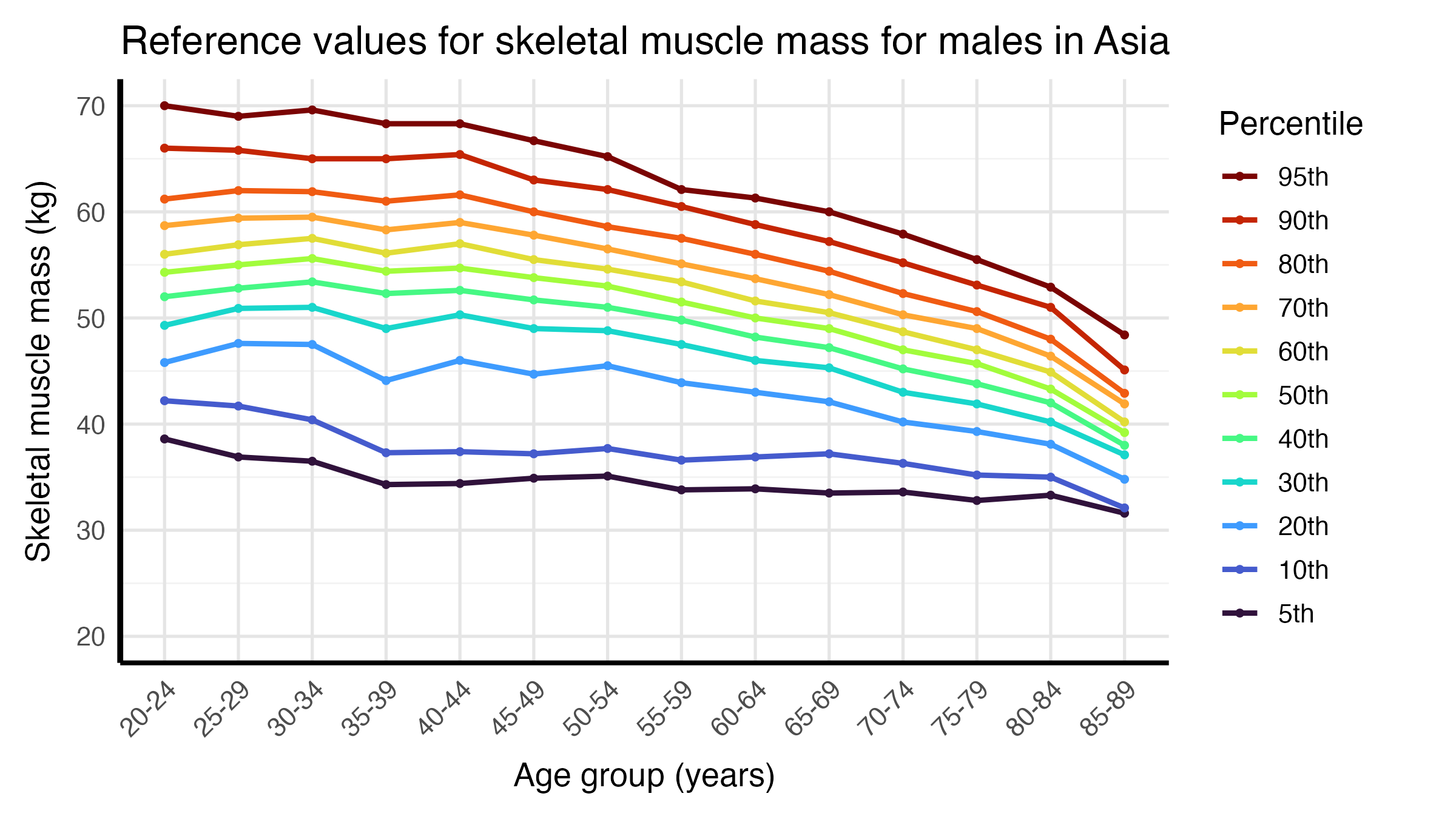


**Supplementary file 82.** Reference values for skeletal muscle mass for females in East Asia (MPBS, KNHANES; pooled *n* = 18,003)

| **Age (years)** | ***n*** | **Percentile (kg)** | | | | | | | | | | |
| --- | --- | --- | --- | --- | --- | --- | --- | --- | --- | --- | --- | --- |
|  |  | **5^th^** | **10^th^** | **20^th^** | **30^th^** | **40^th^** | **50^th^** | **60^th^** | **70^th^** | **80^th^** | **90^th^** | **95^th^** |
| 20-24 | 827 | 30.8 | 32.0 | 33.7 | 35.0 | 36.0 | 37.0 | 38.4 | 40.0 | 41.9 | 44.8 | 47.3 |
| 25-29 | 963 | 30.8 | 32.6 | 34.3 | 35.7 | 36.8 | 37.9 | 39.1 | 40.6 | 42.3 | 45.0 | 47.2 |
| 30-34 | 1,218 | 31.6 | 33.0 | 34.7 | 36.1 | 37.2 | 38.4 | 39.6 | 41.0 | 42.8 | 45.4 | 48.0 |
| 35-39 | 1,759 | 32.4 | 33.6 | 35.1 | 36.2 | 37.4 | 38.5 | 39.8 | 41.0 | 42.8 | 45.0 | 47.4 |
| 40-44 | 1,814 | 32.3 | 33.6 | 35.4 | 36.8 | 38.0 | 39.1 | 40.2 | 41.6 | 43.2 | 45.8 | 47.8 |
| 45-49 | 1,739 | 32.6 | 33.9 | 35.5 | 37.0 | 38.0 | 39.0 | 40.0 | 41.3 | 43.0 | 45.4 | 47.2 |
| 50-54 | 1,970 | 32.0 | 33.3 | 35.0 | 36.3 | 37.5 | 38.4 | 39.6 | 40.7 | 42.0 | 44.9 | 46.9 |
| 55-59 | 1,705 | 31.7 | 33.0 | 34.8 | 36.0 | 37.1 | 38.1 | 39.1 | 40.2 | 41.5 | 43.6 | 45.6 |
| 60-64 | 1,736 | 31.7 | 32.9 | 34.5 | 35.7 | 36.8 | 37.8 | 38.9 | 40.0 | 41.3 | 43.4 | 45.2 |
| 65-69 | 1,602 | 31.0 | 32.2 | 33.7 | 35.0 | 36.0 | 37.0 | 38.0 | 39.3 | 41.0 | 43.0 | 44.8 |
| 70-74 | 1,232 | 29.7 | 30.9 | 32.5 | 33.7 | 34.7 | 35.7 | 36.9 | 38.0 | 39.7 | 41.8 | 43.7 |
| 75-79 | 885 | 28.6 | 30.0 | 31.8 | 32.8 | 34.0 | 35.0 | 36.0 | 37.1 | 38.6 | 40.6 | 42.0 |
| 80-84 | 553 | 27.5 | 28.5 | 30.1 | 31.2 | 32.3 | 33.1 | 34.7 | 35.8 | 37.0 | 39.4 | 40.5 |
| 85-89 | n/a | n/a | n/a | n/a | n/a | n/a | n/a | n/a | n/a | n/a | n/a | n/a |
| 90-94 | n/a | n/a | n/a | n/a | n/a | n/a | n/a | n/a | n/a | n/a | n/a | n/a |
| 95-99 | n/a | n/a | n/a | n/a | n/a | n/a | n/a | n/a | n/a | n/a | n/a | n/a |
| 100+ | n/a | n/a | n/a | n/a | n/a | n/a | n/a | n/a | n/a | n/a | n/a | n/a |
| MPBS = Mongolia Population-Based Study; KNHANES = Korea National Health and Nutrition Examination Survey | | | | | | | | | | | | |

**Supplementary file 83.** Reference values for skeletal muscle mass for males in East Asia (MPBS, KNHANES; pooled *n* = 13,390)

| **Age (years)** | ***n*** | **Percentile (kg)** | | | | | | | | | | |
| --- | --- | --- | --- | --- | --- | --- | --- | --- | --- | --- | --- | --- |
|  |  | **5^th^** | **10^th^** | **20^th^** | **30^th^** | **40^th^** | **50^th^** | **60^th^** | **70^th^** | **80^th^** | **90^th^** | **95^th^** |
| 20-24 | 588 | 45.0 | 47.2 | 50.0 | 52.2 | 54.0 | 55.6 | 57.0 | 59.4 | 62.0 | 66.0 | 69.8 |
| 25-29 | 844 | 46.0 | 48.3 | 50.6 | 52.3 | 54.1 | 56.0 | 57.9 | 60.0 | 62.6 | 66.0 | 69.4 |
| 30-34 | 891 | 46.2 | 48.3 | 50.8 | 53.0 | 54.9 | 56.4 | 58.2 | 60.0 | 62.1 | 65.7 | 69.7 |
| 35-39 | 1,330 | 45.5 | 47.2 | 50.0 | 52.6 | 54.2 | 55.7 | 57.4 | 59.3 | 61.9 | 65.3 | 68.3 |
| 40-44 | 1,345 | 45.2 | 47.3 | 50.3 | 52.1 | 53.9 | 55.8 | 57.6 | 59.4 | 61.7 | 65.4 | 68.3 |
| 45-49 | 1,247 | 44.2 | 46.4 | 49.4 | 51.4 | 53.1 | 54.9 | 56.6 | 58.5 | 60.5 | 63.2 | 67.0 |
| 50-54 | 1,306 | 43.7 | 45.8 | 48.7 | 50.7 | 52.2 | 53.7 | 55.2 | 57.0 | 59.3 | 62.2 | 65.2 |
| 55-59 | 1,251 | 42.6 | 45.1 | 47.8 | 49.6 | 51.0 | 52.7 | 54.1 | 56.0 | 58.0 | 60.7 | 62.9 |
| 60-64 | 1,333 | 41.9 | 43.9 | 46.4 | 48.2 | 49.9 | 51.0 | 52.8 | 54.8 | 56.6 | 59.2 | 62.0 |
| 65-69 | 1,260 | 40.9 | 42.9 | 45.3 | 47.0 | 48.5 | 50.0 | 51.5 | 53.1 | 55.1 | 58.0 | 60.2 |
| 70-74 | 969 | 39.1 | 41.0 | 43.5 | 45.5 | 47.0 | 48.3 | 49.8 | 51.2 | 53.3 | 56.1 | 58.5 |
| 75-79 | 653 | 37.6 | 39.9 | 41.8 | 43.5 | 45.0 | 46.5 | 47.9 | 49.3 | 51.0 | 54.0 | 56.0 |
| 80-84 | 373 | 36.3 | 38.3 | 40.3 | 42.0 | 43.5 | 44.6 | 46.0 | 47.2 | 49.0 | 51.7 | 53.7 |
| 85-89 | n/a | n/a | n/a | n/a | n/a | n/a | n/a | n/a | n/a | n/a | n/a | n/a |
| 90-94 | n/a | n/a | n/a | n/a | n/a | n/a | n/a | n/a | n/a | n/a | n/a | n/a |
| 95-99 | n/a | n/a | n/a | n/a | n/a | n/a | n/a | n/a | n/a | n/a | n/a | n/a |
| 100+ | n/a | n/a | n/a | n/a | n/a | n/a | n/a | n/a | n/a | n/a | n/a | n/a |
| MPBS = Mongolia Population-Based Study; KNHANES = Korea National Health and Nutrition Examination Survey | | | | | | | | | | | | |

**Supplementary file 84.** Reference values for fat-free mass (bioelectrical impedance analysis) for females in Mongolia (MPBS; pooled *n* = 1,913)

| **Age (years)** | ***n*** | **Percentile (kg)** | | | | | | | | | | |
| --- | --- | --- | --- | --- | --- | --- | --- | --- | --- | --- | --- | --- |
|  |  | **5^th^** | **10^th^** | **20^th^** | **30^th^** | **40^th^** | **50^th^** | **60^th^** | **70^th^** | **80^th^** | **90^th^** | **95^th^** |
| 20-24 | n/a | n/a | n/a | n/a | n/a | n/a | n/a | n/a | n/a | n/a | n/a | n/a |
| 25-29 | n/a | n/a | n/a | n/a | n/a | n/a | n/a | n/a | n/a | n/a | n/a | n/a |
| 30-34 | 25 | 34.6 | 35.9 | 36.5 | 37.1 | 39.3 | 40.4 | 42.1 | 42.8 | 43.1 | 44.5 | 45.0 |
| 35-39 | 219 | 33.2 | 34.9 | 36.4 | 37.5 | 38.7 | 39.9 | 40.9 | 41.9 | 43.5 | 44.8 | 46.7 |
| 40-44 | 286 | 33.0 | 34.3 | 36.3 | 37.7 | 38.6 | 39.8 | 40.9 | 41.8 | 43.0 | 44.6 | 46.1 |
| 45-49 | 269 | 33.1 | 34.8 | 36.5 | 37.7 | 38.7 | 39.2 | 40.1 | 41.0 | 42.1 | 44.0 | 45.2 |
| 50-54 | 312 | 32.1 | 33.0 | 35.0 | 36.5 | 37.7 | 38.6 | 39.7 | 40.6 | 41.8 | 44.0 | 46.1 |
| 55-59 | 336 | 31.6 | 33.2 | 35.1 | 36.2 | 37.3 | 38.3 | 39.3 | 40.2 | 41.2 | 42.8 | 45.1 |
| 60-64 | 240 | 32.4 | 33.2 | 34.4 | 35.5 | 36.4 | 37.3 | 38.3 | 39.6 | 40.7 | 42.0 | 43.6 |
| 65-69 | 147 | 31.1 | 32.1 | 33.5 | 34.8 | 36.0 | 37.0 | 38.3 | 39.4 | 41.0 | 42.4 | 44.1 |
| 70-74 | 59 | 30.7 | 31.8 | 32.9 | 33.7 | 34.8 | 35.2 | 36.3 | 37.1 | 39.2 | 41.2 | 42.6 |
| 75-79 | 20 | 26.0 | 27.4 | 30.2 | 32.4 | 33.4 | 34.9 | 35.3 | 36.7 | 38.5 | 41.5 | 45.5 |
| 80-84 | n/a | n/a | n/a | n/a | n/a | n/a | n/a | n/a | n/a | n/a | n/a | n/a |
| 85-89 | n/a | n/a | n/a | n/a | n/a | n/a | n/a | n/a | n/a | n/a | n/a | n/a |
| 90-94 | n/a | n/a | n/a | n/a | n/a | n/a | n/a | n/a | n/a | n/a | n/a | n/a |
| 95-99 | n/a | n/a | n/a | n/a | n/a | n/a | n/a | n/a | n/a | n/a | n/a | n/a |
| 100+ | n/a | n/a | n/a | n/a | n/a | n/a | n/a | n/a | n/a | n/a | n/a | n/a |
| MPBS = Mongolia Population-Based Study | | | | | | | | | | | | |

**Supplementary file 85.** Reference values for fat-free mass (bioelectrical impedance analysis) for males in Mongolia (MPBS; pooled *n* = 1,231)

| **Age (years)** | ***n*** | **Percentile (kg)** | | | | | | | | | | |
| --- | --- | --- | --- | --- | --- | --- | --- | --- | --- | --- | --- | --- |
|  |  | **5^th^** | **10^th^** | **20^th^** | **30^th^** | **40^th^** | **50^th^** | **60^th^** | **70^th^** | **80^th^** | **90^th^** | **95^th^** |
| 20-24 | n/a | n/a | n/a | n/a | n/a | n/a | n/a | n/a | n/a | n/a | n/a | n/a |
| 25-29 | n/a | n/a | n/a | n/a | n/a | n/a | n/a | n/a | n/a | n/a | n/a | n/a |
| 30-34 | n/a | n/a | n/a | n/a | n/a | n/a | n/a | n/a | n/a | n/a | n/a | n/a |
| 35-39 | 140 | 42.0 | 46.1 | 49.3 | 51.7 | 54.3 | 55.6 | 57.3 | 59.4 | 61.7 | 64.6 | 68.1 |
| 40-44 | 169 | 41.5 | 44.1 | 48.8 | 51.8 | 53.8 | 55.0 | 57.1 | 59.0 | 61.1 | 64.6 | 67.3 |
| 45-49 | 181 | 44.0 | 45.4 | 48.2 | 50.2 | 52.1 | 54.4 | 55.5 | 57.4 | 60.1 | 63.9 | 66.4 |
| 50-54 | 205 | 41.7 | 43.8 | 47.1 | 49.4 | 51.5 | 53.3 | 54.9 | 57.0 | 59.0 | 61.7 | 64.0 |
| 55-59 | 198 | 41.2 | 43.6 | 46.9 | 49.4 | 51.3 | 52.9 | 55.0 | 57.0 | 59.5 | 62.1 | 64.3 |
| 60-64 | 172 | 41.8 | 43.9 | 46.9 | 48.6 | 50.1 | 51.4 | 53.4 | 55.4 | 56.9 | 60.8 | 62.2 |
| 65-69 | 110 | 41.5 | 43.1 | 46.0 | 47.5 | 48.7 | 50.5 | 51.9 | 53.8 | 57.1 | 59.6 | 62.5 |
| 70-74 | 36 | 40.7 | 43.2 | 46.1 | 47.2 | 48.1 | 50.6 | 51.0 | 52.5 | 54.6 | 56.1 | 58.3 |
| 75-79 | 20 | 42.2 | 46.2 | 47.5 | 48.3 | 50.2 | 51.6 | 52.2 | 54.0 | 56.5 | 58.0 | 59.2 |
| 80-84 | n/a | n/a | n/a | n/a | n/a | n/a | n/a | n/a | n/a | n/a | n/a | n/a |
| 85-89 | n/a | n/a | n/a | n/a | n/a | n/a | n/a | n/a | n/a | n/a | n/a | n/a |
| 90-94 | n/a | n/a | n/a | n/a | n/a | n/a | n/a | n/a | n/a | n/a | n/a | n/a |
| 95-99 | n/a | n/a | n/a | n/a | n/a | n/a | n/a | n/a | n/a | n/a | n/a | n/a |
| 100+ | n/a | n/a | n/a | n/a | n/a | n/a | n/a | n/a | n/a | n/a | n/a | n/a |
| MPBS = Mongolia Population-Based Study | | | | | | | | | | | | |

**Supplementary file 86.** Reference values for fat-free mass (bioelectrical impedance analysis) for females in the Republic of Korea (KNHANES; pooled *n* = 5,378)

| **Age (years)** | ***n*** | **Percentile (kg)** | | | | | | | | | | |
| --- | --- | --- | --- | --- | --- | --- | --- | --- | --- | --- | --- | --- |
|  |  | **5^th^** | **10^th^** | **20^th^** | **30^th^** | **40^th^** | **50^th^** | **60^th^** | **70^th^** | **80^th^** | **90^th^** | **95^th^** |
| 20-24 | 259 | 31.5 | 32.9 | 34.7 | 35.9 | 37.0 | 38.0 | 40.0 | 41.0 | 43.7 | 46.1 | 48.0 |
| 25-29 | 283 | 32.0 | 33.1 | 35.0 | 36.4 | 38.0 | 39.0 | 41.0 | 41.9 | 43.5 | 46.0 | 49.3 |
| 30-34 | 286 | 33.6 | 34.7 | 36.9 | 38.0 | 39.0 | 41.0 | 42.0 | 43.0 | 45.0 | 48.0 | 50.0 |
| 35-39 | 346 | 34.0 | 35.0 | 36.4 | 37.5 | 38.6 | 39.4 | 41.0 | 42.2 | 44.0 | 46.3 | 49.0 |
| 40-44 | 490 | 33.2 | 34.4 | 36.0 | 37.3 | 39.0 | 40.0 | 41.0 | 42.5 | 44.0 | 47.0 | 50.0 |
| 45-49 | 438 | 33.3 | 34.9 | 36.3 | 38.0 | 39.0 | 40.0 | 41.3 | 43.0 | 44.5 | 47.1 | 49.8 |
| 50-54 | 549 | 33.0 | 34.0 | 36.0 | 37.0 | 38.0 | 39.0 | 40.0 | 41.0 | 42.8 | 46.0 | 48.0 |
| 55-59 | 501 | 32.6 | 33.7 | 35.4 | 37.0 | 37.9 | 39.0 | 40.0 | 41.0 | 42.2 | 44.0 | 46.0 |
| 60-64 | 633 | 32.0 | 33.0 | 35.0 | 36.2 | 37.3 | 38.4 | 39.4 | 40.7 | 42.0 | 44.0 | 45.4 |
| 65-69 | 571 | 31.3 | 33.2 | 34.6 | 36.0 | 37.0 | 38.0 | 39.0 | 40.0 | 41.0 | 43.8 | 45.0 |
| 70-74 | 413 | 30.6 | 31.9 | 33.0 | 34.7 | 35.7 | 36.9 | 38.0 | 39.0 | 40.0 | 42.2 | 44.0 |
| 75-79 | 366 | 30.0 | 31.5 | 33.0 | 34.0 | 35.0 | 36.2 | 37.0 | 38.0 | 39.0 | 41.0 | 42.2 |
| 80-84 | 243 | 28.0 | 29.5 | 31.1 | 32.7 | 33.3 | 34.7 | 35.6 | 36.7 | 37.5 | 40.0 | 41.0 |
| 85-89 | n/a | n/a | n/a | n/a | n/a | n/a | n/a | n/a | n/a | n/a | n/a | n/a |
| 90-94 | n/a | n/a | n/a | n/a | n/a | n/a | n/a | n/a | n/a | n/a | n/a | n/a |
| 95-99 | n/a | n/a | n/a | n/a | n/a | n/a | n/a | n/a | n/a | n/a | n/a | n/a |
| 100+ | n/a | n/a | n/a | n/a | n/a | n/a | n/a | n/a | n/a | n/a | n/a | n/a |
| KNHANES = Korea National Health and Nutrition Examination Survey | | | | | | | | | | | | |

**Supplementary file 87.** Reference values for fat-free mass (bioelectrical impedance analysis) for males in the Republic of Korea (KNHANES; pooled *n* = 4,122)

| **Age (years)** | ***n*** | **Percentile (kg)** | | | | | | | | | | |
| --- | --- | --- | --- | --- | --- | --- | --- | --- | --- | --- | --- | --- |
|  |  | **5^th^** | **10^th^** | **20^th^** | **30^th^** | **40^th^** | **50^th^** | **60^th^** | **70^th^** | **80^th^** | **90^th^** | **95^th^** |
| 20-24 | 210 | 44.1 | 47.0 | 50.6 | 52.8 | 55.0 | 56.3 | 59.0 | 61.0 | 64.0 | 68.3 | 72.8 |
| 25-29 | 262 | 45.5 | 49.0 | 51.6 | 53.0 | 55.0 | 56.6 | 59.0 | 61.5 | 64.0 | 67.0 | 70.4 |
| 30-34 | 241 | 47.0 | 50.0 | 53.0 | 55.0 | 56.8 | 59.0 | 60.1 | 61.8 | 63.3 | 67.6 | 71.7 |
| 35-39 | 274 | 48.0 | 50.0 | 52.9 | 54.6 | 56.0 | 58.0 | 60.0 | 62.0 | 64.8 | 68.9 | 73.0 |
| 40-44 | 346 | 47.2 | 50.0 | 52.5 | 55.0 | 56.8 | 58.6 | 60.9 | 62.6 | 65.0 | 68.0 | 71.0 |
| 45-49 | 323 | 46.0 | 48.4 | 51.9 | 53.6 | 55.0 | 56.9 | 58.3 | 60.0 | 62.0 | 64.9 | 68.2 |
| 50-54 | 353 | 46.1 | 47.5 | 50.6 | 52.0 | 53.5 | 55.0 | 56.2 | 58.0 | 61.1 | 64.5 | 68.0 |
| 55-59 | 364 | 44.3 | 46.0 | 48.5 | 50.0 | 52.0 | 54.0 | 55.0 | 57.0 | 59.0 | 61.2 | 63.0 |
| 60-64 | 454 | 42.0 | 44.0 | 47.1 | 48.9 | 50.2 | 52.0 | 53.7 | 55.9 | 57.0 | 60.0 | 62.8 |
| 65-69 | 470 | 41.6 | 43.0 | 46.0 | 48.0 | 49.0 | 50.8 | 52.0 | 53.2 | 56.0 | 59.0 | 61.0 |
| 70-74 | 340 | 39.1 | 41.3 | 44.0 | 46.0 | 47.7 | 49.0 | 50.9 | 52.0 | 53.8 | 56.7 | 58.7 |
| 75-79 | 287 | 38.9 | 41.0 | 43.0 | 44.8 | 46.0 | 47.0 | 49.0 | 50.0 | 51.0 | 54.0 | 55.9 |
| 80-84 | 198 | 36.7 | 39.0 | 41.0 | 42.4 | 43.8 | 45.0 | 46.2 | 47.7 | 49.0 | 52.0 | 53.6 |
| 85-89 | n/a | n/a | n/a | n/a | n/a | n/a | n/a | n/a | n/a | n/a | n/a | n/a |
| 90-94 | n/a | n/a | n/a | n/a | n/a | n/a | n/a | n/a | n/a | n/a | n/a | n/a |
| 95-99 | n/a | n/a | n/a | n/a | n/a | n/a | n/a | n/a | n/a | n/a | n/a | n/a |
| 100+ | n/a | n/a | n/a | n/a | n/a | n/a | n/a | n/a | n/a | n/a | n/a | n/a |
| KNHANES = Korea National Health and Nutrition Examination Survey | | | | | | | | | | | | |

**Supplementary file 88.** Reference values for lean body mass (dual-energy x-ray absorptiometry) for females in the Republic of Korea (KNHANES; pooled *n* = 10,694)

| **Age (years)** | ***n*** | **Percentile (kg)** | | | | | | | | | | |
| --- | --- | --- | --- | --- | --- | --- | --- | --- | --- | --- | --- | --- |
|  |  | **5^th^** | **10^th^** | **20^th^** | **30^th^** | **40^th^** | **50^th^** | **60^th^** | **70^th^** | **80^th^** | **90^th^** | **95^th^** |
| 20-24 | 567 | 30.4 | 31.6 | 33.4 | 34.5 | 35.7 | 36.7 | 37.8 | 39.2 | 41.2 | 43.5 | 46.5 |
| 25-29 | 670 | 30.6 | 31.9 | 34.0 | 35.5 | 36.4 | 37.4 | 38.7 | 39.8 | 41.6 | 44.3 | 46.7 |
| 30-34 | 907 | 31.3 | 32.7 | 34.3 | 35.6 | 36.7 | 37.6 | 38.9 | 40.4 | 42.0 | 44.4 | 46.7 |
| 35-39 | 1,194 | 32.2 | 33.1 | 34.6 | 35.8 | 36.7 | 38.0 | 39.1 | 40.5 | 42.2 | 44.7 | 47.2 |
| 40-44 | 1,038 | 31.7 | 33.2 | 35.0 | 36.2 | 37.3 | 38.5 | 39.7 | 41.2 | 42.8 | 45.4 | 47.6 |
| 45-49 | 1,032 | 32.4 | 33.4 | 35.0 | 36.3 | 37.5 | 38.6 | 39.6 | 40.7 | 42.6 | 44.9 | 46.7 |
| 50-54 | 1,109 | 31.5 | 32.9 | 34.8 | 35.9 | 37.1 | 38.1 | 39.2 | 40.4 | 41.8 | 44.3 | 46.6 |
| 55-59 | 868 | 31.1 | 32.6 | 34.3 | 35.6 | 36.8 | 37.7 | 38.6 | 39.8 | 41.1 | 43.2 | 45.1 |
| 60-64 | 863 | 31.2 | 32.4 | 34.1 | 35.3 | 36.2 | 37.2 | 38.4 | 39.6 | 41.0 | 43.3 | 45.5 |
| 65-69 | 884 | 30.8 | 31.8 | 33.3 | 34.5 | 35.6 | 36.4 | 37.4 | 38.5 | 40.4 | 42.5 | 44.5 |
| 70-74 | 760 | 29.4 | 30.6 | 32.2 | 33.2 | 34.3 | 35.3 | 36.3 | 37.5 | 39.3 | 41.5 | 43.3 |
| 75-79 | 499 | 28.0 | 29.2 | 30.9 | 32.0 | 33.0 | 34.2 | 35.1 | 36.3 | 37.8 | 39.7 | 41.4 |
| 80-84 | 303 | 27.3 | 28.4 | 29.6 | 30.4 | 31.5 | 32.4 | 33.2 | 34.9 | 36.0 | 38.1 | 39.9 |
| 85-89 | n/a | n/a | n/a | n/a | n/a | n/a | n/a | n/a | n/a | n/a | n/a | n/a |
| 90-94 | n/a | n/a | n/a | n/a | n/a | n/a | n/a | n/a | n/a | n/a | n/a | n/a |
| 95-99 | n/a | n/a | n/a | n/a | n/a | n/a | n/a | n/a | n/a | n/a | n/a | n/a |
| 100+ | n/a | n/a | n/a | n/a | n/a | n/a | n/a | n/a | n/a | n/a | n/a | n/a |
| KNHANES = Korea National Health and Nutrition Examination Survey | | | | | | | | | | | | |

**Supplementary file 89.** Reference values for lean body mass (dual-energy x-ray absorptiometry) for males in the Republic of Korea (KNHANES; pooled *n* = 8,012)

| **Age (years)** | ***n*** | **Percentile (kg)** | | | | | | | | | | |
| --- | --- | --- | --- | --- | --- | --- | --- | --- | --- | --- | --- | --- |
|  |  | **5^th^** | **10^th^** | **20^th^** | **30^th^** | **40^th^** | **50^th^** | **60^th^** | **70^th^** | **80^th^** | **90^th^** | **95^th^** |
| 20-24 | 377 | 45.2 | 47.5 | 49.9 | 52.0 | 53.6 | 55.2 | 56.7 | 58.4 | 60.5 | 64.0 | 67.7 |
| 25-29 | 579 | 46.1 | 48.2 | 50.3 | 52.1 | 53.8 | 55.6 | 57.3 | 59.4 | 61.4 | 64.9 | 68.6 |
| 30-34 | 634 | 46.2 | 47.7 | 50.1 | 52.2 | 54.1 | 55.9 | 57.3 | 59.1 | 61.7 | 65.3 | 68.7 |
| 35-39 | 916 | 45.3 | 46.9 | 49.4 | 52.0 | 53.6 | 55.2 | 56.6 | 58.3 | 60.9 | 64.3 | 67.2 |
| 40-44 | 830 | 45.4 | 47.2 | 49.7 | 51.3 | 53.2 | 54.6 | 56.7 | 58.3 | 60.2 | 63.3 | 66.6 |
| 45-49 | 743 | 43.5 | 46.2 | 49.0 | 51.0 | 52.7 | 54.1 | 55.8 | 57.7 | 59.6 | 62.3 | 65.1 |
| 50-54 | 748 | 43.5 | 45.5 | 48.2 | 50.3 | 51.9 | 53.2 | 54.7 | 56.6 | 58.3 | 61.6 | 64.3 |
| 55-59 | 689 | 42.7 | 45.0 | 47.5 | 49.2 | 50.7 | 52.2 | 53.6 | 55.0 | 57.1 | 59.6 | 61.6 |
| 60-64 | 707 | 41.6 | 43.8 | 45.9 | 47.9 | 49.3 | 50.6 | 52.0 | 53.8 | 56.1 | 58.5 | 60.7 |
| 65-69 | 680 | 40.7 | 42.5 | 45.1 | 46.5 | 47.8 | 49.5 | 51.0 | 52.8 | 54.7 | 57.1 | 59.8 |
| 70-74 | 593 | 39.1 | 40.7 | 43.2 | 45.2 | 46.5 | 48.0 | 49.1 | 50.5 | 53.0 | 55.8 | 58.3 |
| 75-79 | 346 | 36.9 | 38.7 | 41.3 | 42.3 | 44.0 | 45.2 | 46.8 | 48.6 | 50.2 | 52.7 | 55.3 |
| 80-84 | 170 | 35.5 | 37.7 | 39.6 | 41.2 | 43.2 | 44.3 | 45.6 | 46.8 | 48.7 | 50.8 | 53.3 |
| 85-89 | n/a | n/a | n/a | n/a | n/a | n/a | n/a | n/a | n/a | n/a | n/a | n/a |
| 90-94 | n/a | n/a | n/a | n/a | n/a | n/a | n/a | n/a | n/a | n/a | n/a | n/a |
| 95-99 | n/a | n/a | n/a | n/a | n/a | n/a | n/a | n/a | n/a | n/a | n/a | n/a |
| 100+ | n/a | n/a | n/a | n/a | n/a | n/a | n/a | n/a | n/a | n/a | n/a | n/a |
| KNHANES = Korea National Health and Nutrition Examination Survey | | | | | | | | | | | | |

**Supplementary file 90.** Reference values for lean body mass (dual-energy x-ray absorptiometry) for females in Singapore (PIONEER; pooled *n* = 1,277)

| **Age (years)** | ***n*** | **Percentile (kg)** | | | | | | | | | | |
| --- | --- | --- | --- | --- | --- | --- | --- | --- | --- | --- | --- | --- |
|  |  | **5^th^** | **10^th^** | **20^th^** | **30^th^** | **40^th^** | **50^th^** | **60^th^** | **70^th^** | **80^th^** | **90^th^** | **95^th^** |
| 20-24 | n/a | n/a | n/a | n/a | n/a | n/a | n/a | n/a | n/a | n/a | n/a | n/a |
| 25-29 | n/a | n/a | n/a | n/a | n/a | n/a | n/a | n/a | n/a | n/a | n/a | n/a |
| 30-34 | n/a | n/a | n/a | n/a | n/a | n/a | n/a | n/a | n/a | n/a | n/a | n/a |
| 35-39 | n/a | n/a | n/a | n/a | n/a | n/a | n/a | n/a | n/a | n/a | n/a | n/a |
| 40-44 | n/a | n/a | n/a | n/a | n/a | n/a | n/a | n/a | n/a | n/a | n/a | n/a |
| 45-49 | n/a | n/a | n/a | n/a | n/a | n/a | n/a | n/a | n/a | n/a | n/a | n/a |
| 50-54 | n/a | n/a | n/a | n/a | n/a | n/a | n/a | n/a | n/a | n/a | n/a | n/a |
| 55-59 | n/a | n/a | n/a | n/a | n/a | n/a | n/a | n/a | n/a | n/a | n/a | n/a |
| 60-64 | 227 | 26.5 | 28.0 | 29.3 | 31.1 | 32.3 | 33.4 | 34.9 | 36.2 | 37.8 | 40.6 | 43.3 |
| 65-69 | 247 | 26.4 | 28.2 | 29.5 | 31.7 | 32.9 | 33.8 | 34.8 | 36.1 | 37.6 | 40.0 | 42.1 |
| 70-74 | 280 | 25.2 | 27.3 | 28.9 | 30.1 | 31.3 | 32.3 | 33.5 | 34.4 | 35.8 | 38.3 | 39.6 |
| 75-79 | 150 | 26.3 | 27.4 | 28.2 | 29.0 | 30.2 | 31.2 | 32.4 | 33.9 | 35.5 | 37.1 | 39.0 |
| 80-84 | 246 | 24.4 | 25.6 | 27.4 | 28.5 | 29.3 | 30.5 | 31.6 | 32.8 | 34.5 | 36.4 | 37.4 |
| 85-89 | 103 | 22.5 | 24.4 | 25.8 | 27.1 | 29.0 | 29.8 | 30.8 | 32.9 | 34.0 | 37.6 | 38.6 |
| 90-94 | 24 | 24.6 | 25.7 | 27.6 | 28.1 | 28.8 | 29.5 | 30.4 | 30.7 | 32.1 | 34.9 | 36.6 |
| 95-99 | n/a | n/a | n/a | n/a | n/a | n/a | n/a | n/a | n/a | n/a | n/a | n/a |
| 100+ | n/a | n/a | n/a | n/a | n/a | n/a | n/a | n/a | n/a | n/a | n/a | n/a |
| PIONEER = PopulatION HEalth and Eye Disease PRofilE in Elderly Singaporeans Study | | | | | | | | | | | | |

**Supplementary file 91.** Reference values for lean body mass (dual-energy x-ray absorptiometry) for males in Singapore (PIONEER; pooled *n* = 1,038)

| **Age (years)** | ***n*** | **Percentile (kg)** | | | | | | | | | | |
| --- | --- | --- | --- | --- | --- | --- | --- | --- | --- | --- | --- | --- |
|  |  | **5^th^** | **10^th^** | **20^th^** | **30^th^** | **40^th^** | **50^th^** | **60^th^** | **70^th^** | **80^th^** | **90^th^** | **95^th^** |
| 20-24 | n/a | n/a | n/a | n/a | n/a | n/a | n/a | n/a | n/a | n/a | n/a | n/a |
| 25-29 | n/a | n/a | n/a | n/a | n/a | n/a | n/a | n/a | n/a | n/a | n/a | n/a |
| 30-34 | n/a | n/a | n/a | n/a | n/a | n/a | n/a | n/a | n/a | n/a | n/a | n/a |
| 35-39 | n/a | n/a | n/a | n/a | n/a | n/a | n/a | n/a | n/a | n/a | n/a | n/a |
| 40-44 | n/a | n/a | n/a | n/a | n/a | n/a | n/a | n/a | n/a | n/a | n/a | n/a |
| 45-49 | n/a | n/a | n/a | n/a | n/a | n/a | n/a | n/a | n/a | n/a | n/a | n/a |
| 50-54 | n/a | n/a | n/a | n/a | n/a | n/a | n/a | n/a | n/a | n/a | n/a | n/a |
| 55-59 | n/a | n/a | n/a | n/a | n/a | n/a | n/a | n/a | n/a | n/a | n/a | n/a |
| 60-64 | 206 | 37.6 | 38.8 | 42.3 | 44.2 | 45.7 | 47.3 | 48.9 | 51.0 | 52.9 | 57.0 | 59.9 |
| 65-69 | 217 | 36.8 | 39.1 | 40.9 | 43.0 | 44.7 | 46.1 | 47.6 | 49.3 | 51.4 | 53.2 | 55.0 |
| 70-74 | 202 | 34.9 | 37.7 | 39.8 | 41.6 | 42.7 | 43.6 | 45.2 | 46.6 | 48.9 | 50.8 | 53.1 |
| 75-79 | 135 | 34.7 | 36.5 | 38.5 | 40.3 | 41.8 | 42.8 | 44.9 | 46.9 | 48.9 | 52.3 | 55.5 |
| 80-84 | 192 | 33.4 | 35.0 | 36.7 | 38.1 | 39.4 | 41.1 | 42.2 | 43.5 | 45.6 | 48.3 | 50.8 |
| 85-89 | 86 | 31.8 | 33.3 | 37.0 | 37.8 | 38.4 | 39.9 | 41.7 | 42.4 | 43.3 | 46.9 | 48.6 |
| 90-94 | n/a | n/a | n/a | n/a | n/a | n/a | n/a | n/a | n/a | n/a | n/a | n/a |
| 95-99 | n/a | n/a | n/a | n/a | n/a | n/a | n/a | n/a | n/a | n/a | n/a | n/a |
| 100+ | n/a | n/a | n/a | n/a | n/a | n/a | n/a | n/a | n/a | n/a | n/a | n/a |
| PIONEER = PopulatION HEalth and Eye Disease PRofilE in Elderly Singaporeans Study | | | | | | | | | | | | |

**Supplementary file 92.** Reference values for fat-free mass (bioelectrical impedance analysis) for females in Sri Lanka (SLHAS; pooled *n* = 3,211)

| **Age (years)** | ***n*** | **Percentile (kg)** | | | | | | | | | | |
| --- | --- | --- | --- | --- | --- | --- | --- | --- | --- | --- | --- | --- |
|  |  | **5^th^** | **10^th^** | **20^th^** | **30^th^** | **40^th^** | **50^th^** | **60^th^** | **70^th^** | **80^th^** | **90^th^** | **95^th^** |
| 20-24 | 190 | 21.1 | 21.7 | 22.5 | 23.4 | 24.2 | 24.8 | 25.5 | 26.3 | 27.4 | 28.6 | 30.0 |
| 25-29 | 256 | 21.1 | 22.1 | 22.9 | 23.6 | 24.3 | 25.0 | 25.6 | 26.3 | 27.1 | 28.3 | 29.5 |
| 30-34 | 205 | 21.3 | 22.0 | 22.9 | 23.4 | 24.0 | 24.6 | 25.5 | 26.5 | 27.4 | 29.1 | 30.5 |
| 35-39 | 356 | 21.2 | 21.6 | 22.4 | 23.2 | 23.8 | 24.5 | 25.1 | 25.8 | 26.8 | 28.4 | 29.6 |
| 40-44 | 287 | 20.9 | 21.5 | 22.5 | 23.1 | 23.6 | 24.3 | 24.9 | 25.3 | 26.0 | 27.2 | 28.4 |
| 45-49 | 290 | 21.0 | 21.5 | 22.5 | 23.1 | 23.9 | 24.6 | 25.1 | 25.8 | 26.7 | 27.9 | 29.5 |
| 50-54 | 271 | 20.2 | 20.8 | 21.7 | 22.4 | 23.2 | 24.0 | 24.6 | 25.1 | 25.8 | 26.9 | 28.1 |
| 55-59 | 294 | 19.9 | 20.5 | 21.4 | 22.1 | 22.6 | 23.1 | 23.6 | 24.4 | 25.3 | 26.5 | 27.0 |
| 60-64 | 286 | 20.6 | 21.1 | 21.9 | 22.6 | 23.2 | 23.8 | 24.2 | 25.1 | 25.9 | 27.4 | 28.3 |
| 65-69 | 277 | 20.5 | 21.5 | 22.2 | 22.9 | 23.5 | 24.1 | 24.8 | 25.4 | 26.2 | 27.3 | 28.5 |
| 70-74 | 292 | 20.0 | 20.7 | 21.8 | 22.7 | 23.3 | 24.0 | 24.6 | 25.1 | 26.1 | 27.5 | 29.0 |
| 75-79 | 142 | 19.8 | 20.8 | 21.8 | 22.5 | 23.0 | 23.7 | 24.6 | 25.4 | 26.4 | 27.9 | 28.3 |
| 80-84 | 65 | 19.7 | 20.4 | 21.5 | 22.3 | 23.0 | 23.2 | 24.0 | 24.8 | 25.4 | 26.9 | 27.7 |
| 85-89 | n/a | n/a | n/a | n/a | n/a | n/a | n/a | n/a | n/a | n/a | n/a | n/a |
| 90-94 | n/a | n/a | n/a | n/a | n/a | n/a | n/a | n/a | n/a | n/a | n/a | n/a |
| 95-99 | n/a | n/a | n/a | n/a | n/a | n/a | n/a | n/a | n/a | n/a | n/a | n/a |
| 100+ | n/a | n/a | n/a | n/a | n/a | n/a | n/a | n/a | n/a | n/a | n/a | n/a |
| SLHAS = Sri Lanka Health and Ageing Study | | | | | | | | | | | | |

**Supplementary file 93.** Reference values for fat-free mass (bioelectrical impedance analysis) for males in Sri Lanka (SLHAS; pooled *n* = 2,851)

| **Age (years)** | ***n*** | **Percentile (kg)** | | | | | | | | | | |
| --- | --- | --- | --- | --- | --- | --- | --- | --- | --- | --- | --- | --- |
|  |  | **5^th^** | **10^th^** | **20^th^** | **30^th^** | **40^th^** | **50^th^** | **60^th^** | **70^th^** | **80^th^** | **90^th^** | **95^th^** |
| 20-24 | 150 | 33.0 | 35.2 | 37.0 | 38.6 | 39.9 | 41.7 | 42.9 | 44.1 | 45.0 | 46.3 | 47.0 |
| 25-29 | 161 | 31.0 | 32.6 | 35.1 | 36.2 | 37.4 | 38.4 | 39.7 | 41.0 | 42.9 | 44.5 | 46.1 |
| 30-34 | 178 | 31.7 | 33.3 | 34.6 | 35.6 | 36.9 | 37.7 | 38.8 | 40.3 | 42.4 | 43.9 | 45.6 |
| 35-39 | 357 | 31.0 | 32.5 | 33.7 | 34.9 | 36.1 | 36.9 | 38.0 | 39.2 | 41.0 | 42.7 | 43.7 |
| 40-44 | 287 | 31.1 | 32.1 | 33.3 | 34.0 | 34.9 | 35.8 | 36.7 | 37.6 | 39.0 | 40.8 | 41.8 |
| 45-49 | 284 | 30.9 | 32.0 | 33.6 | 34.8 | 35.4 | 36.1 | 36.9 | 38.1 | 39.3 | 41.3 | 42.2 |
| 50-54 | 255 | 30.0 | 32.1 | 33.6 | 34.5 | 35.3 | 36.0 | 36.7 | 37.4 | 38.7 | 40.5 | 41.5 |
| 55-59 | 276 | 29.6 | 31.0 | 32.7 | 33.7 | 34.4 | 35.2 | 36.2 | 37.2 | 38.7 | 40.3 | 41.1 |
| 60-64 | 273 | 29.5 | 30.3 | 31.7 | 33.0 | 33.9 | 34.5 | 35.6 | 36.4 | 37.5 | 39.0 | 40.5 |
| 65-69 | 236 | 29.2 | 30.0 | 32.0 | 32.8 | 33.3 | 34.2 | 35.1 | 36.0 | 36.7 | 38.4 | 39.3 |
| 70-74 | 205 | 29.4 | 31.0 | 32.0 | 32.7 | 33.8 | 34.7 | 35.3 | 36.3 | 37.2 | 38.2 | 39.0 |
| 75-79 | 133 | 28.7 | 29.4 | 30.7 | 31.8 | 32.9 | 33.7 | 34.5 | 35.1 | 35.9 | 37.3 | 38.2 |
| 80-84 | 56 | 28.7 | 29.7 | 31.2 | 32.2 | 32.8 | 33.3 | 33.6 | 34.3 | 35.4 | 36.8 | 37.9 |
| 85-89 | n/a | n/a | n/a | n/a | n/a | n/a | n/a | n/a | n/a | n/a | n/a | n/a |
| 90-94 | n/a | n/a | n/a | n/a | n/a | n/a | n/a | n/a | n/a | n/a | n/a | n/a |
| 95-99 | n/a | n/a | n/a | n/a | n/a | n/a | n/a | n/a | n/a | n/a | n/a | n/a |
| 100+ | n/a | n/a | n/a | n/a | n/a | n/a | n/a | n/a | n/a | n/a | n/a | n/a |
| SLHAS = Sri Lanka Health and Ageing Study | | | | | | | | | | | | |

**Supplementary file 94.** Reference values for height-adjusted appendicular muscle mass (bioelectrical impedance analysis) for females in the Republic of Korea (KNHANES; pooled *n* = 5,319)

| **Age (years)** | ***n*** | **Percentile (kg/m^2^)** | | | | | | | | | | |
| --- | --- | --- | --- | --- | --- | --- | --- | --- | --- | --- | --- | --- |
|  |  | **5^th^** | **10^th^** | **20^th^** | **30^th^** | **40^th^** | **50^th^** | **60^th^** | **70^th^** | **80^th^** | **90^th^** | **95^th^** |
| 20-24 | 259 | 4.7 | 4.9 | 5.4 | 5.7 | 5.9 | 6.0 | 6.2 | 6.4 | 6.6 | 7.0 | 7.4 |
| 25-29 | 283 | 4.9 | 5.2 | 5.6 | 5.8 | 6.0 | 6.1 | 6.2 | 6.4 | 6.5 | 6.9 | 7.5 |
| 30-34 | 286 | 5.1 | 5.4 | 5.8 | 6.0 | 6.1 | 6.2 | 6.4 | 6.6 | 6.8 | 7.3 | 7.6 |
| 35-39 | 346 | 5.4 | 5.6 | 5.8 | 5.9 | 6.1 | 6.3 | 6.4 | 6.5 | 6.7 | 7.1 | 7.5 |
| 40-44 | 490 | 5.2 | 5.5 | 5.8 | 6.0 | 6.2 | 6.3 | 6.4 | 6.6 | 6.8 | 7.1 | 7.5 |
| 45-49 | 437 | 5.3 | 5.7 | 5.9 | 6.1 | 6.2 | 6.3 | 6.5 | 6.6 | 6.8 | 7.4 | 7.6 |
| 50-54 | 548 | 5.3 | 5.6 | 5.8 | 6.0 | 6.2 | 6.3 | 6.4 | 6.5 | 6.8 | 7.2 | 7.5 |
| 55-59 | 500 | 5.3 | 5.6 | 5.8 | 6.0 | 6.1 | 6.3 | 6.4 | 6.5 | 6.7 | 7.0 | 7.4 |
| 60-64 | 632 | 5.2 | 5.5 | 5.8 | 5.9 | 6.1 | 6.3 | 6.4 | 6.5 | 6.7 | 7.0 | 7.2 |
| 65-69 | 570 | 5.1 | 5.4 | 5.7 | 5.9 | 6.1 | 6.2 | 6.4 | 6.5 | 6.7 | 7.0 | 7.3 |
| 70-74 | 406 | 4.8 | 5.1 | 5.5 | 5.8 | 5.9 | 6.1 | 6.2 | 6.3 | 6.5 | 6.7 | 6.9 |
| 75-79 | 344 | 4.7 | 5.1 | 5.4 | 5.6 | 5.8 | 5.9 | 6.1 | 6.3 | 6.5 | 6.7 | 6.9 |
| 80-84 | 218 | 4.4 | 4.6 | 5.0 | 5.3 | 5.6 | 5.7 | 5.9 | 6.1 | 6.2 | 6.6 | 6.7 |
| 85-89 | n/a | n/a | n/a | n/a | n/a | n/a | n/a | n/a | n/a | n/a | n/a | n/a |
| 90-94 | n/a | n/a | n/a | n/a | n/a | n/a | n/a | n/a | n/a | n/a | n/a | n/a |
| 95-99 | n/a | n/a | n/a | n/a | n/a | n/a | n/a | n/a | n/a | n/a | n/a | n/a |
| 100+ | n/a | n/a | n/a | n/a | n/a | n/a | n/a | n/a | n/a | n/a | n/a | n/a |
| KNHANES = Korea National Health and Nutrition Examination Survey | | | | | | | | | | | | |

**Supplementary file 95.** Reference values for height-adjusted appendicular muscle mass (bioelectrical impedance analysis) for males in the Republic of Korea (KNHANES; pooled *n* = 4,102)

| **Age (years)** | ***n*** | **Percentile (kg/m^2^)** | | | | | | | | | | |
| --- | --- | --- | --- | --- | --- | --- | --- | --- | --- | --- | --- | --- |
|  |  | **5^th^** | **10^th^** | **20^th^** | **30^th^** | **40^th^** | **50^th^** | **60^th^** | **70^th^** | **80^th^** | **90^th^** | **95^th^** |
| 20-24 | 210 | 6.5 | 6.9 | 7.3 | 7.6 | 7.7 | 7.8 | 8.0 | 8.4 | 8.7 | 9.2 | 9.6 |
| 25-29 | 262 | 6.8 | 7.0 | 7.4 | 7.6 | 7.8 | 7.9 | 8.1 | 8.4 | 8.7 | 9.0 | 9.3 |
| 30-34 | 241 | 6.9 | 7.2 | 7.6 | 7.7 | 7.9 | 8.1 | 8.4 | 8.6 | 8.8 | 9.2 | 9.5 |
| 35-39 | 274 | 7.0 | 7.3 | 7.6 | 7.8 | 7.9 | 8.1 | 8.3 | 8.6 | 8.9 | 9.2 | 9.5 |
| 40-44 | 345 | 7.1 | 7.4 | 7.6 | 7.8 | 7.9 | 8.2 | 8.4 | 8.7 | 8.9 | 9.2 | 9.5 |
| 45-49 | 323 | 6.9 | 7.3 | 7.6 | 7.8 | 7.9 | 8.1 | 8.3 | 8.5 | 8.7 | 9.0 | 9.3 |
| 50-54 | 353 | 7.0 | 7.2 | 7.5 | 7.7 | 7.8 | 8.0 | 8.1 | 8.4 | 8.7 | 9.0 | 9.4 |
| 55-59 | 364 | 6.8 | 7.0 | 7.4 | 7.5 | 7.7 | 7.8 | 8.0 | 8.2 | 8.4 | 8.8 | 9.1 |
| 60-64 | 452 | 6.4 | 6.7 | 7.1 | 7.3 | 7.5 | 7.7 | 7.9 | 8.0 | 8.2 | 8.6 | 8.9 |
| 65-69 | 468 | 6.5 | 6.7 | 7.1 | 7.3 | 7.5 | 7.6 | 7.8 | 7.9 | 8.2 | 8.5 | 8.7 |
| 70-74 | 337 | 6.1 | 6.3 | 6.7 | 7.0 | 7.2 | 7.4 | 7.6 | 7.7 | 8.0 | 8.3 | 8.6 |
| 75-79 | 283 | 6.0 | 6.4 | 6.6 | 6.8 | 7.1 | 7.2 | 7.4 | 7.6 | 7.8 | 8.0 | 8.3 |
| 80-84 | 190 | 5.9 | 6.1 | 6.3 | 6.5 | 6.7 | 6.9 | 7.1 | 7.3 | 7.6 | 7.9 | 8.1 |
| 85-89 | n/a | n/a | n/a | n/a | n/a | n/a | n/a | n/a | n/a | n/a | n/a | n/a |
| 90-94 | n/a | n/a | n/a | n/a | n/a | n/a | n/a | n/a | n/a | n/a | n/a | n/a |
| 95-99 | n/a | n/a | n/a | n/a | n/a | n/a | n/a | n/a | n/a | n/a | n/a | n/a |
| 100+ | n/a | n/a | n/a | n/a | n/a | n/a | n/a | n/a | n/a | n/a | n/a | n/a |
| KNHANES = Korea National Health and Nutrition Examination Survey | | | | | | | | | | | | |

**Supplementary file 96.** Reference values for height-adjusted appendicular muscle mass (dual-energy x-ray absorptiometry) for females in the Republic of Korea (KNHANES; pooled *n* = 8,659)

| **Age (years)** | ***n*** | **Percentile (kg/m^2^)** | | | | | | | | | | |
| --- | --- | --- | --- | --- | --- | --- | --- | --- | --- | --- | --- | --- |
|  |  | **5^th^** | **10^th^** | **20^th^** | **30^th^** | **40^th^** | **50^th^** | **60^th^** | **70^th^** | **80^th^** | **90^th^** | **95^th^** |
| 20-24 | 476 | 4.5 | 4.8 | 5.1 | 5.5 | 5.9 | 6.2 | 6.7 | 7.2 | 8.3 | 10.0 | 11.3 |
| 25-29 | 576 | 4.6 | 4.9 | 5.4 | 5.7 | 6.0 | 6.4 | 6.7 | 7.3 | 8.6 | 10.2 | 11.6 |
| 30-34 | 741 | 4.6 | 4.9 | 5.3 | 5.7 | 6.0 | 6.3 | 6.7 | 7.2 | 7.9 | 10.0 | 11.3 |
| 35-39 | 938 | 4.5 | 4.8 | 5.2 | 5.6 | 5.9 | 6.2 | 6.6 | 7.1 | 7.9 | 9.5 | 10.8 |
| 40-44 | 823 | 4.6 | 5.0 | 5.4 | 5.7 | 6.0 | 6.4 | 6.8 | 7.2 | 7.9 | 9.5 | 10.7 |
| 45-49 | 812 | 4.6 | 4.9 | 5.4 | 5.7 | 6.0 | 6.4 | 6.7 | 7.3 | 8.1 | 9.5 | 10.5 |
| 50-54 | 889 | 4.5 | 4.8 | 5.2 | 5.6 | 5.9 | 6.2 | 6.5 | 7.0 | 7.6 | 9.1 | 10.3 |
| 55-59 | 727 | 4.4 | 4.7 | 5.1 | 5.5 | 5.9 | 6.1 | 6.5 | 6.9 | 7.6 | 8.9 | 9.9 |
| 60-64 | 719 | 4.4 | 4.7 | 5.2 | 5.5 | 5.8 | 6.1 | 6.4 | 6.8 | 7.5 | 8.9 | 9.7 |
| 65-69 | 714 | 4.3 | 4.6 | 5.0 | 5.3 | 5.6 | 5.9 | 6.3 | 6.7 | 7.3 | 8.5 | 9.4 |
| 70-74 | 599 | 4.2 | 4.4 | 4.8 | 5.1 | 5.3 | 5.6 | 6.0 | 6.4 | 7.0 | 8.0 | 9.1 |
| 75-79 | 398 | 4.0 | 4.3 | 4.7 | 4.9 | 5.2 | 5.5 | 5.7 | 6.0 | 6.6 | 7.5 | 8.1 |
| 80-84 | 247 | 3.7 | 4.0 | 4.3 | 4.7 | 4.9 | 5.2 | 5.5 | 5.8 | 6.3 | 7.2 | 8.0 |
| 85-89 | n/a | n/a | n/a | n/a | n/a | n/a | n/a | n/a | n/a | n/a | n/a | n/a |
| 90-94 | n/a | n/a | n/a | n/a | n/a | n/a | n/a | n/a | n/a | n/a | n/a | n/a |
| 95-99 | n/a | n/a | n/a | n/a | n/a | n/a | n/a | n/a | n/a | n/a | n/a | n/a |
| 100+ | n/a | n/a | n/a | n/a | n/a | n/a | n/a | n/a | n/a | n/a | n/a | n/a |
| KNHANES = Korea National Health and Nutrition Examination Survey | | | | | | | | | | | | |

**Supplementary file 97.** Reference values for height-adjusted appendicular muscle mass (dual-energy x-ray absorptiometry) for males in the Republic of Korea (KNHANES; pooled *n* = 6,542)

| **Age (years)** | ***n*** | **Percentile (kg/m^2^)** | | | | | | | | | | |
| --- | --- | --- | --- | --- | --- | --- | --- | --- | --- | --- | --- | --- |
|  |  | **5^th^** | **10^th^** | **20^th^** | **30^th^** | **40^th^** | **50^th^** | **60^th^** | **70^th^** | **80^th^** | **90^th^** | **95^th^** |
| 20-24 | 289 | 4.6 | 5.1 | 5.8 | 7.2 | 7.8 | 8.4 | 9.2 | 9.8 | 10.3 | 11.3 | 12.3 |
| 25-29 | 460 | 5.0 | 5.4 | 6.5 | 7.6 | 8.2 | 8.8 | 9.4 | 9.9 | 10.6 | 11.2 | 11.9 |
| 30-34 | 516 | 4.7 | 5.3 | 6.1 | 7.4 | 8.1 | 8.6 | 9.2 | 9.7 | 10.3 | 11.3 | 12.0 |
| 35-39 | 759 | 4.7 | 5.4 | 6.5 | 7.5 | 8.1 | 8.6 | 9.1 | 9.5 | 10.1 | 11.0 | 11.8 |
| 40-44 | 704 | 4.9 | 5.4 | 6.6 | 7.4 | 8.0 | 8.5 | 9.0 | 9.6 | 10.1 | 11.1 | 11.8 |
| 45-49 | 613 | 4.9 | 5.4 | 6.1 | 6.9 | 7.5 | 8.0 | 8.6 | 9.3 | 9.9 | 10.8 | 11.3 |
| 50-54 | 614 | 4.8 | 5.2 | 6.1 | 7.0 | 7.7 | 8.2 | 8.6 | 9.1 | 9.6 | 10.3 | 11.1 |
| 55-59 | 547 | 4.9 | 5.4 | 6.5 | 7.1 | 7.6 | 8.1 | 8.6 | 8.9 | 9.4 | 10.0 | 10.8 |
| 60-64 | 567 | 4.8 | 5.1 | 6.3 | 6.8 | 7.3 | 7.7 | 8.1 | 8.6 | 9.3 | 9.9 | 10.5 |
| 65-69 | 557 | 4.7 | 5.1 | 5.9 | 6.5 | 7.0 | 7.5 | 8.0 | 8.5 | 9.0 | 9.7 | 10.7 |
| 70-74 | 478 | 4.5 | 5.1 | 5.9 | 6.4 | 7.0 | 7.5 | 7.7 | 8.1 | 8.7 | 9.4 | 10.1 |
| 75-79 | 294 | 4.0 | 4.4 | 5.0 | 5.7 | 6.3 | 6.8 | 7.1 | 7.4 | 8.0 | 8.8 | 9.3 |
| 80-84 | 144 | 3.9 | 4.1 | 4.9 | 5.5 | 6.0 | 6.4 | 6.9 | 7.3 | 8.0 | 8.6 | 9.3 |
| 85-89 | n/a | n/a | n/a | n/a | n/a | n/a | n/a | n/a | n/a | n/a | n/a | n/a |
| 90-94 | n/a | n/a | n/a | n/a | n/a | n/a | n/a | n/a | n/a | n/a | n/a | n/a |
| 95-99 | n/a | n/a | n/a | n/a | n/a | n/a | n/a | n/a | n/a | n/a | n/a | n/a |
| 100+ | n/a | n/a | n/a | n/a | n/a | n/a | n/a | n/a | n/a | n/a | n/a | n/a |
| KNHANES = Korea National Health and Nutrition Examination Survey | | | | | | | | | | | | |

**Supplementary file 98.** Percentile curves for calf circumference for females in Asia (CLHLS, LASI DAD; pooled *n* = 10,859)


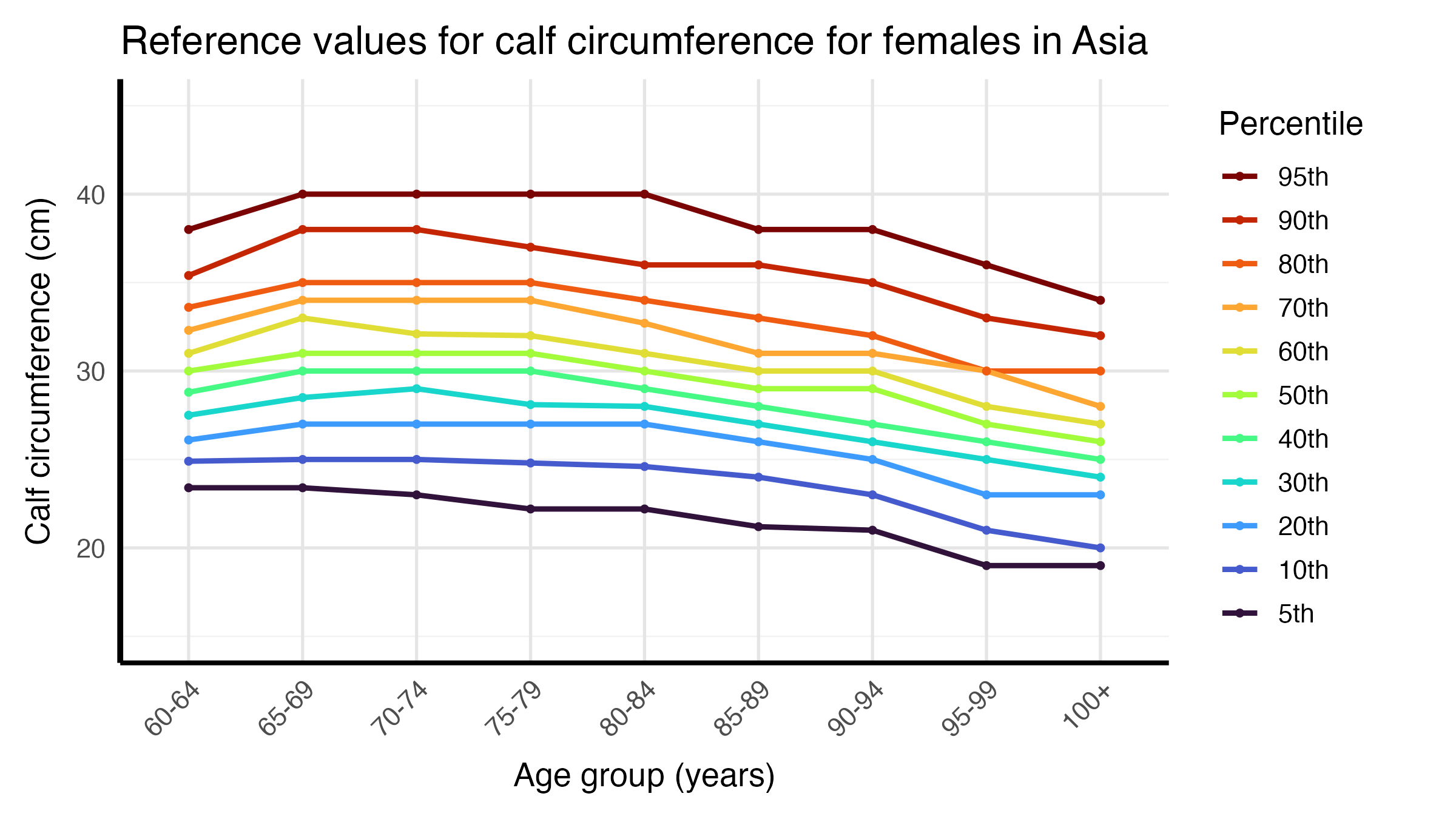


**Supplementary file 99.** Percentile curves for calf circumference for males in Asia (CLHLS, LASI DAD; pooled *n* = 8,606)


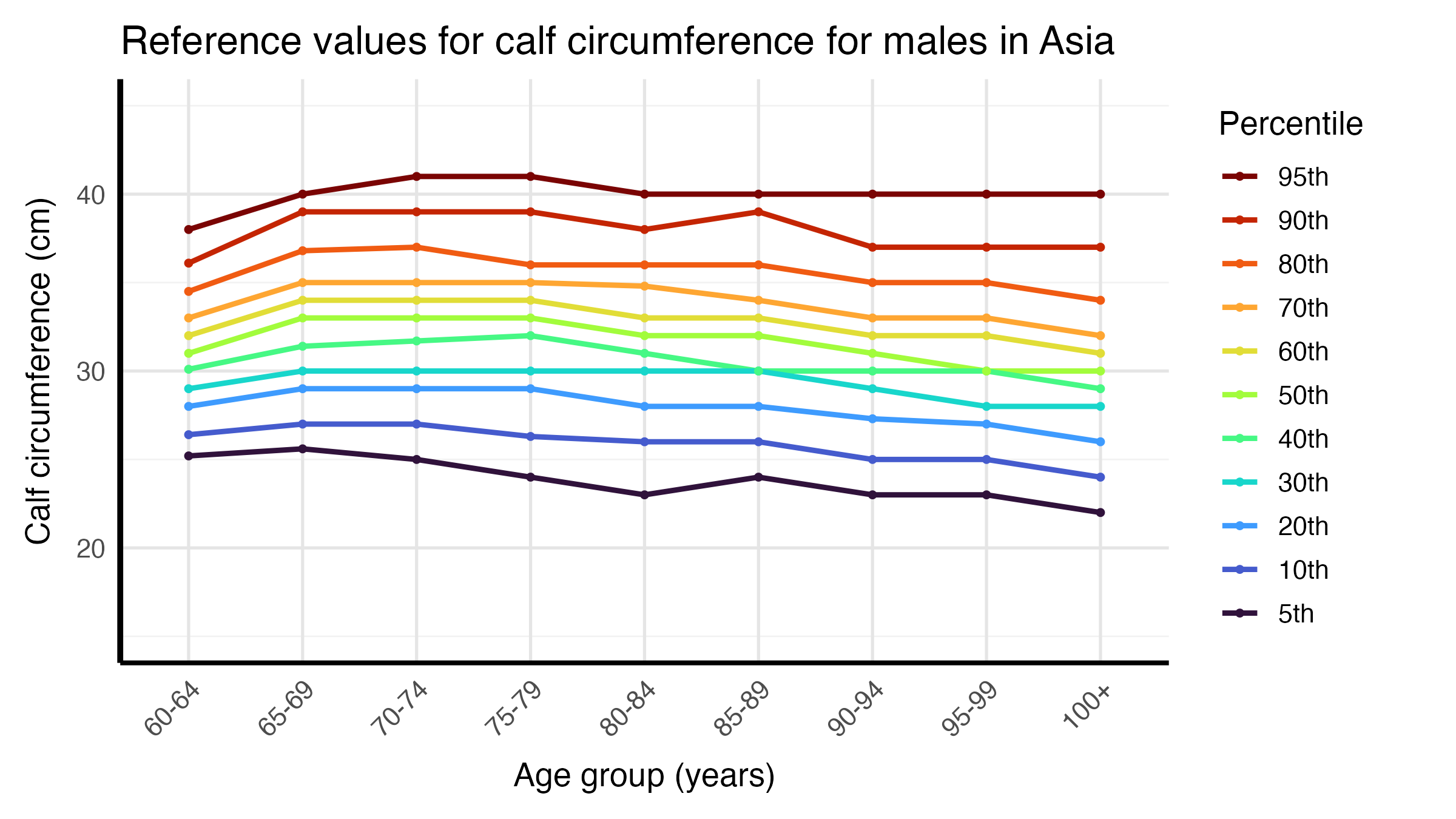


**Supplementary file 100.** Reference values for calf circumference for females in China (CLHLS; pooled *n* = 8,685)

| **Age (years)** | ***n*** | **Percentile (cm)** | | | | | | | | | | |
| --- | --- | --- | --- | --- | --- | --- | --- | --- | --- | --- | --- | --- |
|  |  | **5^th^** | **10^th^** | **20^th^** | **30^th^** | **40^th^** | **50^th^** | **60^th^** | **70^th^** | **80^th^** | **90^th^** | **95^th^** |
| 20-24 | n/a | n/a | n/a | n/a | n/a | n/a | n/a | n/a | n/a | n/a | n/a | n/a |
| 25-29 | n/a | n/a | n/a | n/a | n/a | n/a | n/a | n/a | n/a | n/a | n/a | n/a |
| 30-34 | n/a | n/a | n/a | n/a | n/a | n/a | n/a | n/a | n/a | n/a | n/a | n/a |
| 35-39 | n/a | n/a | n/a | n/a | n/a | n/a | n/a | n/a | n/a | n/a | n/a | n/a |
| 40-44 | n/a | n/a | n/a | n/a | n/a | n/a | n/a | n/a | n/a | n/a | n/a | n/a |
| 45-49 | n/a | n/a | n/a | n/a | n/a | n/a | n/a | n/a | n/a | n/a | n/a | n/a |
| 50-54 | n/a | n/a | n/a | n/a | n/a | n/a | n/a | n/a | n/a | n/a | n/a | n/a |
| 55-59 | n/a | n/a | n/a | n/a | n/a | n/a | n/a | n/a | n/a | n/a | n/a | n/a |
| 60-64 | 38 | 24.6 | 26.0 | 30.4 | 31.1 | 32.8 | 33.0 | 34.2 | 35.9 | 38.6 | 41.0 | 41.2 |
| 65-69 | 755 | 25.0 | 28.0 | 30.0 | 31.0 | 32.0 | 33.0 | 34.0 | 35.0 | 37.0 | 39.0 | 40.3 |
| 70-74 | 805 | 23.2 | 27.0 | 29.0 | 30.0 | 32.0 | 33.0 | 33.0 | 35.0 | 36.0 | 38.0 | 40.8 |
| 75-79 | 1,037 | 24.0 | 26.0 | 28.0 | 30.0 | 31.0 | 32.0 | 33.0 | 34.0 | 35.0 | 37.0 | 40.0 |
| 80-84 | 1,112 | 23.0 | 25.0 | 27.0 | 29.0 | 30.0 | 30.0 | 32.0 | 33.0 | 34.0 | 37.0 | 40.0 |
| 85-89 | 947 | 21.3 | 24.0 | 26.0 | 27.8 | 29.0 | 30.0 | 30.0 | 32.0 | 33.0 | 36.0 | 38.0 |
| 90-94 | 1,138 | 21.0 | 23.0 | 25.0 | 26.0 | 28.0 | 29.0 | 30.0 | 31.0 | 32.0 | 35.0 | 38.0 |
| 95-99 | 770 | 19.0 | 21.0 | 23.0 | 25.0 | 26.0 | 27.0 | 28.0 | 30.0 | 31.0 | 33.0 | 36.0 |
| 100+ | 2,083 | 19.0 | 20.0 | 23.0 | 24.0 | 25.0 | 26.0 | 27.0 | 28.0 | 30.0 | 32.0 | 34.0 |
| CLHLS = Chinese Longitudinal Health and Longevity Survey | | | | | | | | | | | | |

**Supplementary file 101.** Reference values for calf circumference for males in China (CLHLS; pooled *n* = 6,744)

| **Age (years)** | ***n*** | **Percentile (cm)** | | | | | | | | | | |
| --- | --- | --- | --- | --- | --- | --- | --- | --- | --- | --- | --- | --- |
|  |  | **5^th^** | **10^th^** | **20^th^** | **30^th^** | **40^th^** | **50^th^** | **60^th^** | **70^th^** | **80^th^** | **90^th^** | **95^th^** |
| 20-24 | n/a | n/a | n/a | n/a | n/a | n/a | n/a | n/a | n/a | n/a | n/a | n/a |
| 25-29 | n/a | n/a | n/a | n/a | n/a | n/a | n/a | n/a | n/a | n/a | n/a | n/a |
| 30-34 | n/a | n/a | n/a | n/a | n/a | n/a | n/a | n/a | n/a | n/a | n/a | n/a |
| 35-39 | n/a | n/a | n/a | n/a | n/a | n/a | n/a | n/a | n/a | n/a | n/a | n/a |
| 40-44 | n/a | n/a | n/a | n/a | n/a | n/a | n/a | n/a | n/a | n/a | n/a | n/a |
| 45-49 | n/a | n/a | n/a | n/a | n/a | n/a | n/a | n/a | n/a | n/a | n/a | n/a |
| 50-54 | n/a | n/a | n/a | n/a | n/a | n/a | n/a | n/a | n/a | n/a | n/a | n/a |
| 55-59 | n/a | n/a | n/a | n/a | n/a | n/a | n/a | n/a | n/a | n/a | n/a | n/a |
| 60-64 | 42 | 24.1 | 28.1 | 30.2 | 31.3 | 32.0 | 33.0 | 34.0 | 35.0 | 36.8 | 39.0 | 40.0 |
| 65-69 | 752 | 28.0 | 30.0 | 31.0 | 32.0 | 33.0 | 35.0 | 36.0 | 37.0 | 38.0 | 40.0 | 42.4 |
| 70-74 | 914 | 26.0 | 29.0 | 30.0 | 32.0 | 33.0 | 34.0 | 35.0 | 36.0 | 38.0 | 40.0 | 42.0 |
| 75-79 | 983 | 24.0 | 28.0 | 30.0 | 31.0 | 32.0 | 34.0 | 35.0 | 36.0 | 37.0 | 40.0 | 42.0 |
| 80-84 | 1,044 | 24.0 | 26.3 | 29.0 | 30.0 | 31.0 | 32.0 | 34.0 | 35.0 | 36.0 | 39.0 | 40.0 |
| 85-89 | 813 | 24.0 | 27.0 | 29.0 | 30.0 | 31.0 | 32.0 | 33.0 | 35.0 | 36.0 | 39.0 | 40.0 |
| 90-94 | 969 | 23.0 | 25.0 | 28.0 | 29.0 | 30.0 | 31.0 | 32.0 | 34.0 | 35.0 | 37.0 | 40.0 |
| 95-99 | 556 | 23.0 | 25.0 | 27.0 | 28.0 | 30.0 | 30.0 | 32.0 | 33.0 | 35.0 | 37.0 | 40.0 |
| 100+ | 671 | 22.0 | 24.0 | 26.0 | 28.0 | 29.0 | 30.0 | 31.0 | 32.0 | 34.0 | 37.0 | 40.0 |
| CLHLS = Chinese Longitudinal Health and Longevity Survey | | | | | | | | | | | | |

**Supplementary file 102.** Reference values for calf circumference for females in India (LASI DAD; pooled *n* = 2,154)

| **Age (years)** | ***n*** | **Percentile (cm)** | | | | | | | | | | |
| --- | --- | --- | --- | --- | --- | --- | --- | --- | --- | --- | --- | --- |
|  |  | **5^th^** | **10^th^** | **20^th^** | **30^th^** | **40^th^** | **50^th^** | **60^th^** | **70^th^** | **80^th^** | **90^th^** | **95^th^** |
| 20-24 | n/a | n/a | n/a | n/a | n/a | n/a | n/a | n/a | n/a | n/a | n/a | n/a |
| 25-29 | n/a | n/a | n/a | n/a | n/a | n/a | n/a | n/a | n/a | n/a | n/a | n/a |
| 30-34 | n/a | n/a | n/a | n/a | n/a | n/a | n/a | n/a | n/a | n/a | n/a | n/a |
| 35-39 | n/a | n/a | n/a | n/a | n/a | n/a | n/a | n/a | n/a | n/a | n/a | n/a |
| 40-44 | n/a | n/a | n/a | n/a | n/a | n/a | n/a | n/a | n/a | n/a | n/a | n/a |
| 45-49 | n/a | n/a | n/a | n/a | n/a | n/a | n/a | n/a | n/a | n/a | n/a | n/a |
| 50-54 | n/a | n/a | n/a | n/a | n/a | n/a | n/a | n/a | n/a | n/a | n/a | n/a |
| 55-59 | n/a | n/a | n/a | n/a | n/a | n/a | n/a | n/a | n/a | n/a | n/a | n/a |
| 60-64 | 668 | 23.4 | 24.8 | 26.1 | 27.3 | 28.5 | 29.5 | 31.0 | 32.0 | 33.4 | 35.0 | 37.3 |
| 65-69 | 644 | 23.0 | 24.0 | 25.3 | 26.5 | 27.4 | 28.7 | 30.0 | 31.2 | 33.0 | 35.0 | 36.6 |
| 70-74 | 378 | 23.0 | 24.0 | 25.2 | 26.3 | 27.0 | 28.1 | 29.0 | 30.5 | 32.0 | 33.9 | 35.8 |
| 75-79 | 242 | 21.8 | 22.2 | 24.0 | 25.0 | 25.9 | 27.0 | 27.9 | 28.5 | 30.0 | 32.5 | 34.2 |
| 80-84 | 125 | 20.4 | 21.8 | 23.9 | 25.0 | 25.9 | 26.6 | 27.3 | 28.0 | 30.2 | 32.1 | 33.8 |
| 85-89 | 64 | 21.3 | 22.4 | 23.3 | 24.4 | 25.2 | 27.0 | 27.6 | 28.0 | 29.1 | 31.5 | 34.6 |
| 90-94 | 33 | 19.7 | 21.0 | 21.7 | 23.4 | 24.9 | 25.3 | 26.0 | 26.0 | 27.2 | 29.1 | 30.1 |
| 95-99 | n/a | n/a | n/a | n/a | n/a | n/a | n/a | n/a | n/a | n/a | n/a | n/a |
| 100+ | n/a | n/a | n/a | n/a | n/a | n/a | n/a | n/a | n/a | n/a | n/a | n/a |
| LASI DAD = Longitudinal Aging Study in India-Diagnostic Assessment of Dementia | | | | | | | | | | | | |

**Supplementary file 103.** Reference values for calf circumference for males in India (LASI DAD; pooled *n* = 1,853)

| **Age (years)** | ***n*** | **Percentile (cm)** | | | | | | | | | | |
| --- | --- | --- | --- | --- | --- | --- | --- | --- | --- | --- | --- | --- |
|  |  | **5^th^** | **10^th^** | **20^th^** | **30^th^** | **40^th^** | **50^th^** | **60^th^** | **70^th^** | **80^th^** | **90^th^** | **95^th^** |
| 20-24 | n/a | n/a | n/a | n/a | n/a | n/a | n/a | n/a | n/a | n/a | n/a | n/a |
| 25-29 | n/a | n/a | n/a | n/a | n/a | n/a | n/a | n/a | n/a | n/a | n/a | n/a |
| 30-34 | n/a | n/a | n/a | n/a | n/a | n/a | n/a | n/a | n/a | n/a | n/a | n/a |
| 35-39 | n/a | n/a | n/a | n/a | n/a | n/a | n/a | n/a | n/a | n/a | n/a | n/a |
| 40-44 | n/a | n/a | n/a | n/a | n/a | n/a | n/a | n/a | n/a | n/a | n/a | n/a |
| 45-49 | n/a | n/a | n/a | n/a | n/a | n/a | n/a | n/a | n/a | n/a | n/a | n/a |
| 50-54 | n/a | n/a | n/a | n/a | n/a | n/a | n/a | n/a | n/a | n/a | n/a | n/a |
| 55-59 | n/a | n/a | n/a | n/a | n/a | n/a | n/a | n/a | n/a | n/a | n/a | n/a |
| 60-64 | 443 | 25.3 | 26.4 | 27.8 | 29.0 | 30.0 | 31.0 | 32.0 | 33.0 | 34.3 | 36.0 | 37.1 |
| 65-69 | 578 | 24.9 | 26.0 | 27.3 | 28.3 | 29.7 | 30.3 | 31.3 | 32.3 | 33.6 | 35.2 | 37.0 |
| 70-74 | 382 | 24.0 | 25.0 | 27.0 | 28.0 | 29.0 | 29.8 | 30.5 | 31.5 | 32.8 | 34.3 | 35.5 |
| 75-79 | 228 | 24.2 | 25.2 | 26.6 | 27.5 | 28.3 | 29.3 | 30.4 | 31.6 | 32.9 | 34.0 | 34.8 |
| 80-84 | 136 | 22.8 | 24.5 | 25.5 | 26.2 | 27.4 | 29.0 | 29.4 | 30.3 | 31.3 | 33.7 | 35.0 |
| 85-89 | 66 | 23.9 | 24.2 | 25.5 | 26.3 | 27.0 | 28.0 | 28.5 | 30.3 | 32.0 | 33.9 | 35.8 |
| 90-94 | 20 | 23.5 | 24.0 | 24.3 | 25.0 | 25.3 | 26.2 | 27.2 | 28.0 | 28.0 | 29.0 | 29.2 |
| 95-99 | n/a | n/a | n/a | n/a | n/a | n/a | n/a | n/a | n/a | n/a | n/a | n/a |
| 100+ | n/a | n/a | n/a | n/a | n/a | n/a | n/a | n/a | n/a | n/a | n/a | n/a |
| LASI DAD = Longitudinal Aging Study in India-Diagnostic Assessment of Dementia | | | | | | | | | | | | |

**Supplementary file 104.** Country-specific 50^th^ percentile for handgrip strength (kg)

**
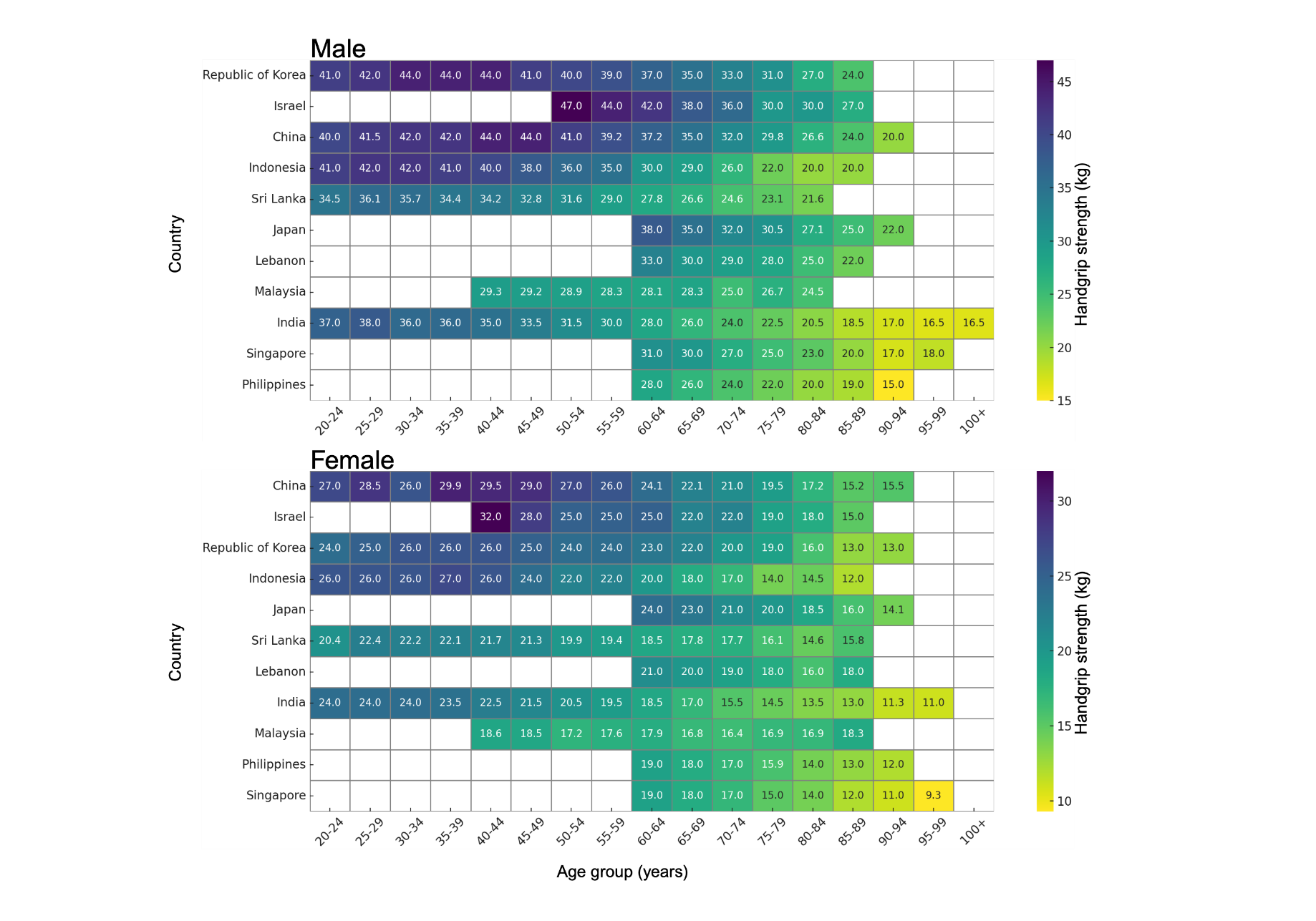
**

**Supplementary references**

S1. Zhao Y, Hu Y, Smith JP, Strauss J, Yang G. Cohort profile: the China Health and Retirement Longitudinal Study (CHARLS). *Int J Epidemiol* 2014; **43:** 61-8.

S2. Zeng Y. Towards Deeper Research and Better Policy for Healthy Aging -- Using the Unique Data of Chinese Longitudinal Healthy Longevity Survey. *China Economic J* 2012; **5:** 131-149.

S3. Kowal P, Chatterji S, Naidoo N, et al. Data resource profile: the World Health Organization Study on global AGEing and adult health (SAGE). *Int J Epidemiol*. 2012; **41:** 1639-49.

S4. Perianayagam A, Bloom D, Lee J, et al. Cohort Profile: The Longitudinal Ageing Study in India (LASI). *Int J Epidemiol* 2022; **51:** e167-e76.

S5. Strauss J, Witoelar F, Sikoki B. The fifth wave of the Indonesia family life survey: overview and field report; 2016.

S6. Börsch-Supan A, Brandt M, Hunkler C, et al. Data Resource Profile: the Survey of Health, Ageing and Retirement in Europe (SHARE). *Int J Epidemiol*. 2013; **42:** 992-1001.

S7. National Survey of the Japanese Elderly <Wave8>, 2012. Available online: <https://ssjda.iss.u-tokyo.ac.jp/Direct/gaiyo.php?eid=1476>. Accessed on 7^th^ July, 2025.

S8. The Nihon University Japanese Longitudinal Study of Aging (NUJLSOA) – History and New Directions. Available online: <https://iafor.org/the-nihon-university-japanese-longitudinal-study-of-aging-nujlsoa-history-and-new-directions-yasuhiko-saito/>. Accessed on 7^th^ July, 2025.

S9. Lebanon Study on Aging and Health (LSAH). Available online: <https://sites.aub.edu.lb/lsaha/>. Accessed on 7^th^ July, 2025.

S10. Social Wellbeing Research Centre. Malaysia ageing and retirement survey (MARS) wave 1–2018/2019: FULL REPORT. Kuala Lumpur: Social Wellbeing Research Centre (SWRC); 2021.

S11. Dayan A, Erkhembayar R, Luvsandavaajav O, Mukhtar Y, Enkhtuvshin B, Tumenbayar B. Prevalence of Type 2 Diabetes in Mongolia: Results from Population-Based Survey Compared with 1999 Study. *Diabetes Metab Syndr Obes* 2023; **16:** 1833-46.

S12. Cruz GT, Saito Y, Cruz CJP, Paguirigan MRB. (2019). The 2018 longitudinal study of ageing and health in The Philippines. In GT Cruz, CJP Cruz, & Y Saito (Eds.). Economic Research Institute for ASEAN and East Asia.

S13. Natividad JN. (2019). The 2007 Philippine Study on Aging. In Encyclopedia of Gerontology and Population Aging (pp. 1-6). Springer, Cham.

S14. Oh K, Kim Y, Kweon S, et al. Korea National Health and Nutrition Examination Survey, 20th anniversary: accomplishments and future directions. *Epidemiol Health* 2021; **43:** e2021025.

S15. Lee J. KLoSA—Korean Longitudinal Study of Aging. *Korean J Fam Med*. 2020; **41:** 1–2.

S16. Malhotra R, Ang S, Allen JC, et al. Normative Values of Hand Grip Strength for Elderly Singaporeans Aged 60 to 89 Years: A Cross-Sectional Study. *J Am Med Dir Assoc*. 2016; **17**: 864.e1-7.

S17. Gupta P, Man REK, Fenwick EK, et al. Rationale and Methodology of The PopulatION HEalth and Eye Disease PRofile in Elderly Singaporeans Study [PIONEER]. *Aging Dis*. 2020; **11**: 1444-1458.

S18. Ong HL, Abdin E, Chua BY, et al. Hand-grip strength among older adults in Singapore: a comparison with international norms and associative factors. *BMC Geriatr*. 2017; **17:** 176.

S19. Rannan-Eliya RP, Dissanayake VH, Perera P, et al. Cohort Profile: The Sri Lanka Health and Ageing Study (SLHAS). *Int J Epidemiol*. 2024; **53:** dyae044.
